# Benefits and Harms of Antenatal/Intrapartum Screening for Maternal Group B Streptococcus and Use of Intrapartum Antibiotic Prophylaxis Versus Risk‐Based Protocols or No Intervention: A Rapid Review

**DOI:** 10.1111/apa.70568

**Published:** 2026-04-30

**Authors:** Pauline Campbell, Cathryn Broderick, Candida Fenton, Bridget Davis, Wei Xu, Alex Todhunter‐Brown, Narendra Aladangady, Richard F. Chin, Rosie Hill, Ryan Kean, Charlotte‐Eve Short, Julie Cowie, NESSIE—NIHR Evidence Synthesis Scotland InitiativE

**Affiliations:** ^1^ Research Centre for Health (ReaCH) Department of Nursing, Community and Public Health, School of Health and Life Sciences Glasgow Caledonian University Glasgow Scotland; ^2^ NESSIE—NIHR Evidence Synthesis Scotland InitiativE, Usher Institute, Usher Building The University of Edinburgh Edinburgh Scotland; ^3^ Homerton University Hospital London England; ^4^ The Muir Maxwell Epilepsy Centre The University of Edinburgh Edinburgh Scotland; ^5^ Royal Hospital for Children and Young People Edinburgh Scotland; ^6^ Department of Biological and Biomedical Sciences Glasgow Caledonian University Glasgow Scotland; ^7^ Department of Infectious Disease Imperial College London London England; ^8^ Yunus Centre Glasgow Caledonian University Glasgow Scotland

## Abstract

**Aim:**

To synthesise evidence on the effectiveness, harms and benefits of different approaches to prevent early‐onset Group B Streptococcus (EOGBS) and identify gaps in short and longer‐term outcomes.

**Methods:**

A two‐phase rapid review. Phase 1 included an overview of systematic reviews (SRs). Phase 2 identified primary studies from SRs supplemented by additional searches. Outcomes included screening effectiveness, maternal and neonatal health outcomes and reported harms or benefits.

**Results:**

Phase 1 identified three moderate‐high quality reviews; 78 primary studies met phase 2 eligibility criteria. Any prevention strategy reduced EOGBS incidence, all‐cause early‐onset sepsis (EOS) and EOGBS‐related mortality compared to no strategy. Universal screening was more effective in reducing EOGBS and all‐cause EOS compared with risk‐based approaches, with no evidence of any difference between the approaches for non‐GBS EOS incidence or EOGBS‐related mortality. Evidence was low to very‐low certainty. Other neonatal outcomes (meningitis, pneumonia, late‐onset GBS or maternal outcomes) were limited. Long‐term child outcomes were under‐reported. Few studies reported women's views. Protocol violations and missed opportunities were reported.

**Conclusion:**

Any prevention strategy reduces the incidence of EOGBS, all‐cause EOS and EOGBS‐related mortality compared to no strategy. Differences between universal and risk‐based approaches remain unclear. Improved reporting, longer‐term evaluation and implementation‐focused research are needed.

## Summary

- There is no consensus on the optimal screening approach for the prevention of early‐onset Group B Streptococcus (GBS), and concerns remain about overtreatment with intrapartum antibiotic prophylaxis and potential neonatal impacts.
- Any prevention approach, universal or risk‐based, reduces EOGBS incidence compared to no strategy.
- Reporting of short‐term neonatal and long‐term outcomes is limited and further high‐quality research is needed.

**Abbreviations**
EOGBS: early‐onset Group B Streptococcal infection
EOS: early‐onset sepsis
GBS: group B streptococcus
IAP: intrapartum antibiotic prophylaxis
LMIC: low‐ or middle‐income country
MA: meta‐analysis
RCT: randomised‐controlled trial
RR: risk ratio
SR: systematic review

## 1 Introduction

Group B Streptococcus (GBS) is a leading cause of neonatal morbidity and mortality, contributing to early‐onset sepsis, pneumonia and meningitis [1]. In 2017, the global incidence of early‐onset GBS was 0.41 per 1000 live births [2]. Intrapartum antibiotic prophylaxis (IAP) is widely used to prevent GBS transmission from the pregnant woman^1^ to the neonate. Worldwide, three approaches are used to identify women who may be eligible for IAP to prevent GBS: (1) universal screening using antenatal cultures or rapid intrapartum PCR tests; (2) risk‐based approach based on the clinical assessment of factors such as preterm labour, fever, prolonged rupture of membranes, or prior history of GBS; or (3) no strategy, in which the decision to administer IAP is based on the individual case. There is no clear consensus on which screening strategy is optimal, and concerns have been raised about the potential for overtreatment along with potential impacts on the neonate, the emergence of antimicrobial resistance [3], and uncertain long‐term effects on child health [4, 5]. Consideration of women's perceptions around different screening strategies and IAP is also often overlooked [6, 7, 8].

Here, we report our findings from a two‐phase rapid review. In phase 1, we present evidence from high‐quality systematic reviews summarising the effectiveness of different screening approaches in reducing the incidence of EOGBS infections, mortality rates, antimicrobial infections, IAP exposure rates and other neonatal infections. In phase 2, we examine the evidence from primary studies conducted in high‐income countries to identify the benefits and harms of different screening strategies and to document any other neonatal and maternal outcomes not previously reported in the existing systematic reviews.

## 2 Methods

Our methods are described in detail in a prospectively registered protocol [9]. Key definitions used throughout this paper are presented in Data S1. A small ‘Reference group’ was formed to provide content expertise. Details of the Reference Group involvement are reported according to the ACTIVE framework [10] in Data S2.

In brief, we conducted a two‐phase rapid review using established methods [11] and following reporting guidelines [12]. In phase 1, we conducted systematic searches of two electronic databases (Medline, Embase) from 1 January to 26 February 2025 to identify high‐quality SR/MA that compared different screening approaches. Search strategies are provided in Data S3. Two reviewers independently screened titles, abstracts and assessed full text papers and conducted quality assessment using the AMSTAR‐2 tool. We excluded SR/MAs if they were not published in English or were judged as having a high risk of bias in any of the AMSTAR‐2 critical domains [13]. We resolved disagreements through team consensus meetings.

In phase 2 we conducted a targeted search of Medline and Embase (1 January 2019–11 March 2025) to identify any additional relevant primary studies (with a comparator group) that may have been published after the SR/MAs in phase 1. Studies were screened by two independent reviewers and excluded if they were not in English, lacked a comparator, or were conducted in low‐ or middle‐income (LMIC) countries due to potential contextual differences in infrastructure and available resources.

Data extraction in both phases was conducted by one reviewer and cross‐checked by a second reviewer. In phase 1, we extracted outcomes related to effectiveness of screening strategies (e.g., incidence of EOGBS infection, mortality and case‐fatality rate, incidence of other cause infection, antimicrobial resistance, IAP exposure rates and timing of GBS determination). In phase 2, we focused on extracting data not addressed in the SRs/MAs including neonatal health outcomes (e.g., meningitis, pneumonia, incidence of late‐onset GBS (LOGBS); maternal health outcomes (e.g., anaphylaxis, adverse events)) and any other data related to the potential harms and benefits of screening.

Quality assessment of cohort studies was performed using the ROBINS‐I tool (v1) [14] and RCTs were assessed using the CASP‐RCT checklist [15]. Where an existing ROBINS‐I assessment was conducted by one of the included systematic reviews, this assessment is reported. Where more than one review has conducted a risk of bias assessment, both assessments have been considered and are presented. Where additional information was needed, we attempted to contact review or study authors.

## 3 Results

### 3.1 Phase 1: Overview of Reviews

**FIGURE 1:**
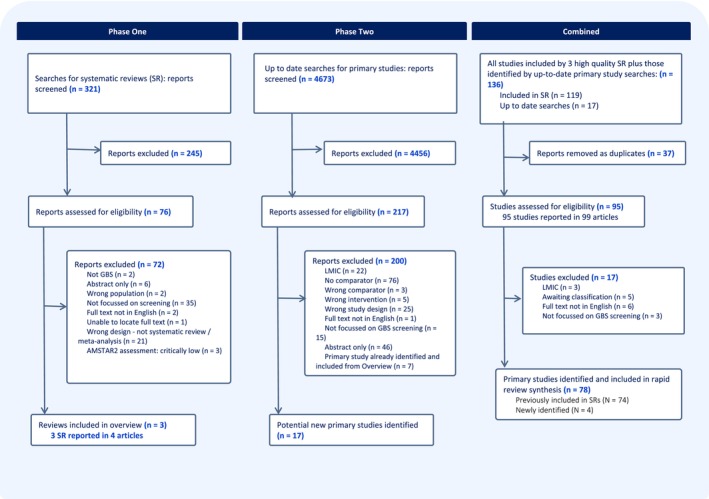
PRISMA flowchart. The search was conducted in two phases. Phase 1 focused on identifying relevant SRs using a scoping search, with key reviews selected by the authors to inform a targeted search (restricted to English‐language and published from 2016). Phase 2 involved a search for primary studies in Medline and Embase (from 1 January 2019 to 11 March 2025) for English language publications. Details of the searches are included in Data S4. The combined PRISMA outlines the overall flow of literature across the rapid review.

Six SRs/MAs met the selection criteria [3, 16, 17, 18, 19, 20]. Of these, three [3, 16, 17] were excluded as they were judged to be critically low quality on AMSTAR‐2 [13] (Data S4). Reasons for exclusion are provided in Figure 1 and Data S5.

Key characteristics of the three high quality systematic reviews (reported across four articles) [18, 19, 20, 21] are summarised in Table 1. There was significant overlap in the primary studies reported in the reviews (see Data S6 and S7). At the level of overview of reviews, the definitions of approaches varied, and gestational age and participant characteristics were poorly reported. When assessed, the certainty of the evidence for these outcomes was low or very low. Table 2 provides a summary of key findings from the three SRs/MAs comparing outcomes across screening approaches. Further details are provided in Data [Link], [Link], [Link].

**TABLE 1:** Table of characteristics of included systematic reviews.

	Hasperhoven 2020	Li 2020	Panneflek 2024
Protocol registration	CRD42019127633	NR	CRD42023411806
Aim (verbatim)	To determine the relative success of screening‐based and risk‐based protocols in preventing EOGBS disease in newborn infants	To compare the effects of these 2 strategies in reducing the incidence of early‐onset GBS sepsis (GBS‐EOS) and their effects on the incidence of non‐GBS sepsis	To provide up‐to‐date evidence on effectiveness of different strategies by comparing perinatal outcomes
Definition of EOGBS	GBS culture from a normally sterile site < 7 days of age	Referred to as GBS‐EOS. EOS determined by positive blood, CSF, or other sterile fluids culture, within 72 h or 7 days after birth	GBS detected within 7 days of birth in normally sterile fluids, such as blood and CSF
Definition of Universal screening	The Centers for Disease Control (CDC) recommend universal screening for maternal colonisation between 36 and 38 weeks of pregnancy	The screening‐based strategy refers to the method of IAP when observing GBS colonisation by using either molecular tests (i.e., PCR testing) or microbiologic methods [i.e., selective medium inoculated with sample from vaginal or vaginal‐rectal swabs in the third trimester (≥ 28 weeks of gestation)]	Defined as strategies where GBS colonisation was determined using microbiological testing and IAP was administered to all women positive for GBS colonisation
Definition of risk‐based approach	Clinical indicators include prolonged rupture of membranes, bacteriuria, an earlier child with EOGBS, and maternal fever	The risk‐based strategy refers to the method of IAP based on prenatal risk factors (i.e., delivery at < 37 weeks of gestation, rupture of the membranes > 18 h duration, intrapartum temperature > 38.0°C, GBS bacteriuria during this pregnancy, previous infant with invasive GBS disease, etc.) that may increase the risk of EOS	IAP was administered according to the presence of any risk factor for EOGBS during pregnancy, such as a history of an infant with EOGBS infection, presence of GBS bacteriuria during pregnancy, or presence of intrapartum fever, preterm labour or prolonged rupture of membranes > 18 h
Definition of no strategy or no policy	‘No policy’ defined as a situation in which no consistent protocol was used but IAP could have been administered on an individual basis	NR	IAP was sometimes found to be administered without any official screening (‘no’) strategy on an individual basis and at the discretion of the attending physician
Definition of ‘other’ strategies	NA	NA	Defined as strategies where IAP was administered based on any combination of elements from risk‐based and universal strategies. Typically consisted of strategies where both risk‐based and universal strategies were implemented in parallel and selective strategies that only treat pregnant women positive for GBS colonisation with the presence of least one risk
Search date	March 2019	December 2018	May 2024
Language or setting restrictions	English and Dutch	English language	None
Number of included studies (included in MA)	17 (15)	18 (18)	72 (48)
Quality assessment tool and findings	ROBINS‐I Overall: 10/17 moderate; moderate to serious 2/17; serious 3/17; critical 1/17; impossible to define 1/17	Newcastle‐ Ottawa Scale Overall: 11/18 high quality; 7/18 low quality	ROBINS‐I Overall: low 1/72; moderate 22/72; moderate to serious 3/72; serious 43/72; critical 3/72
Countries in which studies conducted	*N* = 10 countries (Australia, USA, UK, Hong Kong, Italy, Turkey, Netherlands, New Zealand, Sweden, Taiwan)	NR ‘Most studies included in the meta‐analysis were from Europe and United States’	*N* = 30 countries (Qatar, Saudi Arabia, Spain, Australia, USA, Netherlands, Finland, Iceland, China, Taiwan, New Zealand, Brazil, UK, Austria, Sweden, South Korea, Hungary, Spain, France, Japan, Ireland, Denmark, Switzerland, Israel, Czech Republic, Germany, India, Chile, Italy, Turkey)
Total number of neonates within included studies	11 M	604 869	Over 10 M

Abbreviations: CSF, cerebrospinal fluid; EOGBS, early‐onset GBS; EOS, early‐onset sepsis; GBS, Group B streptococci; h, hours; IAP, intrapartum antibiotic prophylaxis; M, million; MA, meta‐analysis; NA, not applicable; NR, not reported; PCR, polymerase chain reaction; RCT, randomised controlled trials.

**TABLE 2:** Evidence from high quality SRs/MAs identified in phase 1 comparing the effectiveness of different screening approaches for GBS.

Outcome	Strategy	SR ref	Studies (*n*)	Participants (*n*)	Effect RR (95% CI) *p* value (unless otherwise stated)	*I* ^2^ (%)	GRADE certainty of evidence^h^
EOGBS incidence: All births^a^	Any versus no strategy	[19]	34	10 041 490	RR 0.46 (0.36–0.60), *P* = NR	93	Very low
	Universal versus no strategy	[21]	4	3 172 204	RR 0.31 (0.11–0.84), *p* = 0.021	90.9	Not assessed
		[19]	16	1 579 173	RR 0.37 (0.25–0.55), *P* = NR	78	Very low
	Risk‐based versus no strategy	[21]	7	7 506 263	RR 0.86 (0.61–1.20), *p* = 0.382	89.36	Not assessed
		[19]	11	3 004 723	RR 0.65 (0.48–0.87), *P* = NR	82	Very low
	Universal versus risk‐based approach	[21]	10	931 794	RR 0.43 (0.32–0.56), *p* < 0.00001	13	Not assessed
		[20]	18	604 869	RR 0.45 (0.34–0.59), *p* = 0.02	45	Not assessed
		[19]	17	1 806 092	RR 0.41 (0.30–0.55), *P* = NR	60	Low
Incidence of EOGBS infection: Term‐deliveries only^a^	Any versus no strategy	[19]	6	943 373	RR 0.34 (0.24–0.48), *P* = NR	11	Low
	Universal versus no strategy	[19]	3	490 024	RR 0.26 (0.13–0.54), *P* = NR	9	Low
	Risk‐based versus no strategy	[19]	1	46 959	RR 0.25 (0.10–0.59), *P* = NR	NA	Low
	Universal versus risk‐based approach	[19]	8	709 956	RR 0.29 (0.17–0.51), *P* = NR	37	Low
EOGBS infection related mortality incidence^b^, ^c^	Any versus no strategy	[19]	Any: 19 No strategy: 15	NR	Any: 0.028 (0.022–0.036), *P* = NR No strategy: 0.089 (0.047–0.17), *P* = NR	Any: 0 No strategy: 86	Not assessed
	Universal versus risk‐based approach	[19]	Risk‐based: 6 Universal: 10	NR	Risk‐based: 0.026 (0.019–0.037), *P* = NR Universal: 0.028 (0.015–0.054), *P* = NR	Risk‐based: 36 Universal: 0	Not assessed
EOGBS CFR^b^, ^c^	Any versus no strategy	[19]	Any: 20 No strategy: 17	NR	Any: 6% (5%–8%), *P* = ? No strategy: 10% (7%–15%), *P* = NR	Any: 7 No strategy: 71	NR
	Universal versus risk‐based approach	[19]	Risk‐based: 8 Universal: 11	NR	Risk‐based: 5% (4%–7%), *P* = NR Universal: 4% (3%–6%), *P* = NR	Risk‐based: 0 Universal: 0	Not assessed
All cause EOS^c^	Any versus no strategy	[19]	14	3 878 681	RR 0.60 (0.48–0.74), P NR	85	Very low
	Universal versus no strategy	[19]	7	2 120 056	RR 0.60 (0.45–0.80), P NR	78	Very low
	Risk‐based versus no strategy	[19]	6	347 112	RR 0.73 (0.61–0.89), P NR	13	Low
	Screening versus Risk‐based	[20]	4	187 994	RR 0.78 (0.62–0.98), P NR	38	Not assessed
	Universal versus risk‐based approach	[19]	6	674 033	RR 0.72 (0.65–0.80), P NR	44	Low
*E. coli* GBS^c^	Screening versus Risk‐based	[20]	4	187 994	RR 0.98 (0.69–1.40), P NR	0	Not assessed
Non‐GBS EOS^c^	Any versus no strategy	[19]	13	1 357 432	RR 0.75 (0.56–0.99), P NR	68	Low
	Universal versus no strategy	[19]	6	395 440	RR 0.86 (0.71–1.04), P NR	2	Very low
	Risk‐based versus no strategy	[19]	6	347 112	RR 0.76 (0.51–1.14), P NR	69	Very low
	Screening versus Risk‐based	[20]	7	280 896	RR 0.91 (0.74–1.11), P NR	18	Not assessed
	Universal versus risk‐based approach	[19]	6	674 033	RR 0.93 (0.82–1.05), P NR	0	Very low
Antimicrobial resistance to GBS isolates^d^	All strategies	[21]	Penicillin/ampicillin: 5 Erythromycin: 4 Clindamycin: 2	NR	No resistance–penicillin/ampicillin Resistance–erythromycin (WM: 19%), and clindamycin (WM: 16%)	NA	NR
		[19]	Penicillin, ampicillin, or β‐lactams: 11	NR	No resistance–penicillin, ampicillin, or β‐lactams. Varying levels of resistance were observed–erythromycin and clindamycin. No resistance–vancomycin was reported.	NA	NR
Antimicrobial resistance to ampicillin‐resistant *E. coli* in EOS^d^	Screening and risk‐based approaches	[20]	3	170 807	RR 1.28 (0.74–2.21), *P* = NR	0	NR
IAP exposure rates^e^	Any versus no strategy	[19]	No strategy (3 studies) Any strategy (16 studies)	NR	Pooled IAP rate more than doubled when comparing periods with no strategy 8% (3%–17%)) and any strategy (19% (16%–22%)	NA	NA
	Universal versus risk‐based	[21]	Universal screening (4 studies) Risk‐based (3 studies)	NR	Weighted mean IAP exposure was 31% in universal screening versus 29% in risk‐based	NA	NR
		[19]	9	514 023	RR 1.29 (0.95–1.75), *P* = NR	99	Very low
Missed EOGBS cases not indicated by positive screening or detection of risk factors (i.e., no IAP administered)^f^		[21]	13	6 409 097	Risk‐based missed: 41.3% of neonates with EOGBS were born to mothers without risk factors Universal screening missed, 24.2% of EOGBS cases occurred in neonates born to mothers with negative cultures	NA	NA
Timing of GBS determination on EOGBS infection^g^	26–28 weeks versus 35–37 weeks' gestation	[19]	1	13 754	RR 1.24 (0.25–6.12), *P* = NR	NA	Very low
	Intrapartum versus antenatal screening	[19]	1	30 798	RR 0.21 (0.07–0.64), *P* = NR	NA	Moderate

Abbreviations: ß‐lactams, beta‐lactam antibiotics; CFR, case‐fatality rate; CI, confidence interval; EOGBS, early‐onset GBS; EOS, early onset sepsis; GBS, group B Streptococcus; *I* ^2^ , statistical measure of heterogeneity; IAP, intrapartum antibiotic prophylaxis; MA, meta‐analysis; *n*, number of studies; NA, not applicable; NR, not reported; *p*, *p* value; RR, risk ratio; S, Supporting Information file; SR, systematic review; WM, weighted mean.

^a^ Additional detail provided in Data S8.

^b^ Mortality and CFR not reported for universal versus no strategy or risk‐based versus no strategy Data S9.

^c^ Additional detail provided in Data S10.

^d^ Additional detail provided in Data S11.

^e^ Additional detail provided in Data S12.

^f^ Additional detail provided in Data S14.

^g^ Additional detail provided in Data S18.

^h^ GRADE judgements as reported by review authors (see Data [Link], [Link], [Link], [Link], [Link]).

#### 3.1.1 Any Prevention Strategy (Universal/Risk‐Based or Other) Versus No Strategy

One review [19] showed that any prevention strategy (universal/risk‐based or other) reduced the EOGBS incidence compared with no strategy, including only term deliveries. Any strategy also reduced the risk of all‐cause EOS and non‐GBS EOS. EOGBS infection related mortality rate was lower with any screening strategy compared to no strategy.

#### 3.1.2 Universal Screening Versus No Strategy

Two reviews [19, 21] reported that universal screening reduced EOGBS incidence compared with no strategy. This was also the case for the subgroup of term‐only deliveries. Universal approaches were also associated with a significant reduction in all‐cause EOS, but neither review found a significant reduction in non‐GBS EOS incidence. Mortality outcomes were not reported for this comparison.

#### 3.1.3 Risk‐Based Approach Versus No Strategy

Conflicting results were reported for the incidence of EOGBS infection. Hasperhoven 2020 [21] found no difference between risk‐based approach and no strategy; however, Panneflek 2024 [19] reported a reduction in EOGBS infection with a risk‐based approach. The incidence of all‐cause EOS was reduced with risk‐based approaches compared to no strategy, but no significant difference for non‐GBS EOS incidence. Mortality outcomes were not reported.

#### 3.1.4 Universal Screening Versus Risk‐Based Approaches

All three reviews [19, 20, 21] reported that universal screening reduced EOGBS incidence compared to risk‐based approaches. Two reviews [19, 20] also reported a reduction in all‐cause EOS incidence for the universal screening strategy compared with risk‐based approaches, but no significant difference for non‐GBS EOS incidence. Mortality and case‐fatality rates did not differ between strategies.

Antimicrobial resistance and IAP exposure rates were also reported, and findings are presented in Table 2 and Data S11 and S12 respectively. Missed cases in which no IAP was administered are provided in Data S13.

### 3.2 Phase 2: Findings From Primary Studies Conducted in High‐Income Countries

Seventy‐eight primary studies met the eligibility criteria (Summarised in Data S14): 74/78 were identified from the three reviews [19, 20, 21] identified in phase one, and four new studies from phase 2 searches [22, 23, 24, 25] (Figure 1).

### 3.3 Risk of Bias Assessment

#### 3.3.1 Randomised Control Trials

The quality appraisal of the two newly identified RCTs is presented in Data S4 [24, 26]. Both studies were judged as having potential risk of bias due to the lack of blinding of participants, providers and outcome assessors. Additionally, generalisability was limited; Kolkman 2020 [24] reported no relevant outcomes, and the Daniels 2022 study [26] only included women with risk factors.

#### 3.3.2 Non‐Randomised Primary Studies

Of the 76 studies evaluated:

- 2 studies were judged as having a critical risk of bias in one or more domains [27, 28].
- 46 studies were judged to have a serious risk of bias overall.
- 27 studies were judged as having moderate risk of bias overall.
- 1 study was judged as low risk of bias [29].

Risk of bias was due to confounding factors, missing data and reporting of outcome measures. Due to the presence of risk of bias, results should be interpreted cautiously, as biases directly impact the validity of findings and subsequently conclusions drawn in the review.

#### 3.3.3 Participant Characteristics

The number of participants was reported in 41/78 studies (*n* = 2 087 687). The reporting of key characteristics was inconsistent with ethnicity reported in 30/78 studies and the GBS colonisation rates in 37/78. EOGBS was defined in most studies (65/78) but only 19/78 studies provided a definition for LOGBS. Gestational age was poorly reported with only 16/78 studies providing this data. Further participant details are provided in Data S15 and the different screening strategies provided are presented in Data S16.

#### 3.3.4 IAP Administration

All the studies administered IAP with 54/78 studies reporting the type of antibiotic (see Data S14), with most delivering penicillin or ampicillin. Thirty‐one studies reported the dose, and 24/78 studies reported alternatives for penicillin allergy.

#### 3.3.5 IAP Exposure Rates

Two new studies reported IAP exposure rates and found no difference in the proportion of women receiving IAP between universal and risk‐based approaches, or between rapid intrapartum PCR testing and risk‐based screening (see Data S17).

#### 3.3.6 Timing of GBS Screening

Sixty studies reported the GBS screening timing. Of these, 15/60 stated adherence to guideline recommendations. Eighteen studies provided no details about screening timing. Of those that reported timing, most screened at 35–37 weeks' gestation. Further details are available in Data S18 and S19.

#### 3.3.7 Neonatal Health Outcomes

Meningitis was reported in 21 studies; of these five studies stratified their data according to screening (see Data S20):

- No screening policy versus other strategy (*n* = 2 studies) [30, 31]. Trijbels‐Smeulders 2007 [31] found no difference in the corrected incidence of proven GBS meningitis (0.14 vs. 0.17 per 1000 live births). Lukacs and Schrag 2012 [30] reported no significant differences in the percentage of meningitis‐related hospitalisations.
- No screening policy versus risk‐based approach (*n* = 2 studies) [32, 33]. Håkansson 2017 [32] reported the total number of verified cases of sepsis/meningitis for each strategy, and O'Sullivan 2019 [33] reported a similar incidence of GBS meningitis (0.15 vs. 0.17 per 1000 live births).
- Risk‐based approach versus universal screening strategy (*n* = 1 study) [34]. Gopal‐Rao 2017 [34] reported no cases of meningitis in the risk‐based group compared with one case in the universal screening group.
- Universal strategy versus other screening strategy (*n* = 1 study) [30]. Lukacs and Schrag 2012 reported no significant differences in the percentage of hospitalisations with meningitis.

Early‐onset pneumonia outcomes were described in eight studies (10%). Two studies reported pneumonia cases associated with LOGBS infection [35, 36] (See Data S21). Encephalopathy was not reported in any study. Other outcomes, such as respiratory issues, the need for additional oxygen support, urinary tract infections and cellulitis, were also reported and are detailed in Data S22.

Incidence of LOGBS was poorly reported, with only five studies providing information on numbers of cases or incidence by approach [36, 37, 38, 39, 40].

#### 3.3.8 Maternal Health Outcomes

Various maternal peripartum infections were reported across the included studies (*n* = 16/78). Of these, chorioamnionitis was the most frequently reported. Anaphylaxis outcomes were reported in nine studies (9/78; 12%). Of these, six did not provide data stratified by screening strategy. Only one case of anaphylaxis was reported in these nine studies, with no details provided on the IAP agent involved (Data S25). Nine studies reported adverse events. Of these, six reported no adverse events, and three studies reported a post‐partum rash [41, 42, 43] (Data S23).

#### 3.3.9 Longer‐Term Outcomes

There was limited data on the medium and long‐term outcomes reported across the included studies (see Data S23). Long‐term complications including cerebral palsy, epilepsy, hearing loss, learning disabilities and developmental delay were described in seven studies (9%) (see Data S23).

#### 3.3.10 Harms and Benefits

Several studies described the benefits of reducing or preventing EOGBS, and shorter length of hospital stay and related costs [23, 44, 45, 46]. One study described that screening enabled ‘informed decision‐making with opportunity for counselling’ [29].

Studies also described harms for both mothers and neonates, and included the following:

- False negative GBS‐carriers were reported in 8 studies. However, the incidence of false negatives, or true negatives and positives at delivery, varied widely and was inconsistently reported across screening approaches.
- Health outcomes related to exposure to antibiotics during labour were poorly reported and primarily related to postpartum rashes. One study noted an increase in antibiotic‐resistant *E. coli* infections among preterm infants compared to term, while incidence of *E. coli* overall remained stable [47].
- Poor adherence to screening protocols or guidelines potentially leading to undertreatment [24, 48] or overtreatment [24, 29, 49].

Screening limitations were frequently reported and included missed opportunities for prevention which may occur as screening protocols cannot always predict GBS status at delivery due to timing of screening and transient colonisation, and therefore IAP may not be administered to mothers who may be GBS carriers. Protocol compliance and/or violations which were usually implementation issues (e.g., failure to give indicated IAP or conversely administering IAP when not indicated) were also frequently reported. Protocol violations were usually implementation issues which involved failure to give indicated IAP or conversely administering IAP when not indicated. Compliance with protocol by the mothers was rarely reported, with only one study clearly reporting one case of a woman refusing IAP [50]. Further details of harms and benefits are provided in Data S24.

## 4 Discussion

### 4.1 Summary of Main Findings

In the first phase of our review, we identified three moderate‐high quality SRs/MAs which compared different screening approaches. The definitions of screening approaches varied widely across the reviews, and gestational age, participant characteristics (e.g., ethnicity, maternal obesity), timing and methods of GBS were poorly reported. There was a clear consensus across the reviews that the implementation of any prevention strategy (universal or risk‐based) reduced the incidence of EOGBS infection compared with no strategy. Universal screening further reduced EOGBS incidence compared with risk‐based screening. Employing any strategy approach also reduced all‐cause EOS, and universal screening further reduced all cause EOS compared to risk‐based approaches. For non‐GBS infection, any strategy reduced the incidence compared with no strategy, but there was no difference in the incidence for either universal or risk‐based approaches. Any strategy also reduced EOGBS‐related mortality compared with no strategy, but no difference in mortality was evident when comparing universal and risk‐based approaches. The certainty of evidence was judged as very‐low to low.

Our review found no difference in exposure to antibiotic resistance between universal or risk‐based approaches, and GBS isolates showed little antibiotic resistance, although one study reported higher rates of antibiotic‐resistant *E. coli* infections in preterm deliveries.

Reporting of harms and benefits in the primary studies was limited. The studies described a reduction in EOGBS incidence as the primary benefit of screening. Other studies also reported the cost‐effectiveness of scarce healthcare resources, including a shorter length of hospital stay. Harms included reports of false negatives and poor adherence to screening protocols. Several studies also described implementation challenges which may have resulted in over‐treatment or under‐treatment. Failure to provide or administer the appropriate IAP coupled with missed opportunities for prevention were common. However, these benefits and harms were spread across 14 different types of screening comparisons, making it difficult to draw any clear conclusions about the additional harms and benefits of one screening approach compared with another.

Our review also found insufficient evidence to draw any clear conclusions about neonatal outcomes such as meningitis, pneumonia or LOGBS by screening approach, limiting our ability to draw any meaningful conclusions. Long‐term child health outcomes were described in less than 10% of primary studies. This is an important gap in the evidence, as a recent meta‐analysis of observational studies reported that IAP exposure is associated with a modest increase in BMI and increased risk of autoimmune disease [51]. While the Moradi meta‐analysis [51] does not compare different screening approaches, it highlights the possibility that greater IAP exposure may have wider clinical implications beyond the neonatal period. This supports the need for studies comparing screening approaches to incorporate longer‐term follow‐up to assess potential downstream harms. Follow up of babies born to women in the ongoing cluster‐randomised GBS3 trial [52] presents a unique opportunity to investigate any long‐term impacts of intrapartum exposure to antibiotics. Finally, our review found that less than one in four studies reported maternal health outcomes and only one study reported women's views or perspectives about the different screening approaches [53]. Recent qualitative evidence shows that women are keen to know if they could choose the best timepoint for themselves if the testing was introduced routinely, while others valued healthcare providers' opinions on what would work best for them [8].

### 4.2 Strengths and Limitations

Our review followed established methods for conducting rapid reviews and provides a high‐quality summary of the current evidence on the effectiveness of different screening strategies. It also brings together important evidence related to short and longer‐term neonatal and maternal health outcomes, highlighting several evidence gaps.

Our review has several limitations. Our searches were limited to two databases, which may mean that some relevant studies were inadvertently missed. Phase 1 searches involved systematic identification of studies from high‐quality reviews; however, none of these reviews examined outcomes beyond seven days. While we supplemented our searches for additional primary studies from 2019, we may have missed some relevant studies reporting long‐term outcomes. We only included studies published in English and limited phase 2 to primary studies from high‐income settings. This limits the generalisability of our findings.

We did not plan or conduct any meta‐analyses, and as such, the findings of the four new studies have not been integrated with those from earlier meta‐analyses. However, we do not believe these would impact the findings of the systematic reviews. Finally, there are setting specific differences in strategies classified as universal screening or risk‐based approaches due to factors such as time‐period (i.e., early or late antenatal and intrapartum), culture and definitions. These differences could have resulted in comparisons of strategies that were, in some cases, not consistent in definition nor implementation. We have attempted to account for this variation (see Data S16), but caution is needed with interpreting the findings.

### 4.3 Clinical Implications

Our review identified several clear benefits supporting the implementation of any type of screening strategy compared with no screening strategy, including a reduction in EOGBS incidence, all‐cause EOS and mortality. Universal screening approaches were found to be more effective at reducing EOGBS infection incidence and all‐cause EOS compared with risk‐based approaches, although there was no difference observed for non‐GBS incidence or mortality. Our review highlighted the substantial variation in screening methods, and we found limited data on maternal and long‐term neonatal outcomes. Women's perspectives about the screening approaches were largely absent, and real‐world implementation challenges further complicated our ability to identify an optimal screening approach.

### 4.4 Implications for Future Research

Current evidence relays heavily on non‐randomised comparative studies reporting varying and often poorly described approaches. Even with larger studies, the total number of events for outcomes such as EOGBS related mortality may be insufficient to detect differences between approaches. Our review has highlighted several evidence gaps. First, the concerns about potential longer‐term effects of IAP, including any impact on antimicrobial resistance, have not been addressed. Big data approaches using data‐linkage methodology or individual participant level data are needed to evaluate these uncertainties and evaluate medium‐ and longer‐term maternal and neonatal outcomes. Future research should also aim to improve the reporting of key demographic factors and outcomes. A key priority is the urgent need to improve adherence to existing screening approaches. Several primary studies described avoidable protocol violations because of system‐level and workflow challenges. Future research should consider using an implementation science lens to identify factors that affect the delivery of IAP in different screening approaches. Finally, qualitative research that explores women's perspectives on screening approaches and IAP should be embedded in future studies.

## Author Contributions

**Narendra Aladangady:** writing – review and editing. **Bridget Davis:** writing – review and editing, conceptualisation, methodology. **Candida Fenton:** methodology, conceptualisation, software, writing – review and editing, data curation. **Wei Xu:** writing – review and editing, conceptualisation, methodology. **Cathryn Broderick:** investigation, writing – review and editing, visualisation, formal analysis, conceptualisation, methodology, project administration, data curation, validation, writing – original draft. **Pauline Campbell:** writing – original draft, writing – review and editing, supervision, data curation, formal analysis, investigation, methodology, visualisation, project administration, conceptualisation, validation. **Alex Todhunter‐Brown:** methodology, writing – review and editing, supervision, conceptualisation, project administration. **Richard F. Chin:** writing – review and editing. **Rosie Hill:** writing – review and editing, conceptualisation, methodology. **Ryan Kean:** writing – review and editing. **Charlotte‐Eve Short:** writing – review and editing. **Julie Cowie:** writing – original draft, writing – review and editing, supervision, data curation, formal analysis, investigation, methodology, visualisation, project administration, conceptualisation, validation.

## Funding

This study is being conducted by NIHR Evidence Synthesis Scotland InitiativE (NESSIE), which is funded by the NIHR Evidence Synthesis Programme (ESP; NIHR153425; study ID NIHR176047). The funder has had no role in conducting the research and preparation of the manuscript.

## Conflicts of Interest

R.F.C. has provided paid consultancy for Zogenix, Red Nucleus, Jazz and GW Pharma (now part of Jazz) and received speaker fees from UCB, Eisai, Zogenix and GW Pharma. The other authors declare no conflicts of interest.

## Supporting information


**Data S1:** Key definitions.

**Data S2:** Reference group involvement.

**Data S3:** Search strategy and report.

**Data S4:** Quality assessments.

**Data S5:** Excluded studies.

**Data S6:** GROOVE overlap.

**Data S7:** Overlap of primary studies within included reviews.

**Data S8:** EOGBS infection: Summary of meta‐analysis and GRADE judgements.

**Data S9:** EOGBS mortality and fatality: Summary of meta‐analysis and GRADE judgements.

**Data S10:** Other infection: Summary of meta‐analysis and GRADE judgements.

**Data S11:** Antimicrobial resistance: Summary of meta‐analysis and GRADE judgements.

**Data S12:** IAP exposure: Summary of meta‐analysis and GRADE judgements.

**Data S13:** Summary of missed cases.

**Data S14:** Key characteristics: Primary study level.

**Data S15:** Demographics: Primary study level.

**Data S16:** Strategies compared in primary studies.

**Data S17:** IAP exposure: primary study level.

**Data S18:** Timing of determination: summary of meta‐analysis and GRADE judgements.

**Data S19:** Timing of maternal GBS screening: primary study level.

**Data S20:** Meningitis: Neonatal health outcomes.

**Data S21:** Pneumonia: Neonatal health outcomes.

**Data S22:** Other: Neonatal health outcomes.

**Data S23:** Maternal health outcomes.

**Data S24:** Harms and benefits.

## Data Availability

The data that support the findings of this study are available from the corresponding author upon reasonable request.

## Endnotes

^1^
In line with the UK Network of Professors of Midwifery and Maternal and Newborn Health, we use the words women and woman throughout this protocol, recognising that this reflects the biology and identity of the great majority of those who are childbearing; for the purpose of this protocol, these terms include girls, and people whose identity does not correspond with their birth sex or who may have a non‐binary identity 3. UK Network of Professors of Midwifery and Maternal and Newborn Health. *Position Statement: Use of sexed language in relation to women's reproductive health*. UK: Council of Deans of Health; 2023. URL: https://www.councilofdeans.org.uk/2024/02/midwifery‐network‐position‐paper‐use‐of‐sexed‐language/ (accessed 27 January, 2025).

## References

[1] E. Horvath‐Puho , M. N. van Kassel , B. P. Goncalves , et al., “Mortality, Neurodevelopmental Impairments, and Economic Outcomes After Invasive Group B Streptococcal Disease in Early Infancy in Denmark and The Netherlands: A National Matched Cohort Study,” Lancet. Child & Adolescent Health 5, no. 6 (2021): 398–407. 33894156 10.1016/S2352-4642(21)00022-5 PMC8131199

[2] L. Madrid , A. C. Seale , M. Kohli‐Lynch , et al., “Infant Group B Streptococcal Disease Incidence and Serotypes Worldwide: Systematic Review and Meta‐Analyses,” Clinical Infectious Diseases 65, no. 2 (2017): S160–S172. 29117326 10.1093/cid/cix656 PMC5850457

[3] K. Braye , J. Ferguson , D. Davis , C. Catling , A. Monk , and M. Foureur , “Effectiveness of Intrapartum Antibiotic Prophylaxis for Early‐Onset Group B Streptococcal Infection: An Integrative Review,” Women and Birth 31, no. 4 (2018): 244–253. 29129472 10.1016/j.wombi.2017.10.012

[4] S. Kenyon , K. Pike , D. R. Jones , et al., “Childhood Outcomes After Prescription of Antibiotics to Pregnant Women With Spontaneous Preterm Labour: 7‐Year Follow‐Up of the ORACLE II Trial,” Lancet 372, no. 9646 (2008): 1319–1327. 18804276 10.1016/S0140-6736(08)61203-9

[5] M. Moradi , J. Grieger , X. Tong Dr Teong , and L. Heilbronn , “Associations Between Intrapartum Antibiotic Prophylaxis and Childhood Autoimmune Diseases and Obesity: A Systematic Review and Meta‐Analysis of Observational Studies,” Obesity Research and Clinical Practice 18, no. 1 (2024): S63.

[6] G. Constantinou , S. Ayers , E. J. Mitchell , et al., “The Acceptability of Group B Streptococcal Bacteria (GBS) Testing to Women, Including Self‐Swabbing Procedures: A Qualitative Study,” Midwifery 135 (2024): 104063. 38896943 10.1016/j.midw.2024.104063

[7] G. Constantinou , S. Ayers , E. J. Mitchell , et al., “Women's Knowledge of and Attitudes Towards Group B Streptococcus (GBS) Testing in Pregnancy: A Qualitative Study,” BMC Pregnancy and Childbirth 23, no. 1 (2023): 339. 37170236 10.1186/s12884-023-05651-0 PMC10173516

[8] G. Constantinou , S. Ayers , E. J. Mitchell , et al., “The Acceptability of Implementation of Group B Streptococcus Testing: Perspectives From Women and Health Professionals in the GBS3 Trial: A Qualitative Study,” Women and Birth 37, no. 6 (2024): 101832. 39418758 10.1016/j.wombi.2024.101832

[9] P. Campbell , J. Cowie , C. Fenton , et al., “Benefits and Harms of Antenatal or Intrapartum Screening for Maternal GBS Carriage and Subsequent Use of Intrapartum Antibiotic Prophylaxis Versus Risk‐Based Protocols or no Intervention: A Rapid Review: PROSPERO,” (2025). 10.1111/apa.70568 PMC13371836 42060466

[10] A. Pollock , P. Campbell , C. Struthers , et al., “Development of the ACTIVE Framework to Describe Stakeholder Involvement in Systematic Reviews,” Journal of Health Services Research & Policy 24, no. 4 (2019): 245–255. 30997859 10.1177/1355819619841647

[11] C. Garritty , C. Hamel , M. Trivella , et al., “Updated Recommendations for the Cochrane Rapid Review Methods Guidance for Rapid Reviews of Effectiveness,” BMJ 384 (2024): e076335. 38320771 10.1136/bmj-2023-076335

[12] M. J. Page , J. E. McKenzie , P. M. Bossuyt , et al., “The PRISMA 2020 Statement: An Updated Guideline for Reporting Systematic Reviews,” BMJ 372 (2021): n71. 33782057 10.1136/bmj.n71 PMC8005924

[13] B. J. Shea , B. C. Reeves , G. Wells , et al., “AMSTAR 2: A Critical Appraisal Tool for Systematic Reviews That Include Randomised or Non‐Randomised Studies of Healthcare Interventions, or Both,” BMJ 358 (2017): j4008. 28935701 10.1136/bmj.j4008 PMC5833365

[14] J. A. Sterne , M. A. Hernan , B. C. Reeves , et al., “ROBINS‐I: A Tool for Assessing Risk of Bias in Non‐Randomised Studies of Interventions,” BMJ 355 (2016): i4919. 27733354 10.1136/bmj.i4919 PMC5062054

[15] CASP , “Critical Appraisal Skills Programme (CASP),” (2025).

[16] N. M. Alotaibi , S. Alroqi , A. Alharbi , et al., “Clinical Characteristics and Treatment Strategies for Group B Streptococcus (GBS) Infection in Pediatrics: A Systematic Review,” Medicina (Kaunas, Lithuania) 59 (2023): 597. 37512090 10.3390/medicina59071279 PMC10383037

[17] H. D. da Silva and W. L. Kretli , “Universal Gestational Screening for *Streptococcus agalactiae* Colonization and Neonatal Infection—A Systematic Review and Meta‐Analysis,” Journal of Infection and Public Health 12, no. 4 (2019): 479–481. 30940481 10.1016/j.jiph.2019.03.004

[18] C. Allen , E. Naznin , T. J. R. Panneflek , T. Lavin , and M. E. Hoque , “The Cost‐Effectiveness of Group B Streptococcus Screening Strategies in Pregnant Women for the Prevention of Newborn Early‐Onset Group B Streptococcus: A Systematic Review,” medRxiv (2024): 26.

[19] T. J. R. Panneflek , G. F. Hasperhoven , Y. Chimwaza , et al., “Intrapartum Antibiotic Prophylaxis to Prevent Group B Streptococcal Infections in Newborn Infants: A Systematic Review and Meta‐Analysis Comparing Various Strategies,” EClinicalMedicine 74 (2024): 102748. 39569026 10.1016/j.eclinm.2024.102748 PMC11577566

[20] Q. Y. Li , D. Y. Wang , H. T. Li , and J. M. Liu , “Screening‐Based and Risk‐Based Strategy for the Prevention of Early‐Onset Group B Streptococcus/Non‐Group B Streptococcus Sepsis in the Neonate: A Systematic Review and Meta‐Analysis,” Pediatric Infectious Disease Journal 39, no. 8 (2020): 740–748. 32404781 10.1097/INF.0000000000002674

[21] G. F. Hasperhoven , S. Al‐Nasiry , V. Bekker , E. Villamor , and B. Kramer , “Universal Screening Versus Risk‐Based Protocols for Antibiotic Prophylaxis During Childbirth to Prevent Early‐Onset Group B Streptococcal Disease: A Systematic Review and Meta‐Analysis,” BJOG 127, no. 6 (2020): 680–691. 31913562 10.1111/1471-0528.16085 PMC7187465

[22] J. Daniels , E. F. Dixon , A. Gill , et al., “A Rapid Intrapartum Test for Group B Streptococcus to Reduce Antibiotic Usage in Mothers With Risk Factors: The GBS2 Cluster RCT,” Health Technology Assessment (Winchester) 26, no. 12 (2022): 1–82. 10.3310/BICF1187 35195519

[23] N. El Helali , F. Habibi , E. Azria , et al., “Point‐Of‐Care Intrapartum Group B Streptococcus Molecular Screening: Effectiveness and Costs,” Obstetrics & Gynecology 133, no. 2 (2019): 276–281. 30633130 10.1097/AOG.0000000000003057

[24] D. G. E. Kolkman , M. E. B. Rijnders , M. Wouters , P. V. Dommelen , C. J. M. de Groot , and M. A. H. Fleuren , “Adherence to Three Different Strategies to Prevent Early Onset GBS Infection in Newborns,” Women & Birth: Journal of the Australian College of Midwives 33, no. 6 (2020): e527–e534. 31874785 10.1016/j.wombi.2019.12.004

[25] R. Mirsky , D. M. Carpenter , D. A. Postlethwaite , and A. C. Regenstein , “Preventing Early‐Onset Group B Streptococcal Sepsis: Is There a Role for Rescreening Near Term?,” Journal of Maternal‐Fetal and Neonatal Medicine 33, no. 22 (2020): 3791–3797. 30890002 10.1080/14767058.2019.1586874

[26] J. P. Daniels , E. Dixon , A. Gill , et al., “Rapid Intrapartum Test for Maternal Group B Streptococcal Colonisation and Its Effect on Antibiotic Use in Labouring Women With Risk Factors for Early‐Onset Neonatal Infection (GBS2): Cluster Randomised Trial With Nested Test Accuracy Study,” BMC Medicine 20, no. 1 (2022): 9. 35027057 10.1186/s12916-021-02202-2 PMC8759240

[27] M. Abdelmaaboud and A. F. Mohammed , “Universal Screening Vs. Risk‐Based Strategy for Prevention of Early‐Onset Neonatal Group‐B Streptococcal Disease,” Journal of Tropical Pediatrics 57, no. 6 (2011): 444–450. 21335324 10.1093/tropej/fmr014

[28] A. van den Hoogen , L. J. Gerards , M. A. Verboon‐Maciolek , A. Fleer , and T. G. Krediet , “Long‐Term Trends in the Epidemiology of Neonatal Sepsis and Antibiotic Susceptibility of Causative Agents,” Neonatology 97, no. 1 (2010): 22–28. 19571584 10.1159/000226604

[29] L. Youden , M. Downing , B. Halperin , H. Scott , B. Smith , and S. A. Halperin , “Group B Streptococcal Testing During Pregnancy: Survey of Postpartum Women and Audit of Current Prenatal Screening Practices,” Journal of Obstetrics and Gynaecology Canada 27, no. 11 (2005): 1006–1012. 16529666 10.1016/s1701-2163(16)30498-4

[30] S. L. Lukacs and S. J. Schrag , “Clinical Sepsis in Neonates and Young Infants, United States, 1988‐2006,” Journal of Pediatrics 160, no. 6 (2012): 960–965. 22261508 10.1016/j.jpeds.2011.12.023

[31] M. Trijbels‐Smeulders , G. A. de Jonge , P. C. Pasker‐de Jong , et al., “Epidemiology of Neonatal Group B Streptococcal Disease in The Netherlands Before and After Introduction of Guidelines for Prevention,” Archives of Disease in Childhood. Fetal and Neonatal Edition 92, no. 4 (2007): F271–F276. 17227807 10.1136/adc.2005.088799 PMC2675425

[32] S. Hakansson , M. Lilja , B. Jacobsson , and K. Kallen , “Reduced Incidence of Neonatal Early‐Onset Group B Streptococcal Infection After Promulgation of Guidelines for Risk‐Based Intrapartum Antibiotic Prophylaxis in Sweden: Analysis of a National Population‐Based Cohort,” Acta Obstetricia et Gynecologica Scandinavica 96, no. 12 (2017): 1475–1483. 28832916 10.1111/aogs.13211

[33] C. P. O'Sullivan , T. Lamagni , D. Patel , et al., “Group B Streptococcal Disease in UK and Irish Infants Younger Than 90 Days, 2014‐15: A Prospective Surveillance Study,” Lancet Infectious Diseases 19, no. 1 (2019): 83–90. 30497953 10.1016/S1473-3099(18)30555-3

[34] G. Gopal Rao , J. Townsend , D. Stevenson , et al., “Early‐Onset Group B Streptococcus (EOGBS) Infection Subsequent to Cessation of Screening‐Based Intrapartum Prophylaxis: Findings of an Observational Study in West London, UK,” BMJ Open 7, no. 11 (2017): e018795. 10.1136/bmjopen-2017-018795 PMC5701994 29158327

[35] K. Matsubara , K. Hoshina , and Y. Suzuki , “Early‐Onset and Late‐Onset Group B Streptococcal Disease in Japan: A Nationwide Surveillance Study, 2004‐2010,” International Journal of Infectious Diseases 17, no. 6 (2013): e379–e384. 23305911 10.1016/j.ijid.2012.11.027

[36] C. R. Phares , R. Lynfield , M. M. Farley , et al., “Epidemiology of Invasive Group B Streptococcal Disease in the United States, 1999‐2005,” JAMA 299, no. 17 (2008): 2056–2065. 18460666 10.1001/jama.299.17.2056

[37] M. S. Bauserman , M. M. Laughon , C. P. Hornik , et al., “Group B Streptococcus and *Escherichia coli* Infections in the Intensive Care Nursery in the Era of Intrapartum Antibiotic Prophylaxis,” Pediatric Infectious Disease Journal 32, no. 3 (2013): 208–212. 23011013 10.1097/INF.0b013e318275058a PMC3572304

[38] Y. T. V. Chan , S. Y. F. Lau , S. Y. A. Hui , et al., “Incidence of Neonatal Sepsis After Universal Antenatal Culture‐Based Screening of Group B Streptococcus and Intrapartum Antibiotics: A Multicentre Retrospective Cohort Study,” BJOG: An International Journal of Obstetrics & Gynaecology 130, no. 1 (2023): 24–31. 36002935 10.1111/1471-0528.17279

[39] B. Horvath , M. Grasselly , T. Bodecs , I. Boncz , and J. Bodis , “Screening Pregnant Women for Group B Streptococcus Infection Between 30 and 32 Weeks of Pregnancy in a Population at High Risk for Premature Birth,” International Journal of Gynaecology and Obstetrics 122, no. 1 (2013): 9–12. 23579102 10.1016/j.ijgo.2013.01.027

[40] M. A. Trijbels‐Smeulders , J. L. Kimpen , L. A. Kollée , et al., “Serotypes, Genotypes, and Antibiotic Susceptibility Profiles of Group B Streptococci Causing Neonatal Sepsis and Meningitis Before and After Introduction of Antibiotic Prophylaxis,” Pediatric Infectious Disease Journal 25, no. 10 (2006): 945–948. 17006295 10.1097/01.inf.0000237821.65559.08

[41] R. L. Davis , M. B. Hasselquist , V. Cardenas , et al., “Introduction of the New Centers for Disease Control and Prevention Group B Streptococcal Prevention Guideline at a Large West Coast Health Maintenance Organization,” American Journal of Obstetrics and Gynecology 184, no. 4 (2001): 603–610. 11262460 10.1067/mob.2001.110308

[42] D. P. Reisner , M. J. Haas , R. W. Zingheim , M. A. Williams , and D. A. Luthy , “Performance of a Group B Streptococcal Prophylaxis Protocol Combining High‐Risk Treatment and Low‐Risk Screening,” American Journal of Obstetrics and Gynecology 182, no. 6 (2000): 1335–1343. 10871447 10.1067/mob.2000.106246

[43] L. Riley , K. Appollon , S. Haider , et al., “‘Real World’ Compliance With Strategies to Prevent Early‐Onset Group B Streptococcal Disease,” Journal of Perinatology 23, no. 4 (2003): 272–277. 12774132 10.1038/sj.jp.7210895

[44] V. Bjorklund , T. Nieminen , V. M. Ulander , T. Ahola , and H. Saxen , “Replacing Risk‐Based Early‐Onset‐Disease Prevention With Intrapartum Group B Streptococcus PCR Testing,” Journal of Maternal‐Fetal & Neonatal Medicine 30, no. 3 (2017): 368–373. 27033364 10.3109/14767058.2016.1173030

[45] A. S. Coco , “Comparison of Two Prevention Strategies for Neonatal Group B Streptococcal Disease,” Journal of the American Board of Family Practice 15, no. 4 (2002): 272–276. 12150459

[46] G. J. Gilson , F. Christensen , H. Romero , K. Bekes , L. Silva , and C. R. Qualls , “Prevention of Group B Streptococcus Early‐Onset Neonatal Sepsis: Comparison of the Center for Disease Control and Prevention Screening‐Based Protocol to a Risk‐Based Protocol in Infants at Greater Than 37 Weeks' Gestation,” Journal of Perinatology 20, no. 8 Pt 1 (2000): 491–495. 11190588 10.1038/sj.jp.7200463

[47] A. Alarcon , P. Peña , S. Salas , M. Sancha , and F. Omeñaca , “Neonatal Early Onset *Escherichia coli* Sepsis: Trends in Incidence and Antimicrobial Resistance in the Era of Intrapartum Antimicrobial Prophylaxis,” Pediatric Infectious Disease Journal 23, no. 4 (2004): 295–299. 15071281 10.1097/00006454-200404000-00004

[48] A. Schuchat , A. Roome , E. R. Zell , H. Linardos , S. Zywicki , and K. L. O'Brien , “Integrated Monitoring of a New Group B Streptococcal Disease Prevention Program and Other Perinatal Infections,” Maternal and Child Health Journal 6, no. 2 (2002): 107–114. 12092979 10.1023/a:1015416324579

[49] J. Y. Hong , S. H. Kim , S. M. Kim , et al., “Evaluation of the Early Onset Neonatal Sepsis According to Two Antenatal Group B Streptococcus Screening Methods: Risk‐Based Versus Universal Screening,” Perinatology 30, no. 4 (2019): 200–207.

[50] R. S. Gibbs , R. S. McDuffie, Jr. , F. McNabb , G. E. Fryer , T. Miyoshi , and G. Merenstein , “Neonatal Group B Streptococcal Sepsis During 2 Years of a Universal Screening Program,” Obstetrics & Gynecology 84, no. 4 (1994): 496–500. 8090382

[51] M. Moradi , J. A. Grieger , X. T. Teong , and L. K. Heilbronn , “Intrapartum Antibiotic Prophylaxis and Child Health Outcomes: A Systematic Review and Meta‐Analysis of Observational Studies,” BJOG 133 (2025): 556–567. 40999925 10.1111/1471-0528.70015 PMC12884238

[52] ISRCTN49639731 , “Routine Testing for Group B Streptococcus in Pregnancy (GBS3 Trial),” (2026).

[53] D. G. E. Kolkman , L. Martin , S. Jans , et al., “Evaluation of Women's Worries in Different Strategies for the Prevention of Early Onset Group B Streptococcal Disease in Neonates,” Midwifery 86 (2020): 102623. 32278230 10.1016/j.midw.2019.102623

