# Supplementary material for: Benefits and Harms of Antenatal/Intrapartum Screening for Maternal Group B Streptococcus and Use of Intrapartum Antibiotic Prophylaxis Versus Risk‐Based Protocols or No Intervention: A Rapid Review
Source: Acta Paediatr. 2026 Apr 30;115(8):1598–610. doi: 10.1111/apa.70568 (PMC13371836; doi:10.1111/apa.70568)
Supplement: Supplementary file 1 — Data S1: Key definitions. [file APA-115-1598-s005.docx]

## Supplementary materials File 1 (S1). Key definitions

| **Term** | **Definition** |
| --- | --- |
| *Antenatal screening* | Refers to culture-based screening to identify pregnant women with GBS maternal colonization. |
| *Antibiotic prophylaxis* | Refers to the use of antibiotics in a pregnant woman with GBS colonisation or considered high risk for GBS colonisation or carriage to a newborn. |
| *Clinically suspected neonatal infection* | The following acute onset clinical or laboratory features will be used as part of the definition for clinically suspected neonatal infection, if the blood and CSF cultures are negative/unknown and intravenous antibiotics are given for ≥5 days, starting within 7 days of birth for early-onset, and 28 days for late-onset) (1):   - Increase in oxygen requirement or increase in ventilatory support or *new or an increase in frequency of episodes of apnoea - Increase in frequency of episodes of bradycardia or hypotension (needing inotrope support or other intervention) - Temperature ≥37.5C or <36.5C - Enteral feeds intolerance or abdominal distension - *Reduced urine output to <1 ml/kg/hr - *Impaired peripheral perfusion (capillary refill time >3 seconds or skin mottling or core peripheral temperature gap >2°C) - Irritability or lethargy or hypotonia (clinician-defined) - *Serum C-reactive protein levels >15 mg/L or procalcitonin ≥2 mg/mL - *White blood cells count 20×10^9^ cells/L or platelet count <100x10^9^/L - Glucose intolerance (blood glucose <2.2mmol/l or >10mmol/l) or metabolic acidosis (base excess <-10 mmol/L or lactate >2 mmol/L)   *It is acknowledged that these signs or symptoms may not be able to be identified from some routine data sources. |
| *Culture positive neonatal infection* | A positive culture of infection from blood or cerebrospinal fluid, together with signs of clinical disease in a neonate. |
| *Culture negative neonatal infection* | Symptoms or signs of sepsis, pneumonia or meningitis with a negative culture from blood or cerebrospinal fluid, in a neonate. |
| *Early all-cause neonatal infection* | Using the definition provided by (1)   - A positive culture of a pathogenic bacteria from microbiological tests (e.g., from blood; cerebrospinal fluid; pleural fluid; peritoneal fluid urine sample) at <7 days after birth - Negative/ unknown culture status with ≥3 agreed clinical signs or symptoms, for which intravenous antibiotics are given for ≥5 days, starting within 7 days of birth - Death which is <7 days after birth, if infection or sepsis was recorded on the death certificate.   We note that definitions used within the literature may vary. Therefore, we will adopt a pragmatic approach (in line with our rapid review methodology) and we will include any studies which focus on “all-cause neonatal infection” or “all-cause neonatal sepsis” (or other terms with definitions that align with the focus of our review), as defined by the study authors, as long as this occurs <28 days after birth. We use this cut-off (i.e., <28 days) as this aligns with definitions of “neonate” (2). |
| *Early-onset GBS (EOGBS) infection* | Invasive GBS disease in infants aged 0–6 days after birth. |
| *Early onset sepsis (EOS)* | Please see the definition of *Early all-cause neonatal infection* above. Neonatal sepsis with onset during the first 6 days of life. All-cause EOS refers to sepsis caused by both GBS and non-GBS infections, GBS EOS refers to sepsis caused by GBS, and non-GBS EOS refers to sepsis caused by infections other than GBS. |
| *GBS* | Group B streptococcus, also known as Streptococcus agalactiae, a facultative gram-positive organism. |
| *GBS colonisation* | Microbiological confirmation of GBS on swabs from nose, umbilicus, axilla, rectum or ear. |
| *GBS maternal colonisation* | A pregnant woman with positive confirmation of GBS (from rectovaginal or peri-anal region) (3) |
| *Intrapartum antibiotic prophylaxis (IAP)* | Antibiotics administered to pregnant women during labour. IAP exposure refers to the proportion of women receiving antibiotics during labour. |
| *Intrapartum screening* | Refers to screening at the start of labour. |
| *Invasive GBS infection* | Microbiological confirmation of GBS together with any symptoms or signs of sepsis, pneumonia or meningitis. |
| *Late-onset all-cause neonatal infection* | - A positive culture of a pathogenic bacteria taken at ≥7 days and <28 days after birth - Negative/unknown culture status with ≥3 agreed clinical signs or symptoms, for which intravenous antibiotics are given for ≥5 days, starting within 28 days of birth - Death which is <28 days after birth, if infection or sepsis was recorded on the death certificate |
| *Late-onset GBS (LOGBS) infection* | Invasive GBS disease in infants 7–89 days after birth. |
| *Long-term health conditions* | Relates to childhood, a time extending from 1-18 years of age. We note that different studies may use different thresholds to define their study outcomes. Therefore, we will adopt a pragmatic approach, documenting definitions used by study authors where these vary. |
| *Maternal GBS disease* | Laboratory isolation of GBS from a sterile site (blood or cerebrospinal fluid [CSF] only) in a pregnant or postpartum woman (up to 42 days postpartum), with a minimum of fever and physician suspicion of sepsis (4) |
| *Medium-term health conditions* | Relates to infancy, a time extending from the first month after birth to approximately 12 months of age (5). We note that different studies may use different thresholds to define their study outcomes. Therefore, we will adopt a pragmatic approach, documenting definitions used by study authors where these vary. |
| *Risk-based management strategies* | Refers to approaches where an assessment of risks is used to identify newborn babies more likely to develop invasive GBS disease, with antibiotic prophylaxis offered to reduce the risk of the newborn developing GBS disease. |
| *Short-term health conditions* | Relates to the neonatal period, which has been defined by the World Health Organization (WHO) as “beginning at birth and ending at 28 completed days of life” (6). We note that different studies may use different thresholds to define their study outcomes. Therefore, we will adopt a pragmatic approach, documenting definitions used by study authors where these vary. |
| *Systematic review* | EPPI-Centre definition of a systematic review as a review that uses explicit and transparent methods, follows a standard set of stages and can be considered accountable, replicable and updateable (7) |
| *Timing of screening* | Screening of pregnant women to detect maternal GBS can occur during pregnancy (antenatal) or during labour/delivery (intrapartum). |

References

1. Daniels J, Walker K, Bradshaw L, Dorling J, Ojha S, Gray J, et al. Routine testing for group B streptococcus in pregnancy: protocol for a UK cluster randomised trial (GBS3). *BMJ Open* 2025; 15 6:e087887.

2. NHS Data Model and Dictionary. Neonate. 2024

3. Paul P, Gonçalves BP, Le Doare K, Lawn JE. 20 million pregnant women with group B streptococcus carriage: consequences, challenges, and opportunities for prevention. *Curr Opin Pediatr* 2023; 35 2:223-30.

4. Hall J, Adams NH, Bartlett L, Seale AC, Lamagni T, Bianchi-Jassir F, et al. Maternal Disease With Group B Streptococcus and Serotype Distribution Worldwide: Systematic Review and Meta-analyses. *Clin Infect Dis* 2017; 65 suppl_2:S112-S24.

5. Madrid L, Seale AC, Kohli-Lynch M, Edmond KM, Lawn JE, Heath PT, et al. Infant Group B Streptococcal Disease Incidence and Serotypes Worldwide: Systematic Review and Meta-analyses. *Clin Infect Dis* 2017; 65 suppl_2:S160-S72.

6. Lehtonen L, Gimeno A, Parra-Llorca A, Vento M. Early neonatal death: A challenge worldwide. *Semin Fetal Neonatal Med* 2017; 22 3:153-60.

7. EPPI-Centre. What is a systematic review? UK: Social Science Research Unit, UCL Institute of Education.; 2021
