# Supplementary material for: Benefits and Harms of Antenatal/Intrapartum Screening for Maternal Group B Streptococcus and Use of Intrapartum Antibiotic Prophylaxis Versus Risk‐Based Protocols or No Intervention: A Rapid Review
Source: Acta Paediatr. 2026 Apr 30;115(8):1598–610. doi: 10.1111/apa.70568 (PMC13371836; doi:10.1111/apa.70568)
Supplement: Supplementary file 2 — Data S2: Reference group involvement. [file APA-115-1598-s003.docx]

Supplementary materials File 2. Reference Group involvement

**SHORT TOOL FOR *PLANNING & RECORDING* PPI IN A REVIEW (NIHR Evidence Synthesis Group PPI in a Review)**

**This NIHR PPI short tool has been developed by the NIHR Evidence Synthesis Group PPI working group. It incorporates ACTIVE framework and GRIPP2 guidance and other domains identified as important by the NIHR ESG PPI working group.**

**Review Title: Antenatal Screening for Group B Streptococcus (GBS) rapid reviews**

**Who completed this form?** *(Consider involving different team members, including public contributors)*

| **Name** | **Role** | **Section(s) completed** | **Date** |
| --- | --- | --- | --- |
| **Bridget Davis** | **NESSIE reviewer** | **All sections** | **01/07/2025** |
| **Rosie Hill** | **NESSIE PPI Co-applicant** | **Reviewed all sections** | **09/07/2025** |
| **Narendra Aladangady** | **Neonatologist** | **Section 4** | **19/07/2025** |
| **Ryan Kean** | **Lecturer in Clinical Microbiology** | **Section 4** | **27/06/2025** |

|  | | **Background** | | **At protocol stage** | **On review completion** | |
| --- | --- | --- | --- | --- | --- | --- |
| 1 | | **What were the REVIEW QUESTIONS?**  (The NESSIE GBS Reference group works across the three linked rapid reviews) | | **Review A:** **1: UK DATA ON INCIDENCE AND NATURAL HISTORY**  Q1.1 What are the reported incidences of early and late-onset GBS infection in the UK and how does incidence vary according to key characteristics of the population?  Q1.2 What are the reported rates of death, and short-, medium-, or long-term health conditions attributed to EOGBS or LOGBS infection in the UK?  **2: UK and other high-income country data on the health consequences of EOGBS or LOGBS infection**  Q2 What short-, medium- or long-term health conditions have been specifically shown to occur at higher rates in neonates/infants/children who had EOGBS or LOGBS infection compared with those who did not?  **Review B: 1: UK DATA ON INCIDENCE AND NATURAL HISTORY**  Q1.1 What are the reported incidences of early-onset and/or late-onset all-cause neonatal infection in the UK and how does incidence vary according to key characteristics of the population?  Q1.2 What are the reported rates of death, and short-, medium-, or long-term health conditions attributed to early-onset and/or late-onset all-cause neonatal infection in the UK?  **2: UK and other high-income country data on the health consequences of all-cause neonatal infection**  Q2 What short-, medium- or long-term health conditions have been specifically shown to occur at higher rates in neonates/infants/children who had early-onset or late-onset all-cause neonatal infection compared with those who did not?  **Review C:** **BENEFITS AND HARMS OF ANTENATAL OR INTRAPARTUM SCREENING FOR MATERNAL GBS CARRIAGE AND SUBSEQUENT USE OF INTRAPARTUM ANTIBIOTIC PROPHYLAXIS VERSUS RISK-BASED PROTOCOLS OR NO INTERVENTION: A RAPID REVIEW.**  **RQ1.** Do maternal GBS screening-based protocols (antenatal or intrapartum) and subsequent use of intrapartum antibiotic prophylaxis reduce the risk of EOGBS, LOGBS and other cause neonatal infections compared with risk-based protocols or no intervention?  **RQ2.** What benefits and harms of undergoing (antenatal or intrapartum) screening for GBS and subsequent use of intrapartum antibiotic prophylaxis have been reported by high-quality research (i.e., studies with appropriate comparator groups) conducted in high-income countries? | There was no change to review questions | |
| 2 | | **WHAT were the key factors in relation to timeline, resources, review method etc** | | We planned three linked, rapid reviews:   - We had a deadline of 6 months to complete and submit all 3 reviews - We started contacting potential reference group members at the draft protocol stage to engage participation at the earliest possible point in the review process - Securing expertise at this stage was important because the review team were unfamiliar with the topic and we anticipated that experts would help our understanding and ensure accuracy in the protocol content | We took a pragmatic, time-efficient approach. We sourced recommended contacts who were known to have relevant background and expertise. At completion of the reviews, all Reference group members had participated to some degree and had stayed with the reviews to completion | |
| 3 | | **WHY were people involved? What was the purpose / objective of involving people?** | | We wanted to establish a small ‘Reference Group’ comprising people that could bring relevant experience and expertise in the topic area:   - People with clinical / scientific experience (e.g. midwives, gynaecologists, neonatologists, microbiologists) - People with policy experience - People representing those with lived experience (e.g. mother & baby representatives) - A NESSIE PPI co-applicant   These people would be invited to advise throughout the progression of the review. The topic area was unfamiliar and complex in nature to the review team and we hoped that the Reference group would assist us at the most basic level (e.g. terminology, knowledge around specific areas of the topic), and advise us in many aspects of the review. | The Reference group members contributed throughout the review until completion | |
|  | | **Who was involved and how did they get involved?** | | **Plans** | **What happened** | |
| 4 | | WHY did Reference group members get involved? What were their motivations? What did they expect they were going to contribute? | | People were invited specifically for their expertise and experience with the expectation that they would advise us throughout the reviews on terminology, scientific content, study inclusion decisions, and reporting and dissemination (which would provide authorship opportunities). | To offer insight and experience, especially to complex and specific aspects of the review.  Offer views on inclusion of studies for the review.  Hope to be involved in publication opportunities.  Provide advice pertaining to specialist areas (microbiology, clinical policy, health policy). | |
| 5 | | WHO was involved? State if these were patients/public/carers, health professionals or other stakeholders. | | Health professionals/clinicians; people with policy expertise and knowledge; people with lived experience of GBS screening. | We secured the involvement of:   - A NESSIE PPI co-applicant with knowledge of UK public health policy and who has expertise in most areas of the review progress - A midwife with clinical and policy knowledge - Two microbiologists / lecturers in clinical microbiology with expertise in bacterial and fungal pathogens affecting womens’ health - A Neonatologist - A Paediatrician who could advise on the long-term (usually age 2-5 years) outcomes of neonates - A mother with lived experience of GBS screening during pregnancy | |
| 6 | | How many people were involved? | | We planned to contact a small number of people from across the UK | Seven Reference group members were recruited | |
| 7 | | What were the characteristics/qualifications of the people involved?   - Patients / public / carers / patient organisation / health professional - Lived experience of health condition / topic - Equality, diversity and inclusion (EDI) characteristics | | We contacted a small number of key people from across the UK with professional experience and expertise in microbiology, midwifery, neonatology, and paediatrics. We also sought to contact people with lived experience of GBS screening during pregnancy.  We did not collect EDI characteristics of Reference Group members | See point 5 | |
| 8 | | HOW were people recruited?  (What was the process of inviting people to contribute to the review?) | | The NESSIE review team knew a small number of key people who had experience and expert knowledge of the topic area. We anticipated that these people would have relevant expertise and/or capacity to be involved, and that they may know additional, relevant contacts we could also invite as members of the Reference group.  We then drafted an **invitational email** to these additional, suggested contacts describing why we were contacting them personally, including a brief description of the NESSIE ESG role and purpose (and online website link), and an outline of the review, explaining the nature of the rapid review and strict timeline. | | Interested people who responded to the invitational attended either an informal, online meeting with the Review Lead and a NESSIE reviewer (ATB/BD), or a telephone call (with BD); we also shared the draft review protocols prior to the meeting.  The purpose of the meeting was:   - To provide a more detailed description of the review and review process - To discuss the potential role of reference group members to gauge whether they felt they had relevant experience/expertise and capacity to be involved and - To answer any questions they may have - We also asked specific protocol-related questions to help writing and planning our work (e.g scientific queries, definitions of sepsis)   We held two online meetings and a telephone call with potential contributors at this stage |
| 9 | | Why were people recruited in this way? | | This direct, focussed approach was adopted to:   - Recruit and involve reference group members at the earliest possible stage of the reviews to meet the tight deadlines - We decided to avoid wider circulation of information as would be our usual strategy, (e.g. contacting charitable, patient, and other relevant organisations; reaching out via social media) due the emotive and politically sensitive nature of the topic area. | **At protocol draft stage:** We met (online)  **Meeting 1:** Present were a Professor of midwifery, our NESSIE co-applicant (RH), and a Clinical Lecturer in microbiology. Some discussion points were:   - We were advised that when referring to pregnancy we should use sexed language i.e ‘pregnant women’ rather than ‘pregnant person’ (referencing the Midwifery Network Position Paper). - We discussed our understanding of GBS microbiology and were able to confirm correct wording and scientific terminology within the protocol. - We discussed the appropriateness of involving people from 3^rd^ sector organisations given that topic area is emotive and politically sensitive, and agreed to take this forward for further discussion with our wider NESSIE team.   **Meeting 2:** A neonatologist and Clinical Senior Lecturer in GU medicine. We continued to establish our understanding of the topic area, especially around the current definitions of sepsis.  **Meeting 3:** A telephone call with a mother who has lived experience of GBS screening within pregnancy.  Meeting with these people helped our understanding of the topic matter at this stage, especially in writing the protocol. They all read and some commented on the protocol drafts.  **Later in the review process (full text screening stage):**  We contacted a paediatrician and a practising midwife. We followed the same email process as before but did not meet with them (although a meeting was offered).  All the people we contacted and spoke to (with the exception of the practising midwife from whom we had no response) agreed to be members of the Reference group and to read and comment on the draft protocols. We established the main method of  communication would be through email unless it was felt at any time that a meeting would be preferable. | |
|  | | **Approach / methods of involvement** | | **Plans** | **What happened** | |
| 10 | | WHAT did you do? Give a brief summary of what you did, clearly stating when in the review process this was. | | We contacted people as early as possible to clarify our understanding of the complex review topic matter while writing the protocol.  We did not plan a series of PPI meetings over the course of the reviews as we would normally have done, instead we planned to engage the expert opinion of group members as and when we needed, primarily using email.  As we progressed through the review process we sought to recruit a paediatrician and a practising midwife. | **At protocol draft stage:**  Reference group members read and commented on the protocol drafts. Communication was mainly through emails. Virtual or telephone meetings were conducted where required.  **Throughout the screening and data extraction phase:**  We contacted appropriate Reference Group members and asked their advice on:   - Refinement of search terms (condition and population) - Advice on inclusion/exclusion decisions - Advice on specific scientific or medical aspects of individual studies   **Drafting and writing of main paper:**   - We asked members to read and comment on the three manuscripts   **Dissemination**   - We were advised on appropriate journals for potential publication | |
| 11 | | WHEN did you do it? | |  | Stages at which people were involved is outlined in response to Point 10. | |
|  | | **Review methods – consideration of PPI in included studies** | | **Plans** | **What happened** | |
| 12 | | Was public involvement within the studies included in the systematic review considered? *(e.g. during study selection, data extraction, synthesis, reporting)* | | Public involvement within included studies was not considered for this rapid review |  | |
|  | | **Impact of involvement** | | **Anticipated impact** | **Actual impact** | |
| 13 | | WHAT was the impact on the review? Summarise what the impact of involvement was on the review. | | Increase the review team’s knowledge and understanding of the topic area | The Reference Group were instrumental in helping our understanding of this complex topic area. | |
|  |  |  |  | Writing of the review protocols. | Reference group members read the protocols. Some commented on the documents where relevant | |
|  |  |  |  | Opinion and advice on various aspects of the review including:   - Refinement of search terms (specifically on condition and population) - Advice relating to inclusion/exclusion of specific studies - Provide information relating to specific study to help our understanding of the topic | The reference group’s input helped refinement of search terms prior to screening, decision making around inclusion/exclusion of studies, and confirmed understanding of complex data within individual studies. | |
|  |  |  |  | Identification of the most appropriate journal for publication of the reviews | Members advised us about which journals were the best fit for publication. This helped our decision on which journal to choose for potential publication | |
|  |  |  |  | Dissemination: Reviewing and commenting on draft manuscripts and securing authorship (we circulated ICMJE criteria authorship in advance) | Members read and commented on manuscript drafts. Those who contributed were offered authorship. | |
| 14 | | WHAT was the impact on the Reference group members? | |  | Reference Group members reported that they:     - Learnt rapid review process - Gained knowledge on incidence of GBS disease in UK, disease burden and short and long-term outcomes of GBS disease. - Various GBS screening methods practiced and their impact. - Also, incidence and outcomes of all cause neonatal infection - Future research need: women's experience of GBS screening, large national and multinational studies of GBS disease with clear definition of EOGBS and LOGBS infection   They reported that “overall role/responsibility was time consuming but very worthwhile”. | |
| 15 | | HOW DID IT GO? Reflection on the involvement.  (Including reflections from researchers AND PPI contributors) | |  | **Researcher reflections:** Being a researcher without any prior knowledge relating to the review topic, we found the input of the Reference Group extremely valuable. During the initial meetings the Reference Group members answered my questions, helping ensure that I had an understanding of key issues and terminology. We achieved our goal of contacting and recruiting most Reference Group members in time to review and comment on the protocol which was extremely helpful in expanding our initial understanding of the topic while writing the protocol.  Being explicit about our tight timeline was probably helpful in the success we had in Reference group members communicating promptly when asked for advice and comments on manuscripts.  Being able to contact Reference Group members during the review conduct period, particularly asking questions about whether specific papers met inclusion criteria was important, and increased our confidence in the decisions that we were making. Having feedback and comments on all the draft manuscripts was essential to ensure that we had written the reviews in a way that was relevant and understandable to the key audience. Suggestions around journals that were read by relevant professionals impacted our decision on the journals to target for our publications.  The mother with lived experience felt she lacked the scientific knowledge to comment on the draft documents; however, should we require a plain English summary, her input would be valuable. | |
| 16 | | Evidence gaps. Were topics/issues raised by public contributors which were not identified in the systematic review? | |  | No, not that we are aware of aware of | |
|  | | **Other** | | **At the planning stage?** | **At review completion?** | |
| 17 | | Key lessons learnt | |  | Our approach in having a Reference Group was successful for this rapid review and is an approach that we would use again | |
| 18 | | Did you report the PPI? (e.g. in the protocol, main report, separate paper, supplementary files) | | Yes, we outlined our planned PPI strategy in the review protocols | Yes, we completed the NIHR ESG PPI tool (short form). Researchers, the PPI co-applicant, and Reference members contributed to this document as supplementary material to the main paper | |
| 19 | | Any other comments about PPI in this review? | |  |  | |

Abbreviations: EDI: Equality, Diversity and Inclusion; EOGBS: early-onset GBS; ESG: Evidence Synthesis Group; GBS: Group B Streptococcus; GU: Genitourinary; ICMJE: International Committee of Medical Journal Editors; LOGBS: late-onset GBS; NESSIE: NIHR Evidence Synthesis Scotland Initiative; NIHR: National Institute for Health and Care Research; PPI: Patient and public involvement
