# Supplementary material for: Benefits and Harms of Antenatal/Intrapartum Screening for Maternal Group B Streptococcus and Use of Intrapartum Antibiotic Prophylaxis Versus Risk‐Based Protocols or No Intervention: A Rapid Review
Source: Acta Paediatr. 2026 Apr 30;115(8):1598–610. doi: 10.1111/apa.70568 (PMC13371836; doi:10.1111/apa.70568)
Supplement: Supplementary file 3 — Data S3: Search strategy and report. [file APA-115-1598-s001.docx]

## Supplementary materials File 3 (S3). Search strategy and report

Benefits and harms of antenatal or intrapartum screening for maternal GBS carriage and subsequent use of intrapartum antibiotic prophylaxis versus risk-based protocols or no intervention: a rapid review – systematic reviews

**Phase One search time frame: 1 January 2016 to 26 February 2025**

| **Source** | **Version/Platform/URL** | **Date of Search** | **Records retrieved** |
| --- | --- | --- | --- |
| 1. Medline (Ovid MEDLINE® Epub Ahead of Print, In-Process & Other Non-Indexed Citations, Ovid MEDLINE® Daily and Ovid MEDLINE®) 1946 to present | OVID | 26.2.25 | 179 |
| 2. EMBASE | OVID | 26.2.25 | 243 |
| TOTAL before de-duplication |  |  | 422 |

| **Source** | **Search strategy** | **Hits retrieved** |
| --- | --- | --- |
| 1. Medline (Ovid MEDLINE® Epub Ahead of Print, In-Process & Other Non-Indexed Citations, Ovid MEDLINE® Daily and Ovid MEDLINE®) 1946 to present | 1. exp Streptococcus agalactiae/  2. "group b streptococc*".ti,ab.  3. "streptococcus agalactiae".ti,ab.  4. "Streptococcus Group B".ti,ab.  5. EOGBS.ti,ab.  6. GBS.ti,ab.  7. iGBS.ti,ab.  8. LOGBS.ti,ab.  9. Pregnancy Complications, Infectious/di  10. or/1-9  11. exp Mass Screening/  12. Streptococcal Infections/di  13. Infectious Disease Transmission, Vertical/pc  14. test*.ti,ab.  15. screen*.ti,ab.  16. or/11-15  17. (systematic review or meta-analysis).pt.  18. meta-analysis/ or systematic review/ or systematic reviews as topic/ or meta-analysis as topic/ or "meta analysis (topic)"/ or "systematic review (topic)"/ or exp technology assessment, biomedical/ or network meta-analysis/  19. ((systematic* adj3 (review* or overview*)) or (methodologic* adj3 (review* or overview*))).ti,ab,kf.  20. ((quantitative adj3 (review* or overview* or synthes*)) or (research adj3 (integrati* or overview*))).ti,ab,kf.  21. ((integrative adj3 (review* or overview*)) or (collaborative adj3 (review* or overview*)) or (pool* adj3 analy*)).ti,ab,kf.  22. (data synthes* or data extraction* or data abstraction*).ti,ab,kf.  23. (handsearch* or hand search*).ti,ab,kf.  24. (mantel haenszel or peto or der simonian or dersimonian or fixed effect* or latin square*).ti,ab,kf.  25. (met analy* or metanaly* or technology assessment* or HTA or HTAs or technology overview* or technology appraisal*).ti,ab,kf.  26. (meta regression* or metaregression*).ti,ab,kf.  27. (meta-analy* or metaanaly* or systematic review* or biomedical technology assessment* or bio-medical technology assessment*).mp,hw.  28. (medline or cochrane or pubmed or medlars or embase or cinahl).ti,ab,hw.  29. (cochrane or (health adj2 technology assessment) or evidence report).jw.  30. (comparative adj3 (efficacy or effectiveness)).ti,ab,kf.  31. (outcomes research or relative effectiveness).ti,ab,kf.  32. ((indirect or indirect treatment or mixed-treatment or bayesian) adj3 comparison*).ti,ab,kf.  33. (multi* adj3 treatment adj3 comparison*).ti,ab,kf.  34. (mixed adj3 treatment adj3 (meta-analy* or metaanaly*)).ti,ab,kf.  35. (multi* adj2 paramet* adj2 evidence adj2 synthesis).ti,ab,kf.  36. (multiparamet* adj2 evidence adj2 synthesis).ti,ab,kf.  37. (multi-paramet* adj2 evidence adj2 synthesis).ti,ab,kf.  38. or/17-37  39. 10 and 16 and 38  40. limit 39 to english language  41. (2016* or 2017* or 2018* or 2019* or 2020* or 2021* or 2022* or 2023* or 2024* or 2025*).ed.  42. 40 and 41 | Feb 2025: 179 |
| 2. EMBASE via Ovid | 1. exp Streptococcus agalactiae/  2. "group b streptococc*".ti,ab.  3. "streptococcus agalactiae".ti,ab.  4. "Streptococcus Group B".ti,ab.  5. EOGBS.ti,ab.  6. GBS.ti,ab.  7. iGBS.ti,ab.  8. LOGBS.ti,ab.  9. exp infectious pregnancy complication/di [Diagnosis]  10. or/1-9  11. exp mass screening/  12. exp Streptococcus infection/di [Diagnosis]  13. exp vertical transmission/pc [Prevention]  14. test*.ti,ab.  15. screen*.ti,ab.  16. or/11-15  17. (systematic review or meta-analysis).pt.  18. meta-analysis/ or systematic review/ or systematic reviews as topic/ or meta-analysis as topic/ or "meta analysis (topic)"/ or "systematic review (topic)"/ or exp technology assessment, biomedical/ or network meta-analysis/  19. ((systematic* adj3 (review* or overview*)) or (methodologic* adj3 (review* or overview*))).ti,ab,kf.  20. ((quantitative adj3 (review* or overview* or synthes*)) or (research adj3 (integrati* or overview*))).ti,ab,kf.  21. ((integrative adj3 (review* or overview*)) or (collaborative adj3 (review* or overview*)) or (pool* adj3 analy*)).ti,ab,kf.  22. (data synthes* or data extraction* or data abstraction*).ti,ab,kf.  23. (handsearch* or hand search*).ti,ab,kf.  24. (mantel haenszel or peto or der simonian or dersimonian or fixed effect* or latin square*).ti,ab,kf.  25. (met analy* or metanaly* or technology assessment* or HTA or HTAs or technology overview* or technology appraisal*).ti,ab,kf.  26. (meta regression* or metaregression*).ti,ab,kf.  27. (meta-analy* or metaanaly* or systematic review* or biomedical technology assessment* or bio-medical technology assessment*).mp,hw.  28. (medline or cochrane or pubmed or medlars or embase or cinahl).ti,ab,hw.  29. (cochrane or (health adj2 technology assessment) or evidence report).jw.  30. (comparative adj3 (efficacy or effectiveness)).ti,ab,kf.  31. (outcomes research or relative effectiveness).ti,ab,kf.  32. ((indirect or indirect treatment or mixed-treatment or bayesian) adj3 comparison*).ti,ab,kf.  33. (multi* adj3 treatment adj3 comparison*).ti,ab,kf.  34. (mixed adj3 treatment adj3 (meta-analy* or metaanaly*)).ti,ab,kf.  35. (multi* adj2 paramet* adj2 evidence adj2 synthesis).ti,ab,kf.  36. (multiparamet* adj2 evidence adj2 synthesis).ti,ab,kf.  37. (multi-paramet* adj2 evidence adj2 synthesis).ti,ab,kf.  38. or/17-37  39. 10 and 16 and 38  40. limit 39 to english language  41. (2016* or 2017* or 2016* or 2017* or 2018* or 2019* or 2020* or 2021* or 2022* or 2023* or 2024* or 2025*).dc.  42. 40 and 41 | Feb 2025: 243 |
| TOTAL before de-duplication | | 422 |
| TOTAL after de-duplication | | 323 |

Benefits and harms of antenatal or intrapartum screening for maternal GBS carriage and subsequent use of intrapartum antibiotic prophylaxis versus risk-based protocols or no intervention: a rapid review - primary studies

**Phase Two search time frame: 1 January 2019 to 11 March 2025**

| **Source** | **Version/Platform/URL** | **Date of Search** | **Records retrieved** |
| --- | --- | --- | --- |
| 1. Medline (Ovid MEDLINE® Epub Ahead of Print, In-Process & Other Non-Indexed Citations, Ovid MEDLINE® Daily and Ovid MEDLINE®) 1946 to present | OVID | 11 March 2025 | 2361 |
| 2. EMBASE | OVID | 11 March 2025 | 3547 |
| TOTAL before de-duplication |  |  | 5908 |

| **Source** | **Search strategy** | **Hits retrieved** |
| --- | --- | --- |
| 1. Medline (Ovid MEDLINE® Epub Ahead of Print, In-Process & Other Non-Indexed Citations, Ovid MEDLINE® Daily and Ovid MEDLINE®) 1946 to present | 1. exp Streptococcus agalactiae/  2. "group b streptococc*".ti,ab.  3. "streptococcus agalactiae".ti,ab.  4. "Streptococcus Group B".ti,ab.  5. EOGBS.ti,ab.  6. GBS.ti,ab.  7. iGBS.ti,ab.  8. LOGBS.ti,ab.  9. Pregnancy Complications, Infectious/di  10. or/1-9  11. exp Mass Screening/  12. Streptococcal Infections/di  13. Infectious Disease Transmission, Vertical/pc  14. test*.ti,ab.  15. screen*.ti,ab.  16. or/11-15  17. 10 and 16  18. limit 17 to english language  19. (2019* or 2020* or 2021* or 2022* or 2023* or 2024* or 2025*).ed.  20. 18 and 19 | March 2025: 2361 |
| Embase Classic+Embase <1947 to 2025 Week 10> | 1. exp Streptococcus agalactiae/  2. "group b streptococc*".ti,ab.  3. "streptococcus agalactiae".ti,ab.  4. "Streptococcus Group B".ti,ab.  5. EOGBS.ti,ab.  6. GBS.ti,ab.  7. iGBS.ti,ab.  8. LOGBS.ti,ab.  9. exp infectious pregnancy complication/di [Diagnosis]  10. or/1-9  11. exp mass screening/  12. exp Streptococcus infection/di [Diagnosis]  13. exp vertical transmission/pc [Prevention]  14. test*.ti,ab.  15. screen*.ti,ab.  16. or/11-15  17. 10 and 16  18. limit 17 to english language  19. (2019* or 2020* or 2021* or 2022* or 2023* or 2024* or 2025*).dc.  20. 18 and 19 | March 2025: 3547 |
| TOTAL before de-duplication | | 5908 |
| TOTAL after de-duplication | | 4674 |
