# Supplementary material for: Benefits and Harms of Antenatal/Intrapartum Screening for Maternal Group B Streptococcus and Use of Intrapartum Antibiotic Prophylaxis Versus Risk‐Based Protocols or No Intervention: A Rapid Review
Source: Acta Paediatr. 2026 Apr 30;115(8):1598–610. doi: 10.1111/apa.70568 (PMC13371836; doi:10.1111/apa.70568)
Supplement: Supplementary file 4 — Data S4: Quality assessments. [file APA-115-1598-s012.docx]

## Supplementary materials File (S4). Quality Assessments

### File 4.1. Judgements of systematic reviews using the AMSTAR-2 critical outcomes

| **Study ID** | **Title** | **Item 2: Protocol registered before commencement of the review** | **Item 4: Adequacy of the literature search** | **Item 7: Justification for excluding individual studies** | **Item 9: ROB from individual studies being included in the review** | **Item 11: Appropriateness of meta-analytical methods** | **Item 13: Consideration of ROB when interpreting the results of the review** | **Item 15: Assessment of presence and likely impact of publication bias** | **Judgement** |
| --- | --- | --- | --- | --- | --- | --- | --- | --- | --- |
| **Alotaibi 2023 (1)** | Clinical Characteristics and Treatment Strategies for Group B Streptococcus (GBS) Infection in Pediatrics: A Systematic Review | Unsure | No | No | Partial yes | No meta-analysis conducted | No | No meta-analysis conducted | CRITICALLY LOW |
| **Braye 2018 (2)** | Effectiveness of intrapartum antibiotic prophylaxis for early-onset group B Streptococcal infection: An integrative review | Unsure | Partial yes | No | Partial yes | No meta-analysis conducted | Yes | No meta-analysis conducted | CRITICALLY LOW / LOW |
| **da Silva 2019 (3)** | Universal gestational screening for Streptococcus agalactiae colonization and neonatal infection - A systematic review and meta-analysis | Unsure | No | No | No | No | No | No | CRITICALLY LOW |
| **Hasperhoven 2020 (4)** | Universal screening versus risk-based protocols for antibiotic prophylaxis during childbirth to prevent early-onset group B streptococcal disease: a systematic review and meta-analysis | Yes | Partial yes | Yes | Yes | Yes | Yes | Yes | MODERATE/HIGH |
| **Li 2020 (5)** | Screening-based and Risk-based Strategy for the Prevention of Early-onset Group B Streptococcus/Non-group B Streptococcus Sepsis in the Neonate: A Systematic Review and Meta-analysis | Unsure | Partial yes | Yes | Yes | Yes | Yes | Yes | MODERATE/HIGH |
| **Panneflek 2024 (6)** | Intrapartum antibiotic prophylaxis to prevent Group B streptococcal infections in newborn infants: a systematic review and meta-analysis comparing various strategies | Yes | Partial yes | Yes* | Yes | Yes | Yes | Yes | MODERATE/HIGH |

*Review authors provided list of excluded studies with reasons on request

File 4.2. Judgements of RCTs using CASP tool

| **Study ID** | **1. Did the study address a clearly formulated research question?** | **2. Was the assignment of participants to interventions randomised?** | **3. Were all participants who entered the study accounted for at its conclusion?** | **4. (a) Were the participants ‘blind’ to intervention they were given?** | **4. (b) Were the investigators ‘blind’ to the intervention they were giving to participants?** | **4. (c) Were the people assessing/analysing outcome/s ‘blinded’?** | **5. Were the study groups similar at the start of the randomised controlled trial?** | **6. Apart from the experimental intervention, did each study group receive the same level of care (that is, were they treated equally)?** | **7. Were the effects of intervention reported comprehensively?** | **8. Was the precision of the estimate of the intervention or treatment effect reported?** | **9. Do the benefits of the experimental intervention outweigh the harms and costs?** | **10. Can the results be applied to your local population/in your context?** | **11. Would the experimental intervention provide greater value to the people in your care than any of the existing interventions?** | **Additional notes** |
| --- | --- | --- | --- | --- | --- | --- | --- | --- | --- | --- | --- | --- | --- | --- |
| Daniels 2022 (7, 8) | Yes | Yes | Yes | No | No | No | Yes | Yes | Yes | Yes | Yes | Can't tell | Can't tell | Lack of blinding may influence participant and investigator behaviour. Participants restricted to women with risk factors, information on ethnicity not collected, implications on applicability |
| Kolkman 2022 (9, 10) | Yes | Yes | Yes | No | No | No | No | Yes | Yes | Yes | Can't tell | Can't tell | Can't tell | Lack of blinding may influence participant and investigator behaviour and groups not comparable. Low adherence and does not report on outcomes of interest |

Abbreviations: CASP: Critical Appraisal programme; RCT: randomised controlled trial;

### File 4.3. Risk of Bias assessment for non-randomised primary studies using ROBINS-I

|  | **Bias due to confounding** | **Bias in selection of participants into the study** | **Bias in classification of interventions** | **Bias due to deviations from intended interventions** | **Bias due to missing data** | **Bias in measurement of outcomes** | **Bias in selection of the reported result** | **Overall bias** |
| --- | --- | --- | --- | --- | --- | --- | --- | --- |
| **Abdelmaaboud 2011**(11) | Serious | Low | Low | NI | Moderate | Moderate | Critical | Critical |
| **Alarcon 2004**(12) | Moderate | Moderate | Low | NI | Moderate | Moderate | Low | Moderate |
| **Al Luhidan 2019**(13) | Serious | Low | Low | NI | Moderate | Moderate | Low | Serious |
| **Angstetra 2007 (P)**(14) | Low | Low | Serious | NI | Low | Moderate | Low | Serious |
| **Angstetra 2007 (H)**(14) | Moderate | Low | Moderate | NI | Moderate | Moderate | Moderate | Moderate |
| **Bauserman 2013**(15) | Moderate | Serious | Serious | NI | Moderate | Moderate | Low | Serious |
| **Bekker 2014**(16) | Serious | Low | Low | NI | Low | Low | Serious | Moderate |
| **Bizzarro 2005**(17) | Serious | Low | Serious | NI | Moderate | Moderate | Moderate | Serious |
| **Bjorklund 2017**(18) | Low | Low | Low | Low | Moderate | Moderate | Moderate | Moderate |
| **Bjornsdottir 2019**(19) | Serious | Serious | Serious | NI | Moderate | Moderate | Moderate | Serious |
| **Brozanski 2000**(20) | Serious | Low | Serious | Serious | Moderate | Moderate | Low | Serious |
| **Chan 2023**(21) | Serious | Low | Low | NI | Moderate | Moderate | Low | Serious |
| **Chen 2001 (22)** | Moderate | Low | Low | NI | Moderate | Moderate | Low | Moderate |
| **Chen 2005**(23) | Serious | Moderate | Moderate | Serious | Moderate | Moderate | Moderate | Serious |
| **Cho 2019**(24) | Moderate | Low | Moderate | Low | Moderate | Moderate | Moderate | Moderate |
| **Clemens 2002**(25) | Serious | Low | Serious | Low | Moderate | Serious | Low | Serious |
| **Coco 2002**(26) | Low | Low | Moderate | Low | Moderate | Moderate | Low | Moderate |
| **Darlow 2016**(27) | Serious | Low | Moderate | Serious | Moderate | Serious | Moderate | Serious |
| **Davis 2001**(28) | Moderate | Low | Low | Moderate | Moderate | Moderate | Low | Moderate |
| **Eberly 2009**(29) | Serious | Low | Low | NI | Moderate | Moderate | Low | Serious |
| **Ecker 2013**(30) | Serious | Low | Serious | NI | Moderate | Moderate | Low | Serious |
| **Edwards 2003 (P)**(31) | Serious | Low | Serious | NI | Moderate | Moderate | Low | Serious |
| **Edwards 2003 (H)**(31) | Serious | Moderate | Serious | Serious | Moderate | Low | Moderate | Serious |
| **Eisenberg 2005 (P)**(32) | Moderate | Low | Moderate | Moderate | Moderate | Low | Low | Moderate |
| **Eisenberg 2005 (H)**(32) | Serious | Moderate | Serious | Moderate | NI | Low | Serious | Moderate |
| **El Helali 2019**(33) | Moderate | Low | Low | Low | Moderate | Moderate | Low | Moderate |
| **Factor 1998**(34) | Low | Low | Low | Moderate | Moderate | Low | Low | Moderate |
| **Garland 1991**(35) | Low | Moderate | Low | NI | Moderate | Serious | Low | Serious |
| **Gibbs 1994**(36) | Serious | Low | Low | Low | Moderate | Serious | Low | Serious |
| **Gilson 2000 (P)**(37) | Low | Moderate | Low | Moderate | Moderate | Moderate | Low | Moderate |
| **Gilson 2000 (H)**(37) | Moderate | Serious | Moderate | Low | Moderate | Low | Moderate | Moderate |
| **Gopal Rao 2017 (P)**(38) | Moderate | Low | Low | Low | Moderate | Low | Low | Moderate |
| **Gopal Rao 2017 (H)**(38) | Moderate | Low | Moderate | Low | Low | Low | Moderate | Moderate |
| **Gosling 2002**(39) | Serious | Serious | Moderate | NI | Low | Moderate | Low | Serious |
| **Hafner 1998**(40) | Moderate | Low | Low | Low | Moderate | Serious | Low | Serious |
| **Hakansson 2017 (P)**(41) | Moderate | Low | Moderate | Moderate | Moderate | Low | Low | Moderate |
| **Hakansson 2017 (H)**(41) | Serious | Low | Low | Moderate | Low | Moderate | Moderate | Serious |
| **Hong 2019**(42) | Serious | Low | Low | Low | Moderate | Moderate | Low | Serious |
| **Horvath 2013**(43) | Serious | Low | Low | NI | Low | Moderate | Low | Serious |
| **Hung 2018**(44) | NI | NI | NI | NI | Serious | Low | NI | Serious |
| **Isaacs 1999**(45) | Serious | Moderate | Low | NI | Low | Low | Low | Serious |
| **Jeffery 1998**(46) | Serious | Low | Low | NI | Low | Low | Low | Serious |
| **Johansson Gudjónsdóttir 2019**(47) | Serious | Low | Moderate | NI | Moderate | Moderate | Moderate | Serious |
| **Katz 1994**(48) | Serious | Low | Low | Low | Moderate | Moderate | Low | Serious |
| **Katz 1999**(49) | Serious | Low | Low | NI | Moderate | Moderate | Low | Serious |
| **Ko 2021**(50) | Serious | Serious | Moderate | NI | Moderate | Moderate | Moderate | Serious |
| **Lee 2021**(51) | Serious | Low | Low | Serious | Moderate | Moderate | Low | Serious |
| **Levine 1999**(52) | Serious | Low | Low | NI | Moderate | Low | Low | Serious |
| **Lin 2011**(52) | Serious | Low | Low | Low | Moderate | Moderate | Low | Serious |
| **Locksmith 1999**(53) | Moderate | Low | Low | NI | Moderate | Low | Low | Moderate |
| **Lopez Sastre 2005**(54) | Serious | Low | Low | NI | Low | Low | Low | Serious |
| **Lu 2022**(55) | Moderate | Low | Moderate | NI | Moderate | Serious | Low | Serious |
| **Lukacs 2012** (56) | Serious | Low | Low | NI | Moderate | Low | Low | Serious |
| **Ma 2018**(57) | Serious | Moderate | Moderate | Moderate | NI | Low | Moderate | Serious |
| **Main 2000 (P)**(58) | Moderate | Low | Low | Low | Low | Low | Low | Moderate |
| **Main 2000 (H)**(58) | Moderate | Low | Low | Low | Low | Low | Moderate | Moderate |
| **Matsubara 2007**(59) | Moderate | Serious | Low | NI | Moderate | Moderate | Low | Serious |
| **Matsubara 2013**(60) | Serious | Low | Low | NI | Moderate | Low | Low | Serious |
| **Mirsky 2020**(61) | Low | Low | Moderate | Moderate | Moderate | Moderate | Moderate | Moderate |
| **O'Sullivan 2019 (P)**(62) | Serious | Low | Low | NI | Low | Low | Low | Serious |
| **O'Sullivan 2019 (H)**(62) | Moderate | Moderate | Low | Moderate | Moderate | Low | Low | Moderate |
| **Petersen 2014**(63) | Moderate | Low | Moderate | NI | Moderate | Moderate | Low | Moderate |
| **Phares 2008 (P)**(64) | Serious | Low | Serious | NI | Moderate | Low | Moderate | Serious |
| **Phares 2008 (H)**(64) | Moderate | Moderate | Low | NI | Moderate | Low | NI | Moderate |
| **Poulain 1997**(65) | Serious | Low | Low | Moderate | Moderate | Serious | Low | Serious |
| **Puopolo 2010**(66) | Serious | Low | Low | NI | Moderate | Moderate | Low | Serious |
| **Reisner 2000**(67) | Low | Low | Low | Moderate | Low | Low | Low | Moderate |
| **Renner 2006**(68) | Serious | Low | Moderate | NI | Moderate | Moderate | Low | Serious |
| **Riley 2003**(69) | Serious | Serious | Moderate | Low | Moderate | Serious | Moderate | Serious |
| **Rottenstreich 2019**(70) | Serious | Low | Moderate | NI | Moderate | Moderate | Low | Serious |
| **Sakata 2012**(71) | Serious | Low | Low | NI | Moderate | Moderate | Low | Serious |
| **Schrag 2002 (P)**(72) | Low | Low | Moderate | Moderate | Moderate | Moderate | Low | Moderate |
| **Schrag 2002 (H)**(72) | Moderate | Moderate | Moderate | Low | Moderate | Low | Moderate | Moderate |
| **Schuchat 2002**(73) | Low | Low | Low | Moderate | Moderate | Moderate | Low | Moderate |
| **Share 2001**(74) | Serious | Low | Low | NI | Moderate | Moderate | Low | Serious |
| **Sutkin 2005**(75) | Serious | Low | Low | NI | Moderate | Moderate | Low | Serious |
| **Towers & Briggs 2002**(76) | Serious | Low | Low | NI | Low | Low | Low | Serious |
| **Trijbels-Smeulders 2006**(77) | Moderate | Low | Low | NI | Low | Low | Low | Moderate |
| **Trijbels-Smeulders 2007**(78) | Serious | Low | Low | NI | Moderate | Low | Low | Serious |
| **Trollfors 2022**(79) | Serious | Serious | Serious | NI | Moderate | Serious | Moderate | Serious |
| **Uy 2002**(80) | Low | Low | Low | Moderate | Moderate | Moderate | Low | Moderate |
| **van den Hoogen 2010**(81) | Serious | Low | Low | NI | Low | Low | Critical | Critical |
| **Van Dyke 2009**(82) | Moderate | Low | Low | Low | Moderate | Low | Low | Moderate |
| **Vergani 2002 (P)**(83) | Moderate | Low | Low | NI | Moderate | Moderate | Low | Moderate |
| **Vergani 2002 (H)**(83) | Moderate | Moderate | Moderate | Moderate | Moderate | Low | Serious | Moderate |
| **Wicker 2019**(84) | Serious | Low | Moderate | NI | Low | Low | Low | Serious |
| **Youden 2005**(85) | Low | Low | Low | Low | Low | Low | Low | Low |

Abbreviations**:** H: judgements from Hasperhoven 2020; NI: no information; P: judgements from Panneflek 2024; ROBINS-I: Risk Of Bias In Non-randomised Studies - of Interventions

### Figure 4.4 Robvis visual representation of ROBINS-I risk of bias

  
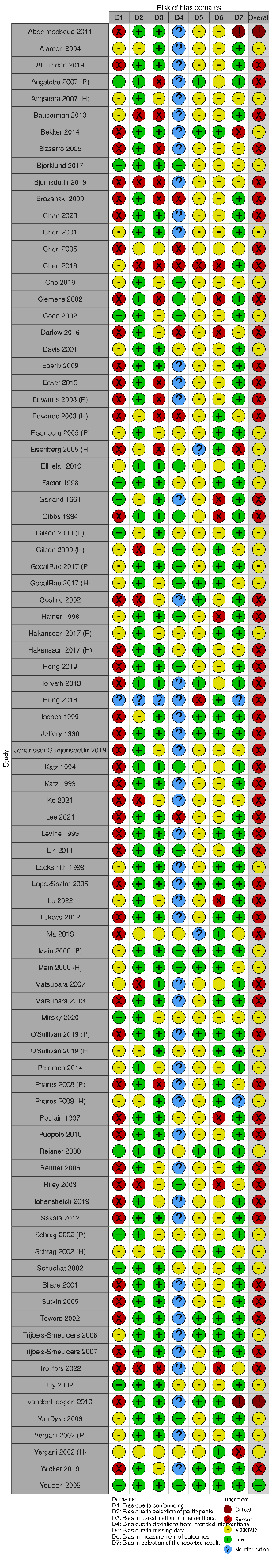


References

1. Alotaibi NM, Alroqi S, Alharbi A, Almutiri B, Alshehry M, Almutairi R, et al. Clinical Characteristics and Treatment Strategies for Group B Streptococcus (GBS) Infection in Pediatrics: A Systematic Review. *Medicina (Kaunas)* 2023; 59 7.

2. Braye K, Ferguson J, Davis D, Catling C, Monk A, Foureur M. Effectiveness of intrapartum antibiotic prophylaxis for early-onset group B Streptococcal infection: An integrative review. *Women Birth* 2018; 31 4:244-53.

3. da Silva HD, Kretli Winkelstroter L. Universal gestational screening for Streptococcus agalactiae colonization and neonatal infection - A systematic review and meta-analysis. *J Infect Public Health* 2019; 12 4:479-81.

4. Hasperhoven GF, Al-Nasiry S, Bekker V, Villamor E, Kramer B. Universal screening versus risk-based protocols for antibiotic prophylaxis during childbirth to prevent early-onset group B streptococcal disease: a systematic review and meta-analysis. *BJOG* 2020; 127 6:680-91.

5. Li QY, Wang DY, Li HT, Liu JM. Screening-based and Risk-based Strategy for the Prevention of Early-onset Group B Streptococcus/Non-group B Streptococcus Sepsis in the Neonate: A Systematic Review and Meta-analysis. *Pediatr Infect Dis J* 2020; 39 8:740-8.

6. Panneflek TJR, Hasperhoven GF, Chimwaza Y, Allen C, Lavin T, Te Pas AB, et al. Intrapartum antibiotic prophylaxis to prevent Group B streptococcal infections in newborn infants: a systematic review and meta-analysis comparing various strategies. *EClinicalMedicine* 2024; 74:102748.

7. Daniels J, Dixon EF, Gill A, Bishop J, D'Amico M, Ahmed K, et al. A rapid intrapartum test for group B Streptococcus to reduce antibiotic usage in mothers with risk factors: the GBS2 cluster RCT. *Health Technology Assessment (Winchester, England)* 2022; 26 12:1-82.

8. Daniels JP, Dixon E, Gill A, Bishop J, Wilks M, Millar M, et al. Rapid intrapartum test for maternal group B streptococcal colonisation and its effect on antibiotic use in labouring women with risk factors for early-onset neonatal infection (GBS2): cluster randomised trial with nested test accuracy study. *BMC Medicine* 2022; 20 1:9.

9. Kolkman DGE, Martin L, Jans S, Wouters M, van Dommelen P, Fleuren MAH, et al. Evaluation of women's worries in different strategies for the prevention of early onset group B streptococcal disease in neonates. *Midwifery* 2020; 86:102623.

10. Kolkman DGE, Rijnders MEB, Wouters M, Dommelen PV, de Groot CJM, Fleuren MAH. Adherence to three different strategies to prevent early onset GBS infection in newborns. *Women & Birth: Journal of the Australian College of Midwives* 2020; 33 6:e527-e34.

11. Abdelmaaboud M, Mohammed AF. Universal screening vs. risk-based strategy for prevention of early-onset neonatal Group-B streptococcal disease. *J Trop Pediatr* 2011; 57 6:444-50.

12. Alarcon A, Pena P, Salas S, Sancha M, Omenaca F. Neonatal early onset Escherichia coli sepsis: trends in incidence and antimicrobial resistance in the era of intrapartum antimicrobial prophylaxis. *Pediatr Infect Dis J* 2004; 23 4:295-9.

13. Al Luhidan L, Madani A, Albanyan EA, Al Saif S, Nasef M, AlJohani S, et al. Neonatal Group B Streptococcal Infection in a Tertiary Care Hospital in Saudi Arabia: A 13-year Experience. *Pediatr Infect Dis J* 2019; 38 7:731-4.

14. Angstetra D, Ferguson J, Giles WB. Institution of universal screening for Group B streptococcus (GBS) from a risk management protocol results in reduction of early-onset GBS disease in a tertiary obstetric unit. *Aust N Z J Obstet Gynaecol* 2007; 47 5:378-82.

15. Bauserman MS, Laughon MM, Hornik CP, Smith PB, Benjamin DK, Jr., Clark RH, et al. Group B Streptococcus and Escherichia coli infections in the intensive care nursery in the era of intrapartum antibiotic prophylaxis. *Pediatr Infect Dis J* 2013; 32 3:208-12.

16. Bekker V, Bijlsma MW, van de Beek D, Kuijpers TW, van der Ende A. Incidence of invasive group B streptococcal disease and pathogen genotype distribution in newborn babies in the Netherlands over 25 years: a nationwide surveillance study. *Lancet Infect Dis* 2014; 14 11:1083-9.

17. Bizzarro MJ, Raskind C, Baltimore RS, Gallagher PG. Seventy-five years of neonatal sepsis at Yale: 1928-2003. *Pediatrics* 2005; 116 3:595-602.

18. Bjorklund V, Nieminen T, Ulander VM, Ahola T, Saxen H. Replacing risk-based early-onset-disease prevention with intrapartum group B streptococcus PCR testing. *J Matern Fetal Neonatal Med* 2017; 30 3:368-73.

19. Bjornsdottir ES, Martins ER, Erlendsdottir H, Haraldsson G, Melo-Cristino J, Ramirez M, et al. Group B Streptococcal Neonatal and Early Infancy Infections in Iceland, 1976-2015. *Pediatr Infect Dis J* 2019; 38 6:620-4.

20. Brozanski BS, Jones JG, Krohn MA, Sweet RL. Effect of a screening-based prevention policy on prevalence of early-onset group B streptococcal sepsis. *Obstet Gynecol* 2000; 95 4:496-501.

21. Chan YTV, Lau SYF, Hui SYA, Ma T, Kong CW, Kwong LT, et al. Incidence of neonatal sepsis after universal antenatal culture-based screening of group B streptococcus and intrapartum antibiotics: A multicentre retrospective cohort study. *BJOG* 2023; 130 1:24-31.

22. Chen KT, Tuomala RE, Cohen AP, Eichenwald EC, Lieberman E. No increase in rates of early-onset neonatal sepsis by non-group B Streptococcus or ampicillin-resistant organisms. *Am J Obstet Gynecol* 2001; 185 4:854-8.

23. Chen KT, Puopolo KM, Eichenwald EC, Onderdonk AB, Lieberman E. No increase in rates of early-onset neonatal sepsis by antibiotic-resistant group B Streptococcus in the era of intrapartum antibiotic prophylaxis. *Am J Obstet Gynecol* 2005; 192 4:1167-71.

24. Cho CY, Tang YH, Chen YH, Wang SY, Yang YH, Wang TH, et al. Group B Streptococcal infection in neonates and colonization in pregnant women: An epidemiological retrospective analysis. *J Microbiol Immunol Infect* 2019; 52 2:265-72.

25. Clemens CJ, Gable EK. The development of a group B streptococcus prevention policy at a community hospital. *J Perinatol* 2002; 22 7:523-5.

26. Coco AS. Comparison of two prevention strategies for neonatal group B streptococcal disease. *J Am Board Fam Pract* 2002; 15 4:272-6.

27. Darlow BA, Voss L, Lennon DR, Grimwood K. Early-onset neonatal group B streptococcus sepsis following national risk-based prevention guidelines. *Aust N Z J Obstet Gynaecol* 2016; 56 1:69-74.

28. Davis RL, Hasselquist MB, Cardenas V, Zerr DM, Kramer J, Zavitkovsky A, et al. Introduction of the new Centers for Disease Control and Prevention group B streptococcal prevention guideline at a large West Coast health maintenance organization. *Am J Obstet Gynecol* 2001; 184 4:603-10.

29. Eberly MD, Rajnik M. The effect of universal maternal screening on the incidence of neonatal early-onset group B streptococcal disease. *Clin Pediatr (Phila)* 2009; 48 4:369-75.

30. Ecker KL, Donohue PK, Kim KS, Shepard JA, Aucott SW. The impact of group B Streptococcus prophylaxis on early onset neonatal infections. *J Neonatal Perinatal Med* 2013; 6 1:37-44.

31. Edwards RK, Jamie WE, Sterner D, Gentry S, Counts K, Duff P. Intrapartum antibiotic prophylaxis and early-onset neonatal sepsis patterns. *Infect Dis Obstet Gynecol* 2003; 11 4:221-6.

32. Eisenberg E, Craig AS, Gautam S, Khalil MM, Shaktour B, Schaffner W, et al. Beyond screening: identifying new barriers to early onset group B streptococcal disease prevention. *Pediatr Infect Dis J* 2005; 24 6:520-4.

33. El Helali N, Habibi F, Azria E, Giovangrandi Y, Autret F, Durand-Zaleski I, et al. Point-of-Care Intrapartum Group B Streptococcus Molecular Screening: Effectiveness and Costs. *Obstetrics & Gynecology* 2019; 133 2:276-81.

34. Factor SH, Levine OS, Nassar A, Potter J, Fajardo A, O'Sullivan MJ, et al. Impact of a risk-based prevention policy on neonatal group B streptococcal disease. *Am J Obstet Gynecol* 1998; 179 6 Pt 1:1568-71.

35. Garland SM. Early onset neonatal Group-B streptococcus (GBS) infection - associated obstetric risk-factors. *Aust N Z J Obstet Gynaecol* 1991; 31 2.

36. Gibbs RS, McDuffie RS, Jr., McNabb F, Fryer GE, Miyoshi T, Merenstein G. Neonatal group B streptococcal sepsis during 2 years of a universal screening program. *Obstet Gynecol* 1994; 84 4:496-500.

37. Gilson GJ, Christensen F, Romero H, Bekes K, Silva L, Qualls CR. Prevention of group B streptococcus early-onset neonatal sepsis: comparison of the Center for Disease Control and prevention screening-based protocol to a risk-based protocol in infants at greater than 37 weeks' gestation. *J Perinatol* 2000; 20 8 Pt 1:491-5.

38. Gopal Rao G, Townsend J, Stevenson D, Nartey G, Hiles S, Bassett P, et al. Early-onset group B Streptococcus (EOGBS) infection subsequent to cessation of screening-based intrapartum prophylaxis: findings of an observational study in West London, UK. *BMJ Open* 2017; 7 11:e018795.

39. Gosling IA, Stone PR, Grimwood K. Early-onset group B streptococcus prevention protocols in New Zealand public hospitals. *Aust N Z J Obstet Gynaecol* 2002; 42 4:362-4.

40. Hafner E, Sterniste W, Rosen A, Schuchter K, Plattner M, Asboth F, et al. Group B streptococci during pregnancy: a comparison of two screening and treatment protocols. *Am J Obstet Gynecol* 1998; 179 3 Pt 1:677-81.

41. Hakansson S, Lilja M, Jacobsson B, Kallen K. Reduced incidence of neonatal early-onset group B streptococcal infection after promulgation of guidelines for risk-based intrapartum antibiotic prophylaxis in Sweden: analysis of a national population-based cohort. *Acta Obstet Gynecol Scand* 2017; 96 12:1475-83.

42. Hong JY, . , Kim SH, ., Kim SM, al. e. Evaluation of the early onset neonatal sepsis according to two antenatal group B Streptococcus screening methods: risk-based versus universal screening. *Perinatology* 2019; 30 4:200-7.

43. Horvath B, Grasselly M, Bodecs T, Boncz I, Bodis J. Screening pregnant women for group B streptococcus infection between 30 and 32 weeks of pregnancy in a population at high risk for premature birth. *Int J Gynaecol Obstet* 2013; 122 1:9-12.

44. Hung LC, Kung PT, Chiu TH, Su HP, Ho M, Kao HF, et al. Risk factors for neonatal early-onset group B streptococcus-related diseases after the implementation of a universal screening program in Taiwan. *BMC Public Health* 2018; 18 1:438.

45. Isaacs D, Royle JA. Intrapartum antibiotics and early onset neonatal sepsis caused by group B Streptococcus and by other organisms in Australia. Australasian Study Group for Neonatal Infections. *Pediatr Infect Dis J* 1999; 18 6:524-8.

46. Jeffery HE, Moses Lahra M. Eight-year outcome of universal screening and intrapartum antibiotics for maternal group B streptococcal carriers. *Pediatrics* 1998; 101 1:E2.

47. Johansson Gudjónsdóttir M, Elfvin A, Hentz E, Adlerberth I, Tessin I, B. T. Changes in incidence and etiology of early-onset neonatal infections 1997-2017 - a retrospective cohort study in western Sweden. *BMC Pediatr* 2019; 19 1:490.

48. Katz VL, Moos MK, Cefalo RC, Thorp JM, Jr., Bowes WA, Jr., Wells SD. Group B streptococci: results of a protocol of antepartum screening and intrapartum treatment. *Am J Obstet Gynecol* 1994; 170 2:521-6.

49. Katz PF, Hibbard JU, Ranganathan D, Meadows W, Ismail M. Group B streptococcus: to culture or not to culture? *J Perinatol* 1999; 19 5:337-42.

50. Ko MH CH, Li ST, et al. An 18-year retrospective study on the epidemiology of early-onset neonatal sepsis - emergence of

uncommon pathogens. *Pediatr Neonatol* 2021; 62 5:491–8.

51. Lee J, Naiduvaje K, Chew KL, Charan N, Chan YH, Lin RT, et al. Preventing early-onset group B streptococcal sepsis: clinical risk factor-based screening or culture-based screening? *Singapore Med J* 2021; 62 1:34-8.

52. Levine EM, Ghai V, Barton JJ, Strom CM. Intrapartum antibiotic prophylaxis increases the incidence of gram-negative neonatal sepsis. *Infect Dis Obstet Gynecol* 1999; 7 4:210-3.

53. Locksmith GJ, Clark P, Duff P. Maternal and neonatal infection rates with three different protocols for prevention of group B streptococcal disease. *Am J Obstet Gynecol* 1999; 180 2 Pt 1:416-22.

54. Lopez Sastre JB, Fernandez Colomer B, Coto Cotallo GD, Ramos Aparicio A, Grupo de Hospitales C. Trends in the epidemiology of neonatal sepsis of vertical transmission in the era of group B streptococcal prevention. *Acta Paediatr* 2005; 94 4:451-7.

55. Lu IC, Chang YC, Chen YT, Lin HY, Chiu HY, Tsai ML, et al. Epidemiological evolution of early-onset neonatal sepsis over 12 years: A single center, population-based study in central Taiwan. *J Neonatal Perinatal Med* 2022; 15 3:575-82.

56. Lukacs SL, Schrag SJ. Clinical sepsis in neonates and young infants, United States, 1988-2006. *J Pediatr* 2012; 160 6:960-5 e1.

57. Ma TWL, Chan V, So CH, Hui ASY, Lee CN, Hui APW, et al. Prevention of early onset group B streptococcal disease by universal antenatal culture-based screening in all public hospitals in Hong Kong. *J Matern Fetal Neonatal Med* 2018; 31 7:881-7.

58. Main EK, Slagle T. Prevention of early-onset invasive neonatal group B streptococcal disease in a private hospital setting: the superiority of culture-based protocols. *Am J Obstet Gynecol* 2000; 182 6:1344-54.

59. Matsubara K, Kawai M, Nakahata T, Kato F, Tsukahara H, Yamakawa M, et al. Procedures for prevention of perinatal group B streptococcal diseases: a multicenter questionnaire survey of hospitals in the Kyoto Neonatal Disease Study Group, Japan. *J Infect Chemother* 2007; 13 1:59-62.

60. Matsubara K, Hoshina K, Suzuki Y. Early-onset and late-onset group B streptococcal disease in Japan: a nationwide surveillance study, 2004-2010. *Int J Infect Dis* 2013; 17 6:e379-84.

61. Mirsky R, Carpenter DM, Postlethwaite DA, Regenstein AC. Preventing early-onset group B streptococcal sepsis: is there a role for rescreening near term? *Journal of Maternal-Fetal & Neonatal Medicine* 2020; 33 22:3791-7.

62. O'Sullivan CP, Lamagni T, Patel D, Efstratiou A, Cunney R, Meehan M, et al. Group B streptococcal disease in UK and Irish infants younger than 90 days, 2014-15: a prospective surveillance study. *Lancet Infect Dis* 2019; 19 1:83-90.

63. Petersen KB, Johansen HK, Rosthoj S, Krebs L, Pinborg A, Hedegaard M. Increasing prevalence of group B streptococcal infection among pregnant women. *Dan Med J* 2014; 61 9:A4908.

64. Phares CR, Lynfield R, Farley MM, Mohle-Boetani J, Harrison LH, Petit S, et al. Epidemiology of invasive group B streptococcal disease in the United States, 1999-2005. *JAMA* 2008; 299 17:2056-65.

65. Poulain P, Betremieux P, Donnio PY, Proudhon JF, Karege G, Giraud JR. Selective intrapartum anti-bioprophylaxy of group B streptococci infection of neonates: a prospective study in 2454 subsequent deliveries. *Eur J Obstet Gynecol Reprod Biol* 1997; 72 2:137-40.

66. Puopolo KM, Eichenwald EC. No change in the incidence of ampicillin-resistant, neonatal, early-onset sepsis over 18 years. *Pediatrics* 2010; 125 5:e1031-8.

67. Reisner DP, Haas MJ, Zingheim RW, Williams MA, Luthy DA. Performance of a group B streptococcal prophylaxis protocol combining high-risk treatment and low-risk screening. *Am J Obstet Gynecol* 2000; 182 6:1335-43.

68. Renner RM, Renner A, Schmid S, Hoesli I, Nars P, Holzgreve W, et al. Efficacy of a strategy to prevent neonatal early-onset group B streptococcal (GBS) sepsis. *J Perinat Med* 2006; 34 1:32-8.

69. Riley L, Appollon K, Haider S, Chan-Flynn S, Cohen A, Ecker J, et al. "Real World" compliance with strategies to prevent early-onset group B streptococcal disease. *J Perinatol* 2003; 23 4:272-7.

70. Rottenstreich M, Rotem R, Bergman M, Farkash R, Schimmel MS, Samueloff A, et al. Assessment of maternal GBS colonization and early-onset neonatal disease rate for term deliveries: a decade perspective. *J Perinat Med* 2019; 47 5:528-33.

71. Sakata H. Evaluation of intrapartum antibiotic prophylaxis for the prevention of early-onset group B streptococcal infection. *J Infect Chemother* 2012; 18 6:853-7.

72. Schrag SJ, Zell ER, Lynfield R, Roome A, Arnold KE, Craig AS, et al. A population-based comparison of strategies to prevent early-onset group B streptococcal disease in neonates. *N Engl J Med* 2002; 347 4:233-9.

73. Schuchat A, Roome A, Zell ER, Linardos H, Zywicki S, O'Brien KL. Integrated monitoring of a new group B streptococcal disease prevention program and other perinatal infections. *Matern Child Health J* 2002; 6 2:107-14.

74. Share L, Chaikin S, Pomeranets S, Kiwi R, Jacobs M, Fanaroff AA. Implementation of guidelines for preventing early onset group B streptococcal infection. *Semin Perinatol* 2001; 25 2:107-13.

75. Sutkin G, Krohn MA, Heine RP, Sweet RL. Antibiotic prophylaxis and non-group B streptococcal neonatal sepsis. *Obstet Gynecol* 2005; 105 3:581-6.

76. Towers CV, Briggs GG. Antepartum use of antibiotics and early-onset neonatal sepsis: the next 4 years. *Am J Obstet Gynecol* 2002; 187 2:495-500.

77. Trijbels-Smeulders MA, Kimpen JL, Kollee LA, Bakkers J, Melchers W, Spanjaard L, et al. Serotypes, genotypes, and antibiotic susceptibility profiles of group B streptococci causing neonatal sepsis and meningitis before and after introduction of antibiotic prophylaxis. *Pediatr Infect Dis J* 2006; 25 10:945-8.

78. Trijbels-Smeulders M, de Jonge GA, Pasker-de Jong PC, Gerards LJ, Adriaanse AH, van Lingen RA, et al. Epidemiology of neonatal group B streptococcal disease in the Netherlands before and after introduction of guidelines for prevention. *Arch Dis Child Fetal Neonatal Ed* 2007; 92 4:F271-6.

79. Trollfors B MF, Gudjonsdottir MJ, et al. . Group B streptococcus - a pathogen not restricted to neonates. *IJID Reg* 2022; 4:171-5.

80. Uy IP, D'Angio CT, Menegus M, Guillet R. Changes in early-onset group B beta hemolytic streptococcus disease with changing recommendations for prophylaxis. *J Perinatol* 2002; 22 7:516-22.

81. van den Hoogen A, Gerards LJ, Verboon-Maciolek MA, Fleer A, Krediet TG. Long-term trends in the epidemiology of neonatal sepsis and antibiotic susceptibility of causative agents. *Neonatology* 2010; 97 1:22-8.

82. Van Dyke MK, Phares CR, Lynfield R, Thomas AR, Arnold KE, Craig AS, et al. Evaluation of universal antenatal screening for group B streptococcus. *N Engl J Med* 2009; 360 25:2626-36.

83. Vergani P, Patane L, Colombo C, Borroni C, Giltri G, Ghidini A. Impact of different prevention strategies on neonatal group B streptococcal disease. *Am J Perinatol* 2002; 19 6:341-8.

84. Wicker E, Lander F, Weidemann F, Hufnagel M, Berner R, Krause G. Group B Streptococci: Declining Incidence in Infants in Germany. *Pediatr Infect Dis J* 2019; 38 5:516-9.

85. Youden L, Downing M, Halperin B, Scott H, Smith B, Halperin SA. Group B streptococcal testing during pregnancy: survey of postpartum women and audit of current prenatal screening practices. *J Obstet Gynaecol Can* 2005; 27 11:1006-12.
