# Supplementary material for: Benefits and Harms of Antenatal/Intrapartum Screening for Maternal Group B Streptococcus and Use of Intrapartum Antibiotic Prophylaxis Versus Risk‐Based Protocols or No Intervention: A Rapid Review
Source: Acta Paediatr. 2026 Apr 30;115(8):1598–610. doi: 10.1111/apa.70568 (PMC13371836; doi:10.1111/apa.70568)
Supplement: Supplementary file 5 — Data S5: Excluded studies. [file APA-115-1598-s013.docx]

## Supplementary materials File 5. Excluded studies

### Table 5.1. Summary of excluded reviews with reasons (Phase one)

| **Author**  **(reference)** | **Published Year** | **Aim or objective (verbatim)** | **Reason for Exclusion** | **Additional Notes** |
| --- | --- | --- | --- | --- |
| **Abde  (1)** | 2024 | To investigate the association between untreated/treated ASB (>=105 colony-forming units (cfu) of the same bacteria per ml urine in two consecutive voided cultures without any symptoms) and pregnancy outcomes (pyelonephritis, chorioamnionitis, prelabour rupture of membranes (PROM)), and birth outcomes (preterm birth (PTB), low birth weight (LBW) and small for gestational age (SGA)). The impact of the most serious pathogens E. coli and Group B streptococci (GBS) on these outcomes was also examined. | Not focussed on the effectiveness of screening | Bibliography checked to ensure that any relevant primary studies were identified and included in the synthesis to address RQ2. |
| **Adejumo (2)** | 2019 | To review and appraise the practices of IAP in Sub Saharan Africa | Abstract only - no full text available. | Authors contacted by email to see if the full text was published. |
| **Allen (3)** | 2018 | To provide information regarding the management of group B streptococcal (GBS) bacteriuria to midwives, nurses, and physicians who are providing obstetrical care. | Wrong study design; does not meet the criteria for a SR |  |
| **Alotaibi (4)** | 2023 | The aim of this systematic review was to summarize the clinical characteristics and treatment strategies for GBS, with a focus on antibiotics. | Excluded after quality assessment |  |
| **Alshengeti (5)** | 2022 | This systemic review aimed to assess the prevalence of GBS colonization among pregnant women and the incidence of neonatal GBS sepsis in Saudi Arabia. | Not focussed on the effectiveness of screening | Bibliography checked to ensure that any relevant primary studies were identified and included in the synthesis to address RQ2. |
| **Angelescu (6)** | 2016 | Our systematic review had three objectives: firstly, to assess the patient-relevant benefits and harms of screening for ASB versus no screening; secondly, to compare the benefits and harms of different screening strategies; and thirdly, in case no reliable evidence on the overarching screening question was identified, to determine the benefits and harms of treatment of ASB. | Wrong population or not GBS | Bibliography checked to ensure that any relevant primary studies were identified and included in the synthesis to address RQ2. |
| **Ashary (7)** | 2022 | Preterm birth is a major cause of neonatal morbidity and mortality. Vaginal colonization of Group B Streptococci (GBS) is associated with increased risk of preterm birth. However, the association of GBS colonization and preterm births in the Indian context remains an unrecognized problem | Not focussed on the effectiveness of screening |  |
| **Biondi (8)** | 2019 | To determine modern rates of bacteremia and bacterial meningitis in febrile neonates and infants in the second month of life presenting to an ambulatory setting. | Not focussed on the effectiveness of screening |  |
| **Borg (9)** | 2023 | This review aimed to determine whether sample self-collection is as accurate as provider-collection for detection of group B streptococcus colonisation in pregnancy and whether a strategy of self-collection compared to provider-collection might improve maternal and neonatal health outcomes. | Not focussed on the effectiveness of screening |  |
| **Bouchghoul (10)** | 2020 | To evaluate safety of home care, clinical and biological initial examination and effectiveness of prophylactic antibiotic in preventing maternal and neonatal infectious complications in women with term prelabour rupture of membranes | Study not published in English |  |
| **Braye (11)** | 2018 | This review considers clinical trials and observational studies analysing the effectiveness of intrapartum antibiotic prophylaxis. | Described as an integrative review but meets criteria for a SR. Excluded following quality assessment |  |
| **Chelkeba (12)** | 2022 | This review is aimed at providing contemporary bacterial profile and antimicrobial resistance pattern among pregnant women with significant bacteriuria. | Not focussed on the effectiveness of screening |  |
| **Constantinou (13)** | 2024 | To conduct a rapid review to synthesise evidence on women and health professionals': (1) knowledge and awareness of; (2) preferences for; and (3) acceptability of GBS screening programmes, and (4) how feasible they are to implement. | Not focussed on the effectiveness of screening | Bibliography checked to ensure that any relevant primary studies were identified and included in the synthesis to address RQ2. |
| **da Silva (14)** | 2019 | The review aimed to analyze the evidence of the correlation between universal screening for Streptococcus agalactiae colonization in pregnant women and early onset Group B neonatal infection. | Excluded following quality assessment |  |
| **de Souza (15)** | 2023 | Data on UTI prevalence and bacterial profile in Latin American pregnant women remain scarce, necessitating the present systematic review to address this issue. | Not focussed on the effectiveness of screening |  |
| **Dong (16)** | 2020 | This review provides an updated summary regarding the epidemiology, pathogen profile, infectious work-up, and empirical treatment of neonatal sepsis within and beyond China. | Wrong study design; does not meet the criteria for a SR |  |
| **Emaneini (17)** | 2018 | Group B Streptococcus (GBS or S. agalactiae) is a major cause of severe disease in neonates. In perinatal infections or early-onset disease, GBS is transmitted vertically to the newborn from the birth canal during labour and delivery. Limited information is available on the prevalence of GBS recto-vaginal colonization among pregnant women in Iran. | Not focussed on the effectiveness of screening |  |
| **Feuerschuette (18)** | 2018 | The aim of this study was to determine the sensitivity and specificity of RT-PCR when compared to the reference standard culture in selective broth media collected from rectovaginal tract from labouring women. | Not focussed on the effectiveness of screening |  |
| **Fitzpatrick (19)** | 2022 | The aim of this systematic literature review was to estimate the diagnostic yields of maternal TORCH serology in pregnancy after detection of ultrasound abnormalities | Wrong population or not GBS | “TORCH” describes a group of congenital infections including toxoplasmosis, rubella, cytomegalovirus (CMV), and herpes simplex virus (HSV) |
| **Ghia (20)** | 2021 | We performed a narrative review of the available literature (published in the last 10 years) on the epidemiology of GBS, using PubMed and Google Scholar, to understand its impact in India and evaluate potential strategies to prevent the disease in the high-risk population, that is, neonates. | Wrong study design; does not meet the criteria for a SR |  |
| **Gizachew (21)** | 2019 | This meta-analysis was aimed to determine the pooled prevalence of colonization, antibiotic resistance and serotype profiles of GBS reported in Africa. | Not focussed on the effectiveness of screening |  |
| **Glaser (22)** | 2021 | To provide a review of neonatal sepsis by identifying its associated risk factors and most common causative pathogens, reviewing features of the term and preterm neonatal immune systems that increase vulnerability to infection, describing previous and the most current management recommendations, and discussing relevant implications for the neonatal nurse and novice neonatal nurse practitioner. | Wrong study design; does not meet the criteria for a SR |  |
| **Han (23)** | 2021 | Group B Streptococcal (GBS) infection is the primary agent of neonatal morbidity and mortality. Rapid and simple methods to detect GBS are Xpert GBS and GBS LB assays based on real-time polymerase chain reaction (PCR). However, since the diagnostic accuracy of the two techniques in diagnosing GBS remains unclear, we designed this study to appraise the diagnostic accuracy of the aforementioned. | Not focussed on the effectiveness of screening |  |
| **Hanson (24)** | 2022 | To systematically review and meta-analyse studies of the efficacy of probiotics to reduce antenatal Group B Streptococcus (GBS) colonisation. | Not focussed on the effectiveness of screening |  |
| **Hasperhoven (25)** | 2020 | Authors response to letter received re: re: Universal screening versus risk-based protocols for antibiotic prophylaxis during childbirth to prevent early-onset group B streptococcal disease: a systematic review and meta-analysis | Wrong study design; does not meet the criteria for a SR |  |
| **Hasperhoven (26)** | 2020 | Authors response to letter received re: re: Universal screening versus risk-based protocols for antibiotic prophylaxis during childbirth to prevent early-onset group B streptococcal disease: a systematic review and meta-analysis | Wrong study design; does not meet the criteria for a SR |  |
| **Huang (27)** | 2019 | Group B Streptococcus (GBS) is a leading cause of neonatal sepsis and an important cause of maternal disease in many countries; however, no accurate population-based epidemiological data on GBS is yet available in mainland China. In this systematic literature review, we obtained published data regarding the maternal GBS colonization rate, incidence of invasive GBS disease in infants, clinical screening, and the associated GBS typing and clinical outcomes in China. | Not focussed on the effectiveness of screening |  |
| **Hueth (28)** | 2022 | This systematic review and meta-analysis synthesized the literature on the association between the multiplex ME panel and length of hospital stay (LOS), length of acyclovir therapy, and days with antibiotics. | Not focussed on the effectiveness of screening |  |
| **Iadeluca (29)** | 2017 | Group B streptococcus (GBS) rectovaginal carriage during late pregnancy and birth is associated with neonatal GBS disease which is life threatening, and survivors may have long lasting sequelae. GBS screening of pregnant women and subsequent intrapartum antibiotic prophylaxis (IAP) has proved effective at reducing GBS early onset disease (EOD) in areas where it is universally applied. Despite the recommendation of these preventative measures, the incidence of neonatal GBS, particularly late-onset disease (LOD), is increasing. We therefore conducted a literature review to assess the adherence of European countries to GBS screening and IAP. | Abstract only - no full text available. | Authors contacted by email to see if the full text was published. |
| **Impens (30)** | 2019 | Better knowledge about pathogens causing neonatal sepsis in SSA, as well as their antibiotic susceptibility patterns, is needed to design and implement strategies based on pathogens that locally predominate. Therefore, a systematic literature review was conducted, with the objective of providing an overview of these pathogens and their antibiotic susceptibility patterns. | Abstract only - no full text available. | Authors confirm no full text was published. |
| **Jefferies (31)** | 2017 | Early-onset neonatal bacterial sepsis (EOS) is sepsis occurring within the first 7 days of life. This statement provides updated recommendations for the care of term (>=37 weeks' gestational age) newborns at risk of EOS, during the first 24 hours of life. | Wrong study design; does not meet the criteria for a SR |  |
| **Jury (32)** | 2021 | A scoping review comparing peripartum prophylactic antimicrobial use in low- and middle-income countries (LMICs) with WHO recommendations for prevention and treatment of maternal peripartum infection. | Wrong study design; does not meet the criteria for a SR | Bibliography checked to ensure that any relevant primary studies were identified and included in the synthesis to address RQ2. |
| **Kahwati (33)** | 2020 | To update the evidence on screening and treatment of asymptomatic bacterial vaginosis in pregnancy for the US Preventive Services Task Force. | Wrong population or not GBS |  |
| **Karampatsas (34)** | 2022 | Group B streptococcal (GBS) infection remains one of the most significant causes of late-onset sepsis and meningitis (LOGBS) among young infants. However, transmission routes and risk factors for LOGBS are not yet fully understood. We conducted systematic reviews on clinical risk factors previously reported in the literature (prematurity, low birth weight [<2500 g], antenatal colonization, multiple-gestation pregnancy, maternal age <20 years, male infant sex, intrapartum fever, prolonged rupture of membranes) and meta-analyses to determine pooled estimates of risk. | Not focussed on the effectiveness of screening |  |
| **Khademi (35)** | 2020 | The aim of this study was to determine the prevalence of S. agalactiae antibiotic resistance in Iranian patients, especially among pregnant women. | Not focussed on the effectiveness of screening |  |
| **Koucky (36)** | 2022 | Maternal sepsis is a leading cause of maternal and neonatal mortality. Despite the availability of management protocols, there is disparity in case fatality rates for pregnancy-related sepsis compared to other maternity-related complications. The main aim of this systematic review was to assess concordance between international evidence-based guidelines for the prevention and management of childbirth-related bacterial infections. | Not focussed on the effectiveness of screening |  |
| **Kwatra (37)** | 2016 | The most important risk factor for early-onset (babies younger than 7 days) invasive group B streptococcal disease is rectovaginal colonisation of the mother at delivery. We aimed to assess whether differences in colonisation drive regional differences in the incidence of early-onset invasive disease. | Not focussed on the effectiveness of screening |  |
| **Lamont (38)** | 2020 | Letter regarding: Universal screening versus risk-based protocols for antibiotic prophylaxis during childbirth to prevent early-onset group B streptococcal disease: a systematic review and meta-analysis | Wrong study design; does not meet the criteria for a SR |  |
| **Le Doare (39)** | 2019 | In autumn 2016, the UK Department of Health (now Department of Health and Social Care) convened 2 meetings to discuss how to address research evidence gaps in order to minimize the impact of infant group B streptococcus (GBS) disease in the United Kingdom. At that meeting, a number of research priorities were highlighted, including improving the screening for GBS colonization in pregnant women, offering intrapartum antibiotic prophylaxis and point-of-care testing, and understanding the effect of widespread intrapartum antibiotic use on long-term infant health. Further discussions involved investigating the feasibility of a large prospective study of pregnant women and their infants in order to understand the role of antibodies in the protection against GBS disease in infancy following maternal exposure to GBS colonization. Here, we summarize the research uncertainties identified at that meeting | Wrong study design; does not meet the criteria for a SR |  |
| **Le Doare (40)** | 2017 | Intrapartum antibiotic chemoprophylaxis (IAP) prevents most early-onset group B streptococcal (GBS) disease. However, there is no description of how IAP is used around the world. This article is the sixth in a series estimating the burden of GBS disease. Here we aimed to review GBS screening policies and IAP implementation worldwide. | Not focussed on the effectiveness of screening |  |
| **Lemma (41)** | 2022 | The goal of this review was to determine the pooled prevalence Group B streptococcus colonization and antimicrobial susceptibility among Ethiopian pregnant women | Not focussed on the effectiveness of screening |  |
| **Ludmir (42)** | 2018 |  | Wrong study design; does not meet the criteria for a SR | Abstract only – authors not contacted as not SR |
| **Malik (43)** | 2023 | This systematic review aims to investigate the cost-effectiveness and feasibility of providing universal screening for GBS to all pregnant women and to determine if it should be offered by the NHS | Abstract only - no full text available. | Authors contacted: full paper under review but not yet accepted |
| **Mercado-Evans (44)** | 2024 | This systematic review and meta-analysis aims to address current conflicting findings and determine whether GDM should be clinically considered as a risk factor for maternal GBS colonization. | Not focussed on the effectiveness of screening | Bibliography checked to ensure that any relevant primary studies were identified and included in the synthesis to address RQ2 |
| **Money (45)** | 2018 | To review the evidence in the literature and to provide recommendations on the management of pregnant women in labour for the prevention of early-onset neonatal group B streptococcal disease. The key revisions in this updated guideline include changed recommendations for regimens for antibiotic prophylaxis, susceptibility testing, and management of women with pre-labour rupture of membranes. | Wrong study design; does not meet the criteria for a SR |  |
| **Money (46)** | 2016 | To review the evidence in the literature and to provide recommendations on the management of pregnant women in labour for the prevention of early-onset neonatal group B streptococcal disease. The key revisions in this updated guideline include changed recommendations for regimens for antibiotic prophylaxis, susceptibility testing, and management of women with pre-labour rupture of membranes. | Wrong study design; does not meet the criteria for a SR |  |
| **Moradi (47)** | 2024 | The objective of this study is to synthesise the available evidence of IAP exposure on autoimmune diseases and obesity prevalence in childhood and microbial diversity in babies. | Abstract only - no full text available. | Authors contacted: full paper under review but not yet published |
| **Nadeau (48)** | 2022 | Using systematic review and meta-analysis methodology, this study sought to evaluate the agreement between vaginal-perianal or vaginal-perineal culture-based screening for maternal anogenital GBS colonization in the third trimester pregnant patient and the typically utilized vaginal-rectal culture-based screening. | Not focussed on the effectiveness of screening |  |
| **Nanduri (49)** | 2016 | Intrapartum antibiotic prophylaxis (IAP) recommendations for prevention of early-onset neonatal group B streptococcal (GBS) disease were first implemented in the 1990s. Despite declines in newborn disease following these and subsequent recommendations, GBS remains the leading cause of neonatal sepsis and meningitis in the United States. We analysed GBS disease trends among young infants from 2006 through 2014 and estimated the potential impact of serotype-specific vaccines. | Wrong study design; does not meet the criteria for a SR (abstract) |  |
| **Odubamowo (50)** | 2023 | A systematic review of the test accuracy of a self-collected swab compared with a health-care professional collected swab in the diagnosis of GBS colonisation. | Not focussed on the effectiveness of screening |  |
| **Pangerl (51)** | 2021 | This literature review uses a systematic approach and aims to provide a synthesis of what is known about compliance with Group B Streptococcus screening protocols in a variety of global settings, including maternity homes, private obstetric practice, and hospital clinical environments. | Not focussed on the effectiveness of screening |  |
| **Paul (52)** | 2023 | Intrapartum antibiotic prophylaxis (IAP) is currently the only recommended preventive approach against clinical consequences of maternal Group B Streptococcus (GBS) colonization. In this review, we discuss new findings of total perinatal GBS burden and relative effectiveness of differing targeting of IAP, notably microbiology-based and risk factor-based screening, including potential limitations. Finally, we provide updates on maternal GBS vaccines and their potential cost-effectiveness in disease reduction. | Wrong study design; does not meet the criteria for a SR |  |
| **Peng (53)** | 2025 | This study aimed to determine the performance of RT PCR of GBS screening in pregnant women under different situations, especially compared to different reference methods (culture or composite standards) and preprocessing before detection (directly or enrichment). | Not focussed on the effectiveness of screening |  |
| **Ren (54)** | 2021 | This study aimed to perform a meta-analysis to assess the relationship between the intrapartum antibiotic prophylaxis in colonized pregnant women with group B Streptococcus and neonatal infection. | Unable to retrieve full text |  |
| **Rezaei (55)** | 2021 | To give an overview of the recently reported literature on the aetiologies of meningitis and encephalitis in western sub-Saharan Africa | Wrong study design; does not meet the criteria for a SR |  |
| **Rogozinska (56)** | 2016 | To estimate the accuracy of onsite tests to detect asymptomatic bacteriuria among pregnant women. | Not focussed on the effectiveness of screening |  |
| **Ronzoni (57)** | 2022 | To provide clear and concise guidelines for the diagnosis and management of preterm prelabour rupture of membranes (PPROM) | Wrong study design; does not meet the criteria for a SR |  |
| **Sadeh (58)** | 2020 | The aim of this review was to summarize all of the relevant articles published to highlight the prevalence of group B Streptococcus colonization or infection in different regions of Iran. | Not focussed on the effectiveness of screening |  |
| **Seedat (59)** | 2017 | In this systematic review, we appraised and synthesised the evidence on the adverse events of IAP in the mother and/or her child. | Not focussed on GBS screening |  |
| **Seedat (60)** | 2018 | We reviewed whether bacterial load and molecular markers are associated with GBS vertical transmission and progression to EOGBS. | Not focussed on the effectiveness of screening |  |
| **Seedat (61)** | 2018 | The objectives are as follows: The primary objective is to compare the diagnostic accuracy of commercially available real-time polymerase chain reaction (PCR) tests and antenatal culture tests for diagnosing group B Streptococcus (GBS) colonisation in pregnant women during labour | Not focussed on the effectiveness of screening | Cochrane review protocol |
| **Seedat (62)** | 2017 | This review on universal GBS screening for pregnant women was undertaken to assist NSC policy decision-making. | Abstract only | Full text assessed |
| **Senat (63)** | 2020 | To determine the management of patients with term prelabour rupture of membranes. | Study not published in English |  |
| **Tansarli (64)** | 2020 | The aim was to evaluate the meningitis/encephalitis (ME) panel in a diagnostic test accuracy review. | Wrong population |  |
| **Turrentine (65)** | 2016 | The objective of this study was to evaluate the risk of recurrent group B streptococcus (GBS) colonization in a subsequent pregnancy and to assess clinical characteristics that influence this risk. | Not focussed on the effectiveness of screening |  |
| **Walker (66)** | 2020 |  | Wrong study design; does not meet the criteria for a SR | Mini commentary |
| **Walker (67)** | 2021 |  | Wrong study design; does not meet the criteria for a SR | Commentary on clinical practice uncertainties |
| **Wingert (68)** | 2019 | To systematically review screening and treatment effectiveness, and patient preferences, to inform recommendations by the Canadian Task Force on Preventive Health Care on screening for asymptomatic bacteriuria in pregnancy. | Not focussed on the effectiveness of screening |  |
| **Wu (69)** | 2025 | The aim of this study was to provide a basis for empirical antibiotic selection by comprehensively searching Chinese and non-Chinese databases for studies related to neonatal sepsis pathogenesis conducted in China and synthesizing all the results of the studies conducted in hospitals in China during the period under study. | Not focussed on the effectiveness of screening |  |
| **Yektakooshali (70)** | 2018 | The present study was carried out to estimate the prevalence of GBS colonization in pregnant women in Iran. | Not focussed on the effectiveness of screening |  |
| **Yudin (71)** | 2017 | To review the evidence and provide recommendations on screening for and management of bacterial vaginosis in pregnancy. | Wrong study design; does not meet the criteria for a SR |  |
| **Yudin (72)** | 2017 | To review the evidence and provide recommendations on the use of antibiotics in preterm premature rupture of the membranes (PPROM). | Wrong study design; does not meet the criteria for a SR |  |

**Abbreviations:** ASB: asymptomatic bacteriuria; cfu: colony forming units; EOD: early onset disease; EOGBS: early-onset GBS disease; EOS: early-onset sepsis; GBS: Group B streptococci; GDM: gestational diabetes mellitus; IAP: intrapartum antibiotic prophylaxis; LBW: low birth weight; LMICs: low- and middle-income countries; LOD: late-onset disease; LOGBS: late-onset GBS; LOS: length of hospital stay; ME: meningitis/encephalitis; NSC: National Screening Committee; PPROM: preterm prelabour rupture of membranes; PROM: prelabour rupture of membranes; PTB: preterm birth; RQ: research question; RT-PCR: reverse transcription polymerase chain reaction; SGA: small for gestational age; SR: systematic review; SSA: sub- Saharan Africa; UK: United Kingdom; US: United States of America; UTI: urinary tract infection

### Table 5.2. Studies excluded following updated searches (Phase two, from 2019-2025)

| **Author**  **(reference)** | **Published Year** | **Aim or objective (verbatim)** | **Reason for Exclusion** | **Additional Notes** |
| --- | --- | --- | --- | --- |
| **Abrok (73)** | 2020 | In this study, the results of the GBS screening among pregnant women performed between 2012 and 2018 (n = 19267) are presented. For the GBS positive samples, the antibiotic susceptibility of the isolated strains was also tested (n = 3554). | No comparator |  |
| **Achten (74)** | 2020 | The study aimed to evaluate the extent of influence exerted by risk-based GBS screening on management recommendations by the EOS calculator. | Wrong comparator |  |
| **Al Luhidan (75)** | 2019 | This study determined the incidence and burden of GBS infection among neonates in association with maternal GBS screening. | Primary study already identified and included from Overview |  |
| **Alaaraj (76)** | 2022 | A quality improvement project applying the Plan-Do-Study-Act model was conducted to test the efficacy of a proposed protocol to lower the rate of antibiotics administration. | LMIC |  |
| **Ali (77)** | 2022 | In this study, we aim to assess the prevalence, microbiological characteristics as well as clinical outcomes of invasive GBS disease from all ages groups at Hamad Medical Corporation (HMC), Qatar. | Conference abstract | Full text assessed |
| **Alshengeti (78)** | 2020 | The present study aimed to assess the knowledge, attitude and current practices of pregnant women towards GBS screening in Al-Madinah City, Saudi Arabia. | No comparator | Focus is on knowledge - not effectiveness and not clear that there are two groups |
| **Andreasen (79)** | 2019 | To compare the diagnostic performance of BD MAX and GenomEra PCR assays for a rapid PCR detection of vaginal carriage of group B streptococci at delivery. | No comparator |  |
| **Andrikopoulou (80)** | 2019 | To analyze trends in unindicated antibiotic use during vaginal delivery hospitalization. | No comparator |  |
| **Antonello (81)** | 2020 | The objective of this study was to evaluate the effectiveness of cefazolin prophylactic regimen for GBS disease, comparing it to the established penicillin-based protocols, given the opportunity provided by the temporary unavailability of first-choice antibiotics in Brazil. | LMIC |  |
| **Assabahi (82)** | 2022 | The primary objective of this research was to evaluate institutional compliance to these guidelines and secondarily, clinical outcomes. | No comparator |  |
| **Aziz (83)** | 2018 | We sought to determine if antenatal probiotic supplementation during second and third trimesters reduces maternal GBS rectovaginal colonization in pregnant women at 35-37 weeks' gestational age. | Conference abstract |  |
| **Baldwin (84)** | 2021 | Investigation of a local increase in invasive GBS cases in infants. | Conference abstract |  |
| **Bassaw (85)** | 2019 | The objectives of this study conducted in a low-resourced country were to determine the feasibility and acceptability among pregnant women of universal screening for group B streptococcus (GBS), the prevalence of GBS, to calculate the risk of vertical transmission, and to study the neonatal outcome of early onset group B streptococcus disease (EOGBSD). | No comparator |  |
| **Bauer (86)** | 2019 | This study aims to evaluate the performance characteristics of the systemic inflammatory response syndrome (SIRS), quick Sequential [Sepsis-related] Organ Failure Assessment (qSOFA), and maternal early warning (MEW) criteria for identifying cases of impending sepsis in parturients. | Not focussed on GBS screening |  |
| **Belhadi (87)** | 2020 | The objective of this study was to determine the maternal risk of maternal carriage of group B Streptococcus over time, to offer reliable epidemiological data to the health staff working at maternity Meriem Bouatoura, Batna (Northeast Algeria) or even all maternity hospitals in Algeria. | LMIC |  |
| **Berardi (88)** | 2019 | Group B streptococcus (GBS) early-onset sepsis (EOS) has declined after widespread intrapartum antibiotic prophylaxis. However, strategies for preventing EOS may differ across countries. The analysis of their strategies allows to compare the effectiveness of prevention in different countries and suggests opportunities for improvement. | Wrong study design |  |
| **Berardi (89)** | 2020 | Intrapartum antibiotic prophylaxis (IAP) prevents group B streptococcus (GBS) early-onset disease (EOD). No European study evaluates the relative impact of risk factors (RFs) for EOD after a screening-based strategy and widespread IAP use We aimed to evaluate the risks of EOD in an Italian region where a screening-based strategy for preventing EOD was implemented. | Wrong study design |  |
| **Berardi (90)** | 2021 | We retrospectively investigated mother-to-infant transmission of group B Streptococcus (GBS) in 98 cases of late onset disease reported during 2007-2018 by a network in Italy. | No comparator |  |
| **Berardi (91)** | 2018 | In the current commentary we provide area-based data concerning the prevention of EOS in 2 northern Italian regions, and we detail the results of their strategy for managing healthy-appearing newborns at risk for EOS. | Wrong study design | Commentary - could be linked to the other Berardi papers but excluded based on wrong study design |
| **Bjorklund (92)** | 2024 | The aim of this study was to evaluate the effect of SBP as a public health intervention on the epidemiology of early-onset GBS infections. | Primary study already identified and included from Overview |  |
| **Blanquart (93)** | 2024 | The main objective of this study was to evaluate the impact of the results of GBS-PCR on monitoring modifications in newborns of mothers with unknown GBS status. The secondary objectives were to confirm the feasibility of a GBS-PCR-based screening method in everyday practice and to evaluate the impact of GBS-PCR results on the modification of intrapartum antibiotic therapy in pregnant women. | No comparator |  |
| **Bogiel (94)** | 2022 | This research investigated the usefulness of Centers for Disease Control and Prevention (CDC) protocol for S. agalactiae DNA detection in 250 samples of recto-vaginal swabs collected from pregnant women (at 35-37 weeks of gestation) and pre-cultured overnight in liquid medium. | No comparator |  |
| **Bonney (95)** | 2019 | There are few published data that describe peripartum antibiotic exposure to mothers and babies in a hospital context. Such data is important in order to understand patterns of antibiotic resistance, and epidemiological associations between antibiotic exposure and atopic illness. | Conference abstract | Authors confirmed no full text published |
| **Bramugy (96)** | 2022 | We present data on iGBS from 2 decades of surveillance in Manhica, Mozambique, with a focus on NDI. | LMIC |  |
| **Braye (97)** | 2021 | Compliance with our local guideline, referred to here as 'the guideline'. | No comparator |  |
| **Braye (98)** | 2019 | To describe management of maternal GBS colonisation in one local health district using universal screening and assess rates of EOGBS over time. | No comparator |  |
| **Brigtsen (99)** | 2022 | The aim of this study was to determine whether GBS colonization at delivery is associated with increased risk of maternal peripartum infection. | No comparator | Cohort stratified into colonised vs uncolonised; focus is on risk factor association not screening programme |
| **Camus (100)** | 2021 | The aim was first to show non-inferiority of VSS compared with VCS to screen for GIs, STIs, and GBS; second to determine the feasibility of VSS. | No comparator | Same women but comparing two sample approaches: self-collected vs HCP collected |
| **Capraro (101)** | 2020 | We hypothesized that there may be an association between antibiotic exposure during pregnancy and GBS colonization and/or the presence of inducible clindamycin resistance (iCLI-R) in GBS isolates from GBS-colonized pregnant women. | No comparator | One cohort - stratified later into GBS +ve and GBS -ve; did not compare screening strategies |
| **Carreras-Abad (102)** | 2019 | The ultimate aim of this work is to determine the correlate of protection against the major group B streptococcus disease-causing serotypes in infants in the UK. The aim of this feasibility study is to test key operational aspects of the study design. | Not focussed on GBS screening | Focus is not on screening strategies and delivery/timing of IAP - looking at feasibliity of collecting samples ("Interventions: No interventions were performed" Main outcome measures: (1) To test the feasibility of collecting serum at delivery from a large cohort of pregnant women. (2) To test the key operational aspects for a proposed large serocorrelates study. (3) To test the feasibility of collecting samples from those with invasive group B streptococcus.") |
| **Carrillo-Avila (103)** | 2018 | The aim of this study was to compare bacteria isolation by culture and two qPCR techniques, targeting sip and cfb genes, respectively, for detecting colonizing GBS. | No comparator |  |
| **Chan (104)** | 2022 | To compare the incidences of early and late onset neonatal sepsis, including group B streptococcus (GBS) and Escherichia coli (E. Coli) before and after implementation of universal screening on 1 January 2012. | Conference abstract | Full text assessed |
| **Chan (105)** | 2023 | To compare the incidences of early and late-onset neonatal sepsis, including group B streptococcus (GBS) and Escherichia coli (E. coli) before and after implementation of universal screening and intrapartum antibiotics prophylaxis (IAP). | Primary study already identified and included from Overview |  |
| **Chen (106)** | 2023 | We retrospectively analyzed demographic and obstetrical data of pregnant women who have undergone GBS screening and delivered between 2016 and 2018 in Xiamen, China. | LMIC |  |
| **Ching (107)** | 2021 | In this study, we aimed to investigate whether exposure to breast milk is associated with increased risk of LOGBS. | Wrong study design | Focus is on breastfeeding as a risk factor for late onset GBS - case-control but screening programme not clear |
| **Cicalese (108)** | 2020 | We hypothesized that PCR may enhance the detection of neonatal GBS colonization of the GIT, and that the rate will be higher when evaluated with PCR as compared to culture. | No comparator | Focus appears to be on comparing colonisation based on two types of tests not on screening / timing of screening |
| **Constantinou (109)** | 2023 | This study aimed to: (i) determine the acceptability of the different methods of routine testing for Group B Streptococcus (GBS) colonization to pregnant women and Health Care Professionals; (ii) identify the barriers and facilitators to implementation of either routine testing strategy; and (iii) understand how individual and site-level context and process mechanisms influence the acceptability of testing. | Conference abstract | Full text assessed below |
| **Constantinou (110)** | 2024 | To explore women's views on the acceptability of different methods of Group B streptococcal bacteria (GBS) testing in pregnancy, including self-swabbing procedures. | Wrong study design | Discusses harms and benefits  Linked to GBS3 trial |
| **Constantinou (111)** | 2023 | To examine women's knowledge of GBS in pregnancy and their attitudes towards GBS testing. | Wrong study design | Discusses harms and benefits  Linked to GBS3 trial |
| **Constantinou (112)** | 2024 | To determine the acceptability of different methods of routine testing for group B Streptococcus (GBS) colonisation to pregnant women and health care professionals (HCPs), and to examine barriers and facilitators to their implementation. | Wrong study design | Discusses harms and benefits  Linked to GBS3 trial |
| **Constantinou (113)** | 2024 | To conduct a rapid review to synthesise evidence on women and health professionals': (1) knowledge and awareness of; (2) preferences for; and (3) acceptability of GBS screening programmes, and (4) how feasible they are to implement. | Wrong study design | Discusses harms and benefits  Linked to GBS3 trial |
| **Copur (114)** | 2024 | The aim of this study was to investigate the prevalence of Group B Streptococcus (GBS) colonization in pregnancies between 35 and 37 weeks of gestation and to compare the effectiveness of polymerase chain reaction (PCR) method with gold standard technique of culture in antenatal GBS screening. | LMIC; no comparator | Samples were tested using 2 methods from same group of women analyzed using both the culture and PCR method separately) |
| **Costa (115)** | 2023 | The aim of this study was to evaluate if screening Group B Streptococcus colonization by intrapartum polymerase chain reaction could improve intrapartum administration of antibiotic prophylaxis, compared with antepartum culture screening and analyze the sensitivity and specificity of polymerase chain reaction test. | No comparator | "Each woman became her own control and there were no refusal. At time of admission, two rectovaginal swabs were collected: for culture and molecular (PCR) method" |
| **Cynan (116)** | 2024 | The aim was to establish whether a change in practice would be feasible and safe in order to reduce routine use of antibiotics for this group, and to reduce the complications associated with early and prolonged antibiotic use. | Conference abstract |  |
| **d'Otreppe (117)** | 2023 | This study aimed to evaluate the performance and ease of use of the Revogene GBS DS PCR assay for the intrapartum detection of Group B Streptococcus (GBS) colonization, as compared with intrapartum culture and antenatal culture-based screening. | No comparator |  |
| **Daniels (118)** | 2019 | An objective was to identify key process factors that maximise the impact of testing. Two tests are available: antenatal microbiology or intrapartum molecular tests, both requiring a vaginal-rectal swab. | Conference abstract | GBS3 trial ongoing |
| **de Melo (119)** | 2016 | The present study evaluated the antimicrobial susceptibility of GBS isolates from pregnant women who were attended at a public health service in Northern Parana, Brazil. | No comparator |  |
| **Delabaere (120)** | 2017 | This study assesses a new immunoassay, the DIMA test, for identifying GBS-positive patients in the labor ward. | No comparator |  |
| **Desravines (121)** | 2019 | To estimate the prevalence of inadequate intrapartum GBS antibiotic prophylaxis in penicillin-allergic pregnant women and identify potentially modifiable risk factors. | Conference abstract | Full text assessed |
| **Desravines (122)** | 2019 | To estimate the prevalence of and identify modifiable risk factors for alternative antibiotics for group B Streptococcus (GBS) prophylaxis in penicillin-allergic women. | Not focussed on GBS screening | Focus is on alternative antibiotic prophylaxis, not GBS screening |
| **do Nascimento (123)** | 2019 | In the present review, we evaluated published reports addressing the prevalence of GBS in different regions of the country, methods used, and, when available, information regarding antibiotic resistance and serological typing of clinical isolates. | LMIC |  |
| **Doenhardt (124)** | 2020 | The aim of this study is to provide longitudinal epidemiological data on neonatal and young infant sepsis caused by GBS and E. coli to reevaluate existing data and to inform clinical decision-making. | No comparator |  |
| **Duffy (125)** | 2022 | The objective of this study was to describe trends in the use of vancomycin among women undergoing vaginal delivery with group B Streptococcus (GBS) colonization. | No comparator | Focus is on type of antibiotic given |
| **Faby (126)** | 2024 | The aim of this study is to screen for potential bacterial and yeast infections with focus on GBS and Candida infections and its neonatal outcome. | LMIC |  |
| **Fakhraei (127)** | 2024 | We estimate the incidence of early-onset GBS disease (EOD) and late-onset GBS disease (LOD) in Ontario, Canada. | Conference abstract | Full text assessed below |
| **Fakhraei (128)** | 2024 | We estimated infant GBS disease burden in Ontario, Canada and assessed the association of maternal GBS screening (35-37 weeks' gestation) and intrapartum antibiotic prophylaxis (IAP) provision with infant disease rates. | No comparator |  |
| **Farr (129)** | 2020 | This study aimed to evaluate the potential of oral probiotics to eradicate vaginal GBS colonization during the third trimester of pregnancy. | Not focussed on GBS screening |  |
| **Ferreira (130)** | 2018 | In this study, we aimed to compare the performance of conventional polymerase chain reaction and real-time PCR assays as screening methods for S. agalactiae in pregnant women against the microbiological culture method considered as the gold-standard. | LMIC |  |
| **Fowler (131)** | 2020 | We suspect that healthcare costs and length of stay is thereby increased in neonates of mothers who report penicillin allergy. | Conference abstract |  |
| **Fullston (132)** | 2019 | Aiming to improve antimicrobial stewardship and reduce unnecessary maternal and infant exposure to intrapartum antibiotic prophylaxis (IAP), this study assessed the clinical use of a commercially available GBS polymerase chain reaction (PCR) assay for term women with pre-labour rupture of membranes. | No comparator |  |
| **Fung (133)** | 2024 | The study aimed to determine the adherence rate to the universal screening policy a decade after its introduction. Secondly, whether the timing of antibiotics given in GBS carriers reduces the incidence of neonatal sepsis. | No comparator |  |
| **Furfaro (134)** | 2019 | We aimed to assess the comparability of research and diagnostic screening approaches. | No comparator | Focus is on comparing different tests not screening approaches |
| **Furfaro (135)** | 2019 | Previous Australian studies have focused on other regions or included low sample-size representatives; we aimed to describe antenatal GBS colonization in WA | No comparator |  |
| **Gad (136)** | 2024 | This study aims to explore the association between peripartum maternal bacteremia and EONS. | No comparator |  |
| **Galvez (137)** | 2024 | This study aimed to confirm the effect of Ligilactobacillus salivarius V4II-90 on GBS colonisation during pregnancy. | Wrong intervention |  |
| **Gerolymatos (138)** | 2018 | The aim of this study was to assess the prevalence of GBS colonization in pregnant and non-pregnant women and to compare the performance of a polymerase chain reaction (PCR) assay with the established as gold standard technique, culture method, used for the detection of this microorganism. | Wrong comparator | Focus is on comparing two test methods not screening strategies |
| **Go (139)** | 2023 | We aimed to verify the effectivity of these novel diagnostic criteria by comparing antibiotic use and incidence of early-onset bacterial infection between pre- and post-introduction periods. | Wrong intervention | Focus is on reducing antibiotic use not screening |
| **Goh (140)** | 2024 |  | Wrong intervention | Letter to the editor presenting results comparing two tests taken from swabs collected during intrapartum phase so not comparing two screening strategies |
| **Gomi (141)** | 2020 | The present study aimed to investigate the antibiotic susceptibility of GBS by analysis of swab examination results accumulated at our hospital. | Conference abstract |  |
| **Gondim (142)** | 2020 | We aim to determine if the Kaiser neonatal early onset sepsis risk score (KS), which considers gestational age, maternal temperature, duration of membrane rupture, and intrapartum antibiotics in order to estimate the rate of sepsis, can predict which GBS+ placentas will show a placental inflammatory response. | Conference abstract |  |
| **Gopal Rao (143)** | 2019 | To describe the epidemiology of maternal group B streptococcus (GBS) colonisation by racial group. | Wrong study design | This paper is linked to included Gopal Rao 2018 - they have a historical control group |
| **Graham (144)** | 2016 | To determine the prevalence of GBS colonization in a randomly selected group of pregnant women and to devise a screening and management protocol to reduce and possibly prevent early and late-onset GBS disease in our population. | Conference abstract |  |
| **Greenbaum (145)** | 2020 | We aimed to determine whether maternal GBS and the associated intrapartum antibiotic prophylaxis impacts pediatric long-term respiratory infectious morbidity. | Not focussed on GBS screening | Control group are non-GBS+ (so either not screened or screened negative) = not comparing strategies. There is a control group but they seem to have stratified data from one cohort and authors note... 'Therefore, it is plausible that the control group included undiagnosed GBS + gravidas, misclassified in regard to their GBS status'. |
| **Gurudas (146)** | 2022 | We planned to analyze the prevalence of GBS rectovaginal carriage at 36-37 weeks gestation and its effect on early neonatal status. | LMIC |  |
| **Guzman (147)** | 2020 | The purpose of this pilot study was to determine, in a large private practice setting, the proportion of women delivering at term (37 weeks or greater) with an expired GBS negative screen. | Conference abstract |  |
| **Hakim (148)** | 2018 | To determine the applicability of the high-risk group prophylaxis policy for Arab Israeli pregnant women. | Not focussed on GBS screening |  |
| **Hamdan (149)** | 2021 | Therefore, we sought to determine the differences in EOGBS and LOGBS disease by race over the past decade in Tennessee. | No comparator |  |
| **Hanson (150)** | 2022 | The objective of this study was to determine which antibiotics are used in GBS positive patients with documented penicillin allergies and evaluate for antibiotic stewardship improvements at a tertiary hospital in the Midwestern U.S. | Wrong study design |  |
| **Hartvigsen (151)** | 2022 | To investigate the impact of administering Intrapartum Antibiotic Prophylaxis (IAP) to laboring women with one or more risk factors for Early Onset Group B Streptococcal neonatal infection (EOGBS) based on the result of a rapid bedside test for Group B Streptococci (GBS). | No comparator |  |
| **Helmig (152)** | 2019 | The goal of the present study is to report our experience and results from the first half year of GBS testing. | No comparator | Authors report "After introduction of the intrapartum GBS test, we are able to document a 60% reduction in the use of antibiotics in the two groups of women tested" but no comparator |
| **Herrera (153)** | 2023 | To examine the association between unknown maternal Group B Streptococcal (GBS) colonization and the risk of severe neonatal morbidity among individuals undergoing planned caesarean delivery. | No comparator |  |
| **Hillier (154)** | 2019 | A GBS type III capsular polysaccharide (CPS)-tetanus toxoid conjugate (III-TT) vaccine was evaluated for safety and efficacy in preventing acquisition of GBS colonization. | Wrong intervention |  |
| **Hogen Esch (155)** | 2021 | To determine rates and results of maternal Group B streptococcus (GBS) screening during pregnancy and identify sociodemographic characteristics associated with GBS screening in Latin American countries. | Wrong study design; LMIC |  |
| **Hogen Esch (156)** | 2019 | To determine the rates of maternal GBS screening during pregnancy and to identify demographic characteristics associated with maternal GBS screening in Latin America. | Conference abstract | Full text assessed |
| **Horvath-Puho (157)** | 2021 | The aim of this study was to assess long-term mortality, neurodevelopmental impairments (NDIs), and economic outcomes after infant invasive GBS (iGBS) disease up to adolescence in Denmark and the Netherlands. | No comparator | Does not compare strategies but does report long term effects of GBS disease |
| **Hsu (158)** | 2019 | This study aimed to analyze GBS carriage status and genotypic diversity in healthy neonates after implementation of intrapartum antibiotic prophylaxis (IAP) in Taiwan. | No comparator | Does not compare strategies but does report some data for group who did not get antenatal GBS screening |
| **Hung (159)** | 2018 | We examined the risk for Group B streptococcus (GBS)-related diseases in newborns born to mothers who participated in a universal GBS screening program and to determine whether differences are observed in factors affecting the morbidity for neonatal early-onset GBS-related diseases. | Primary study already identified and included from Overview |  |
| **Ishino (160)** | 2022 | This study aimed to determine whether a standardized risk-based strategy of extended antibiotic prophylaxis was associated with a reduced rate of endometritis. | Conference abstract |  |
| **Janczewska (161)** | 2024 | We describe compliance with GBS management and the implementation of IAP in the context of the long-term effect of antibiotics. | No comparator |  |
| **Ji (162)** | 2019 | This study aims to investigate the outcome of a regional anterpartum screening program for EOGBS prevention and to estimate the pros and cons of a new GBS screening strategy employed. | LMIC |  |
| **Johansen (163)** | 2019 | To estimate the prevalence of group B streptococcus at onset of labor and to compare the accuracy of intrapartum antibiotic prophylaxis based on a risk factor strategy versus an intrapartum screening. | No comparator |  |
| **Kabiri (164)** | 2024 | The study aimed to determine whether the performance of membrane stripping, by potentially shortening labor duration, increases the risk of inadequate antibiotic prophylaxis dispensation. | No comparator | Comparing women who have undergone membrane stripping with those who haven't and effectiveness of antibiotics |
| **Kao (165)** | 2019 | The aim of this study was to determine the serotype distribution, antimicrobial resistance, clinical features and molecular characteristics of invasive GBS isolates recovered from Taiwanese infants. | Wrong study design |  |
| **Kasai (166)** | 2024 | Recently, attention has been focused on the efficacy of probiotics during the perinatal period. However, the effect of probiotic intake on the mother-to-child transmission (MTCT) of GBS remains unknown. | Wrong intervention | Addressing effect of probiotics transmission of group B strep between mother and baby |
| **Khalid (167)** | 2022 | We aimed to examine the screening and IAP administration in maternal prior GBS colonisation and the incidence of GBS in this cohort in UHW. | Conference abstract |  |
| **Khalil (168)** | 2019 | The aim of this study was to compare the two strategies, the risk-based approach and the culture-based screening, for identification of vaginal colonization with GBS, using an intrapartum rectovaginal culture as reference standard. | Conference abstract | Full text assessed below |
| **Khalil (169)** | 2019 | To compare a risk-based and culture-based screening approach for identification of group B streptococci (GBS) vaginal colonization using an intrapartum rectovaginal culture as the reference standard. | No comparator | All women self-screening at 35-37 weeks. Results of this compared to the risk factor screening. All screened again prepartum. Does not report on IAP or neonatal outcomes |
| **Khalil (170)** | 2020 | To evaluate whether systematic antepartum screening for Group B Streptococci (GBS) by urine culture improves the risk factor-based selection of pregnant women for intrapartum GBS screening with a rapid polymerase chain reaction (PCR) assay. | No comparator | Specificity of different methods |
| **Khalil (171)** | 2020 | The aim of this study was to assess the performance of routine use of dipstick urine analysis during pregnancy for prediction of recto-vaginal GBS colonisation at the time of labour. | Wrong study design |  |
| **Kirven (172)** | 2021 | Our goal was to examine the relationship between a PcnA label, maternal and neonatal outcomes, and hospital costs. | No comparator |  |
| **Kodama (173)** | 2020 | Chorioamnionitis (CAM) or intrauterine infection has been implicated as a potential cause of perinatal death and cerebral palsy. This regional population-based study assessed its trends over a 20-year period in southern Japan. | Conference abstract |  |
| **Koebnick (174)** | 2021 | For the current study, we tracked body mass index  (BMI) in a large population of children using electronic medical records and examined the association between GBS-IAP  and BMI trajectory during the first 5 years of life. | No comparator | One cohort with same strategy, later stratified to compare those who received IAP with those who did not |
| **Koh (175)** | 2020 | We performed a retrospective audit of women aged 15-49 years who had urine and GBS screens collected during pregnancy (January 2010-June 2019), to determine whether identification of GBS carriage through screening of urines at any time during pregnancy increases the sensitivity of detection of GBS colonisation, compared to screening swabs alone. | Conference abstract |  |
| **Koliwer-Brandl (176)** | 2023 | This study was designed to evaluate applicability of two rapid real-time PCRs in comparison to standard culture identification. | No comparator | Comparing assays for specificity, not strategies |
| **Kovacs (177)** | 2023 | Since selective enrichment media is not avaliable in our laboratory to avoid evergrowing effect of rich vaginal normal flora on GBS we advise clinicians to use parallel sampling (separate vaginal and perianal wipes) instead of usual mixed sampling (when vaginal and perianal areas wiped with the same tampon) process. To confirm this hypothesis, we analysed GBS detection rates in relation of sampling in 2019-2022 period. | Conference abstract |  |
| **Kowal (178)** | 2019 | To evaluate the cost of universal screening for Group B streptococcus (GBS) cultures in pregnancy as compared to the universal treatment of GBS in pregnant women in labor. | No comparator | Comparator is theoretical |
| **Kreicberga (179)** | 2019 | To evaluate the efficacy of universal GBS screening in reducing EOS caused by this pathogen in term neonates. | Conference abstract |  |
| **Kuder (180)** | 2020 | We conducted a retrospective chart review of pregnant patients labeled as penicillin allergic who underwent penicillin skin testing as an outpatient between 2014-2019 at our tertiary academic healthcare institution. | Conference abstract |  |
| **Kugelman (181)** | 2022 | To evaluate whether implementation of GBS fast real-time PCR to all women who require GBS prophylaxis may reduce the use of maternal prophylactic antibiotics. | No comparator |  |
| **Kukovica (182)** | 2025 | The aim of our study was to determine whether self-collection of rectovaginal swabs is a valid alternative to collection by healthcare workers (HCWs). | Wrong study design |  |
| **Lee (183)** | 2021 | We compared the incidence of early-onset GBS sepsis during 2001-2015 between infants born to pregnant women who were screened for GBS colonisation and those born to women who were not screened. | Primary study already identified and included from Overview |  |
| **Lemaire (184)** | 2023 | This study compared the HiberGene loop-mediated isothermal amplification (LAMP) assay to culture, the reference method, for the detection of group B Streptococcus (GBS) in pregnant women. | No comparator |  |
| **Liu (185)** | 2022 | This study aimed to assess the maternal rectovaginal GBS colonization status after IAP, antimicrobial susceptibility and maternal and neonatal outcomes among women administered different antibiotic prophylaxis regimens. | LMIC |  |
| **Liz (186)** | 2019 | The goal of this study was to verify the main outcomes of the GBS screening program in a Portuguese maternity. | Conference abstract |  |
| **Lohrmann (187)** | 2025 | The European DEVANI (Design of a Vaccine Against Neonatal Infections) program assessed the neonatal GBS infection burden in Europe, the clinical characteristics of colonized women and microbiological data of GBS strains in colonized women and their infants with early-onset disease (EOD). | No comparator |  |
| **Lohrmann (188)** | 2023 | This study, a major component of the European DEVANI project (Design of a Vaccine Against Neonatal Infections) describes clinical and important microbiological characteristics of neonatal GBS diseases. It quantifies the rate of antenatal screening and intrapartum antibiotic prophylaxis among cases and identifies risk factors associated with an adverse outcome. | No comparator | Reports adverse outcomes of GBS infection data from 8 EU countries which use different screening strategies but doesn't compare strategies |
| **Low (189)** | 2024 | This study aimed to compare the incidence of group B streptococcus early-onset sepsis among term neonates born to mothers who receive first-line, second-line, or no intrapartum antibiotics and to describe the short-term and survival outcomes of neonates who developed group B streptococcus early-onset sepsis stratified by maternal antepartum prophylaxis. | No comparator |  |
| **Ma (190)** | 2021 | In this study, we present an update on GBS epidemiology in Alberta, Canada, from 2014 to 2020. | No comparator |  |
| **Ma (191)** | 2024 | Analyze Group B Streptococcus (GBS) infection in late-pregnancy pregnant women in Shanghai, the risk factors of GBS infection, and its impact on pregnancy outcomes, providing guidance for early prevention and treatment in clinical practice. | LMIC |  |
| **Maciuleviciute (192)** | 2019 | The aim of the study was to evaluate the efficacy of neonatal early onset GBS disease prevention. | Conference abstract |  |
| **Malik (193)** | 2023 | This systematic review aims to investigate the cost-effectiveness and feasibility of providing universal screening for GBS to all pregnant women and to determine if it should be offered by the NHS. | Wrong study design |  |
| **Manzanares (194)** | 2019 | The aim of the study was to test if maternal obesity and being overweight are independent risk factors for rectovaginal Group B Streptococcus (GBS) colonisation in pregnancy and for early onset GBS disease in the neonate. | No comparator | Focus on maternal obesity |
| **Mazabanda Lopez (195)** | 2022 | We wanted to know if the use of the SRC could be equally efficient in the context of several other infectious risk factors (IRF), in addition to chorioamnionitis, such as intrapartum maternal fever, GBS colonization and/or prolonged rupture of membranes (PROM). | No comparator |  |
| **McCoy (196)** | 2023 | Newer observational data in the era of intrapartum antibiotic prophylaxis suggest a possible reversal of this association; however, it is unclear if this is related to differences in labor management for those with and without Group B Streptococcus colonization. We therefore sought to assess the association between intrapartum antibiotic prophylaxis for Group B Streptococcus colonization and clinical chorioamnionitis within the context of a randomized induction of labor trial with a standardized labor protocol. | Exclusion reason: No comparator; |  |
| **McCoy (197)** | 2023 | Vaginorectal colonization with Group B Streptococcus (GBS) has historically been associated with higher rates of chorioamnionitis and postpartum infection. Data from a recent observational cohort suggests a possible reversal of this association. In order to minimize confounding by differences in labor management, we sought to assess the association between GBS positivity and chorioamnionitis within the context of a randomized induction trial with a standardized labor protocol. | Conference abstract | Full text assessed |
| **McCoy (198)** | 2020 | To compare maternal and cord blood penicillin concentrations in women with and without obesity who are receiving intrapartum group B streptococcus (GBS) prophylaxis. | No comparator | Focus is on maternal obesity |
| **McCoy (199)** | 2025 | In the era of group B Streptococcus (GBS) screening and intrapartum antibiotic prophylaxis (IAP), GBS colonization has been associated with a lower risk of chorioamnionitis, possibly due to a protective effect of IAP. We sought to confirm this finding and assess whether this association varies by gestational week at delivery. | No comparator |  |
| **Meex (200)** | 2022 | Here, we established the performance of the Revogene GBS DS assay for the detection of group B streptococcus on intrapartum vaginal samples in a laboratory environment using a prospective noninterventional study design. | No comparator | Same women screened two tests |
| **Menichini (201)** | 2022 | We wonder whether measures for containment of SARS-CoV-2 transmission would affect Group B streptococcus (GBS) maternal recto-vaginal colonization rates, usually screened at 36-37 weeks' gestation. The primary endpoint of this study was to evaluate the rate of maternal GBS colonization at antenatal screening. | Conference abstract |  |
| **Miselli (202)** | 2023 | To describe epidemiological changes of Group B Streptococcus (GBS) early-onset sepsis (EOD) and late-onset sepsis (LOD) after widespread diffusion of intrapartum antibiotic prophylaxis (IAP). | Conference abstract |  |
| **Moni (203)** | 2020 | Pen skin testing (PST) followed by amoxicillin oral challenge (AOC) have been proven to be safe in pregnancy; the possibility of future sensitization from perinatal Pen administration remains unknown. | Conference abstract |  |
| **Moorhead (204)** | 2019 | To review compliance with, and the effects of education on group B streptococcus screening and intrapartum chemoprophylaxis practices at The Royal Women's Hospital, Melbourne, Australia. | No comparator |  |
| **Morgan (205)** | 2023 | The objective of our study is to determine if human immunodeficiency virus (HIV)-positive pregnant patients have a higher rate of group B streptococcus (GBS) rectovaginal colonization compared with HIV-negative pregnant patients. | No comparator | Focus on HIV as a risk factor |
| **Mukhopadhyay (206)** | 2021 | To determine the difference in rate of weight gain from birth to 5 years based on exposure to maternal group B streptococcal (GBS) intrapartum antibiotic prophylaxis (IAP). | No comparator |  |
| **Nanduri (207)** | 2016 | We analyzed GBS disease trends among young infants from 2006 through 2014 and estimated the potential impact of serotype-specific vaccines. | Conference abstract | Full text assessed |
| **Nanduri (208)** | 2019 | To describe incidence rates, case characteristics, antimicrobial resistance, and serotype distribution of EOD and late-onset disease (LOD; with onset at 7-89 days of life) in the United States from 2006 to 2015 to inform IAP guidelines and vaccine development. | No comparator |  |
| **Nielsen (209)** | 2025 | This cohort study aimed to explore penicillin concentrations in mothers and infants at birth in relation to time elapsed from administration to delivery and to the minimal inhibitory concentration (MIC) for GBS. | Wrong study design |  |
| **O'Sullivan (210)** | 2019 | We aimed to define the burden and clinical features of invasive group B streptococcal disease in infants younger than 90 days in the UK and Ireland, together with the characteristics of disease-causing isolates. | Primary study already identified and included from Overview |  |
| **Pangerl (211)** | 2022 | To investigate Group B Streptococcus (GBS) colonization in pregnancy; adherence to antenatal GBS screening and adherence to the intrapartum antibiotics protocol within two models of care (midwifery and non-midwifery led). | No comparator |  |
| **Peng (212)** | 2022 | To study the effect of intrapartum antibiotic prophylaxis (IAP) of group B streptococcus (GBS) infection on the incidence and bacteriological profile of early-onset neonatal sepsis (EONS). | Full text not in English | Not published in English and conducted in LMIC |
| **Petersen (213)** | 2014 | The present retrospective cohort study determines the trend over time in the rates of GBS and in demographic risk factors for GBS among pregnant women delivering at Rigshospitalet (RH). | Primary study already identified and included from Overview |  |
| **Picchiassi (214)** | 2019 |  | Wrong study design | Editorial commentary |
| **Piffer (215)** | 2022 | The study analyzes the trend of group B streptococcal (GBS) infection in pregnancy in the province of Trento, Italy, where a universal screening of GBS infection in pregnancy has been active for some time. | No comparator |  |
| **Place (216)** | 2021 | A historical cohort study of 1959 women undergoing labor induction by balloon catheter in Helsinki University Hospital, Finland, between January 1, 2014 and December 31, 2017. Women with viable singleton term pregnancy in cephalic presentation, unfavorable cervix (Bishop score <6), and intact amniotic membranes were included. GBS was screened by rapid qualitative in vitro test (XPert R GBS) from vaginal and perineal culture upon admission for labor induction. | No comparator |  |
| **Plainvert (217)** | 2021 | To identify factors associated with vaginal colonization and persistence by group B Streptococcus (GBS) and by the hypervirulent neonatal CC-17 clone in late pregnancy and after delivery, a multicentre prospective observational cohort with 3-month follow-up was established in two university hospitals, Paris area, France. | No comparator |  |
| **Porzio (218)** | 2024 | The study analyzes the adherence of screening and the trend of GBS infection in pregnancy in the province of Caserta, Italy. | No comparator |  |
| **Quinn (219)** | 2021 | The aim of this audit is to compare adequate and inadequate treatment of GBS +ve and PROM mothers and subsequent neonatal outcomes with consideration of the EOS risk calculator. | Conference abstract |  |
| **Roeckner (220)** | 2022 | To (1) assess the impact of antenatal corticosteroids and magnesium sulfate on neonatal outcomes among periviable neonates born from 22w0d-25w6d; and (2) describe the rate of intervention and survival to discharge by year (2013-2020) for fetuses born before 26w0d gestation. | Conference abstract |  |
| **Romagano (221)** | 2019 | The objective is to determine the relationship between maternal antepartum antibiotic administration and antibiotic resistance patterns in preterm neonates admitted to the NICU. | Conference abstract | Full text assessed |
| **Romagano (222)** | 2022 | The objective of this study was to determine the relationship between maternal antepartum antibiotic administration and antibiotic resistance patterns in preterm neonates admitted to the neonatal intensive care unit (NICU). | Not focussed on GBS screening |  |
| **Rosenberg (223)** | 2020 | This study investigated the feasibility of a risk-based screening approach combined with testing of Group B streptococcus (GBS) by polymerase chain reaction (PCR), the effect on use of intrapartum antibiotic prophylaxis (IAP) and the impact on the incidence of early-onset GBS infection (EOGBS). | No comparator |  |
| **Rosenberger (224)** | 2020 | This article examines the role of screening and treatment, evidence-based guidelines, and practice patterns to provide clarification and resolve local practice disparities. | Wrong study design |  |
| **Rowlands (225)** | 2016 |  | Conference abstract |  |
| **Sabroske (226)** | 2023 | We sought to define the frequency of antibiotic resistance over time in a collection of invasive GBS isolates derived from infant early-onset disease (EOD), late-onset disease (LOD), and late-late onset disease (LLOD). | Not focussed on GBS screening |  |
| **Santhanam (227)** | 2018 | To identify the perinatal risk factors for early-onset Group B Streptococcus (EOGBS) sepsis in neonates after inception of a risk-based maternal intrapartum antibiotic prophylaxis strategy in 2004. | LMIC |  |
| **Seedat (228)** | 2017 | This review on universal GBS screening for pregnant women was undertaken to assist NSC policy decision-making. | Conference | Full text assessed |
| **Serra (229)** | 2024 | The purpose of the present study was to evaluate the prevalence of maternal GBS colonization, as well as use of IAP and incidence of episodes of neonatal GBS infection when antibiotic prophylaxis has not been carried out in colonized and/or at risk subjects, in a population of pregnant women during (years 2020-2021) and after (year 2022) the COVID-19 pandemic, also with the aim to establish possible epidemiological and clinical differences in the two subjects' groups. | No comparator |  |
| **Seto (230)** | 2019 | This study compared self-screening by pregnant women with screening by health care workers in a largely Chinese population. | Wrong study design |  |
| **Sgayer (231)** | 2023 | To examine the prevalence and risk factors of extended-spectrum beta-lactamase-producing Enterobacteriaceae (ESBL-E) colonization among women who delivered preterm and at term. | Wrong study design |  |
| **Sharpe (232)** | 2021 | Group B streptococcus (GBS) vaginal/rectal colonization in pregnancy has been associated with early-onset GBS disease (EOGBSD), a leading cause of neonatal morbidity and mortality. In Canada, universal screening for GBS colonization is offered to pregnant people at 35-37 weeks' gestation and those who test positive are offered intrapartum antibiotic prophylaxis (IAP). Universal screening and treatment with IAP have not eradicated all cases of EOGBSD, and IAP has documented side effects. Probiotic supplements have been proposed as a possible way to reduce GBS colonization. | No comparator |  |
| **Shibata (233)** | 2021 | In Japan, universal screening for group B streptococcal (GBS) colonization in pregnant women and intrapartum antibiotic prophylaxis (IAP) are recommended to prevent neonatal GBS infection. However, the dynamics of GBS colonization in Japanese mother/neonate pairs have not been adequately studied. | No comparator |  |
| **Shindler (234)** | 2019 | Therefore, our aim was to investigate molecular patterns of GBS strains from mothers and neonates hospitalized in Maayaney Hayeshua. | Conference abstract |  |
| **Shipitsyna (235)** | 2020 | This study aimed at estimating the distributions of capsular polysaccharide (CPS) types and pilus profiles, and the rates of antimicrobial resistance among GBS strains isolated from colonized pregnant women and newborns in 2010-2011 and 2017-2018 in St. Petersburg, Russia. | Not focussed on GBS screening |  |
| **Shukla (236)** | 2024 | This study investigates the maternal-infant transmission and microbial dynamics of GBS in a tertiary care setting. | LMIC |  |
| **Snoek (237)** | 2022 | The early-onset sepsis calculator (EOSC) reduces unnecessary antibiotic treatment in newborns. However, its performance in identifying cases with early-onset disease (EOD) is unclear. We compared the sensitivity of the EOSC to the current Dutch and National Institute for Health and Care Excellence (NICE) guidelines when applied to a cohort of newborns with culture-positive early-onset sepsis and meningitis. | Not focussed on GBS screening |  |
| **Song (238)** | 2022 | The authors intended to confirm the difference in GBS colonization rate in the conventional culture method, enrichment culture method, and molecular genetic test as screening tests for GBS. | Wrong comparator | Focus is on comparing tests |
| **Subramaniam (239)** | 2019 | To evaluate group B streptococcus (GBS) colonization prevalence and feasibility of intrapartum GBS screening/antibiotic prophylaxis (IAP) in Cameroon, Africa. | LMIC |  |
| **Sumire (240)** | 2021 | The objective of this study is to assess the potential cost-effectiveness of introducing maternal GBS immunisation. | Conference abstract |  |
| **Takahashi (241)** | 2021 | This study aimed to determine the prevalence and risk factors of GBS neonatal umbilical colonization managed by dry cord care in Japan. | Not focussed on GBS screening |  |
| **Tanno (242)** | 2024 | The subculture method using selective enrichment broth significantly improves GBS detection rates in the United States; however, this method is not widely utilized in Japan mainly because of the lack of large-scale validation. Therefore, we aimed to validate the utility of the subculture method in collaboration with multiple facilities. | No comparator |  |
| **Taylor (243)** | 2022 | This study sought to investigate the association between LOGBS and breast milk exposure in infancy. | Wrong study design | Journal club review |
| **Tomlinson (244)** | 2024 | To develop and implement a Group B Streptococcal (GBS) dynamic order set to improve adherence to the American College of Obstetricians and Gynecologists/Centers for Disease Control and Prevention (ACOG/CDC) guidelines. | Not focussed on GBS screening |  |
| **Tran (245)** | 2021 | The objective of the study was to assess antibiotic resistance of GBS in pregnant women at 35-37 weeks of gestation in South Vietnam. | LMIC |  |
| **Ujiie (246)** | 2024 | To compare the prophylactic efficacy of ampicillin and clindamycin against vertical transmission of group B Streptococcus from mothers to their infants by evaluating the rates of group B Streptococcus colonisation. | Not focussed on GBS screening |  |
| **Utekar (247)** | 2022 | The purpose of the study is to find GBS colonization rate for year 2016 and 2017, incidence of EOGBS sepsis in the neonates in the University Maternity Hospital in Limerick (UMHL) and the cost benefit analysis for use of GBS DNA PCR kit. So that only those women who have current GBS would be treated and save all the surveillance tests, antibiotics and costs. | Conference abstract |  |
| **Venkatesh (248)** | 2021 | This study aimed to assess whether colonization with group B streptococcus (GBS) is associated with maternal peripartum infection in an era of routine prophylaxis. | No comparator |  |
| **Virranniemi (249)** | 2019 | The aim of this study was to evaluate the effect of increasing screening-to-labor interval on the performance of group B streptococcus (GBS) screening by late-pregnancy enriched culture compared with intrapartum real-time polymerase chain reaction (RT-PCR) | No comparator | IAP intervention does not change after either AN or IP |
| **Waisman (250)** | 2019 | To quantify effects of different strategies for decreasing neonatal early onset GBS sepsis (EOGBS) in Israel. | Wrong study design | Different screening and treatment scenarios |
| **Walker (251)** | 2019 | Does routine testing of women for group B streptococcus (GBS) colonisation either in late pregnancy or labour to guide the offer of intrapartum antibiotic prophylaxis reduce the occurrence of early onset neonatal sepsis, compared to the current risk factor based strategy? | Conference | GBS3 trial ongoing |
| **Wang (252)** | 2022 | A total of 5,996 pregnant women, who received the Taiwanese universal GBS screening program from 2012 to 2020, were included in this study that investigated GBS colonization, antimicrobial resistance rates and their neonatal incidence of invasive GBS infection. | No comparator |  |
| **Wang (253)** | 2022 | We hypothesize that reported penicillin allergy in GBS-positive mothers is associated with an increased risk of early-onset GBS infection and longer duration of hospitalization for infants with increased interventions. | Conference abstract |  |
| **Wang (254)** | 2023 | We examined the outcomes of infants born to GBS-positive mothers with and without a reported penicillin allergy. | Conference abstract |  |
| **Wang (255)** | 2020 | This retrospective cohort study was undertaken to examine the outcomes of a hybrid risk-and-screen approach to EOGBS prevention using GBS polymerase chain reaction (PCR). | No comparator | Reports relevant outcomes on harms but does not compare strategies |
| **Wang (256)** | 2021 | To assess strategies to reduce EOGBS in China, models were developed to quantify residual EOGBS rates with intrapartum antibiotic prophylaxis in infants >= 35 weeks' gestation in risk factor-based and antepartum screening-based strategies. | LMIC |  |
| **Wang (257)** | 2020 | To examine the efficacy of cervical and lower vaginal secretions in the detection of group B streptococcus (GBS) colonization in women in early and late pregnancy. | LMIC |  |
| **Wilcox (258)** | 2018 | The purpose of the study is to determine the local incidence of chorioamnionitis following the clinical criteria and to test the hypothesis that there is increase antibiotic exposure among neonates who did not fulfill the clinical criteria for chorioamnionitis. | Conference abstract |  |
| **Williams (259)** | 2020 | To evaluate whether group B streptococci (GBS) screening using the 2010 guideline (screening at 35 0/7-37 6/7 weeks of gestation) compared with the 2019 guideline (screening at 36 0/7-37 6/7 weeks of gestation with re-screening of women with GBS-negative results 5 weeks later) was more cost effective. | Wrong study design |  |
| **Yanni (260)** | 2023 | To determine maternal and neonatal risk factors for, and incidence of, neonatal early-onset group B streptococcus (EOGBS) and late-onset (LOGBS) infection in South Australia (SA) and the Northern Territory (NT) | Not focussed on GBS screening |  |
| **Yoshida (261)** | 2025 | This study reviewed maternal and neonatal GBS colonization using polymerase chain reaction, evaluated capsular type distributions, and explored late-onset disease infection routes. | No comparator |  |
| **Zanin (262)** | 2023 | This study aims primarily to estimate the prevalence of maternal GBS positivity and secondarily to evaluate the compliance and the effectiveness of the current GBS prevention protocol. | No comparator |  |
| **Zantow (263)** | 2020 | To evaluate whether Group B Streptococcus (GBS) screening at 35 0/7 to 37 6/7 weeks of gestation versus screening at 36 0/7 to 37 6/7 weeks of gestation with re-screening of GBS negative women five weeks later was most cost-effective. | Conference abstract | Full text assessed - Williams |
| **Zantow (264)** | 2019 | The objective of this study was to evaluate the recommended practice of screening patients admitted preterm for group B streptococcal (GBS) colonization by comparing culture results between those who received antibiotics before culture and those who did not. | Conference abstract |  |
| **Zdjelar (265)** | 2019 |  | Conference abstract |  |
| **Zhang (266)** | 2022 | To investigate the associations between intrapartum antibiotic prophylaxis of group B streptococcus (GBS) in pregnant women and the risk of food allergy in Chinese children | LMIC |  |
| **Zhou (267)** | 2022 | This study aimed to investigate the trend of GBS serotype and genotype change and their correlation with antimicrobial resistance before and after implementation of intrapartum antibiotic prophylaxis (IAP). | Not focussed on GBS screening |  |
| **Zhu (268)** | 2021 | To investigate the incidence of maternal group B Streptococcus (GBS) colonization and neonatal early-onset GBS disease (GBS-EOD), and to study the factors associated with the development of GBS-EOD in the offspring of pregnant women with GBS colonization. | LMIC |  |
| **Zhu (269)** | 2019 | To determine the GBS colonization rate in late pregnancy and neonatal GBS infection in Xiamen, China, and to assess the effectiveness of intrapartum antibiotic prophylaxis (IAP) for the prevention of neonatal GBS-EOD. | LMIC |  |
| **Zietek (270)** | 2020 | The aim of the study is to verify the usefulness of a real-time polymerase chain reaction versus the culture for ante- and intrapartum group B Streptococcus maternal colonization (GBS) and prevalence of discordance during the period between an antepartum screening and delivery. | No comparator | This has a ‘test’ strategy that does not influence IAP |
| **Zoli (271)** | 2023 | This study aimed to assess the awareness of the risks of GBS infection and screening in Jazan Province, Saudi Arabia. | Wrong study design |  |
| **Zonnenberg (272)** | 2019 | This prospective cohort study aims to establish the effect of sepsis after 72 h of life on cognitive, psychomotor, and language development of preterm infants (below 32 weeks gestational age and/or below 1500 g). | Wrong study design |  |

**Abbreviations:** ACOG/CDC: American College of Obstetricians and Gynecologists/Centers for Disease Control and Prevention; AOC: amoxicillin oral challenge; BV: bacterial vaginosis; CAM: chorioamnionitis; CDC: Centers for Disease Control and Prevention; CPS: capsular polysaccharide; d: days; DEVANI: Design of a Vaccine Against Neonatal Infections (study); E. Coli: Escherichia coli; EOD: early-onset disease; EOGBS: early onset group B Streptococcus disease; EOGBSD: early onset group B streptococcus disease; EONS: early-onset neonatal sepsis; EONS: early-onset neonatal sepsis; EOS: early onset sepsis; EOSC: early-onset sepsis calculator; g: grams; GBS: Group B Streptococcus; GI: genital infection; GIT: gastrointestinal tract; HCPs: health care professionals; HCWs: healthcare workers; IAP: intrapartum antibiotic prophylaxis; iCLI-R: inducible clindamycin resistance; iGBS: invasive group B Streptococcus disease; IRF: infectious risk factors; KS: Kaiser neonatal early onset sepsis risk score; LAMP: loop-mediated isothermal amplification; LLOD: late-late onset disease; LOD: late onset disease; LOGBS: late-onset group B Streptococcus disease; MEW: maternal early warning; MIC: minimal inhibitory concentration; MTCT: mother-to-child transmission; NDI: neurodevelopmental impairment; NHS: National Health Service; NICU: neonatal intensive care unit; NSC: National Screening Committee; NT: Northern Territory; PcnA: penicillin allergy; PCR: polymerase chain reaction; PROM: prolonged rupture of membranes; PST: penicillin skin testing; qPCR: quantitative polymerase chain reaction; qSOFA: quick Sequential [Sepsis-related] Organ Failure Assessment; RFs: risk factors; RT-PCR: real-time polymerase chain reaction; SA: South Australia; SARS-CoV-2: severe acute respiratory syndrome coronavirus; SBP: screening-based prophylaxis; SIRS: systemic inflammatory response syndrome; SRC: sepsis risk calculator; STI: sexually transmitted infection; UHW: University Hospital Waterford; VCS: vaginal/cervical classical sampling; VSS: vaginal-self-sampling; w: weeks; WA: Western Australia

### Table 5.3. Table of excluded primary studies from SR and new searches

| **Author**  **(reference)** | **Published Year** | **Aim or objective (verbatim)** | **Reason for Exclusion** | **Additional Notes** |
| --- | --- | --- | --- | --- |
| **Andreu (273)** | 2003 | To analyze the incidence of perinatal sepsis due to group B streptococcus (GBS) as related to compliance with recommendations for its prevention issued by the Catalan Societies for Obstetrics, for Pediatrics, and for Infectious Diseases and Clinical Microbiology in 1997. | Not published in English | Panneflek 2024 |
| **Berardi (274)** | 2019 | We compared cases of EOS (at or above 35 weeks' gestation) registered in 2003-2009 (baseline period: 266,646 LBs) and in 2010-2016, after introduction of a new strategy (serial physical examinations, SPEs) for managing asymptomatic neonates at risk for EOS (intervention period: 265,508 LBs). | Neonatal management, not focused on screening strategies | New study from updated search |
| **Berardi (275)** | 2023 | Italian prospective surveillance cohort data (2003-2022) were used to study the type and duration of IAP according to the timing of symptoms onset of group B streptococcus (GBS) and E. coli culture-confirmed EOS cases. | Neonatal management, not focused on screening strategies | New study from updated search |
| **Chen (276)** | 2019 | We examined trends in EOGBSD rates over time in Queensland in the setting of these guidelines and whether management of cases reflected the recommendations. | Awaiting classification | Insufficient details to be confident there are two strategies  New study from updated search |
| **Eason (277)** | 2021 | The aim of our study was to assess the safety of implementing the Kaiser Permanente Early Onset Sepsis (KPEOS) calculator to minimise antibiotic usage in term infants in line with antimicrobial stewardship, reducing separation from mother at birth and facilitating earlier discharge. | Neonatal management, not focused on screening strategies | New study from updated search |
| **Finale (278)** | 2022 | The aim of this study was therefore to analyze the effectiveness of these recommendations, in terms of the expected increase in the percentage of women tested during pregnancy and of positive culture results, and the expected decrease in variability across the birth centers of the region. | Awaiting classification | Insufficient details to be confident there are two strategies - seems focussed on lab techniques for culturing  New study from updated search |
| **Freitas (279)** | 2017 | To describe early-onset neonatal sepsis (EOS) epidemiology in a public maternity hospital in Brasilia, Brazil. | LMIC | Panneflek 2024 |
| **Gilbert (280)** | 2003 | The aim of this study was to compare two IAP protocols, in a community hospital (A) and a university teaching hospital (B), to assess compliance and factors affecting it. | Awaiting classification | Insufficient details to be confident there are two strategies  Excluded from Panneflek 2024 |
| **Jalil (281)** | 2019 | To evaluate random screening of pregnant women for GBS infection, the rate of neonatal GBS infection and neonatal admission | Awaiting classification | Insufficient details to be confident there are two strategies  Excluded from Panneflek 2024 |
| **Jourdan-da Silva (282)** | 2008 |  | Not published in English | Panneflek 2024 |
| **Sagrera (283)** | 2001 |  | Not published in English | Panneflek 2024 |
| **ScheftelowitzCohen (284)** | 2021 | Our aim was to re-evaluate the current screening policy for Group B Streptococcus (GBS), considering colonization and prevalence rates and costs estimates | Awaiting classification | Insufficient details to be confident there are two strategies |
| **Simetka (285)** | 2010 | To evaluate the effectiveness of introduction of national guideline to prevent early-onset group B streptococcal (EOGBS) disease, number of screened women and incidence of GBS colonization. | Not published in English | Panneflek 2024 |
| **Sorg (286)** | 2021 | Aim of the study was to identify temporal trends in incidence of EOS and their association to GBS Screening. | Not published in English | Panneflek 2024 |
| **Sridhar (287)** | 2014 | This retrospective study was done to determine the incidence of neonatal Group B streptococcal sepsis among newborn between 1998 and 2010. | LMIC | Panneflek 2024 |
| **Tapia (288)** | 2007 | The aim of this study was to compare incidence, bacteriology and associated mortality of neonatal sepsis in a neonatal unit, after (2001-2004) and before (1995-1996) implementation of universal screening for prevention of Group B Streptococcus diseases. | Not published in English | Panneflek 2024 |
| **Van Rossem (289)** | 2023 | We aim to assess if this guideline performs better at reducing the rate of antibiotic treatment for EOS than the old Dutch categorical EOS guideline, which focused primarily on group B streptococcus (GBS) testing and prophylaxis. | Neonatal management, not focused on screening strategies | New study from updated search |
| **Yucesoy (290)** | 2004 | This study was conducted to find out the group B streptococcus colonisation of pregnant women in Kocaeli, Turkey. | LMIC | Hasperhoven 2020 |

**ABREVIATIONS:** EOGBS: early-onset group B streptococcal disease; EOS: early onset sepsis; GBS: Group B streptococcus; IAP: intrapartum antibiotic prophylaxis; KPEOS: Kaiser Permanente Early Onset Sepsis; LBs: live births; LMIC: Low or middle income country; SPEs: serial physical examinations

### References

1. Abde M, Weis N, Kjaerbye-Thygesen A, Moseholm E. Association between asymptomatic bacteriuria in pregnancy and adverse pregnancy- and births outcomes. A systematic review. European Journal of Obstetrics, Gynecology, & Reproductive Biology. 2024;302:116-24.

2. Adejumo RN, Nakakana UN. Current practice of intrapartum antibiotic prevention in Nigeria and sub saharan Africa. Transactions of the Royal Society of Tropical Medicine and Hygiene. 2019;113(Supplement 1):S232-S3.

3. Allen VM, Yudin MH. No. 276-Management of Group B Streptococcal bacteriuria in pregnancy. Journal of Obstetrics & Gynaecology Canada: JOGC. 2018;40(2):e181-e6.

4. Alotaibi NM, Alroqi S, Alharbi A, Almutiri B, Alshehry M, Almutairi R, et al. Clinical characteristics and treatment strategies for Group B Streptococcus (GBS) infectpon in Pediatrics: A systematic review. Medicina. 2023;59(7):1279.

5. Alshengeti A. Group B Streptococcus among pregnant women and neonates in Saudi Arabia: A systemic review. Pathogens. 2022;11(9):(no pagination).

6. Angelescu K, Nussbaumer-Streit B, Sieben W, Scheibler F, Gartlehner G. Benefits and harms of screening for and treatment of asymptomatic bacteriuria in pregnancy: a systematic review. BMC Pregnancy & Childbirth. 2016;16(1):336.

7. Ashary N, Singh A, Chhabria K, Modi D. Meta-analysis on prevalence of vaginal group B streptococcus colonization and preterm births in India. Journal of Maternal-Fetal & Neonatal Medicine. 2022;35(15):2923-31.

8. Biondi EA, Lee B, Ralston SL, Winikor JM, Lynn JF, Dixon A, et al. Prevalence of bacteremia and bacterial meningitis in febrile neonates and infants in the second month of life: A systematic review and meta-analysis. JAMA Network Open. 2019;2(3):e190874.

9. Borg SA, Cao J, Nguyen PY, Aziz S, Vogel JP. Self-collection of samples for group B streptococcus testing during pregnancy: a systematic review and meta-analysis. BMC Medicine. 2023;21(1):498.

10. Bouchghoul H. Term prelabor rupture of membranes: CNGOF Guidelines for clinical practice - initial management. Gynecologie Obstetrique Fertilite et Senologie. 2020;48(1):24-34.

11. Braye K, Ferguson J, Davis D, Catling C, Monk A, Foureur M. Effectiveness of intrapartum antibiotic prophylaxis for early-onset group B Streptococcal infection: An integrative review. Women and Birth. 2018;31(4):244-53.

12. Chelkeba L, Fanta K, Mulugeta T, Melaku T. Bacterial profile and antimicrobial resistance patterns of common bacteria among pregnant women with bacteriuria in Ethiopia: a systematic review and meta-analysis. Archives of Gynecology & Obstetrics. 2022;306(3):663-86.

13. Constantinou G, Webb R, Ayers S, Mitchell EJ, Daniels J. Acceptability and feasibility of maternal screening for Group B Streptococcus: a rapid review. medRxiv. 2024;28.

14. da Silva HD, Kretli Winkelströter L. Universal gestational screening for Streptococcus agalactiae colonization and neonatal infection — A systematic review and meta-analysis. Journal of Infection and Public Health. 2019;12(4):479-81.

15. de Souza HD, Diorio GRM, Peres SV, Francisco RPV, Galletta MAK. Bacterial profile and prevalence of urinary tract infections in pregnant women in Latin America: a systematic review and meta-analysis. BMC Pregnancy & Childbirth. 2023;23(1):774.

16. Dong Y, Basmaci R, Titomanlio L, Sun B, Mercier JC. Neonatal sepsis: within and beyond China. Chinese Medical Journal. 2020;133(18):2219-28.

17. Emaneini M, Jabalameli F, van Leeuwen WB, Beigverdi R. Prevalence of Group B Streptococcus in pregnant women in Iran: A systematic review and meta-analysis. Pediatric Infectious Disease Journal. 2018;37(2):186-90.

18. Feuerschuette OHM, Silveira SK, Cancelier ACL, da Silva RM, Trevisol DJ, Pereira JR. Diagnostic yield of real-time polymerase chain reaction in the diagnosis of intrapartum maternal rectovaginal colonization by group B Streptococcus: a systematic review with meta-analysis. Diagnostic Microbiology & Infectious Disease. 2018;91(2):99-104.

19. Fitzpatrick D, Holmes NE, Hui L. A systematic review of maternal TORCH serology as a screen for suspected fetal infection. Obstetrical and Gynecological Survey. 2022;77(7):398-9.

20. Ghia C, Rambhad G. Disease burden due to Group B Streptococcus in the Indian population and the need for a vaccine - a narrative review. Therapeutic Advances in Infectious Disease. 2021;8:no pagination.

21. Gizachew M, Tiruneh M, Moges F, Tessema B. Streptococcus agalactiae maternal colonization, antibiotic resistance and serotype profiles in Africa: a meta-analysis. Annals of Clinical Microbiology & Antimicrobials. 2019;18(1):14.

22. Glaser MA, Hughes LM, Jnah A, Newberry D. Neonatal sepsis: A review of pathophysiology and current management strategies. Advances in Neonatal Care. 2021;21(1):49-60.

23. Han MY, Xie C, Huang QQ, Wu QH, Deng QY, Xie TA, et al. Evaluation of Xpert GBS assay and Xpert GBS LB assay for detection of Streptococcus agalactiae. Annals of Clinical Microbiology & Antimicrobials. 2021;20(1):62.

24. Hanson L, VandeVusse L, Malloy E, Garnier-Villarreal M, Watson L, Fial A, et al. Probiotic interventions to reduce antepartum Group B streptococcus colonization: A systematic review and meta-analysis. Midwifery. 2022;105:103208.

25. Hasperhoven G, Al-Nasiry S, Bekker V, Villamor E, Kramer B. Authors' reply re: Universal screening versus risk-based protocols for antibiotic prophylaxis during childbirth to prevent early-onset Group B streptococcal disease: a systematic review and meta-analysis. BJOG: An International Journal of Obstetrics & Gynaecology. 2020;127(8):1039-40.

26. Hasperhoven G, Al-Nasiry S, Bekker V, Villamor E, Kramer B. Authors' reply re: Universal screening versus risk-based protocols for antibiotic prophylaxis during childbirth to prevent early-onset group B streptococcal disease: a systematic review and meta-analysis. BJOG: An International Journal of Obstetrics & Gynaecology. 2020;127(9):1168-9.

27. Huang J, Lin XZ, Zhu Y, Chen C. Epidemiology of group B streptococcal infection in pregnant women and diseased infants in mainland China. Pediatrics & Neonatology. 2019;60(5):487-95.

28. Hueth KD, Thompson-Leduc P, Totev TI, Milbers K, Timbrook TT, Kirson N, et al. Assessment of the impact of a meningitis/encephalitis panel on hospital length of stay: A systematic review and meta-analysis. Antibiotics. 2022;11(8):(no pagination).

29. Iadeluca L, Farrington E, McLean T, Rousseau B, Agosti Y, Absalon J, et al. Maternal screening and treatment for group B streptococcus (GBS) are associated with non-adherence to guidelines, false-negative results and high management costs in the United Kingdom, Italy, France, Spain and Germany. Value in Health. 2017;20(9):A797-A8.

30. Impens E, Cools P, Impens A, Mulinganya G, Callens S. Etiology of neonatal sepsis in sub-Saharan Africa: Pathogens and their antibiotic susceptibility patterns. American Journal of Tropical Medicine and Hygiene. 2019;101(5 Supplement):145.

31. Jefferies AL. Management of term infants at increased risk for early-onset bacterial sepsis. Paediatrics and Child Health (Canada). 2017;22(4):223-8.

32. Jury I, Thompson K, Hirst JE. A scoping review of maternal antibiotic prophylaxis in low- and middle-income countries: Comparison to WHO recommendations for prevention and treatment of maternal peripartum infection. International Journal of Gynaecology & Obstetrics. 2021;155(3):319-30.

33. Kahwati LC, Clark R, Berkman N, Urrutia R, Patel SV, Zeng J, et al. Screening for bacterial vaginosis in pregnant adolescents and women to prevent preterm delivery: updated evidence report and systematic review for the US Preventive Services Task Force. JAMA. 2020;323(13):1293-309.

34. Karampatsas K, Davies H, Mynarek M, Andrews N, Heath PT, Le Doare K. Clinical risk factors associated with late-onset invasive Group B Streptococcal disease: Systematic review and meta-analyses. Clinical Infectious Diseases. 2022;75(7):1255-64.

35. Khademi F, Sahebkar A. Group B streptococcus drug resistance in pregnant women in Iran: a meta-analysis. Taiwanese Journal of Obstetrics & Gynecology. 2020;59(5):635-42.

36. Koucky M, Kamel R, Vistejnova L, Kalis V, Ismail KM. A global perspective on management of bacterial infections in pregnancy: a systematic review of international guidelines. Journal of Maternal-Fetal & Neonatal Medicine. 2022;35(19):3751-60.

37. Kwatra G, Cunnington MC, Merrall E, Adrian PV, Ip M, Klugman KP, et al. Prevalence of maternal colonisation with group B streptococcus: a systematic review and meta-analysis. The Lancet Infectious Diseases. 2016;16(9):1076-84.

38. Lamont RF, Jorgensen JS, Vinter CA. Re: Universal screening versus risk-based protocols for antibiotic prophylaxis during childbirth to prevent early-onset group B streptococcal disease: a systematic review and meta-analysis. BJOG: An International Journal of Obstetrics & Gynaecology. 2020;127(9):1167-8.

39. Le Doare K, Heath PT, Plumb J, Owen NA, Brocklehurst P, Chappell LC. Uncertainties in screening and prevention of Group B Streptococcus disease. Clinical Infectious Diseases. 2019;69(4):720-5.

40. Le Doare K, O'Driscoll M, Turner K, Seedat F, Russell NJ, Seale AC, et al. Intrapartum antibiotic chemoprophylaxis policies for the prevention of Group B Streptococcal disease worldwide: Systematic review. Clinical Infectious Diseases. 2017;65(suppl_2):S143-S51.

41. Lemma D, Huluka TK, Chelkeba L. Prevalence and antimicrobial susceptibility of group B streptococci among pregnant women in Ethiopia: A systemic review and meta-analysis study. SAGE Open Medicine. 2022;10:no pagination.

42. Ludmir J. Household or institutional birth, where and by whom-screening GBS. International Journal of Gynecology and Obstetrics. 2018;143(Supplement 3):78.

43. Malik M, Nozad B, Majeed A. Should the NHS offer universal group B Streptococcus screening to pregnant women? Therapeutic Advances in Infectious Disease. 2023;Conference: Group B Strep in Pregnancy and Babies Conference. Virtual. 10:3-4.

44. Mercado-Evans V, Zulk JJ, Hameed ZA, Patras KA. Gestational diabetes as a risk factor for GBS maternal rectovaginal colonization: a systematic review and meta-analysis. BMC Pregnancy & Childbirth. 2024;24(1):488.

45. Money D, Allen VM. No. 298-The prevention of early-onset neonatal Group B Streptococcal disease. Journal of Obstetrics & Gynaecology Canada: JOGC. 2018;40(8):e665-e74.

46. Money D, Allen VM. The prevention of early-onset neonatal Group B Streptococcal disease. Journal of Obstetrics & Gynaecology Canada: JOGC. 2016;38(12S):S326-S35.

47. Moradi M, Grieger J, Teong XT, Heilbronn L. Associations between intrapartum antibiotic prophylaxis and childhood autoimmune diseases and obesity: A systematic review and meta-analysis of observational studies. Obesity Research and Clinical Practice. 2024;18(5 Supplement 1):S63.

48. Nadeau H, Bisson C, Chen X, Zhao D, Williams M, Edwards R. Vaginal-perianal or vaginal-perineal compared with vaginal-rectal culture-based screening for Group B Streptococci (GBS) colonization during the third trimester of pregnancy: A systematic review and meta-analysis. American Journal of Obstetrics and Gynecology. 2022;226(2):318.

49. Nanduri S, Petit S, Baumbach J, Reingold A, Miller L, Harrison L, et al. Trends in group B streptococcal infections among infants <3 months of age and the potential impact of a maternal vaccine in the United States (2006-2014). Open Forum Infectious Diseases Conference: ID Week. 2016;3(Supplement 1).

50. Odubamowo K, Garcia M, Muriithi F, Ogollah R, Daniels JP, Walker KF. Self-collected versus health-care professional taken swab for identification of vaginal-rectal colonisation with group B streptococcus in late pregnancy: a systematic review. European Journal of Obstetrics, Gynecology, & Reproductive Biology. 2023;286:95-101.

51. Pangerl S, Sundin D, Geraghty S. Group B Streptococcus screening guidelines in pregnancy: A critical review of compliance. Maternal & Child Health Journal. 2021;25(2):257-67.

52. Paul P, Goncalves BP, Le Doare K, Lawn JE. 20 million pregnant women with group B streptococcus carriage: consequences, challenges, and opportunities for prevention. Current Opinion in Pediatrics. 2023;35(2):223-30.

53. Peng J, Liu Y, Zou J, Wang J, Jorge Luis CD, Zhong H. Accuracy of real-time polymerase chain reaction test for Group B Streptococcus detection in pregnant women: A systematic review and meta-analysis. European Journal of Obstetrics, Gynecology, & Reproductive Biology. 2025;304:141-51.

54. Ren J, Li Y, Qiang Z, Wang Y. Effect of intrapartum antibiotic prophylaxis on maternal group b streptococcus-related neonatal colonization and infection: A meta-analysis. Latin American Journal of Pharmacy. 2021;40(6):1319-26.

55. Rezaei SJ, Mateen FJ. Encephalitis and meningitis in Western Africa: a scoping review of pathogens. Tropical Medicine and International Health. 2021;26(4):388-96.

56. Rogozinska E, Formina S, Zamora J, Mignini L, Khan KS. Accuracy of onsite tests to detect asymptomatic bacteriuria in pregnancy: A systematic review and eta-analysis. Obstetrics & Gynecology. 2016;128(3):495-503.

57. Ronzoni S, Boucoiran I, Yudin MH, Coolen J, Pylypjuk C, Melamed N, et al. Guideline No. 430: Diagnosis and management of preterm prelabour rupture of membranes. Journal of Obstetrics and Gynaecology Canada. 2022;44(11):1193-208.e1.

58. Sadeh M, Salehi-Abargouei A, Azartoos N, Mirzaei F, Khalili MB. Distribution of streptococcus agalactiae among Iranian women from 1992 to 2018: A systematic review and meta-analysis. Jundishapur Journal of Microbiology. 2020;13(7):1-11.

59. Seedat F, Stinton C, Patterson J, Geppert J, Tan B, Robinson ER, et al. Adverse events in women and children who have received intrapartum antibiotic prophylaxis treatment: a systematic review. BMC Pregnancy and Childbirth. 2017;17(1):247.

60. Seedat F, Brown CS, Stinton C, Patterson J, Geppert J, Freeman K, et al. Bacterial load and molecular markers associated with early-onset Group B Streptococcus: A systematic review amnd Meta-analysis. Pediatric Infectious Disease Journal. 2018;37(12):e306-e14.

61. Seedat F, Cooper JA, Uthman OA, Takwoingi Y, Robinson ER, Kandala NB, et al. Real-time polymerase chain reaction tests versus antenatal culture tests for the screening of maternal group B Streptococcus colonisation in labour. Cochrane Database of Systematic Reviews. 2018;2018(5):(no pagination).

62. Seedat F, Geppert J, Stinton C, Patterson J, Brown CS, Tan B, et al. Universal antenatal culture-based screening for maternal Group B Streptococcus (Gbs) carriage to prevent early-onset Gbs Disease: A systematic review for the UK National Screening Committee (Nsc). Journal of Epidemiology and Community Health. 2017;71(Supplement 1):A18.

63. Senat MV, Schmitz T, Bouchghoul H, Diguisto C, Girault A, Paysant S, et al. Term prelabor rupture of membranes: CNGOF Guidelines for clinical practice - short text. Gynecologie Obstetrique Fertilite et Senologie. 2020;48(1):15-8.

64. Tansarli GS, Chapin KC. Diagnostic test accuracy of the BioFire R FilmArray R meningitis/encephalitis panel: a systematic review and meta-analysis. Clinical Microbiology & Infection. 2020;26(3):281-90.

65. Turrentine MA, Colicchia LC, Hirsch E, Cheng PJ, Tam T, Ramsey PS, et al. Efficiency of screening for the recurrence of antenatal Group B Streptococcus colonization in a subsequent pregnancy: A systematic review and meta-analysis with independent patient data. American Journal of Perinatology. 2016;33(5):510-7.

66. Walker KF, Morris E, Plumb J, Gray J, Thornton JG, Daniels J. Universal testing for group B streptococcus during pregnancy: need for a randomised trial. BJOG: An International Journal of Obstetrics and Gynaecology. 2020;127(6):693.

67. Walker KF, Plumb J, Gray J, Thornton JG, Avery AJ, Daniels JP. Should all pregnant women be offered testing for group B streptococcus? The BMJ. 2021;373:(no pagination).

68. Wingert A, Pillay J, Sebastianski M, Gates M, Featherstone R, Shave K, et al. Asymptomatic bacteriuria in pregnancy: systematic reviews of screening and treatment effectiveness and patient preferences. BMJ Open. 2019;9(3):e021347.

69. Wu R, Cui X, Pan R, Li N, Zhang Y, Shu J, et al. Pathogenic characterization and drug resistance of neonatal sepsis in China: a systematic review and meta-analysis. European Journal of Clinical Microbiology & Infectious Diseases. 2025.

70. Yektakooshali MH, Hamidi M, Tousi SMTR, Nikokar I. Prevalence of group B streptococcus colonization in iranian pregnant women: A systematic review and meta-analysis. International Journal of Reproductive BioMedicine. 2018;16(12):731-44.

71. Yudin MH, Money DM. No. 211-Screening and management of bacterial vaginosis in pregnancy. Journal of Obstetrics & Gynaecology Canada: JOGC. 2017;39(8):e184-e91.

72. Yudin MH, van Schalkwyk J, Van Eyk N. No. 233-Antibiotic therapy in preterm premature rupture of the membranes. Journal of Obstetrics and Gynaecology Canada. 2017;39:e207-e12.

73. Abrok M, Tigyi P, Kostrzewa M, Burian K, Deak J. Evaluation of the results of group b streptococcus screening by MALDI-TOF MS among pregnant women in a Hungarian hospital. Pathogens. 2020;9(1):(no pagination).

74. Achten NB, Dorigo-Zetsma JW, van Rossum AMC, Oostenbrink R, Plotz FB. Risk-based maternal group B Streptococcus screening strategy is compatible with the implementation of neonatal early-onset sepsis calculator. Clinical and Experimental Pediatrics. 2020;63(10):406-10.

75. Al Luhidan L, Madani A, Albanyan EA, Al Saif S, Nasef M, AlJohani S, et al. Neonatal Group B Streptococcal infection in a tertiary care hospital in Saudi Arabia: A 13-year experience. Pediatric Infectious Disease Journal. 2019;38(7):731-4.

76. Alaaraj B, Irshaid A, Al-Lawama M. Decreasing the rate of antibiotics administration to newborns of Mmothers with prolonged rupture of membranes and unknown Group B Streptococcus status using the Plan-Do-Study-Act Quality Improvement model. Infectious Diseases in Clinical Practice. 2022;30(5):(no pagination).

77. Ali M, Alamin M, Alzubaidi K, Ali B, Ismail AL, Abass G, et al. Epidemiology, microbiological characteristics and clinical outcomes of invasive blood stream infections of group b streptococcal isolates from qatar. Journal of Emergency Medicine, Trauma and Acute Care Conference: Qatar Health. 2022(pagination).

78. Alshengeti A, Alharbi A, Alraddadi S, Alawfi A, Aljohani B. Knowledge, attitude and current practices of pregnant women towards group B streptococcus screening: cross-sectional study, Al-Madinah, Saudi Arabia. BMJ Open. 2020;10(2):e032487.

79. Andreasen T, Kjolseth Moller J, Rohi Khalil M. Comparison of BD MAX GBS and GenomEra GBS assays for rapid intrapartum PCR detection of vaginal carriage of group B streptococci. PLoS ONE. 2019;14(4):e0215314.

80. Andrikopoulou M, Huang Y, Duffy CR, Stern-Ascher CN, Wright JD, Goffman D, et al. Antibiotic use without indication during delivery hospitalizations in the United States. Obstetrics & Gynecology. 2019;134(4):718-25.

81. Antonello VS, Dalle J, Dall'Oglio E, Ramos S, Bassols F, Jimenez MF. Alternative antimicrobials for prophylaxis of the Group B Streptococcus maternal-fetal disease. Journal of Infection in Developing Countries. 2020;14(6):664-8.

82. Assabahi A, Driggers R, Keeys CA, Mader A, Norris P. Evaluation of appropriate vancomycin prescribing for the prevention of newborn group B streptococcal infections in a community hospital obstetrics service. Journal of Perinatal Medicine. 2022;50(9):1218-24.

83. Aziz N, Spiegel A, Bentley J, Yoffe P, Klikoff A, Ehrlich K, et al. Evaluation of probiotic oral supplementation effects on group B streptococcus rectovaginal colonization in pregnant women: a randomized double-blind placebo-controlled trial. American Journal of Obstetrics and Gynecology. 2018;219(6):638.

84. Baldwin C, Dhariwal S, Ventilacion L, Zill EHR, Mavrogiorgou E, Prentice S. Investigation of a local increase in rates of invasive group B streptococcal infection in infants; was COVID-19 to blame? Archives of Disease in Childhood. 2021;106:A338-A9.

85. Bassaw B, Romeo-Bassey R, Jaggat A, Manjunath M, Perkins S, Khan S. Screening for group B hemolytic streptococcal infection in pregnancy in a low-resourced country. Clinical and Experimental Obstetrics and Gynecology. 2019;46(1):45-9.

86. Bauer ME, Housey M, Bauer ST, Behrmann S, Chau A, Clancy C, et al. Risk Factors, etiologies, and screening tools for sepsis in pregnant women: A multicenter case-control study. Anesthesia & Analgesia. 2019;129(6):1613-20.

87. Belhadi K, Chayeb FZ, Djaara H. The first Prenatal Group B Streptococcus (GBS) Screening in Late Pregnancy in Algerian population (Northeast Algeria). Acta Microbiologica Hellenica. 2020;65(3):171-9.

88. Berardi A, Rossi C, Spada C, Vellani G, Guidotti I, Lanzoni A, et al. Strategies for preventing early-onset sepsis and for managing neonates at-risk: wide variability across six Western countries. Journal of Maternal-Fetal & Neonatal Medicine. 2019;32(18):3102-8.

89. Berardi A, Spada C, Creti R, Ambretti S, Chiarabini R, Barozzi A, et al. Risk factors for group B streptococcus early-onset disease: an Italian, area-based, case-control study. Journal of Maternal-Fetal & Neonatal Medicine. 2020;33(14):2480-6.

90. Berardi A, Spada C, Creti R, Auriti C, Gambini L, Rizzo V, et al. Maternal carriage in late-onset group b streptococcus disease, Italy. Emerging Infectious Diseases. 2021;27(9):2279-87.

91. Berardi A, Tzialla C, Travan L, Bua J, Santori D, Azzalli M, et al. Secondary prevention of early-onset sepsis: a less invasive Italian approach for managing neonates at risk. Italian Journal of Pediatrics. 2018;44(1):73.

92. Bjorklund V, Saxen H, Hertting O, Malchau Carlsen EL, Hoffmann S, Hakansson S, et al. Early-onset group B streptococcal infections in five Nordic countries with different prevention policies, 1995 to 2019. Euro Surveillance: Bulletin Europeen sur les Maladies Transmissibles = European Communicable Disease Bulletin. 2024;29(3).

93. Blanquart AL, Garnier F, Lauvray T, Mazeau PC, Martinez S, Catalan C, et al. Vaginal screening for group B streptococcus using PCR in pregnant women with unknown colonization status: Impact on newborn monitoring for early-onset sepsis. Archives de Pediatrie. 2024;31(7):461-6.

94. Bogiel T, Ziolkowski S, Domian A, Dobrzynska Z. An application of Real-Time PCR and CDC Protocol may significantly reduce the incidence of Streptococcus agalactiae infections among neonates. Pathogens. 2022;11(9):(no pagination).

95. Bonney DO, Morris SA. Perinatal antibiotics in a tertiary Australian hospital: A point prevalence study. Journal of Paediatrics and Child Health. 2019;55(Supplement 1):9.

96. Bramugy J, Mucasse H, Massora S, Vitorino P, Aerts C, Mandomando I, et al. Short- and long-term outcomes of Group B Streptococcus invasive disease in Mozambican children: Results of a matched cohort and retrospective observational study and implications for future vaccine introduction. Clinical Infectious Diseases. 2022;74(Suppl_1):S14-S23.

97. Braye K, Ferguson J, Ball J, Foureur M. Intrapartum antibiotic prophylaxis for women who are screened positive for group B streptococcal colonisation: Clinical compliance with the guideline. Australian & New Zealand Journal of Obstetrics & Gynaecology. 2021;61(6):870-5.

98. Braye K, Foureur M, de Waal K, Jones M, Putt E, Ferguson J. Group B streptococcal screening, intrapartum antibiotic prophylaxis, and neonatal early-onset infection rates in an Australian local health district: 2006-2016. PLoS ONE. 2019;14(4):e0214295.

99. Brigtsen AK, Jacobsen AF, Dedi L, Melby KK, Espeland CN, Fugelseth D, et al. Group B Streptococcus colonization at delivery is associated with maternal peripartum infection. PLoS ONE. 2022;17(4):e0264309.

100. Camus C, Penaranda G, Khiri H, Camiade S, Molet L, Lebsir M, et al. Acceptability and efficacy of vaginal self-sampling for genital infection and bacterial vaginosis: A cross-sectional study. PLoS ONE. 2021;16(11):e0260021.

101. Capraro GA, Lala S, Khaled K, Gosciniak E, Saadat B, Alvarez SM, et al. Association of sexually-transmitted infection and African-American race with Streptococcus agalactiae colonization in pregnancy. Antimicrobial Resistance & Infection Control. 2020;9(1):174.

102. Carreras-Abad C, Cochet M, Hall T, Ramkhelawon L, Khalil A, Peregrine E, et al. Developing a serocorrelate of protection against invasive group B streptococcus disease in pregnant women: a feasibility study. Health Technology Assessment (Winchester, England). 2019;23(67):1-40.

103. Carrillo-Avila JA, Gutierrez-Fernandez J, Gonzalez-Espin AI, Garcia-Trivino E, Gimenez-Lirola LG. Comparison of qPCR and culture methods for group B Streptococcus colonization detection in pregnant women: evaluation of a new qPCR assay. BMC Infectious Diseases. 2018;18(1):305.

104. Chan YTV, Hui SYA, Ma T, Mok SL, Chan D, Kong M, et al. Incidence of group B streptococcal neonatal infection after universal screening. BJOG: An International Journal of Obstetrics and Gynaecology. 2022;129(Supplement 1):83.

105. Chan YTV, Lau SYF, Hui SYA, Ma T, Kong CW, Kwong LT, et al. Incidence of neonatal sepsis after universal antenatal culture-based screening of group B streptococcus and intrapartum antibiotics: A multicentre retrospective cohort study. BJOG: An International Journal of Obstetrics & Gynaecology. 2023;130(1):24-31.

106. Chen X, Cao S, Fu X, Ni Y, Huang B, Wu J, et al. The risk factors for Group B Streptococcus colonization during pregnancy and influences of intrapartum antibiotic prophylaxis on maternal and neonatal outcomes. BMC Pregnancy & Childbirth. 2023;23(1):207.

107. Ching NS, Buttery JP, Lai E, Steer AC, Standish J, Ziffer J, et al. Breastfeeding and risk of late-onset Group B Streptococcal disease. Pediatrics. 2021;148(3):09.

108. Cicalese E, Lamouse-Smith E, Randis TM, Ratner AJ. Group B streptococcal transmission rates as determined by PCR. Journal of Perinatal Medicine. 2020;48(5):509-13.

109. Constantinou G, Ayers S, Mitchell EJ, Daniels J, Walker K, Harrison E, et al. The acceptability of implementing routine GBS testing from the perspectives of women and healthcare professionals participating in the GBS3 trial in the UK. Therapeutic Advances in Infectious Disease. 2023;Conference: Group B Strep in Pregnancy and Babies Conference. Virtual. 10:15.

110. Constantinou G, Ayers S, Mitchell EJ, Moore S, Jones AM, Downe S, et al. The acceptability of group B streptococcal bacteria (GBS) testing to women, including self-swabbing procedures: A qualitative study. Midwifery. 2024;135:104063.

111. Constantinou G, Ayers S, Mitchell EJ, Walker KF, Daniels J, Moore S, et al. Women's knowledge of and attitudes towards group B streptococcus (GBS) testing in pregnancy: a qualitative study. BMC Pregnancy & Childbirth. 2023;23(1):339.

112. Constantinou G, Ayers S, Mitchell EJ, Walker KF, Downe S, Jones AM, et al. The acceptability of implementation of group B Streptococcus testing: Perspectives from women and health professionals in the GBS3 trial: A qualitative study. Women & Birth: Journal of the Australian College of Midwives. 2024;37(6):101832.

113. Constantinou G, Webb R, Ayers S, Mitchell EJ, Daniels J. Acceptability and feasibility of maternal screening for Group B Streptococcus: a rapid review. medRxiv. 2024;28.

114. Copur D, Koyuncu Ozyurt O, Kandemir H, Ozhak B, Ogunc D, Mendilcioglu I. Comparison of polymerase chain reaction method with culture method in antenatal Group B Streptococcus screening. Ginekologia Polska. 2024;95(12):935-9.

115. Costa SC, Machado AP, Teixeira C, Cerqueira L, Rodrigues T, Ribeiro M, et al. Group B Streptococcus rectovaginal colonization screening on term pregnancies: culture or polymerase chain reaction? Journal of Maternal-Fetal & Neonatal Medicine. 2023;36(2):2262078.

116. Cynan M, Dixie M, Simpson J, Young L. Review of routine antibiotic treatment for preterm babies with low risk of early-onset sepsis at a level 3 neonatal intensive care unit. Archives of Disease in Childhood. 2024;109(Supplement 1):A152-A3.

117. d'Otreppe S, Lefevre P, Meex C, Devey A, Sacheli R, Gerard M, et al. Multicenter performance evaluation of the Revogene GBS DS Real-Time PCR Assay for Group B Streptococcus detection during labor. Molecular Diagnosis and Therapy. 2023;27(5):611-20.

118. Daniels J, Walker K, Mitchell E, Haines R, Bradshaw L, Ogollah R, et al. When routine data is not quite enough: A vertical audit within a massive cluster randomised trial. Trials Conference: 5th International Clinical Trials Methodology Conference, ICTMC. 2019;20(Supplement 1).

119. de Melo SCCS, Santos NCS, de OM, Scodro RBL, Cardoso RF, Padua RAF, et al. Antimicrobial susceptibility of Streptococcus agalactiae isolated from pregnant women. Revista do Instituto de Medicina Tropical de Sao Paulo. 2016;58(no pagination).

120. Delabaere A, Curinier S, Ughetto S, Gibold L, Bonnet R, Rossi A, et al. Accuracy of a rapid intrapartum group B Streptococcus test: A new immunochromatographic assay. Journal of Gynecology Obstetrics and Human Reproduction. 2017;46(5):449-53.

121. Desravines N, Venkatesh K, Hopkins AM, Grant M, McGuire C, Boggess KA. Intrapartum Group B Streptococcus antibiotic prophylaxis in Penicillin allergic pregnant women. Obstetrics and Gynecology Conference: 67th Annual Clinical and Scientific Meeting of the American College of Obstetricians and Gynecologists Nashville, TN United States. 2019;133(Suppl 1).

122. Desravines N, Venkatesh KK, Hopkins A, Waldron J, Grant M, McGuire C, et al. Intrapartum group b streptococcus antibiotic prophylaxis in penicillin allergic pregnant women. AJP Reports. 2019;9(3):E238-E43.

123. do Nascimento CS, Dos Santos NFB, Ferreira RCC, Taddei CR. Streptococcus agalactiae in pregnant women in Brazil: prevalence, serotypes, and antibiotic resistance. Brazilian Journal of Microbiology. 2019;50(4):943-52.

124. Doenhardt M, Seipolt B, Mense L, Winkler JL, Thurmer A, Rudiger M, et al. Neonatal and young infant sepsis by Group B Streptococci and Escherichia coli: a single-center retrospective analysis in Germany-GBS screening implementation gaps and reduction in antibiotic resistance. European Journal of Pediatrics. 2020;179(11):1769-77.

125. Duffy CR, Huang Y, Andrikopoulou M, Stern-Ascher CN, Wright JD, D'Alton ME, et al. Vancomycin during delivery hospitalizations for women with group B streptococcus. Journal of Maternal-Fetal & Neonatal Medicine. 2022;35(5):898-906.

126. Faby A, Anuradha M, Rachel R, Tripathy S, Raveendran SK. Screening of Group B Streptococcal and Candida infections in antenatal women visiting a tertiary care hospital and the neonatal outcome. Journal of Pure and Applied Microbiology. 2024;18(1):193-9.

127. Fakhraei R, El-Chaar D, Sander B, Thampi N, Brown K, Fell D. Estimating the burden of infant Group B Streptococcus disease in Ontario, Canada: a population-based cohort study. American Journal of Obstetrics and Gynecology. 2024;230(2 Supplement):S640.

128. Fakhraei R, Fell DB, El-Chaar D, Thampi N, Sander B, Brown KA, et al. Burden of infant group B Streptococcus disease and impact of maternal screening and antibiotic prophylaxis in Ontario, Canada: a population-based cohort study. The Lancet Regional Health - Americas. 2024;39(no pagination).

129. Farr A, Sustr V, Kiss H, Rosicky I, Graf A, Makristathis A, et al. Oral probiotics to reduce vaginal group B streptococcal colonization in late pregnancy. Scientific Reports. 2020;10(1):19745.

130. Ferreira MB, de-Paris F, Paiva RM, Nunes LS. Assessment of conventional PCR and real-time PCR compared to the gold standard method for screening Streptococcus agalactiae in pregnant women. Brazilian Journal of Infectious Diseases. 2018;22(6):449-54.

131. Fowler D, Evenhouse S, Corrigan C, MacGeorge C, Andrews A, Hayes G, et al. The impact of maternal penicillin allergy on neonatal care and health care cost. Journal of Allergy and Clinical Immunology. 2020;145(2 Supplement):AB86.

132. Fullston EF, Doyle MJ, Higgins MF, Knowles SJ. Clinical impact of rapid polymerase chain reaction (PCR) test for group B Streptococcus (GBS) in term women with ruptured membranes. Irish Journal of Medical Science. 2019;188(4):1269-74.

133. Fung TY, Sahota DS. How can we reduce neonatal sepsis after universal group B streptococcus screening? BMC Pregnancy & Childbirth. 2024;24(1):586.

134. Furfaro LL, Chang BJ, Payne MS. Detection of group B Streptococcus during antenatal screening in Western Australia: a comparison of culture and molecular methods. Journal of Applied Microbiology. 2019;127(2):598-604.

135. Furfaro LL, Nathan EA, Chang BJ, Payne MS. Group B streptococcus prevalence, serotype distribution and colonization dynamics in Western Australian pregnant women. Journal of Medical Microbiology. 2019;68(5):728-40.

136. Gad A, Alkhdr M, Terkawi R, Alsharif H, Ibrahim M, Amin R, et al. Associations between maternal bacteremia during the peripartum period and early-onset neonatal sepsis: a retrospective cohort study. BMC Pediatrics. 2024;24(1):526.

137. Galvez A, Di Az de Teran E, Espinosa JA, Perez-Pedregosa J, Bartha-Rasero JL, Del Valle JG, et al. Ligilactobacillus salivarius V4II-90 eradicates Group B Streptococcus colonisation during pregnancy: a randomised, double-blind, placebo-controlled trial. Beneficial Microbes. 2024;15(4):387-96.

138. Gerolymatos G, Karlovasiti P, Sianou A, Logothetis E, Kaparos G, Grigoriadis C, et al. Antenatal group B streptococcus detection in pregnant women: culture or PCR? Journal of Infection in Developing Countries. 2018;12(8):631-5.

139. Go H, Nagano N, Sato Y, Katayama D, Hara K, Akimoto T, et al. Procalcitonin-based antibiotic use for neonatal early-onset bacterial infections: pre- and post-intervention clinical study. Antibiotics. 2023;12(9):(no pagination).

140. Goh M, Tan HY, Ng YS, Tan I, Yeo KT, Ku CW, et al. Group B Streptococcus screening with antenatal culture and intrapartum polymerase chain reaction: A prospective cohort study. Annals of the Academy of Medicine, Singapore. 2024;53(5):331-3.

141. Gomi Y. Variation of antibiotic susceptibility of group b Streptococcus in Japan: A longterm population-based cohort study. Journal of Obstetrics and Gynaecology Research. 2020;46(Suppl 1):8-9.

142. Gondim M, Hata J. Kaiser neonatal early-onset sepsis risk score and histologic evidence of infection in antibiotic-treated group b streptococcus-positive mothers. Pediatric and Developmental Pathology. 2020;23(6):516.

143. Gopal Rao G, Hiles S, Bassett P, Lamagni T. Differential rates of group B streptococcus (GBS) colonisation in pregnant women in a racially diverse area of London, UK: a cross-sectional study. BJOG: An International Journal of Obstetrics & Gynaecology. 2019;126(11):1347-53.

144. Graham N, Johnson J. Prevalence of Group B streptococcus colonization in pregnant women in The Bahamas. West Indian Medical Journal. 2016;65(Supplement 5):64.

145. Greenbaum S, Sheiner E, Wainstock T, Segal I, Sergienko R, Walfisch A. Group B Streptococcus maternal colonization and respiratory infections in the offspring: lessons learned from an analysis of a population-based cohort with 18-year follow-up. Archives of Gynecology & Obstetrics. 2020;301(1):101-6.

146. Gurudas G, Arjun R, Jain N, Ranganayaki V, Sasikumar C, Mohan V, et al. Prevalence of Group B Streptococcus in pregnant women in Kerala and relation to neonatal outcomes: a prospective cross-sectional study. Journal of Tropical Pediatrics. 2022;68(6):06.

147. Guzman C, Howard D. The prevalence of expired GBS screens among women delivering at term. Obstetrics & Gynecology. 2020;135(Supplement 1):155S.

148. Hakim M, Jabour A, Anton M, Hakim M, Kheirallah S. Screening Arab Israeli pregnant women for Group B Streptococcus by the AmpliVue GBS Assay: Are the rates higher than the national average? Israel Medical Association Journal: Imaj. 2018;20(5):291-4.

149. Hamdan L, Vandekar S, Spieker AJ, Rahman H, Ndi D, Shekarabi ES, et al. Epidemiological trends of racial differences in early- and late-onset Group B Streptococcus disease in Tennessee. Clinical Infectious Diseases. 2021;73(11):e3634-e40.

150. Hanson S, Nelson G, Preszler M, Laible B, Nazir J, Siewert A. Antibiotic prescribing practices in Group B Streptococcus Positive obstetric patients with Penicillin allergy. South Dakota Medicine: The Journal of the South Dakota State Medical Association. 2022;75(10):462-8.

151. Hartvigsen CM, Nielsen SY, Moller JK, Khalil MR. Reduction of intrapartum antibiotic prophylaxis by combining risk factor assessment with a rapid bedside intrapartum polymerase chain reaction testing for group B streptococci. European Journal of Obstetrics, Gynecology, & Reproductive Biology. 2022;272:173-6.

152. Helmig RB, Gertsen JB. Intrapartum PCR-assay for detection of Group B Streptococci (GBS). European Journal of Obstetrics and Gynecology and Reproductive Biology: X. 2019;4(no pagination).

153. Herrera CA, McPherson JA, Vladutiu CJ, Smid MC. Neonatal morbidity associated with maternal Group B Streptococcal colonization in individuals undergoing planned cesarean delivery. Journal of Maternal-Fetal & Neonatal Medicine. 2023;36(1):2183740.

154. Hillier SL, Ferrieri P, Edwards MS, Ewell M, Ferris D, Fine P, et al. A phase 2, randomized, control trial of Group B Streptococcus (GBS) type III capsular polysaccharide-tetanus toxoid (GBS III-TT) vaccine to prevent vaginal colonization with GBS III. Clinical Infectious Diseases. 2019;68(12):2079-86.

155. HogenEsch E, De Mucio B, Haddad LB, Vilajeliu A, Ropero AM, Yildirim I, et al. Differences in maternal group B Streptococcus screening rates in Latin American countries. Vaccine. 2021;39 Suppl 2:B3-B11.

156. HogenEsch E, Haddad LB, Yildirim I, Omer SB. Disparities in group b streptococcus screening during pregnancy in Latin America. American Journal of Obstetrics and Gynecology. 2019;221(6):681.

157. Horvath-Puho E, van Kassel MN, Goncalves BP, de Gier B, Procter SR, Paul P, et al. Mortality, neurodevelopmental impairments, and economic outcomes after invasive group B streptococcal disease in early infancy in Denmark and the Netherlands: a national matched cohort study. The Lancet Child & Adolescent Health. 2021;5(6):398-407.

158. Hsu JF, Chen CL, Lee CC, Lien R, Chu SM, Fu RH, et al. Characterization of group B Streptococcus colonization in full-term and late-preterm neonates in Taiwan. Pediatrics & Neonatology. 2019;60(3):311-7.

159. Hung LC, Kung PT, Chiu TH, Su HP, Ho M, Kao HF, et al. Risk factors for neonatal early-onset group B streptococcus-related diseases after the implementation of a universal screening program in Taiwan. BMC Public Health. 2018;18(1):438.

160. Ishino A, Manickam R, Hastie J, Weintraub MR, Moore G. Expanded risk-based strategy for postpartum antibiotic prophylaxis in deliveries complicated by intraamniotic infection: Results of a pre- and post-intervention study. American Journal of Obstetrics and Gynecology. 2022;226(2):313.

161. Janczewska I, Jassem-Bobowicz J, Hinca K, Stefanska K, Domzalska-Popadiuk I. Group B Streptococcus colonization status and antibiotic use during labour - a single-centre observational study. Ginekologia Polska. 2024;95(7):549-56.

162. Ji Y, Zhao C, Ma XX, Peppelenbosch MP, Ma Z, Pan Q. Outcome of a screening program for the prevention of neonatal early-onset group B Streptococcus infection: a population-based cohort study in Inner Mongolia, China. Journal of Medical Microbiology. 2019;68(5):803-11.

163. Johansen NR, Kjaerbye-Thygesen A, Jonsson S, Westh H, Nilas L, Rorbye C. Prevalence and treatment of group B streptococcus colonization based on risk factors versus intrapartum culture screening. European Journal of Obstetrics, Gynecology, & Reproductive Biology. 2019;240:178-81.

164. Kabiri D, Paltiel O, Ofek-shlomai N, Nir-Paz R, Sompolinsky Y, Ezra Y. Membrane stripping in group B streptococcus carriers does not impede adequate intrapartum antibiotic prophylaxis: a retrospective study. Frontiers in Medicine. 2024;11(no pagination).

165. Kao Y, Tsai MH, Lai MY, Chu SM, Huang HR, Chiang MC, et al. Emerging serotype III sequence type 17 group B streptococcus invasive infection in infants: the clinical characteristics and impacts on outcomes. BMC Infectious Diseases. 2019;19(1):538.

166. Kasai Y, Komatsu M, Toyama Y, Nakano S, Hisata K, Yamada M, et al. Effect of probiotics on mother-to-neonate vertical transmission of group B streptococci: A prospective open-label randomized study. Pediatrics & Neonatology. 2024;65(2):145-51.

167. Khalid L, Khalid A, Rashid H. 298 Need for intrapartum antibiotics prophylaxis in women with prior history of gbs carriage. European Journal of Obstetrics and Gynecology and Reproductive Biology. 2022;Conference: EBCOG 2021. Athens Greece. 270:e85.

168. Khalil M, Uldbjerg N, Thorsen P, Moller J. Risk based approach versus culture based screening for identification of group B streptococci in laboring women. Journal of Perinatal Medicine. 2019;47(Supplement 1):eA179.

169. Khalil MR, Uldbjerg N, Thorsen PB, Moller JK. Risk-based approach versus culture-based screening for identification of group B streptococci among women in labor. International Journal of Gynaecology & Obstetrics. 2019;144(2):187-91.

170. Khalil MR, Uldbjerg N, Thorsen PB, Moller JK. Improvement of selection of pregnant women for intrapartum polymerase chain reaction screening for vaginal Group B Streptococci (GBS) colonization by adding GBS urine screening at 35-37 weeks of pregnancy. International Journal of Gynaecology & Obstetrics. 2020;151(1):124-7.

171. Khalil MR, Uldbjerg N, Thorsen PB, Moller JK. Urine dipstick for predicting intrapartum recto-vaginal colonisation by group B streptococci. Danish Medical Journal. 2020;67(2).

172. Kirven J, Beddow D, Patel L, Smith C, Booker KS, Dawud B, et al. Outcomes in reported penicillin allergic mothers and neonates requiring Group B streptococcal prophylaxis: a retrospective observational cohort study. BMC Pediatrics. 2021;21(1):327.

173. Kodama Y, Doi K, Yamashita R, Yamagichi T, Kaneko M, Sameshima H, et al. Intrauterine infection with poor perinatal outcome: A 20-year regional population-based study in Southern Japan. Reproductive Sciences. 2020;27(1 Supplement):309A.

174. Koebnick C, Sidell MA, Getahun D, Tartof SY, Rozema E, Taylor B, et al. Intrapartum antibiotic exposure and body mass index in children. Clinical Infectious Diseases. 2021;73(4):e938-e46.

175. Koh B, Farhat R, Lee A. Evaluating the utility of detection of GBS bacteriuria in addition to standard screening in pregnant women. Pathology. 2020;52(Supplement 1):S62.

176. Koliwer-Brandl H, Nil A, Birri J, Sachs M, Zimmermann R, Zbinden R, et al. Evaluation of two rapid commercial assays for detection of Streptococcus agalactiae from vaginal samples. Acta Obstetricia et Gynecologica Scandinavica. 2023;102(4):450-6.

177. Kovacs D, Papp K, Ajzner E. Streptococcus agalactiae detection rates in relation to different sampling methods of pregnant females. Clinical Chemistry and Laboratory Medicine. 2023;61(8):eA99.

178. Kowal E, Calhoun BC, Seybold DJ, Williams DL, Dietz PP. Universal screening for group b streptococcus versus universal treatment in pregnant women in appalachia unintended cost shifting consequences. Journal of Reproductive Medicine. 2019;64(3):183-7.

179. Kreicberga I, Pumpure E, Pantejeva T, Rostoka Z, Sleiers J. Group B Streptococcus early-onset neonatal sepsis in term neonates. European Journal of Obstetrics, Gynecology, & Reproductive Biology. 2019;Conference: 26th EBCOG European Congress of Obstetrics and Gynaecology. Paris France. 234:e106.

180. Kuder M, Lennox M, Pien L. Skin testing and oral Amoxicillin challenge in the outpatient Allergy and Clinical Immunology Clinic in Penicillin-allergic pregnant women. Journal of Allergy and Clinical Immunology. 2020;145(2 Supplement):AB99.

181. Kugelman N, Kleifeld S, Shaked-Mishan P, Assaf W, Marom I, Cohen N, et al. Group B Streptococcus real-time PCR may potentially reduce intrapartum maternal antibiotic treatment. Paediatric and Perinatal Epidemiology. 2022;36(4):548-52.

182. Kukovica I, Omahen N, Klobucar N, Bucar M, Franko Rutar A, Perme T, et al. Comparison of self-collected and healthcare worker-collected rectovaginal swabs for group B streptococcus detection in pregnancy using PCR with a commercial collection-enrichment device. Frontiers in Microbiology. 2025;16(no pagination).

183. Lee J, Naiduvaje K, Chew KL, Charan N, Chan YH, Lin RT, et al. Preventing early-onset group B streptococcal sepsis: clinical risk factor-based screening or culture-based screening? Singapore Medical Journal. 2021;62(1):34-8.

184. Lemaire C, Cheminet M, Duployez C, Artus M, Ballaa Y, Devos L, et al. A LAMP-based assay for the molecular detection of group B Streptococcus. European Journal of Clinical Microbiology & Infectious Diseases. 2023;42(10):1245-50.

185. Liu P, Feng Q, Liang Y, Wang X, Xiao Z, Huang L, et al. Maternal Group B Streptococcal rectovaginal colonization after intrapartum antibiotic prophylaxis. Children. 2022;9(12):(no pagination).

186. Liz CF, Soares S, Oliveira A. Prevention of group B streptococcus infection-results from a screening program. Archives of Disease in Childhood. 2019;104(Supplement 3):A141-A2.

187. Lohrmann F, Efstratiou A, Sorensen UBS, Creti R, Decheva A, Krizova P, et al. Maternal Streptococcus agalactiae colonization in Europe: data from the multi-center DEVANI study. Infection. 2025;53(1):373-81.

188. Lohrmann F, Hufnagel M, Kunze M, Afshar B, Creti R, Detcheva A, et al. Neonatal invasive disease caused by Streptococcus agalactiae in Europe: the DEVANI multi-center study. Infection. 2023;51(4):981-91.

189. Low JM, Lee JH, Foote HP, Hornik CP, Clark RH, Greenberg RG. Incidence of group B streptococcus early-onset sepsis in term neonates with second-line prophylaxis maternal intrapartum antibiotics: a multicenter retrospective study. American Journal of Obstetrics and Gynecology. 2024;230(6):673.e1-.e8.

190. Ma A, Thompson LA, Corsiatto T, Hurteau D, Tyrrell GJ. Epidemiological characterization of Group B Streptococcus infections in Alberta, Canada: An update from 2014 to 2020. Microbiology Spectrum. 2021;9(3):e0128321.

191. Ma H, Xu J, Zhang Y, Zhang R, Wu J. The current status of Group B Streptococcus infection in the reproductive tract of late-pregnancy women and its impact on pregnancy outcomes. Archives of Gynecology & Obstetrics. 2024;310(2):1151-5.

192. Maciuleviciute A, Bartuseviciene E, Tameliene R, Maleckiene L, Bartusevicius A, Mockeviciute E, et al. Analysis of different prophylaxis methods against early onset neonatal group B streptococcus infection in Lithuania. European Journal of Obstetrics, Gynecology, & Reproductive Biology. 2019;Conference: 26th EBCOG European Congress of Obstetrics and Gynaecology. Paris France. 234:e122.

193. Malik M, Nozad B, Majeed A. Should the NHS offer universal group B Streptococcus screening to pregnant women? Therapeutic Advances in Infectious Disease. 2023;Conference: Group B Strep in Pregnancy and Babies Conference. Virtual 10:3-4.

194. Manzanares S, Zamorano M, Naveiro-Fuentes M, Pineda A, Rodriguez-Granger J, Puertas A. Maternal obesity and the risk of group B streptococcal colonisation in pregnant women. Journal of Obstetrics & Gynaecology. 2019;39(5):628-32.

195. Mazabanda Lopez DA, Taboada Rubinos C, Hernandez Ortega A, Perez Guedes LDM, Urquia Marti L, Garcia-Munoz Rodrigo F. Management of neonates with 35 weeks of gestational age or more with infectious risk factors at birth: opportunities for improvement. Journal of Perinatal Medicine. 2022;50(8):1150-6.

196. McCoy JA, Bromwich K, Gerson KD, Levine LD. Association between intrapartum antibiotic prophylaxis for Group B Streptococcus colonization and clinical chorioamnionitis among patients undergoing induction of labor at term. American Journal of Obstetrics and Gynecology. 2023;229(6):672.e1-.e8.

197. McCoy JA, Bromwich KA, Gerson KD, Levine LD. Association between group B streptococcus positivity and maternal infectious morbidity. American Journal of Obstetrics and Gynecology. 2023;228(1 Supplement):S490-S1.

198. McCoy JA, Elovitz MA, Alby K, Koelper NC, Nissim I, Levine LD. Association of obesity with maternal and cord blood Penicillin levels in women with Group B Streptococcus colonization. Obstetrics & Gynecology. 2020;136(4):756-64.

199. McCoy JA, Peled T, Weiss A, Levine LD, Grisaru-Granovsky S, Rottenstreich M. Association between Group B Streptococcus and clinical Chorioamnionitis by gestational week at delivery-A multicenter cohort study. American Journal of Perinatology. 2025;42(2):181-8.

200. Meex C, Devey A, Defeche J, Bontems S, Descy J, Hayette MP, et al. Evaluation of the Revogene R GBS DS assay performance for the intrapartum screening of group B streptococcus in comparison with intrapartum culture. Diagnostic Microbiology & Infectious Disease. 2022;102(3):115616.

201. Menichini D, Longo M, Miselli F, Bedetti L, Monari F, Berardi A, et al. GBS antenatal screening and recto-vaginal colonization rates before and during the COVID-19 pandemic. Therapeutic Advances in Infectious Disease. 2022;Conference: Group B Strep in Pregnancy and Babies Conference. Virtual. 9:5-6.

202. Miselli F, Bedetti L, Minotti C, Costantini RC, Reggiani MLB, Tamburini C, et al. Call for action: preventive strategies for Group B Streptococcus late-onset sepsis are needed. Therapeutic Advances in Infectious Disease. 2023;Conference: Group B Strep in Pregnancy and Babies Conference. Virtual. 10:13-4.

203. Moni T, Portuhay S, Brouillette K, Bhatt V, Wu V, Wardha W, et al. Is it safe? Safety of penicillin testing and challenge in pregnancy with outcome of penicillin use at delivery in penicillin 'allergic' pregnant population. Allergy: European Journal of Allergy and Clinical Immunology. 2020;75(Suppl 109):601.

204. Moorhead R, Daley AJ, Lee LY, Gorelik A, Garland SM. Compliance with screening for and recommended management of maternal group B streptococcus carriage in pregnancy. Australian & New Zealand Journal of Obstetrics & Gynaecology. 2019;59(6):837-42.

205. Morgan JA, Hankins ME, Callais NA, Albritton CW, Vanchiere JA, Betcher RE, et al. Group B Streptococcus rectovaginal colonization and resistance patterns in HIV-positive compared to HIV-negative pregnant patients. American Journal of Perinatology. 2023;40(14):1573-8.

206. Mukhopadhyay S, Bryan M, Dhudasia MB, Quarshie W, Gerber JS, Grundmeier RW, et al. Intrapartum group B Streptococcal prophylaxis and childhood weight gain. Archives of Disease in Childhood Fetal & Neonatal Edition. 2021;106(6):649-56.

207. Nanduri S, Petit S, Baumbach J, Reingold A, Miller L, Harrison L, et al. Trends in group B streptococcal infections among infants <3 months of age and the potential impact of a maternal vaccine in the United States (2006-2014). Open Forum Infectious Diseases Conference: ID Week. 2016;3(Suppl 1).

208. Nanduri SA, Petit S, Smelser C, Apostol M, Alden NB, Harrison LH, et al. Epidemiology of invasive early-onset and late-onset Group B Streptococcal disease in the United States, 2006 to 2015: Multistate laboratory and population-based surveillance. JAMA Pediatrics. 2019;173(3):224-33.

209. Nielsen SY, Hoffmann-Lucke E, Henriksen TB, Hartvigsen CM, Helmig RB, Khalil MR, et al. Timing and dosage of intrapartum prophylactic penicillin for preventing early-onset group B streptococcal disease: assessing maternal and umbilical cord blood concentration. Archives of Disease in Childhood Fetal & Neonatal Edition. 2025;110(2):128-32.

210. O'Sullivan CP, Lamagni T, Patel D, Efstratiou A, Cunney R, Meehan M, et al. Group B streptococcal disease in UK and Irish infants younger than 90 days, 2014-15: a prospective surveillance study. The Lancet Infectious Diseases. 2019;19(1):83-90.

211. Pangerl S, Sundin D, Geraghty S. Adherence to screening and management guidelines of maternal Group B Streptococcus colonization in pregnancy. Journal of Advanced Nursing. 2022;78(10):3247-60.

212. Peng ZJ, Bao L. Effect of intrapartum antibiotic prophylaxis of group B streptococcus infection on the incidence and bacteriological profile of early-onset neonatal sepsis. Zhongguo Dangdai Erke Zazhi. 2022;24(1):49-53.

213. Petersen KB, Johansen HK, Rosthoj S, Krebs L, Pinborg A, Hedegaard M. Increasing prevalence of group B streptococcal infection among pregnant women. Danish Medical Journal. 2014;61(9):A4908.

214. Picchiassi E, Coata G, Babucci G, Giardina I, Summa V, Tarquini F, et al. Intrapartum test for detection of Group B Streptococcus colonization during labor. Obstetrical and Gynecological Survey. 2019;74(5):269-70.

215. Piffer S, Rizzello R, Pedron M, Dellanna L, Lauriola AL. Screening of group B Streptococcus infection in pregnancy and neonatal outcomes in the province of Trento, Italy. Infezioni in Medicina. 2022;30(2):254-62.

216. Place K, Rahkonen L, Nupponen I, Kruit H. Vaginal streptococcus B colonization is not associated with increased infectious morbidity in labor induction. Acta Obstetricia et Gynecologica Scandinavica. 2021;100(8):1501-10.

217. Plainvert C, Anselem O, Joubrel C, Marcou V, Falloukh A, Frigo A, et al. Persistence of group B Streptococcus vaginal colonization and prevalence of hypervirulent CC-17 clone correlate with the country of birth: a prospective 3-month follow-up cohort study. European Journal of Clinical Microbiology & Infectious Diseases. 2021;40(1):133-40.

218. Porzio S, Bianchi M. Adherence to universal screening for group B Streptococcus in pregnancy and prevalence of colonised pregnancies in Caserta province, Italy. Infezioni in Medicina. 2024;32(2):213-21.

219. Quinn S, Kilgarrif D, Friesen N. Neonatalsepsis: Current information and how we are doing? Archives of Disease in Childhood. 2021;106(Suppl 2):A57.

220. Roeckner JT, Peterson E, Wiseman T, Flores-Torres J, Duncan JR. Neonatal survival for periviable infants: Impact of antenatal interventions. American Journal of Obstetrics and Gynecology. 2022;226(1 Supplement):S207-S8.

221. Romagano M, Swaminarayan D, Fofah O, Williams S, Apuzzio J, Gittens-Williams L. Maternal antepartum antibiotic administration and patterns of bacterial resistance in early preterm neonates. American Journal of Obstetrics and Gynecology. 2019;221(6):696-7.

222. Romagano MP, Fofah O, Swaminarayan D, Williams S, Apuzzio JJ, Gittens-Williams L. Maternal antepartum antibiotic administration and patterns of bacterial resistance in early preterm neonates. Journal of Maternal-Fetal & Neonatal Medicine. 2022;35(8):1527-31.

223. Rosenberg LR, Normann AK, Henriksen B, Fenger-Gron J, Moller JK, Khalil MR. Risk-based screening and intrapartum group B streptococcus polymerase chain reactionresults reduce use of antibiotics during labour. Danish Medical Journal. 2020;67(11):20.

224. Rosenberger KD, Seibert A, Hormig S. Asymptomatic GBS bacteriuria during antenatal visits: To treat or not to treat? Nurse Practitioner. 2020;45(7):18-25.

225. Rowlands J, Wester R, Pradhan E, Perez P, Zansky SM. Trends in early and late onset group b streptococcus in New York State, 2000-2014. Open Forum Infectious Diseases Conference: ID Week. 2016;3(Supplement 1).

226. Sabroske EM, Iglesias MAS, Rench M, Moore T, Harvey H, Edwards M, et al. Evolving antibiotic resistance in Group B Streptococci causing invasive infant disease: 1970-2021. Pediatric Research. 2023;93(7):2067-71.

227. Santhanam S, Arun S, Rebekah G, Ponmudi NJ, Chandran J, Jose R, et al. Perinatal risk factors for neonatal early-onset Group B Streptococcal sepsis after initiation of risk-based maternal intrapartum antibiotic prophylaxis-A case control study. Journal of Tropical Pediatrics. 2018;64(4):312-6.

228. Seedat F, Geppert J, Stinton C, Patterson J, Brown CS, Tan B, et al. Universal antenatal culture-based screening for maternal Group B Streptococcus (Gbs) carriage to prevent early-onset Gbs Disease: A systematic review for the UK National Screening Committee (Nsc). Journal of Epidemiology and Community Health. 2017;71:A18.

229. Serra G, Lo Scalzo L, Giordano M, Giuffre M, Trupiano P, Venezia R, et al. Group B streptococcus colonization in pregnancy and neonatal outcomes: a three-year monocentric retrospective study during and after the COVID-19 pandemic. Italian Journal of Pediatrics. 2024;50(1):175.

230. Seto MTY, Ko JKY, Cheung KW, To KKW, Hui PW, Lao TT, et al. The accuracy of self-screening of Group B Streptococcus in pregnant women-A randomized Ccrossover study. Journal of Obstetrics & Gynaecology Canada: JOGC. 2019;41(6):792-7.

231. Sgayer I, Glikman D, Shqara RA, Maimon M, Rechnitzer H, Lowenstein L, et al. Maternal colonization with extended-spectrum beta-lactamase-producing Enterobacteriaceae in term versus preterm pregnancies. International Journal of Gynecology and Obstetrics. 2023;161(2):447-54.

232. Sharpe M, Shah V, Freire-Lizama T, Cates EC, McGrath K, David I, et al. Effectiveness of oral intake of Lactobacillus rhamnosus GR-1 and Lactobacillus reuteri RC-14 on Group B Streptococcus colonization during pregnancy: a midwifery-led double-blind randomized controlled pilot trial. Journal of Maternal-Fetal & Neonatal Medicine. 2021;34(11):1814-21.

233. Shibata M, Morozumi M, Maeda N, Komiyama O, Shiro H, Iwata S, et al. Relationship between intrapartum antibiotic prophylaxis and group B streptococcal colonization dynamics in Japanese mother-neonate pairs. Journal of Infection & Chemotherapy. 2021;27(7):977-83.

234. Shindler Y, Rahav G, Madar-Shapiro L, Abtibol J, Ravid M, Maor Y. Molecular patterns of streptococcus agalactiae (GBS) strains associated with different clinical syndromes: Early-onset disease in neonates, intrauterine infection, and vaginal colonization, an orthodox jewish community (OJC) residing in bney brak. Open Forum Infectious Diseases. 2019;6(Supplement 2):S136-S7.

235. Shipitsyna E, Shalepo K, Zatsiorskaya S, Krysanova A, Razinkova M, Grigoriev A, et al. Significant shifts in the distribution of vaccine capsular polysaccharide types and rates of antimicrobial resistance of perinatal group B streptococci within the last decade in St. Petersburg, Russia. European Journal of Clinical Microbiology & Infectious Diseases. 2020;39(8):1487-93.

236. Shukla D, Khan MNA, Paul R, Tiwari K, Singh K. Maternal-infant transmission and microbial dynamics of Group B Streptococcus: A comprehensive study in a tertiary care setting. Journal of Population Therapeutics and Clinical Pharmacology. 2024;31(8):236-41.

237. Snoek L, van Kassel MN, Krommenhoek JF, Achten NB, Plotz FB, van Sorge NM, et al. Neonatal early-onset infections: Comparing the sensitivity of the neonatal early-onset sepsis calculator to the Dutch and the updated NICE guidelines in an observational cohort of culture-positive cases. eClinicalMedicine. 2022;44:(no pagination).

238. Song KE, Hwang N, Ham JY, Cha HH, Chong GO, Lee NY. Prevalence of Group B Streptococcus colonization in pregnant women at a university hospital in Korea. Clinical Laboratory. 2022;68(8):01.

239. Subramaniam A, Blanchard CT, Ngek ESN, Mbah R, Welty E, Welty T, et al. Prevalence of group B streptococcus anogenital colonization and feasibility of an intrapartum screening and antibiotic prophylaxis protocol in Cameroon, Africa. International Journal of Gynaecology & Obstetrics. 2019;146(2):238-43.

240. Sumire S. Cost-effectiveness analysis of maternal immunisation against Group B streptococcus in Japan. Journal of Obstetrics and Gynaecology Research. 2021;47(8):2815.

241. Takahashi K, Sato Y, Ikeda K. Group B streptococcus neonatal umbilical colonization managed by dry cord care in nurseries: A retrospective cohort study. Pediatrics & Neonatology. 2021;62(5):506-11.

242. Tanno D, Saito K, Tomii Y, Nakatsuka Y, Uechi K, Ohashi K, et al. A multicenter study on the utility of selective enrichment broth for detection of Group B Streptococcus in pregnant women in Japan. Japanese Journal of Infectious Diseases. 2024;77(2):68-74.

243. Taylor M, Holzmann-Pazgal G. Case-control study does not demonstrate association between late-onset Group B Streptococcus and breast milk exposure. Journal of the Pediatric Infectious Diseases Society. 2022;11(9):389-90.

244. Tomlinson MW, Baker R, Ulrich J, Shah M, Marginean H, Girolami S. Maternal Group B Streptococcus prophylaxis improvement using an electronic medical record dynamic order set. American Journal of Perinatology. 2024;41(S01):e3124-e32.

245. Tran TNL, Pham TDK, Le TG, Le TMT, Bui NN, de Tran V, et al. Antibiotic resistance of Group B Streptococcus in pregnant women at 35-37 weeks of gestation in Southern Vietnam. Archives of the Balkan Medical Union. 2021;56(4):402-7.

246. Ujiie G, Murase M, Asai H, Igawa M, Okuyama A, Seo K, et al. Intrapartum prophylactic efficacy of ampicillin versus clindamycin in preventing vertical transmission of group B Streptococcus. Acta Paediatrica. 2024;113(7):1694-700.

247. Utekar T, Imcha M. 48 Cost benefit analysis for use of gbs pcr kit in pregnant patient to manage of early onset group-b streptococcus septicemia in neonates. European Journal of Obstetrics and Gynecology and Reproductive Biology. 2022;Conference: EBCOG 2021. Athens Greece. 270:e53.

248. Venkatesh KK, Vladutiu CJ, Glover AV, Strauss RA, Stringer JSA, Stamilio DM, et al. Is Group B Streptococcus colonization associated with maternal peripartum infection in an era of routine prophylaxis? American Journal of Perinatology. 2021;38(S01):e262-e8.

249. Virranniemi M, Raudaskoski T, Haapsamo M, Kauppila J, Renko M, Peltola J, et al. The effect of screening-to-labor interval on the sensitivity of late-pregnancy culture in the prediction of group B streptococcus colonization at labor: A prospective multicenter cohort study. Acta Obstetricia et Gynecologica Scandinavica. 2019;98(4):494-9.

250. Waisman D, Gover A, Molad M, Kedar R, Rotschild A, Benitz WE. While waiting for a vaccine: opportunities for optimization of neonatal group B streptococcal (GBS) disease prevention in Israel. Journal of Perinatology. 2019;39(2):331-8.

251. Walker K, Gray J, Petrou S, Ayers S, Ogollah R, Mitchell E, et al. The clinical and cost-effectiveness of testing for group B streptococcus: A cluster randomised trial with economic and acceptability evaluations (GBS3). BJOG: An International Journal of Obstetrics and Gynaecology. 2019;126(Supplement 1):109.

252. Wang CH, Kung WJ, Lee CH, Lee CF, Kao CL, Chen HC, et al. High rates of colonization and antimicrobial resistance of group B streptococcus highlight the need for vaccination even after implementation of guidelines for intrapartum antibiotic prophylaxis. Vaccine. 2022;40(2):282-7.

253. Wang L, Kalra A, Searns J, Bauer M. Outcomes of infants born to Group B Streptococcus-positive mothers with a reported Penicillin allergy. Journal of Allergy and Clinical Immunology. 2022;149(2 Supplement):AB70.

254. Wang LA, Baer R, Namazy J, Chambers C. Maternal Penicillin allergy and infant outcomes: Results from a large, administrative cohort. Journal of Allergy and Clinical Immunology. 2023;151(2 Supplement):AB184.

255. Wang M, Keighley C, Watts M, Plymoth M, McGee TM. Preventing early-onset Group B Streptococcus neonatal infection and reducing antibiotic exposure using a rapid PCR test in term prelabour rupture of membranes. Australian & New Zealand Journal of Obstetrics & Gynaecology. 2020;60(5):753-9.

256. Wang Y, Zhao Y, Zou L, Qiao J, Benitz WE. Regional Variation of Early-onset Neonatal Group B Streptococcal Disease Prevention Strategies in Mainland China. Pediatric Infectious Disease Journal. 2021;40(7):663-8.

257. Wang YX, Zhong M, Yi H, He HF. Detection of group B streptococcus colonization in cervical and lower vaginal secretions of pregnant women. Clinical and Experimental Obstetrics and Gynecology. 2020;47(5):669-74.

258. Wilcox H, Rios A, Santos RP, Wu V, Munshi U. Increase antibiotic exposure among infants with concern for chorioamnionitis based on isolated maternal fever. Pediatrics Conference: National Conference on Education. 2018;144(2).

259. Williams M, Zantow E, Turrentine M. Cost effectiveness of latest recommendations for Group B Streptococci screening in the United States. Obstetrics & Gynecology. 2020;135(4):789-98.

260. Yanni M, Stark M, Francis L, Francis JR, McMillan M, Baird R, et al. Neonatal Group B Streptococcal infection in Australia: A case-control study. Pediatric Infectious Disease Journal. 2023;42(5):429-35.

261. Yoshida E, Takeda J, Maruyama Y, Suga N, Takeda S, Arai H, et al. Prospective study of peripartum group B streptococcus colonization in Japanese mothers and neonates. Epidemiology & Infection. 2025;153:e1.

262. Zanin V, Parisi N, Visintini F, Driul L, Londero AP. Preventing group B Streptococcus neonatal disease with intrapartum prophylaxis: a retrospective study to detect its use in case of unknown colonization status. Minerva Obstetrics and Gynecology. 2023;75(1):18-26.

263. Zantow E, Williams M, Turrentine M. 771: Evaluating the cost effectiveness of the latest recommendations for Group B Streptococcus screening. American Journal of Obstetrics and Gynecology. 2020;222(1 Supplement):S488.

264. Zantow EW, Wyatt S, Peck JD, Maxted M, Grimes S, Edwards RK. Evaluating the guideline for group B streptococcus screening of pregnant women with hospital admissions prior to term. American Journal of Obstetrics and Gynecology. 2019;221(6):689.

265. Zdjelar S, Lakovic G, Mihajlovic S, Kubat J, Nikolic M. Management of term infants at risk for early onset GBS sepsis in corelation with mothers GBS screening status and intrapartum antibiotic exposure in all natural birth born term infants. Journal of Perinatal Medicine. 2019;47(Supplement 1):eA502.

266. Zhang H, Xu K, Liu Z, Shi Y, Li H, Yin X. Study on the relationship between intrapartum group B streptococcus prophylaxis and food allergy in children. Frontiers in Pediatrics. 2022;10(no pagination).

267. Zhou Y, Wang LQ, Yan Q, Lee CC, Hsu MH, Liao WT, et al. Genomic analysis of Group B Streptococcus from neonatal sepsis reveals clonal CC17 expansion and virulence- and resistance-associated traits after intrapartum antibiotic prophylaxis. Clinical Infectious Diseases. 2022;75(12):2153-60.

268. Zhu Y, Gao L, Huang ZL, Wu JY, Ni Y, Wang YJ, et al. Current status of group B Streptococcus infection in neonates: a multicenter prospective study. Zhongguo Dangdai Erke Zazhi. 2021;23(9):889-95.

269. Zhu Y, Huang J, Lin XZ, Chen C. Group B Streptococcus colonization in late pregnancy and invasive infection in neonates in China: A population-based 3-year study. Neonatology. 2019;115(4):301-9.

270. Zietek M, Jaroszewicz-Trzaska J, Szczuko M, Mantiuk R, Celewicz Z. Intrapartum PCR assay is a fast and efficient screening method for Group B Streptococcus detection in pregnancy. Ginekologia Polska. 2020;91(9):549-53.

271. Zoli SI, Baiti MA, Alhazmi AA, Khormi RM, Sayegh MA, Altubayqi T, et al. Factors affecting awareness of pregnancy screening for Group B Streptococcus infection among women of reproductive age in Jazan Province, Saudi Arabia. Journal of Multidisciplinary Healthcare. 2023;16:2109-16.

272. Zonnenberg IA, van Dijk-Lokkart EM, van den Dungen FAM, Vermeulen RJ, van Weissenbruch MM. Neurodevelopmental outcome at 2 years of age in preterm infants with late-onset sepsis. European Journal of Pediatrics. 2019;178(5):673-80.

273. Andreu A, Sanfeliu I, Vinas L, Barranco M, Bosch J, Dopico E, et al. [Decreasing incidence of perinatal group B streptococcal disease (Barcelona 1994-2002). Relation with hospital prevention policies]. Enfermedades Infecciosas y Microbiología Clínica. 2003;21(4):174-9.

274. Berardi A, Spada C, Reggiani MLB, Creti R, Baroni L, Capretti MG, et al. Group B Streptococcus early-onset disease and observation of well-appearing newborns. PLoS ONE. 2019;14(3):e0212784.

275. Berardi A, Trevisani V, Di Caprio A, Caccamo P, Latorre G, Loprieno S, et al. Timing of symptoms of early-onset sepsis after intrapartum antibiotic prophylaxis: can it inform the neonatal management? Pathogens. 2023;12(4):(no pagination).

276. Chen JC, Jenkins-Marsh S, Flenady V, Ireland S, May M, Grimwood K, et al. Early-onset group B streptococcal disease in a risk factor-based prevention setting: A 15-year population-based study. Australian & New Zealand Journal of Obstetrics & Gynaecology. 2019;59(3):422-9.

277. Eason J, Ward H, Danko O, Richardson K, Vaitkute R, McKeon-Carter R. Early-onset sepsis: can we screen fewer babies safely? Archives of Disease in Childhood. 2021;106(1):86-8.

278. Finale E, Spadea T, Mondo L, Arnulfo A, Capuano A, Ghiotti P, et al. Streptococcus agalactiae in pregnancy and the impact of recommendations on adherence to guidelines: an Italian area-based study. Journal of Maternal-Fetal & Neonatal Medicine. 2022;35(25):7826-30.

279. Freitas FT, Romero GA. Early-onset neonatal sepsis and the implementation of group B streptococcus prophylaxis in a Brazilian maternity hospital: a descriptive study. Brazilian Journal of Infectious Diseases. 2017;21(1):92-7.

280. Gilbert GL, Hewitt MC, Turner CM, Leeder SR. Compliance with protocols for prevention of neonatal group B streptococcal sepsis: practicalities and limitations. Infectious Diseases in Obstetrics and Gynecology. 2003;11(1):1-9.

281. Jalil RAA, Al-Khalifa SM, Dayoub N. The impact of group b streptococcus infection random screening during pregnancy on subsequent neonatal infection/admission rates. Bahrain Medical Bulletin. 2019;41(4):241-5.

282. Jourdan-da Silva N, Antona D, Six C. Neonatal group B streptococcus infections in France: incidence from 1997 to 2006 and current prevention practices in maternity wards. BEH. 2008;14:110–3.

283. Sagrera X, Raspall F, Sala P, et al. Evaluation of the efficacy of a protocol for the prevention of neonatal sepsis by group B streptococcus [Catalan]. Pediatr Catalana. 2001;61(1):17-21.

284. Scheftelowitz Cohen R, Chodik G, Eisenberg VH. Re-evaluating perinatal Group B Streptococcal screening in Israel - Is it time for a change in policy? Preventive Medicine. 2021;153:106716.

285. Simetka O, Petros M, Podesvova H. [Prevention of early-onset neonatal group B streptococcal infection: neonatal outcome after introduction of national screening guideline]. Ceska Gynekologie. 2010;75(1):41-6.

286. Sorg AL, Obermeier V, Armann J, Klemme M, von Kries R. [Decrease in Group B Streptococcal infections in neonates: Analysis of health insurance data 2005 to 2017]. Klinische Pädiatrie. 2021;233(1):17-23.

287. Sridhar S, Grace R, Nithya PJ, Balaji V, Niranjan T, Manish K, et al. Group B streptococcal infection in a tertiary hospital in India--1998-2010. Pediatric Infectious Disease Journal. 2014;33(10):1091-2.

288. Tapia IJ, Reichhard TC, Saldias RM, Abarzua CF, Perez AM, Gonzalez MA, et al. [Neonatal sepsis in the era of antenatal antibiotic prophylaxis]. Revista Chilena de Infectología. 2007;24(2):111-6.

289. Van Rossem EJD, Plotz FB, Felderhof MK. Evaluating the impact of the 2017 Dutch Neonatal Early-Onset Sepsis Guideline. Pediatric Infectious Disease Journal. 2023;42(9):811-5.

290. Yucesoy G, Caliskan E, Karadenizli A, Corakci A, Yucesoy I, Huseyinoglu N, et al. Maternal colonisation with group B streptococcus and effectiveness of a culture-based protocol to prevent early-onset neonatal sepsis. International Journal of Clinical Practice. 2004;58(8):735-9.
