# Supplementary material for: Benefits and Harms of Antenatal/Intrapartum Screening for Maternal Group B Streptococcus and Use of Intrapartum Antibiotic Prophylaxis Versus Risk‐Based Protocols or No Intervention: A Rapid Review
Source: Acta Paediatr. 2026 Apr 30;115(8):1598–610. doi: 10.1111/apa.70568 (PMC13371836; doi:10.1111/apa.70568)
Supplement: Supplementary file 6 — Data S6: GROOVE overlap. [file APA-115-1598-s007.docx]

## Supplementary materials File 6 (S6) GROOVE overlap of included studies

(1) Matrix of studies.

Overlap of primary studies reported within included systematic reviews

| **Primary studies** | **Systematic reviews** | | |
| --- | --- | --- | --- |
| **Study ID** | **Li 2020** | **Hasperhoven 2020** | **Panneflek 2024** |
| Abdelmaaboud 2011 | 1 |  | 1 |
| Alarcron 2004 |  |  | 1 |
| Al Luhidan 2019 | X |  | 1 |
| Andreu 2003 |  |  | 1 |
| Angstetra 2007 | 1 | 1 | 1 |
| Bauserman 2013 |  |  | 1 |
| Bekker 2014 |  | 1 | 1 |
| Bizzarro 2005 | 1 |  |  |
| Björklund 2017 | 1 |  | 1 |
| Björnsdóttir 2019 | X |  | 1 |
| Brozanski 2000 |  |  | 1 |
| Chan 2023 | X | X | 1 |
| Chen 2001 |  |  | 1 |
| Chen 2005 | 1 | 1 | 1 |
| Cho 2019 | X |  | 1 |
| Clemens & Gable 2002 |  |  | 1 |
| Coco 2002 |  |  | 1 |
| Darlow 2016 |  | 1 | 1 |
| Davies 2001 |  |  | 1 |
| Eberly & Rajnik 2009 |  |  | 1 |
| Ecker 2013 | 1 |  | 1 |
| Edwards 2003 | 1 | 1 | 1 |
| Eisenberg 2005 | 1 | 1 | 1 |
| ElHelali 2019 |  |  | 1 |
| Factor 1998 |  |  | 1 |
| Freitas & Romero 2017 |  |  | 1 |
| Garland 1991 |  |  | 1 |
| Gibbs 1994 |  |  | 1 |
| Gilson 2000 | 1 | 1 | 1 |
| Gopal Rao 2017 | 1 | 1 | 1 |
| Gosling 2002 |  |  | 1 |
| Hafner 1988 | 1 |  | 1 |
| Håkansson 2017 |  | 1 | 1 |
| Hong 2019 | X |  | 1 |
| Horváth 2013 |  |  | 1 |
| Hung 2018 |  | 1 | 1 |
| Isaacs & Royle 1999 |  |  | 1 |
| Jeffery & Moses Lahra 1998 |  |  | 1 |
| Johansson Gudjónsdóttir 2019 | X |  | 1 |
| Jourdan-da Silva 2008 |  |  | 1 |
| Katz 1994 |  |  | 1 |
| Katz 1999 |  |  | 1 |
| Ko 2021 | X | X | 1 |
| Lee 2021 | X | X | 1 |
| Levine 1999 |  |  | 1 |
| Lin 2011 |  |  | 1 |
| Locksmith 1999 | 1 |  | 1 |
| López Sastre 2005 |  |  | 1 |
| Lu 2022 | X | X | 1 |
| Lukacs & Schrag 2012 |  |  | 1 |
| Ma 2018 |  | 1 | 1 |
| Main & Slagle 2000 | 1 | 1 | 1 |
| Matsubara 2013 |  |  | 1 |
| Matsubara 2007 |  |  | 1 |
| O’Sullivan 2019 | X | 1 | 1 |
| Petersen 2014 |  |  | 1 |
| Phares 2008 |  | 1 | 1 |
| Poulain 1997 |  |  | 1 |
| Puopolo & Eichenwald 2010 | 1 |  | 1 |
| Reisner 2000 | 1 |  |  |
| Renner 2006 |  |  | 1 |
| Rottenstreich 2019 | X |  | 1 |
| Sagrera 2001 |  |  | 1 |
| Sakata 2012 |  |  | 1 |
| Schrag 2002 | 1 | 1 | 1 |
| Schushat 2002 |  |  | 1 |
| Share 2001 |  |  | 1 |
| Simetka 2010 |  |  | 1 |
| Sorg 2021 | X | X | 1 |
| Sridhar 2014 |  |  | 1 |
| Sutkin 2005 |  |  | 1 |
| Tapia 2007 |  |  | 1 |
| Towers & Briggs 2002 |  |  | 1 |
| Trijbels-Smeulders 2007 |  |  | 1 |
| Trijbels-Smeulders 2006 |  |  | 1 |
| Trollfors 2022 | X | X | 1 |
| Uy 2002 |  |  | 1 |
| van den Hoogen 2010 |  |  | 1 |
| van Dyke 2009 |  |  | 1 |
| Vergani 2002 | 1 | 1 | 1 |
| Wicker 2019 | X |  | 1 |
| Youden 2005 |  |  | 1 |
| Yucesoy 2004 | 1 | 1 | 1 |

(2)


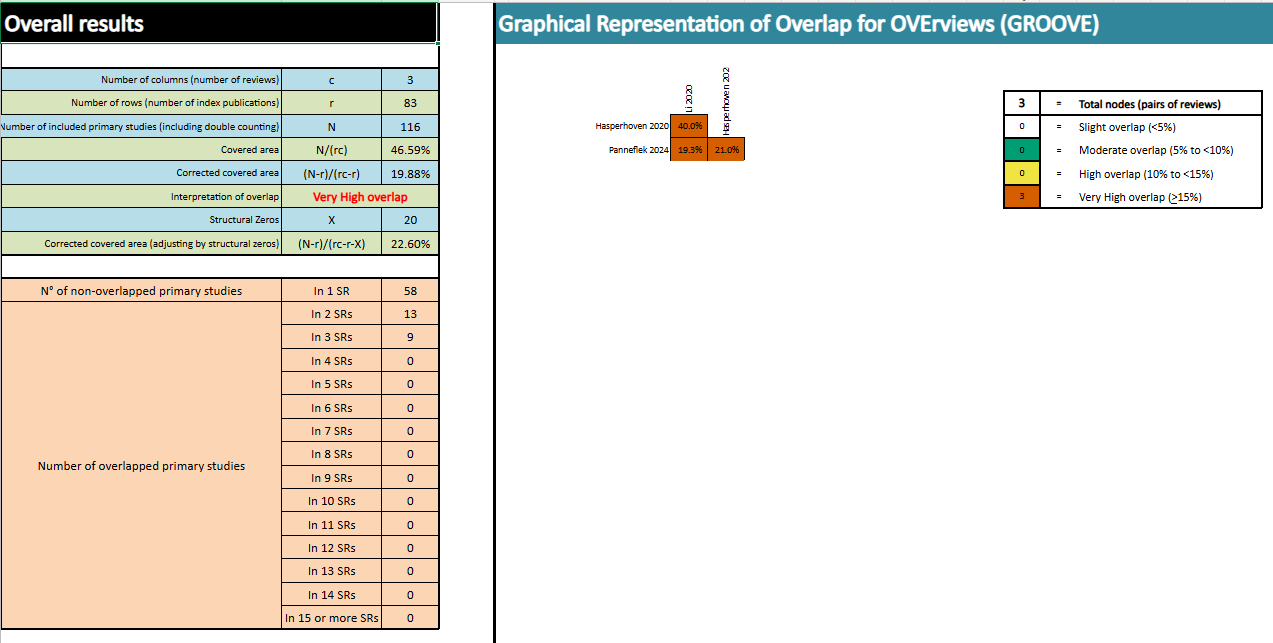


The primary studies depicted in figure S6

- include primary studies from Panneflek 2024 which are not presented in the main Table of included studies but are presented in additional supplementary files
- include primary studies excluded in Phase 2 as they were non-English publications and from low/middle income countries
