# Supplementary material for: Benefits and Harms of Antenatal/Intrapartum Screening for Maternal Group B Streptococcus and Use of Intrapartum Antibiotic Prophylaxis Versus Risk‐Based Protocols or No Intervention: A Rapid Review
Source: Acta Paediatr. 2026 Apr 30;115(8):1598–610. doi: 10.1111/apa.70568 (PMC13371836; doi:10.1111/apa.70568)
Supplement: Supplementary file 7 — Data S7: Overlap of primary studies within included reviews. [file APA-115-1598-s010.docx]

## Supplementary materials. File 7 (S7) Table of potentially linked primary studies

Overlap of primary studies reported within included systematic reviews is shown in S6

The primary studies depicted in figure S6

- include primary studies from Panneflek 2024 which are not presented in the main Table of included studies but are presented in additional supplementary files
- include primary studies excluded in Phase 2 as they were non-English publications and from low/middle income countries

The studies indicated below are potentially linked and there may be some crossover of populations

| **Primary study** | **Potentially linked study** | **Country and time period** | **Births (total/live)** | **Additional Notes** |
| --- | --- | --- | --- | --- |
| **Chan 2023** | | Hong Kong, 2009-2020 | 465,849 |  |
|  | Wang 2023 | Hong Kong, 2006-2017 | 490,034 |  |
|  | Ma 2018 | Hong Kong, 2012-2014 | 122,139 |  |
| **Hung 2018** | | Taiwan, 2005-2013 | 154,088 |  |
|  | Cho 2019 | Taiwan, 2011-2016 | 9845 |  |
|  | Ko 2021 | Taiwan, 2001 - 2018 | 84,182 |  |
|  | Lin 2011 | Taiwan, 2001-2008 | 32,614 |  |
|  | Lu 2022 | Taiwan, 2007-2018 | 30,293 |  |
| **Johansson Gudjonsdottir 2019** | | Sweden, 1975-2017 | 184,853 | Some crossover likely but not same population overall |
|  | Trollfors 2022 | Sweden, 2003-2016 | NR |  |
| **Puopolo 2010** | | USA, 1990-2007 | 162,135 |  |
|  | Chen 2005 | USA, 1990-2002 | 120,952 |  |
|  | Chen 2001 | USA, 1990-1996 | 60,196 |  |
| **Bekker 2014** | | Netherlands, 1987-2011 | NR |  |
|  | Trijbels-Smeulders 2007 | Netherlands, 1997-2001 | NR |  |
|  | Trijbels-Smeulders 2006 | Netherlands, 1997-1999 | NR |  |

**ABREVIATIONS:** NR: not reported; USA: United States of America
