# Supplementary material for: Benefits and Harms of Antenatal/Intrapartum Screening for Maternal Group B Streptococcus and Use of Intrapartum Antibiotic Prophylaxis Versus Risk‐Based Protocols or No Intervention: A Rapid Review
Source: Acta Paediatr. 2026 Apr 30;115(8):1598–610. doi: 10.1111/apa.70568 (PMC13371836; doi:10.1111/apa.70568)
Supplement: Supplementary file 8 — Data S8: EOGBS infection: Summary of meta‐analysis and GRADE judgements. [file APA-115-1598-s023.docx]

## Supplementary materials File 8. EOGBS infection: summary of meta-analysis and GRADE judgements

### File 8.1. Any strategy vs no strategy: EOGBS infection

EOGBS infection as reported by the included high-quality systematic reviews comparing any screening strategy versus no strategy

| **Review (Author, year)** | **Comparison** | **Population** | **Outcome** | **Number of studies (primary studies included in MA)** | **n (total)** | **Effect size (95% confidence interval)** | **Direction of effect** | **GRADE LEVEL (as reported by SR authors)** | **a. ROB, b. Inconsistency, c. Indirectness, d. Imprecision, e. Publication bias (Report downgrades applied by the SR authors)** | **Finding as reported by review authors (verbatim)** |
| --- | --- | --- | --- | --- | --- | --- | --- | --- | --- | --- |
| Panneflek 2024 | EOGBS infection in any strategy versus no strategy | Neonates | EOGBS infection | 34 studies (Andreu 2003, Bekker 2014, Darlow 2016, Eberly 2009, Ecker 2013, Factor 1998, Freitas 2017, Garland 1991, Gibbs 1994, Gosling 2002, Hakansson 2017, Horvath 2013, Isaacs 1999, Jeffery 1998, Johansson Gudjonsdottir 2019, Katz 1994, Katz 1999, Levine 1999, Lin 2011, Loepz Sastre 2005, Main 2000, Matsubara 2007, Matsubara 2013, O'Sullivan 2019, Petersen 2014, Poulain 1997, Puopolo 2010, Renner 2006, Sakata 2012, Share 2001, Simetka 2010, Tapia 2007, Towers 2002, Vergani 2002) | 10,041,490 | RR 0.46, 95% CI 0.36 to 0.60    Heterogeneity (I^2^) = 93% | Favours any strategy | Very-low | a. Most of the studies were assessed to be at serious risk of bias using the ROBINS-I (2 downgrades due to most studies serious study limitation) b. Considerable statistical heterogeneity I^2^=93%, P<0.001, and some overlap in 95%-CI estimates of studies (1 downgrade due to inconsistent finding in the studies). c. Not applicable in this review, because all studies include report a direct comparison. d. The 95%-CI is relatively narrow and excludes a RR of 1.0. Due to large pooled sample size, grading down is unnecessary. e. No apparent publication bias in funnel plot. Egger’s test for funnel plot asymmetry P = 0.065. | Any strategy (i.e. risk-based, universal or ‘other’) was  associated with a reduced risk of EOGBS infection  compared to no strategy |
| Panneflek 2024 | Any screening strategy vs no screening strategy | Neonates | EOGBS infection (term incidences) | 6 studies (Eberly 2009, Freitas 2017, Katz 1994, Renner 2006, Simetka 2010, Towers 2002) | 943,373 | RR 0.34, 95% CI 0.24 to 0.48    Heterogeneity (I^2^) = 11% | Favours any screening strategy | Low | a. Most of the studies were assessed to be at serious risk of bias using the ROBINS-I b. Unimportant statistical heterogeneity I^2^=11%, P = 0.35, and complete overlap in 95%-CI estimates of studies. c. Not applicable in this review, because all studies include report a direct comparison. d. The 95%-CI is relatively narrow and excludes a RR of 1.0. Due to large, pooled sample size, grading down is unnecessary. e. No apparent publication bias in funnel plot, but not enough studies to assess. |  |
| Panneflek 2024 | Any screening strategy vs no screening strategy | Neonates | EOGBS infection (sensitivity analysis) | 7 studies  (Factor 1998, Hakansson 2017, Main 2000, Petersen 2014, Simetka 2010, Tapia 2007, Vergani 2002) | 782,324 | RR 0.68, 95% CI 0.55 to 0.85    Heterogeneity (I^2^) = 33% | Favours any screening strategy | Moderate | a. Most of the studies were assessed to be at moderate risk of bias using the ROBINS-I. b. Moderate statistical heterogeneity I^2^=33%, P = 0.18, and complete overlap in 95%-CI estimates of studies. c. Not applicable in this review. d. The 95%-CI is relatively narrow and excludes a RR of 1.0. Due to large sample size, grading down is unnecessary e. No apparent publication bias in funnel plot, but not enough studies to assess. |  |

Abbreviations: EOS: early-onset sepsis, EOS-GBS: early-onset sepsis Group B Streptococcus, NOS: Newcastle Ottawa Scale, NS: not significant, ROB: risk of bias, RR: risk ratio* or relative risk**, SR: systematic review

**GRADE Working Group grades of evidence**
High quality: Further research is very unlikely to change our confidence in the estimate of effect.
Moderate quality: Further research is likely to have an important impact on our confidence in the estimate of effect and may change the estimate.
Low quality: Further research is very likely to have an important impact on our confidence in the estimate of effect and is likely to change the estimate.
Very low quality: We are very uncertain about the estimate.

### File 8.2. Universal v no strategy: EOGBS infection

EOGBS infection as reported by the included high-quality systematic reviews comparing universal screening strategies versus no strategy

| **Review (Author, year)** | **Comparison** | **Population** | **Outcome** | **Number of studies (primary studies included in MA)** | **n (total)** | **Effect size (95% confidence interval)** | **Direction of effect** | **GRADE LEVEL (as reported by SR authors)** | **a. ROB, b. Inconsistency, c. Indirectness, d. Imprecision, e. Publication bias (Report downgrades applied by the SR authors)** | **Finding as reported by review authors (verbatim)** |
| --- | --- | --- | --- | --- | --- | --- | --- | --- | --- | --- |
| Hasperhoven 2020 | Universal screening policy groups versus no policy groups | Neonates | EOGBS disease (defined as positive GBS culture from a normally sterile site  <7 days of age) | 4 studies  (Chen 2005, Main 2000, Phares 2008, Vergani 2002) | 3,172,204 | RR 0.31, 95% CI 0.11 to 0.84, P = 0.021    Heterogeneity (I^2^) = 90.9% | Favours universal screening | No GRADE | NA | Meta-analysis showed that universal screening was associated with a reduced risk of EOGBS disease when compared either with risk-based protocols or with no policy |
| Panneflek 2024 | EOGBS infection in universal strategies versus no strategy | Neonates | EOGBS infection | 16 studies (Andreu 2003, Eberly 2009, Ecker 2013, Garland 1991, Gibbs 1994, Jeffery 1998, Katz 1994, Lin 2011, Lopez Sastre 2005, Main 2000, Matsubara 2007, Matsubara 2013, Puopolo 2010, Sakata 2012, Simetka 2010, Tapia 2007) | 1,579,173 | RR 0.37, 95% CI 0.25 to 0.55    Heterogeneity (I^2^) = 78% | Favours universal screening strategy | Very-low | a. Most of the studies were assessed to be at serious risk of bias using the ROBINS-I. b. Considerable statistical heterogeneity I^2^=78%, P<0.001, and some overlap in 95%-CI estimates of studies. c. Not applicable in this review. d. The 95%-CI is relatively narrow and excludes a RR of 1.0. Due to large, pooled sample size, grading down is unnecessary. e. No apparent publication bias in funnel plot. Egger’s test for funnel plot asymmetry P = 0.561. | Similarly, risk-based and universal  were also  associated with reduced risk of EOGBS infection  compared to no strategy |
| Panneflek 2024 | Universal screening strategy versus no screening strategy | Neonates | EOGBS infection (term incidences) | 3 studies  (Eberly 2009, Katz 1994, Simetka 2010) | 490,024 | RR 0.26, 95% CI 0.13 to 0.54    Heterogeneity (I^2^) = 9% | Favours universal screening strategy | Low | a. Most of the studies were assessed to be at serious risk of bias using the ROBINS-I. b. Unimportant statistical heterogeneity I^2^=9%, P = 0.33, and complete overlap in 95%-CI estimates of studies. c. Not applicable in this review. d. The 95%-CI is relatively narrow and excludes a RR of 1.0. Due to large, pooled sample size, grading down is unnecessary. e. No apparent publication bias in funnel plot, but not enough studies to assess. |  |
| Panneflek 2024 | Universal screening strategy versus no screening strategy | Neonates | EOGBS infection (sensitivity analysis) | 3 studies  (Main 2000, Simetka 2010, Tapia 2007) | 43,580 | RR 0.17, 95% CI 0.03 to 0.90    Heterogeneity (I^2^) = 55% | Favours universal screening strategy | Moderate | a. Most of the studies were assessed to be at moderate risk of bias using the ROBINS-I. b. Substantial statistical heterogeneity I^2^=55%, P = 0.11, but complete overlap in 95%-CI estimates of studies. c. Not applicable in this review. d. The 95%-CI is relatively narrow and excludes a RR of 1.0. Due to large sample size, grading down is unnecessary. e. No apparent publication bias in funnel plot, but not enough studies to assess. |  |

Abbreviations: EOS: early-onset sepsis, EOS-GBS: early-onset sepsis Group B Streptococcus, NOS: Newcastle Ottawa Scale, NS: not significant, ROB: risk of bias, RR: risk ratio* or relative risk**, SR: systematic review

**GRADE Working Group grades of evidence**
High quality: Further research is very unlikely to change our confidence in the estimate of effect.
Moderate quality: Further research is likely to have an important impact on our confidence in the estimate of effect and may change the estimate.
Low quality: Further research is very likely to have an important impact on our confidence in the estimate of effect and is likely to change the estimate.
Very low quality: We are very uncertain about the estimate.

### File 8.3: Risk-based vs no strategy: EOGBS infection

EOGBS infection as reported by the included high-quality systematic reviews comparing risk-based approaches versus no strategy

| **Review (Author, year)** | **Comparison** | **Population** | **Outcome** | **Number of studies (primary studies included in MA)** | **n (total)** | **Effect size (95% confidence interval)** | **Direction of effect** | **GRADE LEVEL (as reported by SR authors)** | **a. ROB, b. Inconsistency, c. Indirectness, d. Imprecision, e. Publication bias (Report downgrades applied by the SR authors)** | **Finding as reported by review authors (verbatim)** |
| --- | --- | --- | --- | --- | --- | --- | --- | --- | --- | --- |
| Hasperhoven 2020 | Risk-based policy groups versus no policy groups | Neonates | EOGBS disease (defined as positive GBS culture from a normally sterile site  <7 days of age) | 7 studies (Bekker 2014, Chen 2005, Darlow 2016, Hakansson 2017, Main 2000, O'Sullivan 2019, Vergani 2002) | 7,506,263 | RR 0.86, 95% CI 0.61 to 1.20, P = 0.382    Heterogeneity (I^2^) = 89.36% | No harm or benefit | No GRADE | NA | Meta-analysis could not demonstrate a significant effect of risk-based protocols versus no policy |
| Panneflek 2024 | EOGBS infection in risk-based strategy versus no strategy | Neonates | EOGBS infection | 11 studies (Darlow 2016, Ecker 2013, Factor 1998, Hakansson 2017, Johansson Gudjonsdottir 2019, Main 2000, O'Sullivan 2019, Petersen 2014, Puopolo 2010, Towers 2002, Vergani 2002) | 3,004,723 | RR 0.65, 95% CI 0.48 to 0.87    Heterogeneity (I^2^) = 82% | Favours risk-based strategy | Very-low | a. Most of the studies were assessed to be at serious risk of bias using the ROBINS-I. b. Considerable statistical heterogeneity I^2^=82%, P<0.001, and some overlap in 95%-CI estimates of studies. c. The 95%-CI is relatively narrow and excludes a RR of 1.0. Due to large, pooled sample size, grading down is unnecessary. e. The funnel plot demonstrates a high probability of publication bias. Egger’s test for funnel plot asymmetry P = 0.006. | Similarly, risk-based and universal  were also  associated with reduced risk of EOGBS infection  compared to no strategy |
| Panneflek 2024 | Risk-factor based screening strategy vs no screening strategy | Neonates | EOGBS infection (term incidences) | 1 study  (Towers 2002) | 46,959 | RR 0.25, 95% CI 0.10 to 0.59    Heterogeneity (I^2^) = NA | Favours risk-based strategy | Low | a. Study at serious risk of bias. b. Only one study c. Not applicable in this review, because all studies include report a direct comparison. d. The 95%-CI is relatively narrow and excludes a RR of 1.0. Although small sample size, no downgrade, because effect large enough. Only one study. |  |
| Panneflek 2024 | Risk-factor based screening strategy vs no screening strategy | Neonates | EOGBS infection (sensitivity analysis) | 5 studies  (Factor 1998, Hakansson 2017, Main 2000, Petersen 2014, Vergani 2002) | 731,819 | RR 0.74, 95% CI 0.59 to 0.93    Heterogeneity (I^2^) = 14% | Favours risk-based strategy | Moderate | a. Most of the studies were assessed to be at moderate risk of bias using the ROBINS-I. b. Unimportant statistical heterogeneity I^2^=14%, P = 0.32, and complete overlap in 95%-CI estimates of studies. c. Not applicable in this review. d. The 95%-CI is relatively narrow and excludes a RR of 1.0. Due to large sample size, grading down is unnecessary e. No apparent publication bias in funnel plot, but not enough studies to assess. |  |

Abbreviations: EOS: early-onset sepsis, EOS-GBS: early-onset sepsis Group B Streptococcus, NOS: Newcastle Ottawa Scale, NS: not significant, ROB: risk of bias, RR: risk ratio* or relative risk**, SR: systematic review

**GRADE Working Group grades of evidence**
High quality: Further research is very unlikely to change our confidence in the estimate of effect.
Moderate quality: Further research is likely to have an important impact on our confidence in the estimate of effect and may change the estimate.
Low quality: Further research is very likely to have an important impact on our confidence in the estimate of effect and is likely to change the estimate.
Very low quality: We are very uncertain about the estimate.

### File 8.4. Universal v risk-based strategy: EOGBS infection

EOGBS infection as reported by the included high-quality systematic reviews comparing universal screening strategies versus risk-based approaches

| **Review (Author, year)** | **Comparison** | **Population** | **Outcome** | **Number of studies (primary studies included in MA)** | **n (total)** | **Effect size (95% confidence interval)** | **Direction of effect** | **GRADE LEVEL (as reported by SR authors)** | **a. ROB, b. Inconsistency, c. Indirectness, d. Imprecision, e. Publication bias (Report downgrades applied by the SR authors)** | **Finding as reported by review authors (verbatim)** |
| --- | --- | --- | --- | --- | --- | --- | --- | --- | --- | --- |
| Hasperhoven 2020 | Universal screening policy groups versus risk-based policy groups | Neonates | EOGBS disease (defined as positive GBS culture from a normally sterile site  <7 days of age) | 10 studies (Angstetra 2007, Chen 2005, Edwards 2003, Eisenberg 2005, Gilson 2000, Gopal Rao 2017, Main 2000, Schrag 2002, Vergani 2002, Yucesoy 2004) | 931,794 | RR 0.43, 95% CI 0.32 to 0.56, P<0.00001    Heterogeneity (I^2^) = 13% | Favours universal screening | No GRADE | NA | Meta-analysis showed that universal screening was associated with a reduced risk of EOGBS disease when compared either with risk-based protocols or with no policy |
| Li 2020 | Screening-based vs Risk-based strategy | Neonates | GBS-EOS | 18 studies (Locksmith 1999, Gilson 2000, Main 2000, Reisner 2000, Schrag 2002, Yucesoy 2004, Angstetra 2007, Bizzarro 2008, Puopolo 2010, Abdelmaaboud 2011, Rao 2017, Hafner 1998, Vergani 2002, Edwards 2003, Chen 2005, Eisenberg 2005, Ecker 2013, Bjorklund 2017) | 604,869 | RR 0.45, 95% CI 0.34 to 0.59, P = 0.02    Heterogeneity (I^2^) = 45% | Favours screening | No GRADE | NA | The pooled analysis showed that the incidence of GBS-EOS for screening-based strategy was significantly lower than that for risk-based strategy |
| Li 2020 | Screening-based vs Risk-based strategy | Neonates | GBS-EOS (High NOS) | 11 studies (Locksmith 1999, Gilson 2000, Main 2000, Reisner 2000, Schrag 2002, Yucesoy 2004, Angstetra 2007, Bizzarro 2008, Puopolo 2010, Abdelmaaboud 2011, Rao 2017) | 378,434 | RR 0.44, 95% CI 0.32 to 0.62, P = 0.05    Heterogeneity (I^2^) = 46% | Favours screening | No GRADE | NA | In subgroup analyses according to NOS score, the RR showed lower incidence of GBS-EOS in screening-  based group when the analysis was restricted to high-quality  studies |
| Li 2020 | Screening-based vs Risk-based strategy | Neonates | GBS-EOS (Low NOS) | 7 studies  (Hafner 1998, Vergani 2002, Edwards 2003, Chen 2005, Eisenberg 2005, Ecker 2013, Bjorklund 2017) | 226,435 | RR 0.45, 95% CI 0.28 to 0.75, P = 0.06    Heterogeneity (I^2^) = 51% | Favours screening | No GRADE | NA | The RR  for low score studies showed similar results [to the high score studies] |
| Panneflek 2024 | EOGBS infection in universal strategies versus risk strategies | Neonates | EOGBS infection | 17 studies (Abdelmaaboud 2011, Al Luhidan 2019, Bjorklund 2017, Chan 2023, Ecker 2013, Edwards 2003, Eisenberg 2005, Gilson 2000, Gopal Rao 2017, Hafner 1998, Hong 2019, Lee 2021, Main 2000, Puopolo & Eichenwald 2010, Rottenstreich 2019, Schrag 2002, Yucesoy 2004) | 1,806,092 | RR 0.41, 95% CI 0.30 to 0.55    Heterogeneity (I^2^) = 60% | Favours universal screening strategy | Low | a. Most of the studies were assessed to be at serious risk of bias using the ROBINS-I. b. Substantial statistical heterogeneity I^2^=60%, P<0.001, but considerable overlap in 95%-CI estimates of studies. c. Not applicable in this review. d. The 95%-CI is relatively narrow and excludes a RR of 1.0. Due to large, pooled sample size, grading down is unnecessary. e. No apparent publication bias in funnel plot. Egger’s test for funnel plot asymmetry P = 0.925. | In direct comparison, universal strategies were significantly associated with a reduced risk of EOGBS infection  compared to risk-based strategies |
| Panneflek 2024 | Universal screening strategy versus risk-factor based screening strategy | Neonates | EOGBS infection (term incidences) | 8 studies  (Bjorklund 2017, Chan 2023, Edwards 2003, Gilson 2000, Hong 2019, Lee 2021, Main 2000, Rottenstreich 2019) | 709,956 | RR 0.29, 95% CI 0.17 to 0.51    Heterogeneity (I^2^) = 37% | Favours universal screening strategy | Low | a. Most of the studies were assessed to be at serious risk of bias using the ROBINS-I. b. Moderate statistical heterogeneity I^2^=37%, P = 0.013, and almost complete overlap in 95%-CI estimates of studies. c. Not applicable in this review. d. The 95%-CI is relatively narrow and excludes a RR of 1.0. Due to large, pooled sample size, grading down is unnecessary. e. No apparent publication bias in funnel plot, but not enough studies to assess. |  |
| Panneflek 2024 | Universal screening strategy versus risk-factor based screening strategy | Neonates | EOGBS infection (sensitivity analysis) | 7 studies  (Bjorklund 2017, Eisenberg 2005, Gilson 2000, Gopal Rao 2017, Main 2000, Schrag 2002, Yucesoy 2004) | 765,850 | RR 0.46, 95% CI 0.37 to 0.57    Heterogeneity (I^2^) = 19% | Favours universal screening strategy | Moderate | a. Most of the studies were assessed to be at moderate risk of bias using the ROBINS-I. b. Unimportant statistical heterogeneity I^2^=19%, P = 0.28, and complete overlap in 95%-CI estimates of studies. c. Not applicable in this review. d. The 95%-CI is relatively narrow and excludes a RR of 1.0. Due to large sample size, grading down is unnecessary. e. No apparent publication bias in funnel plot, but not enough studies to assess. |  |

Abbreviations: EOS: early-onset sepsis, EOS-GBS: early-onset sepsis Group B Streptococcus, NOS: Newcastle Ottawa Scale, NS: not significant, ROB: risk of bias, RR: risk ratio* or relative risk**, SR: systematic review

**GRADE Working Group grades of evidence**
High quality: Further research is very unlikely to change our confidence in the estimate of effect.
Moderate quality: Further research is likely to have an important impact on our confidence in the estimate of effect and may change the estimate.
Low quality: Further research is very likely to have an important impact on our confidence in the estimate of effect and is likely to change the estimate.
Very low quality: We are very uncertain about the estimate.
