# Supplementary material for: Benefits and Harms of Antenatal/Intrapartum Screening for Maternal Group B Streptococcus and Use of Intrapartum Antibiotic Prophylaxis Versus Risk‐Based Protocols or No Intervention: A Rapid Review
Source: Acta Paediatr. 2026 Apr 30;115(8):1598–610. doi: 10.1111/apa.70568 (PMC13371836; doi:10.1111/apa.70568)
Supplement: Supplementary file 10 — Data S10: Other infection: Summary of meta‐analysis and GRADE judgements. [file APA-115-1598-s006.docx]

## Supplementary materials File 10. Other infection: summary of meta-analysis and GRADE judgements

### File 10.1: Any strategy vs no strategy: All EOS and non-GBS infection

All EOS and non-GBS infections as reported by the included high-quality systematic reviews comparing any screening strategy versus no strategy

| **Review (Author, year)** | **Comparison** | **Population** | **Outcome** | **Number of studies (primary studies included in MA)** | **n (total)** | **Effect size (95% confidence interval)** | **Direction of effect** | **GRADE LEVEL (as reported by SR authors)** | **a. ROB, b. Inconsistency, c. Indirectness, d. Imprecision, e. Publication bias (Report downgrades applied by the SR authors)** | **Finding as reported by review authors (verbatim)** |
| --- | --- | --- | --- | --- | --- | --- | --- | --- | --- | --- |
| Panneflek 2024 | Any screening strategy vs no screening strategy | Neonates | All EOS | 14 studies  (Ecker 2013, Freitas 2017, Isaacs1999, Johansson Gudjonsdottir 2019, Levine 1999, Lin 2011, Lopez Sastre 2005, Lukacs and Schrag 2012, Main 2002, Puopolo & Eichenwald 2010, Tapia 2007, Towers 2002, van den Hoogen 2010, Vergani 2002) | 3,878,681 | RR 0.60, 95% CI 0.48 to 0.74    Heterogeneity (I^2^) = 85% | Favours any screening strategy | Very-low | a. Most of the studies were assessed to be at serious risk of bias using the ROBINS-I. b. Considerable statistical heterogeneity I^2^=85%, P<0.001, and some overlap in 95%-CI estimates of studies. c. Not applicable in this review. d. The 95%-CI is relatively narrow and excludes a RR of 1.0. Due to large sample size, grading down is unnecessary e. No apparent publication bias in funnel plot, but not enough studies to assess. Egger’s test for funnel plot asymmetry P = 0.064. | Similar to EOGBS  infection comparisons, any, risk-based and universal  strategies were all significantly associated with a  reduced risk of all EOS compared to no strategy |
| Panneflek 2024 | Any screening strategy vs no screening strategy | Neonates | Non-GBS EOS | 13 studies  (Ecker 2013, Freitas 2017, Isaacs 1999, Johansson Gudjonsdottir 2019, Levine 1999, Lin 2011, Lopez Sastre 2005, Main 2000, Puopolo & Eichenwald 2010, Tapia 2007, Towers 2002, van den Hoogen 2010, Vergani 2002) | 1,357,432 | RR 0.75, 95% CI 0.56 to 0.99    Heterogeneity (I^2^) = 68% | Favours any screening strategy | Low | a. Most of the studies were assessed to be at serious risk of bias using the ROBINS-I. b. Substantial statistical heterogeneity I^2^=68%, P<0.001, and some overlap in 95%-CI estimates of studies. c. Not applicable in this review. d. The 95%-CI is relatively narrow and excludes a RR of 1.0. Due to large sample size, grading down is unnecessary e. No apparent publication bias in funnel plot. Egger’s test for funnel plot asymmetry P = 0.761. | Non-GBS EOS incidence  decreased after implementation of any strategy |

Abbreviations: EOS: early-onset sepsis, EOS-GBS: early-onset sepsis Group B Streptococcus, NOS: Newcastle Ottawa Scale, NS: not significant, ROB: risk of bias, RR: risk ratio* or relative risk**

**GRADE Working Group grades of evidence**
High quality: Further research is very unlikely to change our confidence in the estimate of effect.
Moderate quality: Further research is likely to have an important impact on our confidence in the estimate of effect and may change the estimate.
Low quality: Further research is very likely to have an important impact on our confidence in the estimate of effect and is likely to change the estimate.
Very low quality: We are very uncertain about the estimate.

### File 10.2: Universal v no strategy: All EOS and non-GBS infection

All EOS and non-GBS infections as reported by the included high-quality systematic reviews comparing universal screening strategies versus no strategy

| **Review (Author, year)** | **Comparison** | **Population** | **Outcome** | **Number of studies (primary studies included in MA)** | **n (total)** | **Effect size (95% confidence interval)** | **Direction of effect** | **GRADE LEVEL (as reported by SR authors)** | **a. ROB, b. Inconsistency, c. Indirectness, d. Imprecision, e. Publication bias (Report downgrades applied by the SR authors)** | **Finding as reported by review authors (verbatim)** |
| --- | --- | --- | --- | --- | --- | --- | --- | --- | --- | --- |
| Panneflek 2024 | Universal screening strategy versus no screening strategy | Neonates | All EOS | 7 studies  (Ecker 2013, Lin 2011, Lopez Sastre 2005, Lukacs and Schrag 2012, Main & Slagle 2002, Puopolo & Eichenwald 2010, Tapia 2007) | 2,120,056 | RR 0.60, 95% CI 0.45 to 0.80    Heterogeneity (I^2^) = 78% | Favours universal screening strategy | Very-low | a. Most of the studies were assessed to be at serious risk of bias using the ROBINS-I. b. Considerable statistical heterogeneity I^2^=78%, P<0.001, and some overlap in 95%-CI estimates of studies. c. Not applicable in this review. d. The 95%-CI is relatively narrow and excludes a RR of 1.0. Due to large sample size, grading down is unnecessary e. No apparent publication bias in funnel plot, but not enough studies to assess. | Similar to EOGBS  infection comparisons, any, risk-based and universal  strategies were all significantly associated with a  reduced risk of all EOS compared to no strategy |
| Panneflek 2024 | Universal screening strategy versus no screening strategy | Neonates | Non-GBS EOS | 6 studies  (Ecker 2013, Lin 2011, Lopez Sastre 2005, Main & Slagle 2000, Puopolo & Eichenwald 2010, Tapia 2007) | 395,440 | RR 0.86, 95% CI 0.71 to 1.04    Heterogeneity (I^2^) = 2% | No harm or benefit | Very-low | a. Most of the studies were assessed to be at serious risk of bias using the ROBINS-I. b. Unimportant statistical heterogeneity I^2^=2%, P = 0.40, and complete overlap in 95%-CI estimates of studies. c. Not applicable in this review. d. The 95%-CI is wide and includes a RR of 1.0. Sample size is not sufficiently large to detect a precise effect. e. No apparent publication bias in funnel plot, but not enough studies to assess. |  |

Abbreviations: EOS: early-onset sepsis, EOS-GBS: early-onset sepsis Group B Streptococcus, NOS: Newcastle Ottawa Scale, NS: not significant, ROB: risk of bias, RR: risk ratio* or relative risk**, SR: systematic review

**GRADE Working Group grades of evidence**
High quality: Further research is very unlikely to change our confidence in the estimate of effect.
Moderate quality: Further research is likely to have an important impact on our confidence in the estimate of effect and may change the estimate.
Low quality: Further research is very likely to have an important impact on our confidence in the estimate of effect and is likely to change the estimate.
Very low quality: We are very uncertain about the estimate.

File 10.3: Risk-based vs no strategy: All EOS and non-GBS EOS infection

All EOS and non-GBS infections as reported by the included high-quality systematic reviews comparing risk-based approaches versus no strategy

| **Review (Author, year)** | **Comparison** | **Population** | **Outcome** | **Number of studies (primary studies included in MA)** | **n (total)** | **Effect size (95% confidence interval)** | **Direction of effect** | **GRADE LEVEL (as reported by SR authors)** | **a. ROB, b. Inconsistency, c. Indirectness, d. Imprecision, e. Publication bias (Report downgrades applied by the SR authors)** | **Finding as reported by review authors (verbatim)** |
| --- | --- | --- | --- | --- | --- | --- | --- | --- | --- | --- |
| Panneflek 2024 | Risk-factor based screening strategy vs no screening strategy | Neonates | All EOS | 6 studies  (Ecker 2013, Johansson Gudjonsdottir 2019, Main 2002, Puopolo 2010, Towers 2002, Vergani 2002) | 347,112 | RR 0.73, 95% CI 0.61 to 0.89    Heterogeneity (I^2^) = 13%^a^ | Favours risk-based strategy | Low | a. Most of the studies were assessed to be at serious risk of bias using the ROBINS-I. b. Moderate statistical heterogeneity I^2^=33%, P = 0.18, and complete overlap in 95%-CI estimates of studies. c. Not applicable in this review. d. The 95%-CI is relatively narrow and excludes a RR of 1.0. Due to large sample size, grading down is unnecessary e. No apparent publication bias in funnel plot, but not enough studies to assess. | Similar to EOGBS  infection comparisons, any, risk-based and universal  strategies were all significantly associated with a  reduced risk of all EOS compared to no strategy |
| Panneflek 2024 | Risk-factor based screening strategy vs no screening strategy | Neonates | Non-GBS EOS | 6 studies  (Ecker 2013, Johansson Gudjonsdottir 2019, Main 2000, Puopolo 2010, Towers 2002, Vergani 2002) | 347,112 | RR 0.76, 95% CI 0.51 to 1.14    Heterogeneity (I^2^) = 69% | No harm or benefit | Very-low | a. Most of the studies were assessed to be at serious risk of bias using the ROBINS-I. b. Substantial statistical heterogeneity I^2^=69%, P = 0.006, but considerable overlap in 95%-CI estimates of studies. c. Not applicable in this review. d. The 95%-CI is wide and includes a RR of 1.0. Sample size is not sufficiently large to detect a precise effect. e. No apparent publication bias in funnel plot, but not enough studies to assess. |  |

**Abbreviations:** EOS: early-onset sepsis, EOS-GBS: early-onset sepsis Group B Streptococcus, NOS: Newcastle Ottawa Scale, NS: not significant, ROB: risk of bias, RR: risk ratio* or relative risk**, SR: systematic review

^a^ 13% figure extracted from Panneflek 2024 supplementary information forest plots. We note that this conflicts with data presented in the GRADE table (33%) and have contacted the review authors to check.

**GRADE Working Group grades of evidence**
High quality: Further research is very unlikely to change our confidence in the estimate of effect.
Moderate quality: Further research is likely to have an important impact on our confidence in the estimate of effect and may change the estimate.
Low quality: Further research is very likely to have an important impact on our confidence in the estimate of effect and is likely to change the estimate.
Very low quality: We are very uncertain about the estimate.

### File 10.4. Universal v risk-based: All EOS and non-GBS EOS infection

All EOS and non-GBS infections as reported by the included high-quality systematic reviews comparing universal screening strategies versus risk-based approaches

| **Review (Author, year)** | **Comparison** | **Population** | **Outcome** | **Number of studies (primary studies included in MA)** | **n (total)** | **Effect size (95% confidence interval)** | **Direction of effect** | **GRADE LEVEL (as reported by SR authors)** | **a. ROB, b. Inconsistency, c. Indirectness, d. Imprecision, e. Publication bias (Report downgrades applied by the SR authors)** | **Finding as reported by review authors (verbatim)** |
| --- | --- | --- | --- | --- | --- | --- | --- | --- | --- | --- |
| Li 2020 | Screening-based vs Risk-based strategy | Neonates | Total EOS | 4 studies (Edwards 2003, Bizzarro 2008, Puopolo 2010, Ecker 2013) | 187,994 | RR 0.78, 95% CI 0.62 to 0.98, (P value not reported)    Heterogeneity (I^2^) = 38% | Favours screening | No GRADE | NA | The pooled analysis showed that the incidence of total EOS for screening-based strategy was significantly lower than that for risk-based strategy |
| Li 2020 | Screening-based vs Risk-based strategy | Neonates | Non-GBS-EOS | 7 studies  (Main 2000, Reisner 2000, Vergani 2002, Edwards 2003, Angstetra 2007, Puopolo 2010, Ecker 2013) | 280,896 | RR 0.91, 95% CI 0.74 to 1.11, P = NS    Heterogeneity (I^2^) = 18% | No harm or benefit | No GRADE | NA | The pooled analysis indicated no significant difference in the incidence of non-GBS EOS  for screening-based strategy compared with risk-based strategy |
| Li 2020 | Screening-based vs Risk-based strategy | Neonates | *E-coli*-EOS | 4 studies (Edwards 2003, Bizzarro 2008, Puopolo 2010, Ecker 2013) | 187,994 | RR 0.98, 95% CI 0.69 to 1.40, P = NS    Heterogeneity (I^2^) = 0% | No harm or benefit | No GRADE | NA | The pooled analysis indicated no significant difference of *E. coli*-EOS between risk-based strategy and screening-based strategy |
| Panneflek 2024 | Universal screening strategy versus risk-based strategy | Neonates | All EOS | 6 studies  (Chan 2023, Ecker 2013, Edwards 2003, Hong 2019, Main 2002, Puopolo 2010) | 674,033 | RR 0.72, 95% CI 0.65 to 0.80^a^    Heterogeneity (I^2^) = 44% | Favours universal screening strategy | Low | a. Most of the studies were assessed to be at serious risk of bias using the ROBINS-I. b. Moderate statistical heterogeneity I^2^=44%, P<=0.11, and complete overlap in 95%-CI estimates of studies. c. Not applicable in this review. d. The 95%-CI is relatively narrow and excludes a RR of 1.0. Due to large sample size, grading down is unnecessary e. No apparent publication bias in funnel plot, but not enough studies to assess. |  |
| Panneflek 2024 | Universal screening strategy versus risk-factor based screening strategy | Neonates | Non-GBS EOS | 6 studies  (Chan 2023, Ecker 2013, Edwards 2003, Hong 2019, Main & Slagle 2002, Puopolo & Eichenwald 2010) | 674,033 | RR 0.93, 95% CI 0.82 to 1.05    Heterogeneity (I^2^) = 0% | No harm or benefit | Very-low | a. Most of the studies were assessed to be at serious risk of bias using the ROBINS-I. b. Unimportant statistical heterogeneity I^2^=0%, P = 0.46, and complete overlap in 95%-CI estimates of studies. c. Not applicable in this review. d. The 95%-CI is wide and includes a RR of 1.0. Sample size is not sufficiently large to detect a precise effect. e. No apparent publication bias in funnel plot, but not enough studies to assess. |  |

**Abbreviations**: EOS: early-onset sepsis, EOS-GBS: early-onset sepsis Group B Streptococcus, NOS: Newcastle Ottawa Scale, NS: not significant, ROB: risk of bias, RR: risk ratio* or relative risk**, SR: systematic review

^a^ Data extracted from Panneflek 2024 supplementary information forest plots. We note that this conflicts with data presented in the GRADE table (RR 0.46, 95% CI 0.36 to 0.60), 6 studies, I² = 44%) and have contacted the review authors to check.

**GRADE Working Group grades of evidence**
High quality: Further research is very unlikely to change our confidence in the estimate of effect.
Moderate quality: Further research is likely to have an important impact on our confidence in the estimate of effect and may change the estimate.
Low quality: Further research is very likely to have an important impact on our confidence in the estimate of effect and is likely to change the estimate.
Very low quality: We are very uncertain about the estimate.
