# Supplementary material for: Benefits and Harms of Antenatal/Intrapartum Screening for Maternal Group B Streptococcus and Use of Intrapartum Antibiotic Prophylaxis Versus Risk‐Based Protocols or No Intervention: A Rapid Review
Source: Acta Paediatr. 2026 Apr 30;115(8):1598–610. doi: 10.1111/apa.70568 (PMC13371836; doi:10.1111/apa.70568)
Supplement: Supplementary file 11 — Data S11: Antimicrobial resistance: Summary of meta‐analysis and GRADE judgements. [file APA-115-1598-s011.docx]

## Supplementary materials File 11. Antimicrobial resistance: summary of meta-analysis and GRADE judgements

### File 11.1. Universal versus risk-based: antimicrobial resistance

Antimicrobial resistance as reported by the included high-quality systematic reviews comparing universal screening strategies versus risk-based approaches

| **Review (Author, year)** | **Comparison** | **Population** | **Outcome** | **Number of studies (primary studies included in MA)** | **n (total)** | **Proportion of GBS resistant isolates** | **Direction of effect** | **GRADE LEVEL (as reported by SR authors)** | **a. ROB, b. Inconsistency, c. Indirectness, d. Imprecision, e. Publication bias (Report downgrades applied by the SR authors)** | **Finding as reported by review authors (verbatim)** |
| --- | --- | --- | --- | --- | --- | --- | --- | --- | --- | --- |
| Hasperhoven 2020 | Screening-based vs Risk-based strategy | Neonates | GBS isolates resistant to penicillin or ampicillin | 5 studies (Chen 2005, Edwards 2003, Main 2000, O’Sullivan 2019, Phares 2008) | NR | Weighted mean: 0% | NA | No GRADE | NA | None of the studies reported GBS resistance to penicillin or ampicillin |
| Hasperhoven 2020 | Screening-based vs Risk-based strategy | Neonates | GBS isolates resistant to Erythromycin | 4 studies (Chen 2005, Edwards 2003, O’Sullivan 2019, Phares 2008) | NR | Weighted mean: 19% (0 to 32%) | NA | No GRADE | NA | Resistance to erythromycin and clindamycin was reported by three studies * |
| Hasperhoven 2020 | Screening-based vs Risk-based strategy | Neonates | GBS isolates resistant to Clindamycin | 2 studies (O’Sullivan 2019, Phares 2008) | NR | Weighted mean: 16% (15 to 17%) | NA | No GRADE | NA | Resistance to erythromycin and clindamycin was reported by three studies* |

Abbreviations: EOS: early-onset sepsis, EOS-GBS: early-onset sepsis Group B Streptococcus, NOS: Newcastle Ottawa Scale, NS: not significant, ROB: risk of bias, SR: systematic review
*Hasperhoven Table S7 shows results for 4 and 2 studies (3 described in text)

### File 11.2. All strategies: antimicrobial resistance

Antimicrobial resistance as reported by the included high-quality systematic reviews with any strategy

| **Review (Author, year)** | **Comparison** | **Population** | **Outcome** | **Number of studies (primary studies included in MA)** | **n (total)** | **Proportion of GBS resistant isolates** | **Direction of effect** | **GRADE LEVEL (as reported by SR authors)** | **a. ROB, b. Inconsistency, c. Indirectness, d. Imprecision, e. Publication bias (Report downgrades applied by the SR authors)** | **Finding as reported by review authors (verbatim)** |
| --- | --- | --- | --- | --- | --- | --- | --- | --- | --- | --- |
| Panneflek 2024 | All strategies | Neonates | Antimicrobial resistance to penicillin or ampicillin in EOGBS isolates | 11 studies (Chen 2005, Ecker 2013, Edwards 2003, Freitas 2017, O'Sullivan 2019, Phares 2008, Sridhar 2014, Sutkin 2005, Trijbels-Smeulders 2006, van den Hoogen 2010, Wang 2023) | NR | Percentage of antimicrobial resistance: NR  Individual studies all 0% | NA | No GRADE | NA | In the 11 studies reporting on antimicrobial resistance of EOGBS isolates, there was no resistance to ampicillin, penicillin or other β-lactams |
| Panneflek 2024 | All strategies | Neonates | Antimicrobial resistance to erythromycin in EOGBS isolates | 5 studies (Chen 2005, O'Sullivan 2019, Phares 2008, Sutkin 2005, Wang 2023) | NR | Percentage of antimicrobial resistance: NR  Individual studies reported 0% to 61% | NA | No GRADE | NA | In the 11 studies reporting on antimicrobial resistance of EOGBS isolates, there was no resistance to ampicillin, penicillin or other β-lactams but varying resistance to antimicrobials administered in the presence of a penicillin allergy, such as erythromycin and clindamycin, but not vancomycin |
| Panneflek 2024 | All strategies | Neonates | Antimicrobial resistance to clindamycin in EOGBS isolates | 5 studies (Chen 2005, O'Sullivan 2019, Phares 2008, Sutkin 2005, Wang 2023) | NR | Percentage of antimicrobial resistance: NR  Individual studies reported 0% to 32% | NA | No GRADE | NA | In the 11 studies reporting on antimicrobial resistance of EOGBS isolates, there was no resistance to ampicillin, penicillin or other β-lactams but varying resistance to antimicrobials administered in the presence of a penicillin allergy, such as erythromycin and clindamycin, but not vancomycin |
| Panneflek 2024 | All strategies | Neonates | Antimicrobial resistance to vancomycin in EOGBS isolates | 3 studies (Chen 2005, Freitas 2017, Phares 2008) | NR | Percentage of antimicrobial resistance: NR  Individual studies reported 0% | NA | No GRADE | NA | In the 11 studies reporting on antimicrobial resistance of EOGBS isolates, there was no resistance to ampicillin, penicillin or other β-lactams but varying resistance to antimicrobials administered in the presence of a penicillin allergy, such as erythromycin and clindamycin, but not vancomycin |

Abbreviations: EOS: early-onset sepsis, EOS-GBS: early-onset sepsis Group B Streptococcus, NOS: Newcastle Ottawa Scale, NS: not significant, ROB: risk of bias, SR: systematic review

### File 11.3. Universal versus risk-based: *E coli* resistance

*E coli* resistance as reported by the included high-quality systematic reviews comparing universal screening strategies versus risk-based approaches

| **Review (Author, year)** | **Comparison** | **Population** | **Outcome** | **Number of studies (primary studies included in MA)** | **n (total)** | **Effect size (95% confidence interval)** | **Direction of effect** | **GRADE LEVEL (as reported by SR authors)** | **a. ROB, b. Inconsistency, c. Indirectness, d. Imprecision, e. Publication bias (Report downgrades applied by the SR authors)** | **Finding as reported by review authors (verbatim)** |
| --- | --- | --- | --- | --- | --- | --- | --- | --- | --- | --- |
| Li 2020 | Screening-based vs Risk-based strategy | Neonates | Ampicillin-resistant *E. coli*-EOS | 3 studies (Bizzarro 2008, Puopolo 2010, Ecker 2013) | 170,807 | RR 1.28, 95% CI 0.74 to 2.21, P = NS    Heterogeneity (I^2^) = 0% | No harm or benefit | No GRADE | NA | The pooled analysis showed that the incidence of ampicillin resistant *E. coli*-EOS was higher in screening-based group compared with risk-based group, but the difference did not reach statistical significance |

Abbreviations: EOS: early-onset sepsis, EOS-GBS: early-onset sepsis Group B Streptococcus, NOS: Newcastle Ottawa Scale, NS: not significant, ROB: risk of bias, SR: systematic review
