# Supplementary material for: Benefits and Harms of Antenatal/Intrapartum Screening for Maternal Group B Streptococcus and Use of Intrapartum Antibiotic Prophylaxis Versus Risk‐Based Protocols or No Intervention: A Rapid Review
Source: Acta Paediatr. 2026 Apr 30;115(8):1598–610. doi: 10.1111/apa.70568 (PMC13371836; doi:10.1111/apa.70568)
Supplement: Supplementary file 12 — Data S12: IAP exposure: Summary of meta‐analysis and GRADE judgements. [file APA-115-1598-s021.docx]

## Supplementary materials File 12 (S12). IAP exposure: summary of meta-analysis and GRADE judgements

### File 12.1. Universal versus risk-based: IAP exposure

Exposure of antibiotics, or antibiotics administered to, women during labour in universal screening strategies compared to risk-based approaches as reported by the included high-quality systematic reviews

| **Review (Author, year)** | **Comparison** | **Population** | **Outcome** | **Number of studies (primary studies included in MA)** | **n (total)** | **Effect size (95% confidence interval)** | **Direction of effect** | **GRADE LEVEL (as reported by SR authors)** | **a. ROB, b. Inconsistency, c. Indirectness, d. Imprecision, e. Publication bias (Report downgrades applied by the SR authors)** | **Finding as reported by review authors (verbatim)** |
| --- | --- | --- | --- | --- | --- | --- | --- | --- | --- | --- |
| Hasperhoven 2020 | Universal screening strategy versus risk-based strategy | Pregnant women | IAP administration | 4 studies (Main 2000, Schrag 2002, Vergani 2002, Yucesoy 2004) | NR | NR  Weighted mean IAP exposure: 31% in universal screening vs 29% in risk based | No harm or benefit | NR | NA | In this meta-analysis, the overall exposure to antibiotics does not appear to differ greatly between the two protocols |
| Panneflek 2024 | Universal screening strategy versus risk-based strategy | Pregnant women | IAP administration | 9 studies  (Bjorklund 2017, Coco 2002, Hafner1998, Hong 2019, Schrag 2002, Schuchat 2002, Wang 2023, Youden 2005, Yucesoy 2004) | 514,023 | RR 1.29, 95% CI 0.95 to 1.75    Heterogeneity (I^2^) = 99% | No harm or benefit | Very-low | a. Most of the studies were assessed to be at moderate risk of bias using the ROBINS-I with regard to IAP administration. b. Considerable statistical heterogeneity I^2^=99%, P<0.001, and some overlap in 95%-CI estimates of studies. c. Not applicable in this review. d. The 95%-CI is wide and includes a RR of 1.0. Sample size is not sufficiently large to detect a precise effect. e. Appearance of publication bias in funnel plot, but not enough studies to assess. | No significant difference in the rate of IAP  administration was found |

Abbreviations: EOS: early-onset sepsis, EOS-GBS: early-onset sepsis Group B Streptococcus, IAP: intrapartum antibiotic anaphylaxis, NOS: Newcastle Ottawa Scale, NS: not significant, ROB: risk of bias, RR: risk ratio* or relative risk**, SR: systematic review

**GRADE Working Group grades of evidence**
High quality: Further research is very unlikely to change our confidence in the estimate of effect.
Moderate quality: Further research is likely to have an important impact on our confidence in the estimate of effect and may change the estimate.
Low quality: Further research is very likely to have an important impact on our confidence in the estimate of effect and is likely to change the estimate.
Very low quality: We are very uncertain about the estimate.

### File 12.2. Any strategy: IAP exposure

Exposure of antibiotics, or antibiotics administered to, women during labour by strategy as reported by included high quality systematic reviews

| **Review (Author, year)** | **Number of studies (primary studies included in MA)** | **Percentage of in pregnant women (IAP exposure) receiving IAP (%, 95% confidence interval)** | | | | **GRADE LEVEL (as reported by SR authors)** | **Finding as reported by review authors (verbatim)** |
| --- | --- | --- | --- | --- | --- | --- | --- |
|  |  | **No strategy** | **Any strategy** | **Risk based** | **Universal screening** |  |  |
| Panneflek 2024 | 3 studies  (Katz 1999, levine 1999, Uy 2002) | 8 (3-17)%  Heterogeneity (I^2^) = 100% |  |  |  | NA | Pooled IAP administration rate during periods with no strategy more than doubled with any strategy |
| Panneflek 2024 | 16 studies  (Bjorklund 2017, Coco 2002, Davies 2001, Hafner1998, Hong 2019, Katz 1999, Locksmith 1999, Levine 1999, Schrag 2002, Schuchat 2002, Uy 2002, van Dyke 2009, Vergani 2002, Wang 2023, Youden 2005, Yucesoy 2004) |  | 19 (16-22)%  Heterogeneity (I^2^) = 100% |  |  | NA |  |
| Panneflek 2024 | 11 studies  (Bjorklund 2017, Coco 2002, Hafner1998, Hong 2019, Schrag 2002, Schuchat 2002, Uy 2002, Vergani 2002, Wang 2023, Youden 2005, Yucesoy 2004) |  |  | 16 (12-20)%  Heterogeneity (I^2^) = 97% |  | Very-low | No significant difference in the rate of IAP administration was found |
| Panneflek 2024 | 12 studies  (Bjorklund 2017, Coco 2002, Davies 2001, Hafner1998, Hong 2019, Locksmith 1999, Schrag 2002, Schuchat 2002, van Dyke 2009, Wang 2023, Youden 2005, Yucesoy 2004) |  |  |  | 21 (18-24)%  Heterogeneity (I^2^) = 98% |  |  |

**Abbreviations:** IAP: intrapartum antibiotic prophylaxis, MA: meta-analysis, NA: not assessed; SR: systematic review
