# Supplementary material for: Benefits and Harms of Antenatal/Intrapartum Screening for Maternal Group B Streptococcus and Use of Intrapartum Antibiotic Prophylaxis Versus Risk‐Based Protocols or No Intervention: A Rapid Review
Source: Acta Paediatr. 2026 Apr 30;115(8):1598–610. doi: 10.1111/apa.70568 (PMC13371836; doi:10.1111/apa.70568)
Supplement: Supplementary file 13 — Data S13: Summary of missed cases. [file APA-115-1598-s017.docx]

## Supplementary materials File 13 (S13). Missed cases: summary from reviews

Missed cases refer to EOGBS cases that were not indicated by positive maternal GBS screening or detection of risk factors and therefore no IAP was administered.

### File 13.1. Missed cases

Table showing the proportion of GBS positive infants born to mothers with no indications for IAP administration reported by included high-quality systematic reviews

| **Review (author/year)** | **Primary study Number of studies (primary studies included in MA)** | **n (total)** | **Proportion of GBS infants born to mothers without risk factors (risk based period)**  **Weighted mean** | **Proportion of GBS infants born to mothers with negative screening result (screening period)**  **Weighted mean** |
| --- | --- | --- | --- | --- |
| Hasperhoven 2020 | 13 studies  (Darlow 2016, Eisenberg 2005, Gilson 2000, Gopal Rao 2017, Håkansson 2017, Hung 2018, Ma 2018, Main 2000, O'Sullivan 2019, Phares 2008, Schrag 2002, Vergani 2002, Yücesoy 2004) | NR | 41.3% | 24.2% |

**Abbreviations:** GBS: Group B Streptococcus, IAP: intrapartum antibiotic prophylaxis, MA: meta-analysis

### File 14.2. Missed cases at primary study level

Table showing the proportion of GBS positive infants born to mothers with no indications for IAP administration reported by primary study

| **Review** | **Study** | **Risk-based period: of the**  **GBS infants, how many**  **were born to mothers**  **without risk factors?** | **Screening period: of the GBS**  **infants, how many were**  **born to mothers with**  **negative screening result?** |
| --- | --- | --- | --- |
| Hasperhoven 2020 | Angstetra 2007 |  |  |
| Hasperhoven 2020 | Bekker 2014 |  |  |
| Hasperhoven 2020 | Chen 2005 |  |  |
| Hasperhoven 2020 | Darlow 2016 | 45% |  |
| Hasperhoven 2020 | Edwards 2003 |  |  |
| Hasperhoven 2020 | Eisenberg 2005 | 58% | 40% |
| Hasperhoven 2020 | Gilson 2000 | 75% | 0% |
| Hasperhoven 2020 | Gopal Rao 2017 | 11% | 0% |
| Hasperhoven 2020 | Håkansson 2017 | 43% |  |
| Hasperhoven 2020 | Hung 2018 |  | 58% |
| Hasperhoven 2020 | Ma 2018 |  | 41.3% |
| Hasperhoven 2020 | Main 2000 | 46% * |  |
| Hasperhoven 2020 | O'Sullivan 2019 | 40% (lowest) |  |
| Hasperhoven 2020 | Phares 2008 |  | 24% |
| Hasperhoven 2020 | Schrag 2002 | 62% ** |  |
| Hasperhoven 2020 | Vergani 2002 | 75% | 17% |
| Hasperhoven 2020 | Yücesoy 2004 | 66% |  |
| **Weighted mean** |  | **41.3%** | **24.2%** |

**Abbreviations:** GBS: Group B Streptococcus, IAP: intrapartum antibiotic prophylaxis, MA: meta-analysis
Three groups of cases are included based on policy and maternal indication for prophylaxis.
*only cases in term newborns are included ** both cases under screening and risk-based protocols taken together
