# Supplementary material for: Benefits and Harms of Antenatal/Intrapartum Screening for Maternal Group B Streptococcus and Use of Intrapartum Antibiotic Prophylaxis Versus Risk‐Based Protocols or No Intervention: A Rapid Review
Source: Acta Paediatr. 2026 Apr 30;115(8):1598–610. doi: 10.1111/apa.70568 (PMC13371836; doi:10.1111/apa.70568)
Supplement: Supplementary file 14 — Data S14: Key characteristics: Primary study level. [file APA-115-1598-s020.docx]

## Supplementary materials File 14. Key characteristics at primary study level

Key characteristics of primary studies as reported by the systematic reviews or newly identified from additional searches. When a study was reported by more than one systematic review, data were extracted from each source.

| **Review** | **Study ID** | **Study design** | **Controls / time analysis** | **Country** | **Participants** | **Setting/data source** | **EOGBS disease/ infection definition** | **Screening-based / universal strategy 1 criteria for IAP** | **IAP criteria: universal strategy 2** | **Risk-based criteria for IAP** | **IAP criteria: other strategies** | **Antibiotic agent** |
| --- | --- | --- | --- | --- | --- | --- | --- | --- | --- | --- | --- | --- |
| Li 2020 (1) | Abdelmaaboud 2011 (2) | Retrospective cohort | H | - | Women and their live births | Microbiology laboratory’s computerized database between January 2003 and December 2009 | EOS was identified through querying of any blood or cerebrospinal fluid culture positive for GBS obtained from neonates before 72 h of age. | 2006–2009. Maternal screening for GBS was done for all pregnant women attending outpatient clinic and emergency department by taking rectovaginal swabs and urine culture at 35–37 weeks of gestation. IAP was offered if culture was positive. (limited information) | - | 2003–2006. All high-risk cases at time of presentation (preterm labor, intrapartum fever of 38°C or above, clinical chorioamnionitis or GBS colonization) were offered IAP. | - | Ampicillin |
| Panneflek 2024 (3) | Abdelmaaboud 2011 (2) | Retrospective observational | H | Qatar | - | Principal public hospital in State | GBS: <72 h blood or cerebrospinal fluid (CSF) | IAP to: i) carriers of rectovaginal and urine GBS culture at 35–37 weeks’ gestation, and ii) carriers of GBS culture at presentation for all high-risk cases (preterm labour (PTL), prolonged rupture of membranes (PROM), intrapartum fever (fever), clinical chorioamnionitis or GBS colonisation). | - | IAP to: PTL, PROM, fever, and a previous infant with GBS disease. | - | Ampicillin initially 2 g IV and 1 g every 6 h. Penicillin allergy, 900 mg clindamycin every 8 h |
| Panneflek 2024 (3) | Al Luhidan 2019 (4) | Retrospective cohort | H | Saudi Arabia | - | Tertiary care hospital | GBS: <7 days in sterile body site (blood, CSF, suprapubic catheter and lungs). | CDC 2002: IAP to: i) carriers of rectovaginal GBS colonisation at 35–37 weeks’ gestation, ii) previous baby with invasive GBS infection, iii) GBS bacteriuria, and iv) if unknown carrier state, presence of PTL, PROM and fever. | - | RCOG 2012: IAP to: PTL, PROM, fever, previous infant with invasive GBS infection, and GBS bacteriuria | - | CDC 2002: Penicillin G 5 million units IV initially and 2.5–3 million units every 4 h. Ampicillin as alternative. Penicillin allergy not at high risk of anaphylaxis, cefazolin 2 g IV and 1 g every 8 h. Penicillin allergy high risk of anaphylaxis and susceptibility available, clindamycin 900 mg IV every 8 h, or erythromycin 500 IV every 6 h. If susceptibility not possible or unknown or isolates resistant to clindamycin or erythromycin, vancomycin 1 g IV every 12 h. RCOG 2012: Penicillin G 3 g IV initially and 1.5 g every 4 h. If allergy not risk of anaphylaxis, then cephalosporin (e.g. cefuroxime 1.5 g IV initially and 750 mg every 8 h). If allergy high risk of anaphylaxis, vancomycin 1 g every 12 h. |
| Panneflek 2024 (3) | Alarcon 2004 (5) | Retrospective cohort | H | Spain | - | University Tertiary Public Hospital | - | CDC 1996. | - | CDC 1996. | - | CDC 1996. |
| Hasperhoven 2020 (6) | Angstetra 2007 (7) | Retrospective cohort | H | Australia | - | One tertiary obstetric unit | Blood culture ++ and necessity for admission to N(I)CU with ABs + ventilation <7 days. | 34–37 weeks. Culture +ve or risk factor: previous GBS, GBS bacteriuria, preterm labour <37 weeks, temp.>38°C, prolonged ROM >18 h (if GBS status unknown) -> IAP | - | NI | - | Benzylpenicillin IV 1.2 g- 600 mg/h OR clindamycin IV 600 mg/8 h OR cephalothin IV 2 g - 1 g/h |
| Panneflek 2024 (3) | Angstetra 2007 (7) | Prospective cohort | H | Australia | - | Tertiary obstetric unit | GBS <7 days in blood or CSF, admission to neonatal unit for treatment with antibiotics and ventilation, and signs of sepsis. | - | - | NI | Rectovaginal swab at 34–37 weeks gestation, IAP to i) carriers of rectovaginal GBS colonisation at 34–37 weeks’ gestation, ii) PTL, fever previous infant with GBS infection, and GBS bacteriuria regardless of GBS status, and iii) if unknown carrier state, presence of PROM. | Penicillin G 1.2 g IV initially then 600 mg 4 h. Penicillin-sensitive individuals either cephalothin 2 g IV initially and 1 g every 6 h or clindamycin 600 mg IV every 8 h. |
| Li 2020 (1) | Angstetra 2007 (7) | Prospective cohort | H | - | Women and their live births | DOS-based Clinical Reporting System (before 2000), and the OBSTET and OBSTETRIX databases (after 2000) from January 1, 1994, to June 30, 2006 | GBS-EOS was defined as any blood or CSF culture positive for GBS obtained from an infant less than 7 d. | 2004–2006. All women offered rectovaginal swab for GBS detection at 34-37 weeks gestation. IAP was offered if culture was positive. (limited information) | - | 1994–2002. IAP offered if previous infant with GBS-EOS, GBS bacteriuria in current pregnancy, preterm labor of less than 37 weeks spontaneous or induced (unless known GBS status within last 2 weeks), intrapartum fever 38°C or more on 2 occasions 2 h apart regardless of GBS status and prolonged rupture of membranes of 18 h or more where GBS status is unknown. | - | Penicillin G |
| Panneflek 2024 (3) | Bauserman 2013 (8) | Retrospective cohort | H | USA | - | 322 NICUs managed by the Paediatric Medical Group | GBS: < 3 days in blood, urine or CSF | CDC 2002 | - | - | - | CDC 2002 |
| Hasperhoven 2020 (6) | Bekker 2014 (9) | Surveillance (retrospective) | H | NL | - | - | Positive blood or CSF culture <7 days | NA | - | Previous GBS child or bacteriuria -> IAP. Confirmed GBS or threat preterm -> consider IAP. PROM + threat preterm -> consider GBS test presentation + positive blood or CSF culture + laboratory indication of sepsis (CRP or leucopenia etc.). Only cases <48 h are reported. | - | Penicillin/ amoxicillin |
| Panneflek 2024 (3) | Bekker 2014 (9) | Retrospective nationwide surveillance | H | NL | - | The Netherlands Reference Laboratory for Bacterial Meningitis | GBS: <7 days in blood or CSF. | - | - | - | Dutch guidelines 1999: IAP to: i) previous infant with GBS infection, GBS bacteriuria or urinary tract infection and fever, and ii) GBS colonisation and PTL or PROM. | Dutch guidelines 1999: Penicillin G 5 million IU IV initially and 2.5 million IU every 4 h or 2 g of amoxicillin initially and 1 g every 4 h. Alternatively amoxicillin or ampicillin 2 g IV initially and 1 g every 4 h. Penicillin allergy, clindamycin 900 mg every 8 h. Alternatively, erythromycin 500 mg every 6 h. |
| Li 2020 (1) | Bizzarro 2005 (10) | Retrospective cohort | H | - | Women and their new-borns | Microbiology laboratory between January 1, 1979, and December 31, 2006 | EOS was defined as positive blood culture within 72 h of life. | 1997–2006. Limited information. Assume screening-based protocol as per the hospital’s predominant IAP practices. | - | 1993–1996. Limited information. Assume risk-based protocol as per the hospital’s predominant IAP practices | - | NA |
| Panneflek 2024 (3) | Björklund 2017 (11) | Retrospective cohort | H | Finland | - | Public financed tertiary delivery unity | GBS: <3 days in clinical records databased with ICD code for GBS sepsis. | IAP to: i) carriers of rectovaginal GBS polymerase chain reaction at admission, and ii) if carrier state unknown on presence of risk-factors. | - | IAP to: PROM, GBS bacteriuria, GBS colonisation, and previous infant with EOGBS. | - | Penicillin G 5 million IU IV initially and 2.5 million IU every 4 h. Penicillin allergy, cefuroxime 1.5 g IV initially and 750 mg every 8 h or clindamycin 900 mg IV every 8 h. |
| Li 2020 (1) | Bjorklund 2017 (11) | Retrospective cohort | C | - | All women and their term vaginally delivered live births | Kätilöopisto Maternity Hospital between January 1, 2014, and November 12, 2014 | New-borns under 3 d of age with proven GBS-EOD were identified | 27 May to 12 November 2014. On admission to hospital for delivery, a double-swab was taken from every woman during the initial clinical examination. Intrapartum vaginal/rectal PCR screening for GBS infection was performed. The midwives then initiated IAP for women who either screened positive, or if there was no result of the vaginal/rectal sample (e.g., PCR invalid or error) and the women presented risk factors. | - | 1 January to 26 May 2014. Prophylactic antibiotics were given to mothers with premature rupture of membranes for more than 18 h, history of GBS-positive vaginal or urine culture earlier during the pregnancy, or a previous child with culture-positive GBS-EOD. | - | Penicillin G |
| Panneflek 2024 (3) | Björnsdóttir 2019 (11) | Retrospective and descriptive case study | H | Iceland | - | - | GBS: <7 days. | - | - | IAP to: PTL, fever, PROM, and positive GBS cultures in late pregnancy or earlier deliveries. | - | NI |
| Panneflek 2024 (3) | Brozanski 2000 (12) | Retrospective observational | H | USA | - | Tertiary referral hospital | GBS: <48 h in blood or CSF. | CDC 1996 (similar to CDC 2002). | - | - | AAP 1992 and no strategy IAP to: i) carriers of rectovaginal GBS colonisation at 26–28 weeks’ gestation or at admission with PTL, (premature) PROM, fever, multiple births, and ii) previous infant with invasive GBS infection. | Ampicillin, penicillin, clindamycin, or erythromycin. |
| Panneflek 2024 (3) | Chan 2023 (13) | Retrospective cohort | H | China | - | Eight public hospitals and 31 health centres | <8 days in blood or CSF. | IAP to: i) carriers of rectovaginal GBS colonisation at 35–37 weeks’ gestation, and ii) carrier state known before 35 weeks’ gestation, GBS bacteriuria or history previous infant affected by GBS disease. | - | IAP to: previous infant with invasive GBS disease, PTL, GBS colonisation, and GBS bacteriuria. | - | Penicillin G 5 million IU IV initially and 2.5 million IU every 4 h. Penicillin allergy, erythromycin, clindamycin, or vancomycin according to sensitivity. |
| Panneflek 2024 (3) | Chen 2001 (14) | Retrospective cohort | H | USA | - | Tertiary care referral centre | - | - | - | IAP to: PTL, PROM and fever | - | Ampicillin. Penicillin allergy, clindamycin |
| Panneflek 2024 (3) | Chen 2005 (15) | Retrospective cohort | H | USA | - | Tertiary care referral centre | GBS: <7 days in blood | IAP to: carriers of rectovaginal GBS screening at 35-37 weeks’ gestation | - | IAP to: PTL, PROM and fever. | - | Ampicillin in risk-factor strategy Penicillin allergy, clindamycin. Penicillin G in universal strategy. Penicillin allergy, erythromycin or clindamycin. |
| Hasperhoven 2020 (6) | Chen 2005 (15) | Retrospective cohort | H | USA | - | One tertiary care centre | Positive blood culture <7 days | 35–37 weeks, vaginal and rectal positive culture ->IAP | - | Preterm birth (threat), fever (non-specified), prolonged rupture of membranes ->IAP | - | Ampicillin or clindamycin; later penicillin G OR erythromycin |
| Li 2020 (1) | Chen 2005 (15) | Retrospective cohort | H | - | Women and their infants | Brigham and Women’s Hospital from January 1, 1990, to December 31, 2002 | Early onset GBS sepsis was determined if GBS was isolated from a blood culture within the first 7 d of life | 1997–2002. IAP was given to women in labor who screened positive for GBS colonization by vaginal and rectal screening culture obtained at 35–37 weeks of gestation. | - | 1993–1996. IAP was given to women in labor who had risk factors for GBS transmission (e.g., preterm delivery, intrapartum fever, prolonged rupture of membranes). | - | Ampicillin or penicillin G |
| Panneflek 2024 (3) | Cho 2019 (16) | Retrospective cohort | H | Taiwan | - | Tertiary obstetric unit | GBS: <7 days in sterile site (blood, CSF or urine) with signs of clinical disease | CDC 2010 (similar to CDC 2002). | - | -- | - | Penicillin, ampicillin, or cefazolin. |
| Panneflek 2024 (3) | Clemens 2002 (16) | Retrospective cohort | H | USA | - | Nonacademically affiliated community hospital | EOGBS | CDC 1996 (similar to CDC 2002). | - | - | - | - |
| Panneflek 2024 (3) | Coco 2002 (17) | Retrospective cohort | H | USA | - | Family practice residency maternal service | - | IAP to: carriers of rectovaginal GBS culture after 35 weeks’ gestation | - | IAP to: PROM and fever. | - | NI |
| Newly identified primary study | Daniels 2022 (18, 19) | Cluster RCT | C | England, UK | Pregnant women with risk factors | Twenty UK maternity units | Culture to detect the presence of GBS/ microbiological culture of blood or cerebrospinal fluid, both 72hrs and <7 days mentioned | - | - | Previous baby with EOGBS or LOGBS; GBS bacteriuria; GBS maternal colonisation +ve; suspected, diagnosed or established preterm labour (< 37 weeks); maternal pyrexia (≥ 38 °C) | Rapid PCR intrapartum test: previous baby with EOGBS or LOGBS; GBS bacteriuria; GBS maternal colonisation +ve; suspected, diagnosed or established preterm labour (< 37 weeks); maternal pyrexia (≥ 38 °C) | 3 g benzylpenicillin at onset of labour, half that dose at 4-hourly intervals until birth. If allergic to penicillin: cephalosporin, history of reactions to beta-lactams, vancomycin. (HTA table 33 for mean dose given) |
| Hasperhoven 2020 (6) | Darlow 2016 (20) | Prospective and retrospective surveillance | H | New Zealand | - | New Zealand Paediatric Surveillance Unit (all hospitals) | Clinical | NA | - | Previous GBS child, bacteriuria, preterm, temp. >38°C, membrane rupture> 18 h -> IAP | - | NI |
| Panneflek 2024 (3) | Darlow 2016 (20) | Prospective and retrospective surveillance | H | New Zealand | - | New Zealand Paediatric Surveillance Unit (all hospitals) | GBS: <3 days in blood, CSF or pleura with clinical and laboratory evidence of sepsis | - | - | IAP to: PTL, PROM, fever, previous infant with GBS infection, and GBS bacteriuria. | - | Penicillin or amoxycillin |
| Panneflek 2024 (3) | Davis 2001 (21) | Retrospective cohort | H | USA | - | Non-profit group and network health maintenance organisation with data from 2 hospitals | - | CDC 1996 | - | - | AAP 1992 | Penicillin G |
| Panneflek 2024 (3) | Eberly & Rajnik 2009 (22) | Retrospective cohort | H | USA | - | Hospital within the Department of Defence | GBS: <7 days with possession of diagnosis group codes and lack of other diagnosis group codes that refer to non-GBS bacteria. | CDC 2002 | - | - | CDC 1996 IAP to: i) PTL, PROM and fever, ii) carriers of rectovaginal GBS screening at 35–37 weeks’ gestation, iii) if unknown carrier state, presence of risk-factors, iv) previous infant with GBS infection, and v) GBS bacteriuria. | CDC 1996: Penicillin G 5 million IU IV initially and 2.5 million IU IV every 4 hours. Alternatively ampicillin 2g IV initially and 1 g every 4 hours. Penicillin allergy not at high risk of anaphylaxis, cefazolin 2 g IV initially and 1 g every 8 h. Penicillin allergy at high risk of anaphylaxis and susceptibility available, clindamycin 900 mg IV every 8 h, or erythromycin 500 IV every 6 h. If susceptibility not possible or unknown or isolates resistant to clindamycin or erythromycin, vancomycin 1 g IV every 12 h. CDC 2002. |
| Panneflek 2024 (3) | Ecker 2013 (23) | Retrospective cohort | H | USA | - | Regional tertiary care centre | GBS1: Non-GBS2: All3: ≤7 days in blood, urine or CSF with appropriate treatment | CDC 2002 | - | NI | - | Penicillin or ampicillin. |
| Li 2020 (1) | Ecker 2013 (23) | Retrospective cohort | H | - | - | Data collected between January 1, 1990, and December 31,2007, obtained from the hospital’s microbiology laboratory database | EOS was identified if any neonate had a positive culture at 7 d of age or younger. | 2003–2007. Limited information. Assumed screening-based protocol as per the hospital’s predominant IAP practices. | - | 1996–2002. Limited information. Assumed risk-based protocol as per the hospital’s predominant IAP practices. | - | Ampicillin or penicillin |
| Hasperhoven 2020 (6) | Edwards 2003 (24) | Retrospective cohort | H | US (Florida) | - | One general hospital | Positive blood culture <7 days | CDC guidelines of 1996 | - | NI | - | Ampicillin until 1995, then penicillin |
| Panneflek 2024 (3) | Edwards 2003 (24) | Retrospective cohort | H | USA | - | General hospital | GBS: <7 days in blood. | CDC 1996 | - | - | - | Ampicillin prior to March 1995 and penicillin thereafter. |
| Li 2020 (1) | Edwards 2003 (24) | Retrospective cohort study | H | - | Women and their live-born infants | Shands Hospital, University of Florida | EOS was defined as a positive blood culture during the first 7 d of life. | 1996–2000. Limited information. Assumed culture-based protocol as per CDC guidelines. | - | 1993–1996. Limited information. Assumed risk-based protocol as per CDC guidelines. | - | Ampicillin or penicillin |
| Panneflek 2024 (3) | Eisenberg 2005 (25) | Retrospective cohort study | C | USA | - | All acute care hospitals in four major counties of Tennessee | GBS: <7 days in blood or CSF. | IAP to: carriers of GBS culture at least 2 days before delivery. | - | IAP to: no GBS culture <2 days and PTL, PROM, fever, GBS bacteriuria, or previous infant with invasive GBS disease. | - | NI |
| Hasperhoven 2020 (6) | Eisenberg 2005 (25) | Retrospective cohort study | C | USA | - | All acute care hospitals in four major counties of Tennessee | Positive blood or CSF culture <7 days | CDC guidelines (retrospective selection: found any GBS status in record, taken at least 2 days before birth: screening group) | - | Risk group = not screened. Preterm <37, PROM> 18 h, temp. >38°C, GBS bacteriuria, previous GBS | - | NI |
| Li 2020 (1) | Eisenberg 2005 (25) | Retrospective cohort | C | - | Women and their live births | - | EOS was defined by a positive culture of blood or cerebrospinal fluid from a new-born younger than 7 d old. | 1998–1999. Women were assigned to screening cohort according to any documentation in maternal records of a GBS culture performed at least 2 d before delivery. IAP for vaginal and rectal cultures at 35–37 weeks (limited information). | - | 1998–1999. Women excluded by screening group were assigned to risk-based cohort. Trained abstractors reviewed records for GBS risk factors including delivery less than 37 weeks, rupture of membranes 18 h or more, temperature 38°C or above, GBS bacteriuria during this pregnancy and previous GBS-EOS infant. IAP offered if any risk factor was observed. | - | NA |
| Panneflek 2024 (3) | El Helali 2019 (26) | Retrospective cohort | H | France | - | General hospital | GBS: <7 days in blood or CSF with clinical signs or biological abnormalities consistent with sepsis | IAP to: i) carriers of rectovaginal GBS culture at 35-37 weeks’ gestation, ii) GBS bacteriuria, iii) previous infant with EOGBS, and iv) if unknown carrier state, PTL, PROM and fever | IAP to: i) carriers of intrapartum rectovaginal GBS PCR, ii) GBS bacteriuria, iii) previous infant with EOGBS, and iv) if unknown carrier state, PTL, PROM and fever. | - | - | Penicillin G 5 million IU IV initially and 2.5 million IU every 4 h. If penicillin allergy, clindamycin or vancomycin, depending susceptibility. |
| Panneflek 2024 (3) | Factor 1998 (27) | Retrospective cohort study | H | USA | - | Non-profit, tertiary care hospital | GBS: <7 days in blood or CSF. | - | - | ACOG 1992: IAP to: PTL, PROM, or fever. | - | Ampicillin, cefazolin, cefoxitin, vancomycin, cephalothin, erythromycin, and oxacillin |
| Panneflek 2024 (3) | Garland 1991 (28) | Retrospective observational study | C | Australia | - | Public teaching hospital | EOGBS: in blood, CSF, surface swabs or urine | IAP to: carriers vaginal GBS culture at 32 weeks’ gestation. | - | - | - | Penicillin 1 million IU IV every 6 h. Penicillin allergy, erythromycin 500 mg every 6 h. |
| Panneflek 2024 (3) | Gibbs 1994 (29) | Retrospective cohort study | H | USA | - | University hospital | EOGBS sepsis: | IAP to: i) carriers of rectovaginal GBS culture at 26–28 weeks’ gestation, ii) PTL, (premature) PROM, fever, chorioamnionitis with negative carrier status that turned positive, iii) previous infant with EOGBS infection, and iv) GBS bacteriuria. | - | - | - | Ampicillin 2 g IV initially and 1 g every 4 h. Penicillin allergy, erythromycin 500 mg every 6 h. |
| Hasperhoven 2020 (6) | Gilson 2000 (30) | Retrospective cohort study | C | US (New Mexico) | - | One hospital | Positive culture from blood, CSF or other fluid. | Women with known GBS + status, and unknown status -> risk factors | - | Rom> 18 h, temp. >38 °C, GBS bacteriuria, previous GBS (NB: preterm infants excluded) | - | Ampicillin 2 g + 2 g/6 h IV before 1995; Penicillin G IV 5 ml units + 2.5 ml units/4 h |
| Panneflek 2024 (3) | Gilson 2000 (30) | Retrospective cohort study | C | USA | - | Academic medical centre | GBS: <7 days in blood, CSF, urine or suprapubic aspiration. | IAP to: i) carriers of GBS centre colonisation at 35–37 weeks’ gestation, and ii) if unknown carrier state, presence of risk-factors. | - | IAP to: PTL, fever, PROM, and previous infant with GBS infection. | - | Before March 1995, ampicillin 2 g IV every 6 h. After March 1995, penicillin G 5 million IU initially and 2.5 million IU every 4 h. Penicillin allergy, clindamycin 900 mg every 8 h. |
| Li 2020 (1) | Gilson 2000 (30) | Retrospective cohort | C | - | Women and their live term infants | University of New Mexico Hospital | EOS was determined through the isolation of GBS from a normally sterile central site within the first 7 d of life | 1994–1996. Women between 35-37 weeks were received 2 swabs combined in transport medium. IAP was offered if culture was positive (limited information). | - | 1994–1996. IAP was given to all GBS positive women and term patients with following risk factors: maternal fever 38°C or above, prolonged rupture of membranes (18 h or more) and history of prior GBS affected infant. | - | Ampicillin or penicillin G |
| Hasperhoven 2020 (6) | Gopal Rao 2017 (31) | Retrospective observational study | H | London, UK | - | One general hospital | Positive blood or CSF culture <7 days | Screening offered to all women in the population. According to CDC (35–37 weeks). Not screened ->risk group | - | previous GBS child; GBS bacteriuria; temp. > 38 °C; chorioamnionitis | - | Benzylpenicillin 3 g IV– 1.5 g/h OR clindamycin 900 mg IV/8 h |
| Panneflek 2024 (3) | Gopal Rao 2017 (31) | Retrospective observational study | H | UK | - | General hospital | GBS: <7 days in blood, CSF or other sterile fluids | IAP to: carriers of rectovaginal GBS colonisation at 35–37 weeks’ gestation | - | NICE 2012. IAP to: previous infant with GBS infection, GBS bacteriuria, GBS colonisation, and fever. | - | Penicillin G 3 g IV initially and 1.5 g every 4 h. Penicillin allergy, clindamycin. |
| Li 2020 (1) | Gopal Rao 2017 (31) | Retrospective cohort | H | - | Women and their live babies | Northwick Park Hospital | GBS-EOS was defined as detection of GBS in the new-borns’ blood cultures, cerebrospinal fluid or other sterile fluids. (limited information) | 2014–2015. Limited information. Assume screening-based protocol as per the hospital’s predominant IAP practices | - | 2009–2013 and 2016–2017. Women who had a previous baby with GBS infection, GBS bacteriuria or GBS detected in vaginal swab (not by screening) during current pregnancy or intrapartum temperature of 38°C or more were recommended IAP. | - | Penicillin |
| Panneflek 2024 (3) | Gosling 2002 (32) | Questionnaire survey | C | New Zealand | - | Nineteen public hospitals | - | - | - | - | IAP to: i) carriers antenatal GBS culture, and ii) PTL, PROM, fever, previous infant with GBS infection, and GBS bacteriuria, and iii) a combination of strategy i) and ii). | Amoxicillin and penicillin. Penicillin allergy, erythromycin, clindamycin or cephalosporins. |
| Panneflek 2024 (3) | Hafner 1998 (33) | Retrospective and prospective cohort | H | Austria | - | Sociomedical centre | GBS: in throat, umbilicus, ears or blood and signs of sepsis. | IAP to: carriers of rectovaginal GBS culture at 33–35 weeks’ gestation. | - | - | IAP to: (premature) PTL, PROM, fever, previous infant with GBS infection, and maternal diabetes mellitus. | Amoxicillin with clavulanic acid 2.2 g every 6 h. Penicillin allergy, 600 mg clindamycin. |
| Li 2020 (1) | Hafner 1998 (33) | Retrospective cohort | H | - | Women and their live births | - | Limited information | 1994–1997. At the time of ultrasonographic screening for malformations, which is routinely done at our service during gestational week 22, all women were recalled for weeks 33–35. When they showed up at this time, swabs were taken from the distal third of the vagina and the rectum. IAP was offered if culture was positive. | - | 1992–1994. IAP was given to women who had premature rupture of membranes before gestational week 37, rupture of membranes 8 h or more, delivery time 18 h or more, maternal fever during delivery (37.5°C or more), group B streptococcal infection of a sibling in the past, and maternal diabetes mellitus. | - | Amoxicillin with clavulanic acid |
| Hasperhoven 2020 (6) | Hakansson 2017 (34) | Retrospective cohort study | H | Sweden | - | National registers (all hospitals included) | Positive blood or CSF culture <7 days. | NA | - | ROM> 18 h; >38°C; preterm <37 week; GBS bacteriuria; previous infant with EOGBS | - | NI |
| Panneflek 2024 (3) | Håkansson 2017 (34) | Retrospective cohort | H | Sweden | - | National health registers | GBS: <7 days in blood or CSF. | - | - | IAP to: PTL, PROM, fever, GBS bacteriuria, and previous infant with GBS infection. | - | NI |
| Panneflek 2024 (3) | Hong 2019 (35) | Retrospective cohort study | H | South Korea | - | Tertiary hospital | <7 days in blood. | CDC 2010 | - | IAP to: PROM, fever, GBS bacteriuria, and previous infant with GBS sepsis. | - | Cefazolin 2 g IV initially and 1 g every 8 h. Cephalosporin allergy, ampicillin 2 g IV initially and 1 g every 4 h. Penicillin allergy, vancomycin and clindamycin. |
| Panneflek 2024 (3) | Horváth 2013 (36) | Prospective cohort study | H | Hungary | - | University hospital | GBS: <7 days in blood or CSF with clinical signs of GBS. | - | - | - | IAP to i) carriers of rectovaginal GBS colonisation at 30–32 weeks’ gestation, and ii) PTL, (premature) PROM, fever, multiple gestation, diabetes and polyhydramnios. | Ampicillin 2 g IV initially and 1 g ampicillin every 4 h. Penicillin allergy, erythromycin or clindamycin in equivalent dosage. |
| Hasperhoven 2020 (6) | Hung 2018 (37) | Retrospective cohort study | H | Taiwan | - | National Health Insurance database (all hospitals) | < 7 days, GBS diseases is mentioned in medical record | CDC guidelines (35-37 weeks) | - | NA | - | NI |
| Panneflek 2024 (3) | Hung 2018 (37) | Retrospective cohort study | H | Taiwan | - | National Health Insurance database (all hospitals) | GBS: <7 days in hospital record with ICD-9 codes for GBS sepsis, meningitis and pneumonia. | "IAP to i) carriers of rectovaginal GBS colonisation at 30–32 weeks’ gestation, and ii) PTL, (premature) PROM, fever, multiple gestation, diabetes and polyhydramnios." | - | - | IAP to i) carriers of rectovaginal GBS colonisation at 30–32 weeks’ gestation, and ii) PTL, (premature) PROM, fever, multiple gestation, diabetes and polyhydramnios. | - |
| Panneflek 2024 (3) | Isaacs & Royle 1999 (38) | Prospective observational study | H | Australia | - | Australian neonatal units of the Australasian Study Group for Neonatal Infection | <48 h in blood, CSF or urine with clinical sepsis | - | - | - | Different strategies in hospitals comprising no, risk-based, universal and other strategies. | Usually ampicillin or penicillin. |
| Panneflek 2024 (3) | Jeffery & Moses Lahra 1998 (39) | Prospective cohort study | H | Australia | - | Tertiary referral hospital | GBS: <48 h in blood and clinical signs of sepsis. | IAP to: i) carriers of vaginal GBS colonisation at 28 weeks’ gestation without risk-factors or 24 weeks if known risk-factor for preterm birth, ii) GBS bacteriuria, iii) previous infant with EOGBS infection, and iv) if unknown carrier state, presence of preterm labour. | - | - | - | Ampicillin 1 g every 6 h. Penicillin allergy, cephalosporin. |
| Panneflek 2024 (3) | Johansson Gudjónsdóttir 2019 (40) | Retrospective cohort study | H | Sweden | - | University hospital | GBS <7 days in blood or CSF. | - | - | IAP to: PTL, PROM, fever, previous infant with EOGBS infection, and GBS bacteriuria. | - | NI |
| Panneflek 2024 (3) | Katz 1994 (41) | Retrospective cohort | H | USA | - | Public academic tertiary care medical centre | GBS: in blood | IAP to: i) carriers of rectovaginal GBS culture at 24–28 weeks’ gestation, ii) GBS bacteriuria, and iii) if carrier state unknown, presence of PROM and PTL. | - | - | - | Ampicillin 2 g IV every 6 h. Penicillin allergy, cefazolin 500 mg or clindamycin 600 mg every 6 h. |
| Panneflek 2024 (3) | Katz 1999 (42) | Retrospective cohort | H | USA | - | Urban tertiary centre | GBS: <7 days in blood or CSF with clinical signs of sepsis. | - | - | - | IAP to: i) carriers of rectovaginal GBS culture at 28 weeks’ gestation and PTL, and ii) (premature) PROM, fever and previous infant with GBS infection. | Ampicillin 2 g IV every 6 h. Penicillin allergy, clindamycin 600 mg every 6 h. |
| Panneflek 2024 (3) | Ko 2021 (43) | Retrospective cohort study | H | Taiwan | - | Two referral hospitals with level 3 NICUs | GBS <3 days in blood or CSF. | IAP to: carriers of rectovaginal GBS colonisation at 35–37 weeks’ gestation. | - | IAP to: PTL, PROM, fever, previous infant with invasive GBS infection and GBS bacteriuria. | - | Ampicillin, penicillin or cefazolin. |
| Newly identified primary study | Kolkman 2020 (44, 45) | Randomised study | C | Netherlands | Pregnant women >30weeks | Three obstetric collaboration regions consisting of one hospital and three to five midwifery practices | - | - | - | IAP is prescribed for all women with an EOGBS risk factor (a previous child with EOGBS, GBS bacteriuria, intrapartum fever, preterm birth or rupture of membranes >18 h) | Dutch strategy: IAP is prescribed in the event of a previous child with EOGBS infection, GBS bacteriuria in pregnancy or maternal fever. A rectovaginal GBS culture is taken in women with imminent preterm birth or rupture of the membranes >18 h, and IAP is provided if the test result is positive. Test results are available within 24–72 h. In the absence of a test result and when birth is imminent, the prescription of IAP is based on clinical observation of signs of infection by care providers. Combination strategy: all women are screened for GBS at 35–37 weeks and IAP is prescribed in women with detected GBS colonisation AND an EOGBS risk factor | first boost with 2 million IE penicillin G followed by 1 million IE penicillin G every 4 h |
| Panneflek 2024 (3) | Lee 2021 (46) | Retrospective cohort study | H | China | - | Tertiary university hospital | GBS: <7 days in blood. | IAP to: i) carriers of rectovaginal GBS colonisation at 35–37 weeks’ gestation. | - | IAP to: i) PTL, PROM and fever. | - | Ampicillin 2 g IV initially and 1 g every 4 h. |
| Panneflek 2024 (3) | Levine 1999 (47) | Retrospective observational study | H | USA | - | The Infection Control Surveillance Database | GBS: <7 days in blood or CSF. | - | - | - | CDC 1996 | Ampicillin or cefoxitin. |
| Panneflek 2024 (3) | Lin 2011 (47) | Retrospective cohort study | H | Taiwan | - | Private hospital | GBS: <72 h in blood. | IAP to: i) carriers of rectovaginal GBS culture at 35–37 weeks’ gestation, ii) GBS bacteriuria, iii) previous infant with GBS infection, and iv) if unknown carrier state, presence of PTL, PROM and fever. | - | - | - | Ampicillin 2 g IV initially and 1 g every 4 h. Alternatively, penicillin G. Ampicillin allergy, cefazolin. Penicillin allergy, clindamycin or erythromycin. |
| Panneflek 2024 (3) | Locksmith 1999 (48) | Retrospective cohort | H | USA | - | University tertiary care centre | GBS: <7 days in blood or CSF. | CDC 1996 | - | - | Until 1993, IAP to: carriers of GBS colonisation at hospital with PTL, premature PROM and another risk-factor(unspecified). | Until 1995, ampicillin 2 g IV initially and 1 g every 6 h. After 1995, penicillin 5 million IU and 2.5 million IU every 4 h. Amoxicillin for premature PROM and GBS colonisation. |
| Panneflek 2024 (3) | Locksmith 1999 (ii) (48) | Retrospective cohort | H | USA | - | University tertiary care centre | GBS: <7 days in blood or CSF. | CDC 1996. | - | - | After 1993, ACOG guidelines but unknown which ones. IAP to: i) if unknown carrier state, presence of PTL, PROM, fever, and previous infant with GBS sepsis, and ii) strategy above. | Until 1995, ampicillin 2 g IV initially and 1 g every 6 h. After 1995, penicillin 5 million IU and 2.5 million IU every 4 h. Amoxicillin for premature PROM and GBS colonisation. |
| Li 2020 (1) | Locksmith 1999 (48) | Retrospective cohort study | H | - | Women and their live infants | University of Florida–Shands Hospital | Early-onset group B streptococcal infection was culture defined as the isolation of group B streptococci from a normally sterile site in an infant younger than 7 d. | 1996–1998. We obtained cultures for all pregnant women between 35 and 37 weeks of gestation and offered intrapartum antibiotics to all who were found to have group B streptococcal colonization. When colonization status was unknown chemoprophylaxis was provided on the basis of risk factors. | - | 1993–1996. Risk factors were defined as anticipated delivery before 37 weeks of gestation, preterm premature rupture of membranes, duration of membrane rupture 18 h or more (prolonged rupture of membranes), maternal fever indicative of chorioamnionitis, and a previous infant with group B streptococcal infection. Women who demonstrated either of the 2 risk factors received antibiotics in labor uniformly, regardless of colonization status. | - | Ticarcillin with clavulanic acid or amoxicillin |
| Panneflek 2024 (3) | López Sastre 2005 (49) | Prospective surveillance study | H | Spain | - | Twenty-eight acute care teaching hospitals | GBS: <3 days in blood with one clinical sign and at least one laboratory abnormality consistent with infection. | IAP to: i) carriers of rectovaginal GBS culture at 35–37 weeks’ gestation, ii) GBS bacteriuria, and iii) previous infant with GBS infection. | - | - | - | Societies of Clinical Microbiology and Infectious Disease and Chemotherapy 1998: Ampicillin 2 g IV initially and 1 g every 4 h or penicillin G 5 million IU IV initially and 2.5 million IU every 4 h. Beta-lactam allergy, clindamycin 900 mg every 8 h, or erythromycin 500 mg every 6 h. |
| Panneflek 2024 (3) | Lu 2022 (50) | Retrospective cohort study | H | Taiwan | - | University hospital with a level 3 NICU | GBS: <72 h in blood or CSF. | IAP to: carriers of rectovaginal GBS culture at 35–37 weeks’ gestation. | - | - | - | NI |
| Panneflek 2024 (3) | Lukacs & Schrag 2012 (51) | Cross sectional study | H | USA | - | National Centre for Health Statistics’ National Hospital Discharge Survey data | All: <7 days with ICD-9 codes for infection in perinatal period or septicaemia. | CDC 2002 IAP to: i) carriers of rectovaginal GBS culture at 35–37 weeks’ gestation, ii) previous baby with invasive GBS infection, iii) GBS bacteriuria, and iv) if GBS carrier rate unknown, PTL, PROM and fever. ACOG 2002 endorses CDC 2002 guidelines | - | - | CDC 1996. AAP 1997 IAP to: i) GBS bacteriuria, ii) previous infant with GBS infection, iii) carriers of rectovaginal GBS culture at 35–37 weeks’ gestation, iv) if unknown carrier state, presence of risk-factors, and iv) PTL, PROM and fever. ACOG 1996 guidelines endorse CDC 1996. | CDC 1996, ACOG 1996 (penicillin), ACOG 2002, CDC 2002, and AAP 1997: Penicillin G 5 million IU IV initially and 2.5 million IU every 4 h. Alternatively, ampicillin 2 g IV initially and 1 g every 4 h. Penicillin allergy, clindamycin or erythromycin IV. |
| Hasperhoven 2020 (6) | Ma 2018 (52) | Retrospective cohort study | H | Hong Kong | - | Eight cohort study hospitals of Hong Kong | Positive blood or CSF culture <7 days | 35–37 weeks GA, later or at admission. 2 separate swabs: vagina and rectum. Non-screened -> risk-based. (women with previous GBS child excluded) practice + tertiary referral centre | - | A previous delivery with EOGBS; GBS bacteriuria; ROM 18 h or more; GA <37 weeks; Intrapartum fever | - | NI |
| Panneflek 2024 (3) | Ma 2018 (52) | Retrospective cohort study | H | China | - | Eight public hospitals and 27 maternal and child health centres | GBS: <7 days in blood, or CSF | IAP to carriers of rectovaginal GBS colonisation at 35–37 weeks’ gestation. | - | IAP to: PTL, PROM, fever, previous infant GBS disease, and GBS bacteriuria. | NI | NI |
| Hasperhoven 2020 (6) | Main & Slagle 2000 (53) | Retrospective cohort & prospective observational study | H | USA | - | Primary obstetric | Positive blood or CSF culture <7 days | 35–37 weeks vaginal and rectal culture (+IAP for preterm) | - | preterm <37, ROM> 18 h, temp. >38 °C, GBS bacteriuria, previous GBS | - | Ampicillin 2 g + 1 g/4 h IV OR clindamycin 900 mg/8 h |
| Panneflek 2024 (3) | Main & Slagle 2000 (53) | Prospective observational study | H | USA | - | Tertiary perinatal referral centre | GBS: <7 days in blood or CSF. | CDC 1996 = ACOG 1996. IAP to: i) carriers of rectovaginal GBS colonisation at 35–37 weeks’ gestation, ii) term patients with fever >38◦ C, iii) previous infant with GBS infection, and iv) if unknown carrier state, presence of PTL. | - | ACOG 1992 and CDC 1996. IAP to: PTL, (premature) PROM, fever, previous infant with EOGBS infection, and GBS bacteriuria. | - | ACOG 1992 and CDC 1996, but in reality: Ampicillin 2 g IV initially and 1 g every 4 h. Penicillin allergy, 900 mg clindamycin every 8 h. |
| Li 2020 (1) | Main & Slagle 2000 (53) | Prospective cohort | H | - | Women and their neonates | California Pacific Medical Center | Early onset GBD was defined as blood or CSF culture positive for group B streptococci within the first 7 d after birth. | 1994–1996. Women between 35 and 37 weeks were received vaginal and anal swabs cultured in selective media. Women with pending results or risk factors were treated with IAP. | - | 1992–1993. Followed the CDC guidelines, risk factors were defined as preterm labor, preterm rupture of membranes, prolonged rupture of membranes (18 h or more) at term, fever (38°C or above), previous infant with GBS-EOS, maternal urinary tract infection with GBS during the index pregnancy (limited information). | - | Ampicillin |
| Panneflek 2024 (3) | Matsubara 2007 (54) | Multicentre questionnaire survey | H | Japan | - | Twenty-eight regional hospitals with 9 NICUs | EOGBS | 23 hospitals routine antenatal determination (likely 33–37 weeks’ gestation in Japan according to JSOG guidelines). | - | - | - | Ampicillin, piperacillin, or cefotiam. Loading doses of antibiotics varied from 0.5 to 2 g and interval differed from every 4 h until every 12 h. |
| Panneflek 2024 (3) | Matsubara 2013 (55) | Retrospective nationwide questionnaire surveillance | H | Japan | - | One hundred fifty-four hospital, 14 managed only out born infants in absence of obstetric department, 62 NICUs and 76 regional centres | GBS: <7 days in blood, CSF or joint aspirate. | IAP to: i) carriers of GBS colonisation at 33–37 weeks’ gestation except for elective caesarean deliveries, ii) previous infant GBS disease, and iii) if unknown carrier state, at delivery irrespective of gestational age. | - | - | - | NI |
| Newly identified primary study | Mirsky 2020 (56) | Retrospective cohort | C | USA | Women delivering liveborn infants | Hospital database | NR | Index screen: IAP to GBS+ | Rescreening: IAP to GBS+ | - | - | NR "GBS-specific prophylactic antibiotics" |
| Panneflek 2024 (3) | O’Sullivan 2019 (57) | Prospective active national surveillance study | H | UK and Ireland | - | Active surveillance (all paediatricians) and laboratory databases (all) | GBS: <7 days in blood, CSF or joint fluid. | - | - | RCOG 2003: IAP to: PTL, fever, previous infant with GBS infection, GBS bacteriuria, GBS colonisation, and suspected chorioamnionitis. NICE 2012 | - | Penicillin G, cephalosporin or vancomycin. |
| Hasperhoven 2020 (6) | O’Sullivan 2019 (57) | Retrospective cohort study | H | UK | - | Active surveillance (all paediatricians) and laboratory databases (all) | < 7 days, positive culture from blood or CSF or joint fluid | NA | - | Previous GBS child or bacteriuria -> IAP. Preterm<37, fever >38, PPROM, prolonged ROM>18 hours-> IAP | - | Penicillin/ ampicillin |
| Panneflek 2024 (3) | Petersen 2014 (58) | Retrospective cohort study | H | Denmark | - | Public referral centre | <7 days in blood, CSF or tracheal swab. | - | - | Denmark recommendation 2004: IAP to: PTL, PROM, fever, GBS bacteriuria, and previous infant with invasive GBS infection | - | NI |
| Hasperhoven 2020 (6) | Phares 2008 (59) | Retrospective cohort study | H | USA | - | All laboratories part of the Emerging Infections Program Network | <7 days, positive culture from blood or CSF | CDC guidelines | - | NA | - | NI |
| Panneflek 2024 (3) | Phares 2008 (59) | Retrospective cohort study | H | USA | - | Database Active Bacterial Core surveillance/Emerging Infections Program Network | GBS: <7 days in blood or CSF. | CDC 2002 | - | - | CDC 1996, ACOG 1996, AAP 1997. | - |
| Panneflek 2024 (3) | Poulain 1997 (60) | Prospective cohort study | H | France | - | University hospital regional reference century | GBS: Immediate in blood or CSF. | - | - | - | AP to: i) carriers of rectovaginal GBS colonisation at 28 weeks’ gestation, GBS bacteriuria and one of the following risk-factors: PTL, PROM, fever, twin pregnancies, maternal diabetes, and ii) previous infant with GBS. | Amoxicillin 2 g IV initially and 1 g every 4 h. |
| Panneflek 2024 (3) | Puopolo & Eichenwald 2010 (61) | Retrospective cohort | H | USA | - | University hospital | GBS <72 h in blood and neonatologist considered infant infected. Surviving infants treated with appropriate course of antibiotics ≥7 days. | IAP to: carriers of rectovaginal GBS colonisation at 35–37 weeks’ gestation. | - | IAP to: PTL, PROM, fever. | - | Ampicillin or clindamycin for risk-factor strategy. Penicillin G for universal strategy. Penicillin allergy, clindamycin, cefazolin, erythromycin and vancomycin. |
| Li 2020 (1) | Puopolo & Eichenwald 2010 (61) | Retrospective cohort study | H | USA | Women and their infants | Brigham and Women’s Hospital | EOS was identified through querying of any positive blood culture for a bacterial species obtained from an infant before 72 h of age. | 1997–2007. Limited information. Assume screening-based protocol as per the hospital’s predominant IAP practices. | - | 1993–1996. Limited information. Assume risk-based protocol as per the hospital’s predominant IAP practices. | - | Ampicillin or penicillin G |
| Li 2020 (1) | Reisner 2000 (62) | Prospective cohort study | H | - | Women and their live births | at Swedish Medical Center, Seattle | Limited information | 1994–1996. Swabs were used to obtain specimens from the vagino-rectal area. IAP was offered if culture was positive (limited information). | - | 1992–1993. A high-risk patient was considered to be a woman in preterm labor or with rupture of membranes at less than 37 weeks’ gestation, with group B streptococcal infection, gestation multiple, gestation complicated by intrauterine growth restriction, any previous positive culture results for group B streptococci, or prolonged rupture of membranes (18 h or more). (limited information) | - | Ampicillin |
| Panneflek 2024 (3) | Renner 2006 (63) | Retrospective observational study | H | Switzerland | - | University hospital | GBS: <7 days in blood. | - | - | - | IAP to: i) carriers of GBS screening at 35–37 weeks + PROM, fever or PTL, ii) unknown carriers state when risk-factor present, and iii) previous infant with GBS sepsis. | NI |
| Newly identified study * | Riley 2003 (64) | Retrospective cohort | C | USA | Pregnant women, full term labour | Hospital delivery records | GBS disease in a neonate less than 7 days old | CDC 1996 | - | ACOG 1996? IAP to: fever > 100.4, ROM > 18 hrs, GBS bacteriuria, prior GBS+ infant | - | Penicillin, for penicillin allergic patients - clindamycin or ampicillin |
| Panneflek 2024 (3) | Rottenstreich 2019 (65) | Retrospective cohort | H | Israel | - | University affiliated medical centre | GBS: <7 days in normally sterile site. | IAP to: i) carriers of rectovaginal GBS colonisation at 35–37 weeks’ gestation, and ii) if unknown carrier state, presence of risk-factors. | - | IAP to: PTL, PROM, fever, previous infant EOGBS, and GBS bacteriuria. | - | NI |
| Panneflek 2024 (3) | Sakata 2012 (66) | Retrospective cohort study | H | Japan | - | Public hospital | GBS: <3 days in blood or CSF with signs of infection. | JSOG and JAOG 2008 IAP to: i) carriers of rectovaginal GBS colonisation at 33–37 weeks’ gestation, ii) earlier infant GBS infection, and iii) unknown carrier state. | - | - | - | Sulbactam or ampicillin 1.5 g. |
| Hasperhoven 2020 (6) | Schrag 2002 (67) | Retrospective cohort study | C | USA | - | Multiple hospitals of the Emerging Infections Program Network | Pos. blood culture, CSF or other ‘normally sterile fluid’. Days unclear. | Retrospective selection: found any GBS status in record, taken at least 2 days before birth -> screening group | - | preterm <37, rupture> 18 h, temp. >38°C, GBS bacteriuria, previous GBS | - | NI |
| Panneflek 2024 (3) | Schrag 2002 (67) | Retrospective cohort study | C | USA | - | Multiple hospitals of the Emerging Infections Program Network | GBS: in normally sterile site. | IAP to: carriers of GBS culture at least 2 days before delivery. | - | IAP to: all pregnant women without documentation of test for GBS with PTL, PROM, fever, previous infant with GBS infection, and GBS bacteriuria. | - | NI |
| Li 2020 (1) | Schrag 2002 (67) | Retrospective cohort | C | - | Women and their infants | Active Bacterial Core Surveillance program of the Emerging Infections Program Network | Early onset GBS disease was defined as GBS sepsis, meningitis, or pneumonia during the initial 7 d of neonatal life. | 1998–1999. Women were screened for carriage of group B streptococcus between 35 and 37 weeks of gestation, and intrapartum chemoprophylaxis was offered to carriers | - | 1998–1999. All mothers without documentation of a test for group B streptococcus that adhered to the above criteria were categorized in the risk-based group. | - | NA |
| Panneflek 2024 (3) | Schushat 2002 (68) | Retrospective cohort | H | USA | - | Multiple hospitals of the Emerging Infections Program Network | - | IAP to: carriers of GBS culture at least 2 days before delivery | - | IAP to: all pregnant women without documentation of test for GBS with PTL, PROM, fever, previous infant with GBS infection, and GBS bacteriuria | - | Penicillin, ampicillin, or cefazolin |
| Panneflek 2024 (3) | Share 2001 (69) | Retrospective cohort | H | USA | - | University hospital | GBS:<72 h in blood or CSF. | - | - | - | AAP 1997, ACOG 1996 | AAP 1997, ACOG 1996 |
| Panneflek 2024 (3) | Sutkin 2005 (70) | Retrospective cohort study | H | USA | - | Tertiary referral hospital | - | CDC 1996. | - | - | AAP 1992 IAP to: carriers of rectovaginal GBS colonisation at 26–28 weeks’ gestation with one of the following risk-factors: PTL (premature) PROM, fever, previous infant with EOGBS disease, and GBS bacteriuria. Before 1993, there was no implemented strategy. | Penicillin and clindamycin. |
| Panneflek 2024 (3) | Towers & Briggs 2002 (71) | Prospective cohort study | H | USA | - | Non-profit hospital | GBS  <7 days in blood with clinically symptomatic infant. | - | - | IAP to: i) PTL, PROM and fever, and ii) carriers of inconsistent screening at 28 weeks’ gestation or first prenatal visit. | IAP to: carriers of GBS colonisation at 35–37- weeks’ gestation, or IAP to: PTL, PROM, fever and; ii) carriers of inconsistent GBS screening at 28 weeks’ gestation or first prenatal visit. | Until 1998 ampicillin. After 1998, penicillin G. Also use of cefazolin and cephalexin in preterm population. Penicillin allergy, erythromycin and/or clindamycin. |
| Panneflek 2024 (3) | Trijbels-Smeulders 2006 (72) | Retrospective cohort | H | The Netherlands | - | 22 Laboratories for Medical Microbiology and from the Netherlands Reference Laboratory for Bacterial Meningitis in 51/93 neonatal and pediatric wards | GBS:  ≤7 days in blood/CSF | - | - | - | Dutch guidelines 1999. | Dutch guidelines 1999 |
| Panneflek 2024 (3) | Trijbels-Smeulders 2007 (73) | Retrospective observational study | H | The Netherlands | - | Dutch Paediatric Surveillance Unit database | GBS: <7 d in blood, CSF or other sterile fluids | - | - | - | Dutch guidelines 1999. | Dutch guidelines 1999. |
| Panneflek 2024 (3) | Trollfors 2022 (74) | Retrospective observational study | H | Sweden | All live births in southwest Sweden | - | GBS: <7 days. | - | - | IAP: fever, PTL, PROM, previous infant with invasive GBS infection, and GBS bacteriuria. | - | Penicillin. |
| Panneflek 2024 (3) | Uy 2002 (75) | Retrospective population survey | H | USA | - | Tertiary care centre, division of university hospital | GBS: <7 days in blood or CSF. | - | - | CDC 1996. Adhere to risk- based guidelines, but prescription left to individual practitioner. | AAP 1992, ACOG 1992. | AAP 1992: Ampicillin 2 g IV initially, 1–2 g every 4–6 h or penicillin G 5 million IU every 6h. Penicillin allergy, clindamycin or erythromycin. Also ACOG 1992 and CDC 1996. |
| Panneflek 2024 (3) | van den Hoogen 2010 (76) | Retrospective cohort study | H | The Netherlands | - | University hospital level 3 NICU unit | Non-GBS2: All3:<48 h in blood and clinical signs of infection. | - | - | - | Dutch guidelines 1999. | Dutch guidelines 1999. |
| Panneflek 2024 (3) | van Dyke 2009 (77) | Retrospective cohort study | H | USA | - | Active Bacterial Core surveillance system 10 US states | GBS: <7 days in normally sterile site. | Any documented colonisation prenatally or at admission performed 2 days or more before delivery, IAP to carriers of GBS colonisation. | - | - | Schrag et al. 2002:  IAP to: i) carriers of GBS culture at least 2 days before delivery, and ii) all pregnant women without documentation of test for GBS with following risk factors: PROM, PTL, fever, GBS bacteriuria and previous infant with GBS infection | Penicillin, ampicillin, cefazolin, clindamycin or vancomycin. |
| Hasperhoven 2020 (6) | Vergani 2002 (78) | Retrospective cohort study | H | Italy | - | One tertiary care centre (university hospital) | Positive blood or CSF culture <7 days | <’97 Vaginal culture between 26–28-week GA. >’97 between 35–37-week GA | - | Preterm <37; ROM >12 h; temp. >37.5°C, GBS bacteriuria; previous child GBS | - | Ampicillin 2 g + 1 g/4 h IV OR Erythromycin 500 mg/6 h |
| Panneflek 2024 (3) | Vergani 2002 (78) | Retrospective cohort | H | Italy | - | Tertiary referral centre | GBS1, Non-GBS2, All3: <7 days in blood, CSF, auricular or pharyngeal culture with at least two inflammatory indices. | - | - | IAP to: PTL, PROM, fever, previous infant with GBS infection, and GBS bacteriuria. | IAP to: i) carriers of rectovaginal GBS culture at 26–28 and 35–37 weeks’ gestation, and ii) PTL, PROM, fever, GBS bacteriuria, previous infant with GBS infection. | Ampicillin 2 g IV initially and 1 g every 4 h. Penicillin allergy, erythromycin 500 mg every 6 h. |
| Li 2020 (1) | Vergani 2002 (78) | Retrospective cohort study | H | - | Women and their neonates | - | Early onset GBS morbidity was defined as GBS sepsis, meningitis, or pneumonia during the initial 7 d of neonatal life. | 1995–1999. Cultures from the distal vagina and anus were obtained with a Dacron swab, plated onto a Columbia colistin- nalidixic acid agar plate and then inoculated into selective broth medium. Women with positive cultures or with risk factors independently from culture results received antibiotic prophylaxis. The antibiotic regimen used was unchanged from the previous study period. GBS screening was performed at 26–28 weeks until December 1997, and at 35–37 weeks from January 1998 to December 1999. | - | 1991–1994. Women with risk factors for neonatal GBS disease, such as preterm labor at less than 37 weeks, prolonged rupture of membranes for over 12 h, maternal fever over 37.5°C, maternal urinary tract infection with group B streptococci during the index pregnancy, or previous pregnancy complicated by neonatal GBS infection, received antibiotic prophylaxis during labor. | - | Ampicillin |
| Panneflek 2024 (3) | Wicker 2019 (79) | Prospective surveillance study | H | Germany | - | German Paediatric Surveillance Unit/Survey of Rare Diseases and Robert Koch Institute | GBS: <7 days in blood or CSF. | IAP to: i) carriers of rectovaginal GBS culture at 35–37 weeks’ gestation, and ii) if carrier state unknown, presence of risk- factors. | - | - | IAP to 2 strategies: i) carriers of rectovaginal culture at 35–37 weeks’ gestation, or ii) PTL, PROM, fever, previous infant with GBS infection, and GBS bacteriuria. | NI |
| Panneflek 2024 (3) | Youden 2005 (80) | Prospective observational study | C | Canada | - | Level one paediatric trauma centre | - | IAP to: carriers of rectovaginal GBS culture at 35-37 weeks’ gestation. | - | IAP to: (premature) PROM, fever, multiple gestation, previous infant with GBS infection and GBS bacteriuria | - | Penicillin G 5 million IU initially and 2.5 million IU every 4 h, or ampicillin 2 g initially and 1 g every 4 h. Penicillin allergy, clindamycin 900 mg every 8 hours, erythromycin 500 mg every 6 h, or cefazolin 2 g every 8 h |

* Newly identified study Riley 2003 was assessed as excluded in Panneflek 2024 as it did not report outcomes of interest.

**Abbreviations:** C: concurrent; CSF: cerebrospinal fluid; EOGBS: early onset GBS; g: grams; H = historical; IAP: intrapartum antibiotic prophylaxis; IU: international units; IV: intravenous; LOGBS: late onset GBS; mg: micrograms; NI: no information; NICU: neonatal intensive care unit; NL: the Netherlands; PCR: polymerase chain reaction; PROM: premature rupture of membranes; PTL: preterm labour; RCOG: Obstetricians and Gynaecologists; ROM: rupture of membrane

References

1. Li QY, Wang DY, Li HT, Liu JM. Screening-based and Risk-based Strategy for the Prevention of Early-onset Group B Streptococcus/Non-group B Streptococcus Sepsis in the Neonate: A Systematic Review and Meta-analysis. *Pediatr Infect Dis J* 2020; 39 8:740-8.

2. Abdelmaaboud M, Mohammed AF. Universal screening vs. risk-based strategy for prevention of early-onset neonatal Group-B streptococcal disease. *J Trop Pediatr* 2011; 57 6:444-50.

3. Panneflek TJR, Hasperhoven GF, Chimwaza Y, Allen C, Lavin T, Te Pas AB, et al. Intrapartum antibiotic prophylaxis to prevent Group B streptococcal infections in newborn infants: a systematic review and meta-analysis comparing various strategies. *EClinicalMedicine* 2024; 74:102748.

4. Al Luhidan L, Madani A, Albanyan EA, Al Saif S, Nasef M, AlJohani S, et al. Neonatal Group B Streptococcal Infection in a Tertiary Care Hospital in Saudi Arabia: A 13-year Experience. *Pediatr Infect Dis J* 2019; 38 7:731-4.

5. Alarcon A, Pena P, Salas S, Sancha M, Omenaca F. Neonatal early onset Escherichia coli sepsis: trends in incidence and antimicrobial resistance in the era of intrapartum antimicrobial prophylaxis. *Pediatr Infect Dis J* 2004; 23 4:295-9.

6. Hasperhoven GF, Al-Nasiry S, Bekker V, Villamor E, Kramer B. Universal screening versus risk-based protocols for antibiotic prophylaxis during childbirth to prevent early-onset group B streptococcal disease: a systematic review and meta-analysis. *BJOG* 2020; 127 6:680-91.

7. Angstetra D, Ferguson J, Giles WB. Institution of universal screening for Group B streptococcus (GBS) from a risk management protocol results in reduction of early-onset GBS disease in a tertiary obstetric unit. *Aust N Z J Obstet Gynaecol* 2007; 47 5:378-82.

8. Bauserman MS, Laughon MM, Hornik CP, Smith PB, Benjamin DK, Jr., Clark RH, et al. Group B Streptococcus and Escherichia coli infections in the intensive care nursery in the era of intrapartum antibiotic prophylaxis. *Pediatr Infect Dis J* 2013; 32 3:208-12.

9. Bekker V, Bijlsma MW, van de Beek D, Kuijpers TW, van der Ende A. Incidence of invasive group B streptococcal disease and pathogen genotype distribution in newborn babies in the Netherlands over 25 years: a nationwide surveillance study. *Lancet Infect Dis* 2014; 14 11:1083-9.

10. Bizzarro MJ, Raskind C, Baltimore RS, Gallagher PG. Seventy-five years of neonatal sepsis at Yale: 1928-2003. *Pediatrics* 2005; 116 3:595-602.

11. Bjorklund V, Nieminen T, Ulander VM, Ahola T, Saxen H. Replacing risk-based early-onset-disease prevention with intrapartum group B streptococcus PCR testing. *J Matern Fetal Neonatal Med* 2017; 30 3:368-73.

12. Brozanski BS, Jones JG, Krohn MA, Sweet RL. Effect of a screening-based prevention policy on prevalence of early-onset group B streptococcal sepsis. *Obstet Gynecol* 2000; 95 4:496-501.

13. Chan YTV, Lau SYF, Hui SYA, Ma T, Kong CW, Kwong LT, et al. Incidence of neonatal sepsis after universal antenatal culture-based screening of group B streptococcus and intrapartum antibiotics: A multicentre retrospective cohort study. *BJOG* 2023; 130 1:24-31.

14. Chen KT, Tuomala RE, Cohen AP, Eichenwald EC, Lieberman E. No increase in rates of early-onset neonatal sepsis by non-group B Streptococcus or ampicillin-resistant organisms. *Am J Obstet Gynecol* 2001; 185 4:854-8.

15. Chen KT, Puopolo KM, Eichenwald EC, Onderdonk AB, Lieberman E. No increase in rates of early-onset neonatal sepsis by antibiotic-resistant group B Streptococcus in the era of intrapartum antibiotic prophylaxis. *Am J Obstet Gynecol* 2005; 192 4:1167-71.

16. Cho CY, Tang YH, Chen YH, Wang SY, Yang YH, Wang TH, et al. Group B Streptococcal infection in neonates and colonization in pregnant women: An epidemiological retrospective analysis. *J Microbiol Immunol Infect* 2019; 52 2:265-72.

17. Coco AS. Comparison of two prevention strategies for neonatal group B streptococcal disease. *J Am Board Fam Pract* 2002; 15 4:272-6.

18. Daniels J, Dixon EF, Gill A, Bishop J, D'Amico M, Ahmed K, et al. A rapid intrapartum test for group B Streptococcus to reduce antibiotic usage in mothers with risk factors: the GBS2 cluster RCT. *Health Technology Assessment (Winchester, England)* 2022; 26 12:1-82.

19. Daniels JP, Dixon E, Gill A, Bishop J, Wilks M, Millar M, et al. Rapid intrapartum test for maternal group B streptococcal colonisation and its effect on antibiotic use in labouring women with risk factors for early-onset neonatal infection (GBS2): cluster randomised trial with nested test accuracy study. *BMC Medicine* 2022; 20 1:9.

20. Darlow BA, Voss L, Lennon DR, Grimwood K. Early-onset neonatal group B streptococcus sepsis following national risk-based prevention guidelines. *Aust N Z J Obstet Gynaecol* 2016; 56 1:69-74.

21. Davis RL, Hasselquist MB, Cardenas V, Zerr DM, Kramer J, Zavitkovsky A, et al. Introduction of the new Centers for Disease Control and Prevention group B streptococcal prevention guideline at a large West Coast health maintenance organization. *Am J Obstet Gynecol* 2001; 184 4:603-10.

22. Eberly MD, Rajnik M. The effect of universal maternal screening on the incidence of neonatal early-onset group B streptococcal disease. *Clin Pediatr (Phila)* 2009; 48 4:369-75.

23. Ecker KL, Donohue PK, Kim KS, Shepard JA, Aucott SW. The impact of group B Streptococcus prophylaxis on early onset neonatal infections. *J Neonatal Perinatal Med* 2013; 6 1:37-44.

24. Edwards RK, Jamie WE, Sterner D, Gentry S, Counts K, Duff P. Intrapartum antibiotic prophylaxis and early-onset neonatal sepsis patterns. *Infect Dis Obstet Gynecol* 2003; 11 4:221-6.

25. Eisenberg E, Craig AS, Gautam S, Khalil MM, Shaktour B, Schaffner W, et al. Beyond screening: identifying new barriers to early onset group B streptococcal disease prevention. *Pediatr Infect Dis J* 2005; 24 6:520-4.

26. El Helali N, Habibi F, Azria E, Giovangrandi Y, Autret F, Durand-Zaleski I, et al. Point-of-Care Intrapartum Group B Streptococcus Molecular Screening: Effectiveness and Costs. *Obstetrics & Gynecology* 2019; 133 2:276-81.

27. Factor SH, Levine OS, Nassar A, Potter J, Fajardo A, O'Sullivan MJ, et al. Impact of a risk-based prevention policy on neonatal group B streptococcal disease. *Am J Obstet Gynecol* 1998; 179 6 Pt 1:1568-71.

28. Garland SM. Early onset neonatal Group-B streptococcus (GBS) infection - associated obstetric risk-factors. *Aust N Z J Obstet Gynaecol* 1991; 31 2.

29. Gibbs RS, McDuffie RS, Jr., McNabb F, Fryer GE, Miyoshi T, Merenstein G. Neonatal group B streptococcal sepsis during 2 years of a universal screening program. *Obstet Gynecol* 1994; 84 4:496-500.

30. Gilson GJ, Christensen F, Romero H, Bekes K, Silva L, Qualls CR. Prevention of group B streptococcus early-onset neonatal sepsis: comparison of the Center for Disease Control and prevention screening-based protocol to a risk-based protocol in infants at greater than 37 weeks' gestation. *J Perinatol* 2000; 20 8 Pt 1:491-5.

31. Gopal Rao G, Townsend J, Stevenson D, Nartey G, Hiles S, Bassett P, et al. Early-onset group B Streptococcus (EOGBS) infection subsequent to cessation of screening-based intrapartum prophylaxis: findings of an observational study in West London, UK. *BMJ Open* 2017; 7 11:e018795.

32. Gosling IA, Stone PR, Grimwood K. Early-onset group B streptococcus prevention protocols in New Zealand public hospitals. *Aust N Z J Obstet Gynaecol* 2002; 42 4:362-4.

33. Hafner E, Sterniste W, Rosen A, Schuchter K, Plattner M, Asboth F, et al. Group B streptococci during pregnancy: a comparison of two screening and treatment protocols. *Am J Obstet Gynecol* 1998; 179 3 Pt 1:677-81.

34. Hakansson S, Lilja M, Jacobsson B, Kallen K. Reduced incidence of neonatal early-onset group B streptococcal infection after promulgation of guidelines for risk-based intrapartum antibiotic prophylaxis in Sweden: analysis of a national population-based cohort. *Acta Obstet Gynecol Scand* 2017; 96 12:1475-83.

35. Hong JY, . , Kim SH, ., Kim SM, al. e. Evaluation of the early onset neonatal sepsis according to two antenatal group B Streptococcus screening methods: risk-based versus universal screening. *Perinatology* 2019; 30 4:200-7.

36. Horvath B, Grasselly M, Bodecs T, Boncz I, Bodis J. Screening pregnant women for group B streptococcus infection between 30 and 32 weeks of pregnancy in a population at high risk for premature birth. *Int J Gynaecol Obstet* 2013; 122 1:9-12.

37. Hung LC, Kung PT, Chiu TH, Su HP, Ho M, Kao HF, et al. Risk factors for neonatal early-onset group B streptococcus-related diseases after the implementation of a universal screening program in Taiwan. *BMC Public Health* 2018; 18 1:438.

38. Isaacs D, Royle JA. Intrapartum antibiotics and early onset neonatal sepsis caused by group B Streptococcus and by other organisms in Australia. Australasian Study Group for Neonatal Infections. *Pediatr Infect Dis J* 1999; 18 6:524-8.

39. Jeffery HE, Moses Lahra M. Eight-year outcome of universal screening and intrapartum antibiotics for maternal group B streptococcal carriers. *Pediatrics* 1998; 101 1:E2.

40. Johansson Gudjónsdóttir M, Elfvin A, Hentz E, Adlerberth I, Tessin I, B. T. Changes in incidence and etiology of early-onset neonatal infections 1997-2017 - a retrospective cohort study in western Sweden. *BMC Pediatr* 2019; 19 1:490.

41. Katz VL, Moos MK, Cefalo RC, Thorp JM, Jr., Bowes WA, Jr., Wells SD. Group B streptococci: results of a protocol of antepartum screening and intrapartum treatment. *Am J Obstet Gynecol* 1994; 170 2:521-6.

42. Katz PF, Hibbard JU, Ranganathan D, Meadows W, Ismail M. Group B streptococcus: to culture or not to culture? *J Perinatol* 1999; 19 5:337-42.

43. Ko MH CH, Li ST, et al. An 18-year retrospective study on the epidemiology of early-onset neonatal sepsis - emergence of

uncommon pathogens. *Pediatr Neonatol* 2021; 62 5:491–8.

44. Kolkman DGE, Martin L, Jans S, Wouters M, van Dommelen P, Fleuren MAH, et al. Evaluation of women's worries in different strategies for the prevention of early onset group B streptococcal disease in neonates. *Midwifery* 2020; 86:102623.

45. Kolkman DGE, Rijnders MEB, Wouters M, Dommelen PV, de Groot CJM, Fleuren MAH. Adherence to three different strategies to prevent early onset GBS infection in newborns. *Women & Birth: Journal of the Australian College of Midwives* 2020; 33 6:e527-e34.

46. Lee J, Naiduvaje K, Chew KL, Charan N, Chan YH, Lin RT, et al. Preventing early-onset group B streptococcal sepsis: clinical risk factor-based screening or culture-based screening? *Singapore Med J* 2021; 62 1:34-8.

47. Levine EM, Ghai V, Barton JJ, Strom CM. Intrapartum antibiotic prophylaxis increases the incidence of gram-negative neonatal sepsis. *Infect Dis Obstet Gynecol* 1999; 7 4:210-3.

48. Locksmith GJ, Clark P, Duff P. Maternal and neonatal infection rates with three different protocols for prevention of group B streptococcal disease. *Am J Obstet Gynecol* 1999; 180 2 Pt 1:416-22.

49. Lopez Sastre JB, Fernandez Colomer B, Coto Cotallo GD, Ramos Aparicio A, Grupo de Hospitales C. Trends in the epidemiology of neonatal sepsis of vertical transmission in the era of group B streptococcal prevention. *Acta Paediatr* 2005; 94 4:451-7.

50. Lu IC, Chang YC, Chen YT, Lin HY, Chiu HY, Tsai ML, et al. Epidemiological evolution of early-onset neonatal sepsis over 12 years: A single center, population-based study in central Taiwan. *J Neonatal Perinatal Med* 2022; 15 3:575-82.

51. Lukacs SL, Schrag SJ. Clinical sepsis in neonates and young infants, United States, 1988-2006. *J Pediatr* 2012; 160 6:960-5 e1.

52. Ma TWL, Chan V, So CH, Hui ASY, Lee CN, Hui APW, et al. Prevention of early onset group B streptococcal disease by universal antenatal culture-based screening in all public hospitals in Hong Kong. *J Matern Fetal Neonatal Med* 2018; 31 7:881-7.

53. Main EK, Slagle T. Prevention of early-onset invasive neonatal group B streptococcal disease in a private hospital setting: the superiority of culture-based protocols. *Am J Obstet Gynecol* 2000; 182 6:1344-54.

54. Matsubara K, Kawai M, Nakahata T, Kato F, Tsukahara H, Yamakawa M, et al. Procedures for prevention of perinatal group B streptococcal diseases: a multicenter questionnaire survey of hospitals in the Kyoto Neonatal Disease Study Group, Japan. *J Infect Chemother* 2007; 13 1:59-62.

55. Matsubara K, Hoshina K, Suzuki Y. Early-onset and late-onset group B streptococcal disease in Japan: a nationwide surveillance study, 2004-2010. *Int J Infect Dis* 2013; 17 6:e379-84.

56. Mirsky R, Carpenter DM, Postlethwaite DA, Regenstein AC. Preventing early-onset group B streptococcal sepsis: is there a role for rescreening near term? *Journal of Maternal-Fetal & Neonatal Medicine* 2020; 33 22:3791-7.

57. O'Sullivan CP, Lamagni T, Patel D, Efstratiou A, Cunney R, Meehan M, et al. Group B streptococcal disease in UK and Irish infants younger than 90 days, 2014-15: a prospective surveillance study. *Lancet Infect Dis* 2019; 19 1:83-90.

58. Petersen KB, Johansen HK, Rosthoj S, Krebs L, Pinborg A, Hedegaard M. Increasing prevalence of group B streptococcal infection among pregnant women. *Dan Med J* 2014; 61 9:A4908.

59. Phares CR, Lynfield R, Farley MM, Mohle-Boetani J, Harrison LH, Petit S, et al. Epidemiology of invasive group B streptococcal disease in the United States, 1999-2005. *JAMA* 2008; 299 17:2056-65.

60. Poulain P, Betremieux P, Donnio PY, Proudhon JF, Karege G, Giraud JR. Selective intrapartum anti-bioprophylaxy of group B streptococci infection of neonates: a prospective study in 2454 subsequent deliveries. *Eur J Obstet Gynecol Reprod Biol* 1997; 72 2:137-40.

61. Puopolo KM, Eichenwald EC. No change in the incidence of ampicillin-resistant, neonatal, early-onset sepsis over 18 years. *Pediatrics* 2010; 125 5:e1031-8.

62. Reisner DP, Haas MJ, Zingheim RW, Williams MA, Luthy DA. Performance of a group B streptococcal prophylaxis protocol combining high-risk treatment and low-risk screening. *Am J Obstet Gynecol* 2000; 182 6:1335-43.

63. Renner RM, Renner A, Schmid S, Hoesli I, Nars P, Holzgreve W, et al. Efficacy of a strategy to prevent neonatal early-onset group B streptococcal (GBS) sepsis. *J Perinat Med* 2006; 34 1:32-8.

64. Riley L, Appollon K, Haider S, Chan-Flynn S, Cohen A, Ecker J, et al. "Real World" compliance with strategies to prevent early-onset group B streptococcal disease. *J Perinatol* 2003; 23 4:272-7.

65. Rottenstreich M, Rotem R, Bergman M, Farkash R, Schimmel MS, Samueloff A, et al. Assessment of maternal GBS colonization and early-onset neonatal disease rate for term deliveries: a decade perspective. *J Perinat Med* 2019; 47 5:528-33.

66. Sakata H. Evaluation of intrapartum antibiotic prophylaxis for the prevention of early-onset group B streptococcal infection. *J Infect Chemother* 2012; 18 6:853-7.

67. Schrag SJ, Zell ER, Lynfield R, Roome A, Arnold KE, Craig AS, et al. A population-based comparison of strategies to prevent early-onset group B streptococcal disease in neonates. *N Engl J Med* 2002; 347 4:233-9.

68. Schuchat A, Roome A, Zell ER, Linardos H, Zywicki S, O'Brien KL. Integrated monitoring of a new group B streptococcal disease prevention program and other perinatal infections. *Matern Child Health J* 2002; 6 2:107-14.

69. Share L, Chaikin S, Pomeranets S, Kiwi R, Jacobs M, Fanaroff AA. Implementation of guidelines for preventing early onset group B streptococcal infection. *Semin Perinatol* 2001; 25 2:107-13.

70. Sutkin G, Krohn MA, Heine RP, Sweet RL. Antibiotic prophylaxis and non-group B streptococcal neonatal sepsis. *Obstet Gynecol* 2005; 105 3:581-6.

71. Towers CV, Briggs GG. Antepartum use of antibiotics and early-onset neonatal sepsis: the next 4 years. *Am J Obstet Gynecol* 2002; 187 2:495-500.

72. Trijbels-Smeulders MA, Kimpen JL, Kollee LA, Bakkers J, Melchers W, Spanjaard L, et al. Serotypes, genotypes, and antibiotic susceptibility profiles of group B streptococci causing neonatal sepsis and meningitis before and after introduction of antibiotic prophylaxis. *Pediatr Infect Dis J* 2006; 25 10:945-8.

73. Trijbels-Smeulders M, de Jonge GA, Pasker-de Jong PC, Gerards LJ, Adriaanse AH, van Lingen RA, et al. Epidemiology of neonatal group B streptococcal disease in the Netherlands before and after introduction of guidelines for prevention. *Arch Dis Child Fetal Neonatal Ed* 2007; 92 4:F271-6.

74. Trollfors B MF, Gudjonsdottir MJ, et al. . Group B streptococcus - a pathogen not restricted to neonates. *IJID Reg* 2022; 4:171-5.

75. Uy IP, D'Angio CT, Menegus M, Guillet R. Changes in early-onset group B beta hemolytic streptococcus disease with changing recommendations for prophylaxis. *J Perinatol* 2002; 22 7:516-22.

76. van den Hoogen A, Gerards LJ, Verboon-Maciolek MA, Fleer A, Krediet TG. Long-term trends in the epidemiology of neonatal sepsis and antibiotic susceptibility of causative agents. *Neonatology* 2010; 97 1:22-8.

77. Van Dyke MK, Phares CR, Lynfield R, Thomas AR, Arnold KE, Craig AS, et al. Evaluation of universal antenatal screening for group B streptococcus. *N Engl J Med* 2009; 360 25:2626-36.

78. Vergani P, Patane L, Colombo C, Borroni C, Giltri G, Ghidini A. Impact of different prevention strategies on neonatal group B streptococcal disease. *Am J Perinatol* 2002; 19 6:341-8.

79. Wicker E, Lander F, Weidemann F, Hufnagel M, Berner R, Krause G. Group B Streptococci: Declining Incidence in Infants in Germany. *Pediatr Infect Dis J* 2019; 38 5:516-9.

80. Youden L, Downing M, Halperin B, Scott H, Smith B, Halperin SA. Group B streptococcal testing during pregnancy: survey of postpartum women and audit of current prenatal screening practices. *J Obstet Gynaecol Can* 2005; 27 11:1006-12.
