# Supplementary material for: Benefits and Harms of Antenatal/Intrapartum Screening for Maternal Group B Streptococcus and Use of Intrapartum Antibiotic Prophylaxis Versus Risk‐Based Protocols or No Intervention: A Rapid Review
Source: Acta Paediatr. 2026 Apr 30;115(8):1598–610. doi: 10.1111/apa.70568 (PMC13371836; doi:10.1111/apa.70568)
Supplement: Supplementary file 15 — Data S15: Demographics: Primary study level. [file APA-115-1598-s016.docx]

## Supplementary materials File 15 (S15). Table of demographics at primary study level

Key study demographics reported by primary study and presented by approach when information available.

| **Study ID** | **Aim of study** | **Inclusion criteria** | **Exclusion criteria** | **LOGBS definition** | **Approach comparison*** | **Approach /Strategy** | **Women (n)** | **Neonates (n)** | **Women age (years, SD)** | **Gestational age (weeks, SD)** | **Ethnicity** | **Prevalence of GBS colonisation (n, (%))** | **Number of women with high risk factors** | **Other clinical risk factors reported** |
| --- | --- | --- | --- | --- | --- | --- | --- | --- | --- | --- | --- | --- | --- | --- |
| Abdelmaaboud 2011 | To evaluate the difference between universal screening and risk-based strategies in the prevention of EOGBS disease | NR | NR | NR | SR | Screening/Universal | 657 | 657 | 24.6 (3.2) | NR | Qatari 288 (43.8)  Non-Qatari 369 (56.2) | NR | Preterm delivery 46 (6.9)  PROM 18 h 53 (8.1)  Intrapartum fever 22 (3.6) GBS bacteriuria 22 (3.3) Previous infant with GBS disease 3 (0.49) | NR |
|  |  |  |  |  |  | Risk based | 611 | 611 (assumed) | 26.3 (7.1) | NR | Qatari 274 (44.8) Non-Qatari 337 (55.2) | NR | Preterm delivery 84 (13.8) PROM 18 h 50 (8.4) Intrapartum fever 26 (3.9) GBS bacteriuria 11 (1.9) Previous infant with GBS disease, n (%): 1 (0.21) | NR |
| Alarcon 2004 | To determine trends in the incidence and antimicrobial resistance of early onset sepsis caused by Escherichia coli in the era of antimicrobial prophylaxis | All new-borns with early onset E. coli infection who were born at La Paz Hospital, a University Tertiary Public Hospital, from January 1, 1992 through December 31, 2002 | NR | NR | NS SR | No strategy | NR | 28506 | NR | > 37 weeks 12/16 32-36 weeks 2/16 < 32 weeks 2/16 | NR | NR | NR by group - overall by EOS+ neonates: 41 cases of early onset E. coli infection: 27/41 (66%) maternal intrapartum temperature (38^o^C in 2/27 (7%)), 40/41 (98%) known duration of rupture of membranes, 28/41 (68%) maternal intrapartum antibiotic, 10/40 (25%) prolonged rupture of membranes (18 h) | NR |
|  |  |  |  |  |  | Screening/Universal | NR | 20262 | NR | > 37 weeks 9/20 32-36 weeks 4/20 < 32 weeks 7/20 | NR | NR | (reported in No strategy) |  |
|  |  |  |  |  |  | Risk based | NR | 35844 | NR | > 37 weeks: 3/5 32-36 weeks: 0 < 32 weeks: 2/5 | NR | NR | (reported in No strategy) |  |
| Al Luhidan 2019 | To determine the pattern of neonatal GBS infections in King Abdulaziz Medical City in Riyadh, Saudi Arabia through  identification of incidence, burden, clinical presentation and outcome of neonatal GBS infection, as well as the association of these factors with screening over 13 years period | All neonatal GBS disease cases identified through microbiology lab records within the first 90 days of life in the hospital from January 2004 to December 2016. | NR | 7-90 days | SR | Overall only, not reported by strategy | 55 | 55 (GBS+ only) | NR | Preterm 9 (16.3) Term 46 (83.6) | NR | General reporting of GBS colonisation (The colonization rates of our hospital for the last 5 years of the study ranged between 12.1% and 15.3%.) | Forty two percent (n = 23) of the neonates‚ mothers presented with some predisposing risk factors for infection transmission, detailed as follows: 16.4% (n = 9) had preterm labor, 14.5% (n = 8) presented with fever at the time of labor, 12.7% (n = 7) had prolonged premature rupture of the membrane and 5.5% (n = 3) had GBS bacteriuria during their respective pregnancy | no documented cases of chorioamnionitis |
| Angstetra 2007 | To determine the effect of institution of a universal screening protocol as per CDC 2002 guidelines had on the incidence of early-onset Group B streptococcal (GBS) and non-GBS disease in a tertiary obstetric unit. | Women who booked at JHH, Newcastle, Australia, for delivery or who were referred to JHH because of a complication with pregnancy between 1994 and June 2006 | We introduced the screening-based protocol in mid-2003 and, therefore, the data from 2003 were not included | NR | RO | Risk based | NR | 30,978 | NR | 2002 data: Gestational age (weeks) < 34: 7.2%  34 ‚ 36: 7%  37+: 85.8% | NR | 24% (Using 2004 sampling data) | NR | NR |
|  |  |  |  |  |  | Other | NR | 8303 | NR | Gestational age (weeks) < 34: 7.6% 34 ‚36: 6.8% 37+: 85.6% | NR | As above | NR | NR |
| Bauserman 2013 | We hypothesized that the widespread use of IAP has changed the incidence of GBS and E. coli as causes of early-and late-onset SBI. | All infants admitted to 322 NICUs managed by the Pediatrix Medical Group in the United States from 1997-2010 | NR | 4-120 days | NS S | Overall only, not reported by strategy | NR | 716,407 | NR | NR | Non-Hispanic White 147 (59.3) African American 48 (19.4) Asian 2 (0.8)  Hispanic 12 (4.8)  Other 39 (15.7) (Unclear which population n=248 represents (table 1)) | NR | NR | NR |
| Bekker 2014 | We assessed the clinical and molecular epidemiology of invasive group B streptococcus infection over 25 years and assess the effect of the introduction of the prevention programme in Netherlands on the incidence of neonatal  group B streptococcus disease | Patients aged 3 months or younger with positive blood culture or cerebrospinal fluid culture for group B streptococcus and Escherichia coli infection. | If no date of birth or early date of illness or age was recorded, the patient was  excluded. | 7days-3 months | NS | Overall only, not reported by strategy | NR | Group B streptococcus (n=1075) Escherichia coli (n=474) | NR | NR | NR | NR | NR | NR |
| Bizzarro 2005 | To update this database with review of neonatal sepsis cases at Y-NHH to identify longitudinal trends in demographics, pathogens, and outcome. | The medical records of all infants and new-borns with positive blood cultures obtained at any time while infants were inpatients in the NICU at Y-NHH from January 1, 1989, to December 31, 2003, as identified by microbiology laboratory culture logs | NR | 5-30 days (late, late onset (>30 days) | SR | Overall only, not reported by strategy | NR | 939 (new-borns with positive blood cultures) | NR | 31 (12) | NR | NR | NR | Birth weight |
| Bjorklund 2017 | To evaluate the effect of a rapid PCR-based GBS test on length of stay in hospital among new-borns, antibiotic use, and GBS-early-onset-disease (EOD) incidence | All term vaginally delivered live births occurred at the Kailoopisto Maternity Hospital between 1 January and 12 November 2014 | Preterm births | NR | SR | Risk based | 3028 | 3049 | NR | NR (other than term) | NR | NR | 536 | Suspected maternal infections |
|  |  |  |  |  |  | Screening/Universal | 3660 | 3681 | NR | NR (other than term) | NR | Out of the 3660 term deliveries, 843 women (23.0%) tested GBS positive, 2637 women (72.1%) tested GBS negative, and 180 women (4.9%) had invalid or no results. | 843/3660 GBS+ | Maternal infection or suspected infection |
| Bjornsdottir 2019 | We have now undertaken the investigation of the prevalence of serotypes, genetic lineages and antimicrobial resistance patterns of GBS in invasive neonatal and early infections in Iceland | All children <1 year with GBS invasive disease from 1976 to 2015 in Iceland. Inclusion criteria were GBS positive cultures from blood, cerebrospinal fluid or both, which are only performed at 2 hospital laboratories in Iceland (Landspitali University Hospital, Reykjavik; and Akureyri Hospital) | NR | 7-90 days (ultra-LOD (ULOD) >3 months of age) | NS R | Risk based | NR | NR | NR | NR | NR | NR | NR | NR |
|  |  |  |  |  |  | No strategy | NR | NR | NR | NR | NR | NR | NR | NR |
| Brozanski 2000 | To assess the effectiveness and feasibility of implementing the Centers for Disease Control and Prevention (CDC) screening-based guidelines for preventing early-onset group B streptococcal sepsis | NR | NR | NR | NS S | No strategy | 31,133 (total live births) | NR | 28.7 (6) | 38.7 (2) | 79.7% white | 24.70% | NR by strategy | NR |
|  |  |  |  |  |  | Screening/Universal | NR by strategy | NR | 29.2 (6) | 38.6 (2) | 80.7% white | 24.60% | NR by strategy | NR |
| Chan 2023 | To compare the incidences of early and late-onset neonatal sepsis, including group B streptococcus (GBS) and Escherichia coli (E. coli) before and after implementation of universal screening and intrapartum antibiotics prophylaxis (IAP). (Wang: We, therefore, aimed to determine the characteristics of our cohort of babies with EOS, whether there have been any changes in incidence and etiology of EOS as well as antibiotic resistance patterns in organisms isolated, and whether there were any differences between infants of different gestational ages) | All women who attended antenatal care in the public obstetrics units from 1 January 2009 to 31 December 2020 were included in the analysis. | Women with miscarriage before 24 completed weeks of gestation or termination of pregnancy were not included. Mothers who did not have culture-based GBS screening were not included.  Of 129 653 women who attended the MCHCs from 1 January 2012 to 31 December 2020, 21 566 (16.6%) were excluded from screening because of (1) a previously affected infant, GBS bacteriuria or known colonisation before 35weeks in the current pregnancy (n = 2497); or (2) GBS screening had already been performed at another place (n = 19 069)" | 8-120 days | SR | Screening/Universal | NR | 194 confirmed EOS | 33 confirmed EOS (IQR 30-36) | 37 (IQR 31-39) confirmed EOS | NR | 20.0% | NR by strategy but by infection status of infant | Only confirmed EOS reported by strategy (birth weight, term) |
|  |  |  |  |  |  | Risk based | NR by strategy but by infection status of infant. | Only confirmed EOS reported by strategy: 328 | Only confirmed EOS reported by strategy: 31 (IQR 27-35) | Only confirmed EOS reported by strategy: 39 (IQR 37-40) | NR |  | NR by strategy but by infection status of infant | Only confirmed EOS reported by strategy (birth weight, term) |
| Chen 2001 | To assess the impact of a risk-based approach to administering GBS intrapartum antibiotic prophylaxis at a tertiary-care hospital on the rates of EONS caused by GBS, non-GBS organisms, and antibiotic-resistant organisms. | Neonates born at the Brigham and Womens Hospital between January 1, 1990, and December 31, 1996 | Neonates born between September 1, 1992 and December 31, 1992, were not included in the analysis in order to give practitioners time to learn and practice these new guidelines | NR | NS R | No strategy | NR | 25,934 | NR | NR | General reporting (The mothers in 1990 and 1996 did not differ significantly with respect to ethnicity and insurance status) | NR | NR | 8.9% pre-term births |
|  |  |  |  |  |  | Risk based | NR | 34,262 | NR | NR | NR {The mothers in 1990 and 1996 did not differ significantly with respect to ethnicity and insurance status) | NR | NR | 8.7% pre-term births |
| Chen 2005 | To assess the rate of early-onset neonatal sepsis by antibiotic-resistant group B Streptococcus. | Data on cases of GBS EONS and antibiotic susceptibility were obtained from the hospital microbiology database | NR | NR | NS SR | No strategy | NR | 28803 | NR | NR | NR | NR | NR | NR |
|  |  |  |  |  |  | Risk based | NR | 34,262 | NR | NR | NR | NR | NR | NR |
|  |  |  |  |  |  | Screening/Universal | NR | 57877 | NR | NR | NR | NR | NR | NR |
| Cho 2019 | To analyze the most recent maternal GBS colonization rate and the changes in neonatal GBS infection rate from 2011 to 2016 | Retrospective chart audit of pregnant mothers who gave birth at Taipei Veterans General Hospital between January 1st, 2011 and June 30th, 2016 to obtain complete records of skin-to-skin contact between the mothers and their live neonates | NR | >7 days | NS S | Overall only, not reported by strategy | 9535 | 9845 | 32.8 (6.2) | 38.1 (2.2) | NR | 22.60% | 47.6% of GBS+ women had any one of the clinical risk factors | Birth asphyxia (5%), infection signs of multiple parities (4%) internal fetal monitor use (1%) |
| Clemens 2002 | To document results of a GBS prophylaxis policy at one nonacademically affiliated, community hospital and discern its effectiveness with regard to compliance as well as in decreasing the incidence of EOGBS disease. | We identified all infants, 37 weeks gestation or greater, who were born to mothers GBS positive or GBS unknown | NR | NR | NS S | No strategy | NR | NR (total births 5300) | NR | NR | NR | 21.2% | NR | NR |
|  |  |  |  |  |  | Screening/Universal | NR | NR | NR | NR | NR | NR | NR | NR |
| Coco 2002 | To compare the two strategies based on the rates of intrapartum-new-born antibiotic administration, protocol adherence, and amount of new-born laboratory testing in a family practice residency maternity service | Data were collected from delivery logs, chart reviews, and medical record reports from April 1998 to March 1999. | Births were determined to be ineligible for the study for two reasons.1 Women who gave birth before 37 weeks were eliminated from the analysis, because they would receive the same care under both protocols.2 Women who underwent elective cesarean sections were not included because their treatment would be independent of either strategy. Women undergoing nonelective cesarean sections were included | NR | SR | Screening/Universal | 171 | 171 | 22.3 (NR) | 39.8 (NR) | NR | 25% | NR | Primiparous women 39% |
|  |  |  |  |  |  | Risk based | 178 | 178 | 22.1 (NR) | 39.6 (NR) | NR | NR | 34/178 73% (25 of 34) of women received treatment for the indication of ruptured membranes for longer than 18 hours | Primiparous women 21.4% |
| Daniels 2022 | We investigated if intrapartum antibiotic prophylaxis directed by a rapid intrapartum test reduces maternal and neonatal antibiotic use, compared with usual care (i.e. risk factor-directed antibiotics), among women with risk factors for vertical group B Streptococcus transmission, and examined the accuracy and cost-effectiveness of the rapid test | A previous baby with early- or late-onset group B Streptococcus disease, as reported by the mother and documented in the maternal notes - group B Streptococcus bacteriuria during the current pregnancy, as documented in the maternal notes, regardless of whether or not the group B Streptococcus bacteriuria was treated at the time of diagnosis with antibiotics - group B Streptococcus colonisation of the vagina and/or rectum (determined from a vaginal/rectal swab) in the current pregnancy, as documented in the maternal notes - preterm labour (< 37 weeks gestation) whether suspected, diagnosed or established and whether in women with intact membranes or women with prelabour rupture of membranes of any duration - maternal pyrexia (38 degrees C) observed at any point in labour, including clinically suspected/confirmed chorioamnionitis | Women were ineligible if they were aged < 16 years, at < 24 weeks gestation, in the second stage of labour at admission or considered likely to give birth to their baby imminently, had a planned elective caesarean birth, or their baby was known to have died in utero or had a congenital anomaly incompatible with survival at birth. | 7 days-3 months | RO | Other: Rapid test in high-risk women | 722 | 749 | 29.3 (5.8) | NR | NR | Rapid test: 241/657 43% (95% CI 39 to 48%)  Selective enrichment culture: 256/619 41% (37-45%) | One risk factor: 674 (93%)  Maternal temperature38 ^O^C: 55 (8%)  Previous baby with GBS: 35 (5%) GBS in this pregnancy: 293 (41%) Preterm labour: 291 (40%)  Two risk factors: 46 (6%)  Three risk factors: 2 (< 1%) | NR |
|  |  |  |  |  |  | Risk based | 906 | 951 | 30.1 (5.8) | NR | NR | 31%, 278/906 | One risk factor: 841 (93%)  Maternal temperature >38 ^o^C: 139 (15%) Previous baby with GBS: 40 (4%) GBS in this pregnancy: 278 (31%)  Preterm labour: 384 (42%) Two risk factors: 63 (7%) Three risk factors: 2 (< 1%) | NR |
| Darlow 2016 | To determine the incidence of early-onset GBS sepsis in New Zealand five years after the publication of national risk-based GBS prevention guidelines. | Cases of EOS | The 1998-1999 survey also included detection of GBS antigen by latex agglutination on a suprapubic bladder aspiration in the definition of GBS sepsis, which accounted for 5 of 56 cases. Because of inaccuracies with this test, we did not include such cases in the 2009-2011 survey | NR | O | Overall only, not reported by strategy | 29 | 29 | 26 (range 16-42) | NR  [(72%) of infants were born at term] | Maternal ethnicity Māori: 5 (17%) Pacific Island: 7 (24%) European: 15 (52%) Other/Unknown: 2 (7%) | NR | NR | Birthweight <1500 g: 3 (10%) 1500-2499 g: 3 (10%) 2500 g: 23 (80%) Median (range) grams: 3265 (770-4405) |
| Davis 2001 | To assess the impact of new consensus guidelines issued by the Centers for Disease Control and Prevention, The American College of Obstetricians and Gynecologists, and the American Academy of Pediatrics to prevent perinatal group B streptococcal disease | All women giving birth in the 2 hospitals between October 1, 1995, and December 31, 1997 | NR | NR | RO | Other | 1337 | 1330 | NR | NR | NR | Term: 251/1123 (22.35%)  Preterm: 10/62 (16.13%) | NR | Amnionitis: 26/1337 (1.94%) Endometritis: 23/1337 (1.72%) Sepsis or bacteraemia: 2/1337 (0.15%) Urinary tract infections: 1/1337 (0.07%) |
|  |  |  |  |  |  | Risk based | 2438 | 1355 | NR | NR | NR | Term: 492/2096 (23.52%) Preterm: 21/91 (23.08%) | NR | Amnionitis: 44/2438 (1.80%)  Endometritis: 14/2438 (0.57%)  Sepsis or bacteraemia: 0/2438 (0.00%)  Urinary tract infections: 4/2438 (0.16%) |
| Eberly 2009 | To determine the effect of the revised guidelines on incidence of neonatal early-onset group B streptococcal disease via retrospective analysis of births in a military population. | All 868 260 live births from hospitals within the Department of Defence (DoD) from January 1993 through December 2007 | Infants were excluded if they were born at civilian centers and later transferred to military facilities. | 7 days-3 months | NS SR | No strategy | NR | 243,320 | NR | NR | NR | NR | NR | Term: incidence 1.89 (no data) Preterm: incidence 3.06 (no data) |
|  |  |  |  |  |  | Risk based | 362,066 | NR | NR | NR | NR | NR | NR | NR |
|  |  |  |  |  |  | Screening/Universal | NR | 262,874 | NR | NR | NR | NR | NR | Term: incidence 0.41 (no data) Preterm: incidence 1.38 (no data) |
| Ecker 2013 | To determine if changes have occurred in the causative pathogens and/or antibiotic susceptibility profiles in early onset neonatal infections since initiation of group B Streptococcus (GBS) prophylaxis and to determine risk factors for ampicillin/penicillin resistant microorganisms | Data on all positive blood, urine, and CSF cultures from infants ‚<7 days of age collected between January 1, 1990 and December 31, 2007 | Infants were excluded from the study if the culture was a virus, from an outpatient visit that did not result in a hospital admission, or interpreted as a contaminant by the treating physician. Infants were also excluded if they were born outside the study hospital. | NR | NS SR | No strategy | 105 | Total: 18,962 With early onset infection: 105 (demographics reported by infants with infection) | 24 (7) | 34 (6) | African American: 75/105 (71.4%) Caucasian: 30/105 (28.6%) Other: 0 | Positive: 13/105 (12.4%) Negative: 3/105 (2.9%) Unknown: 89/105 (84.8%) | NR | NR |
|  |  |  |  |  |  | Risk based | 71 | 13,557 With early onset infection: 71 (demographics reported by infants with infection) | 26 (7) | 31 (6) | African American: 53/71 (74.6%) Caucasian: 14/71 (19.7%) Other: 4/71 (5.6%) | Positive: 10/71 (14.1%) Negative: 28/71 (39.4%) Unknown: 33/71 (46.5%) | NR | NR |
|  |  |  |  |  |  | Screening/Universal | 44 | 9,919  With early onset infection: 44 (demographics reported by infants with infection) | 28 (6) | 34 (5) | African American: 22/44 (50.0%) Caucasian: 14/44 (31.8%) Other: 8/44 (18.2%) | Positive: 9/44 (20.5%) Negative: 21/44 (47.7%) Unknown: 14/44 (31.8%) | NR | NR |
| Edwards 2003 | To compare the relative effects of intrapartum antibiotic prophylaxis regimens on patterns of early-onset neonatal sepsis | Infants were included in the cohort if they had a positive blood culture during the first 7 days of life.  Cases with coagulase-negative staphylococci were included only if the infant received anti-staphylococcal antibiotics for at least 5 days. | Infants born elsewhere and then transferred to our center for neonatal care were excluded, as were infants whose cultures were of organisms generally considered to be contaminants (e.g. Corynebacterium sp., Bacillus sp.) | NR | SR | Risk based | NR | Total: 8287 EOS cases: 34 (demographics below by EOS cases) | NR | 34.1 (5.3) | White: 14 (41%) Black: 18 (53%) Other: 2 (6%) | NR | NR | Birth weight: (g, SD) 2375 (1118) Preterm (prior to 37 weeks): 18/34 (53%) |
|  |  |  |  |  |  | Screening/Universal | NR | Total: 8900 EOS cases: 41 (demographics below by EOS cases) | NR | 32.6 (5.8) | White: 19 (46%) Black: 18 (46%) Other: 4 (10%) | NR | NR | Birth weight (g, SD) 2019 (1236) Preterm (prior to 37 weeks): 29/41 (71%) |
| Eisenberg 2005 | We were interested in determining obstetric practice patterns with regard to the use of the screening-based approach for GBS prophylaxis | A retrospective case-cohort study design was used that included all cases of early onset GBS disease | NR | NR | SR | Risk based | 30,481 | 30,481 | NR by strategy - see below | NR | Not detailed by strategy – see below | NR | N | Not detailed by strategy – as above |
|  |  |  |  |  |  | Screening/Universal | 25,007 | 25,007 | Not detailed by strategy – see below | NR | Not detailed by strategy – see below | positive: 24% (6069) negative in 73% (18,163) unknown in 3% (775) | Not detailed by strategy – see below | NR |
|  |  |  |  |  |  | Other: Overall only, not by strategy | 63,351 | 63,351 | <20: 7255 (no data) 20-34: 46,722 (no data) 35+: 9374 (no data) | NR | White: 34,176 Black: 25,908 Other/unknown: 3267 | NR | 49,932 with no risk factors; 13,419 with at least one risk factor | NR |
| ElHelali 2019 | To assess outcomes and costs associated with around-the-clock point-of-care intrapartum group B streptococcus (GBS) polymerase chain reaction (PCR) screening | All cases of early-onset neonatal GBS disease were retrieved from the microbiology laboratory and linked to medical charts and discharge information | NR | NR | SS | Screening/Universal | 11,226 | 11,818 | NR | Term deliveries median (25th -75th percentile): 40 (39.1-40.8) | NR | 12.20% | NR | NR |
|  |  |  |  |  |  | Screening/Universal | 18,835 | 18,980 | NR | Term deliveries median (25th -75th percentile): 40 (39-40.6) | NR | 14.50% | NR | NR |
| Factor 1998 | Neonatal group B streptococcal infections can be prevented by intrapartum antibiotic prophylaxis. Beginning in 1992, women with obstetric risk factors at University of Miami Jackson Memorial Medical Center were targeted to receive intrapartum antibiotic prophylaxis. We evaluated these preventive efforts. | (Early-onset group B streptococcal disease was defined as isolation of group B streptococci from a normally sterile site (e.g., blood or cerebrospinal fluid) in an infant <7 days old born at the hospital between January 1, 1992, and December 31, 1995. Information on obstetric risk factors associated with case patients was abstracted from maternal medical records.) | NR | NR | NS R | No strategy | NR | NR  17 with EOGBS | NR | NR | participants with EOGBS:  African American, not Hispanic: 10 Hispanic: 4 White: 2 Haitian or other Caribbean: 1 | NR | Among the 17 women who were delivered of infants with EOGBS, 8 (47%) had 1 of the 3 obstetric risk factors (<37 weeks gestation, rupture of membranes for 18 hours, and intrapartum fever 38 degrees C) | % women with preterm deliveries and with prolonged rupture of membranes receiving antibiotics reported in figure 2 and 3 |
|  |  |  |  |  |  | Risk based | NR | NR | NR | NR | NR (The proportion of births to African American women declined from 33% in 1992 to 22% in 1995) | NR | Among the 12 women who were delivered of infants with EOGBS disease (50%) had 1 of these 3 obstetric risk factors | % women with preterm deliveries and with prolonged rupture of membranes receiving antibiotics reported in figure 2 and 3 |
| Garland 1991 | An evaluation of this hospital‚ GBS screening and intrapartum chemoprophylaxis policy. | Obstetric patients attending the Royal Women's Hospital between January 1981 and December 1988 | NR | NR | NS S | Screening/Universal | 30,197 | 30,197 | NR "higher percentage of women < 20 years for public [universal] patients" | NR | NR | NR | NR | NR |
|  |  |  |  |  |  | No strategy | 26,915 | 26,915 | NR “higher percentage of women < 20 years for public [universal] patients” | NR | NR | NR | NR | NR |
| Gibbs 1994 | To assess the feasibility and efficacy of a protocol for universal screening for group B streptococci combined with selective intrapartum prophylaxis at a teaching hospital | All patients receiving prenatal care at University hospital were to have rectal and vaginal cultures collected for GBS at approx. 26-28 weeks | NR | NR | NS S | No strategy | NR | NR | NR | NR | NR | NR | NR | NR |
|  |  |  |  |  |  | Screening/Universal | 3721 screened 419/687 GBS+ delivered by time of the report | NR 411 live births (1 stillborn, 7 delivered elsewhere) to 419 GBS+ women | 22 (median, no data) | NR | White: 63% Black: 21% Hispanic: 13% Asian: 3% | 18.50% | 6.4% (142/411 developed 1 or more risk factors...These 142 represent approx. 6.4% of the screened, delivered population) | NR |
| Gilson 2000 | to compare the consensus guidelines' screening-based strategy and the risk-based strategy as regards the incidence of early-onset GBS infections among term infants born at our institution. | All women who were delivered of live term infants at the University of New Mexico Hospital between October 1, 1994 and June 30, 1996. | Women and their infants with preterm (<35 weeks) labor, or preterm (<35 weeks) premature rupture of membranes | NR | SR | Screening/Universal | 2563 (total) | 420 | 25 (6) | 39.2 (1.4) | NR | 13.30% | ROM (hr): 7.2 (8.8) ROM > 18 hr: 37/420 (8.9%) Chorioamnionitis: 28/420 (6.7%) Endometritis: 11/420 (2.6%) | Other sexually transmitted disease: 28/420 (6.7%) |
|  |  |  |  |  |  | Risk based | 2684 (complete outcomes collected for sample of 407) | 407 | 24 (5.6) | 39.3 (1.4) | NR | NR | ROM (hr): 6.5 (8.3) ROM > 18 hr: 34/407 (8.4%) Chorioamnionitis: 23/407 (5.7%) Endometritis: 15/407 (3.7%) | Other sexually transmitted disease: 28/407 (6.9%) |
| Gopal Rao 2017 | To describe the impact on early-onset group B Streptococcus (EOGBS) infection rates following reversion from screening-based to risk-based intrapartum antimicrobial prophylaxis (IAP) for prevention and compared with historical controls. | Women who gave birth to live babies at the Northwick Park Hospital in the period April 2016-March 2017 (post screening period) were included in the study | There were no exclusions | NR | SR | Screening/Universal | 9801 | 9801 | 29.6 (5.3) | 39.1 (2) | Black: 950 (9.7%)  British/Irish: 849 (8.7%)  White other: 2596 (26.5%) Indian subcontinent: 4593 (48.9%) Other: 813 (8.3%) | NR | NR | NR |
|  |  |  |  |  |  | Risk based | 30,108 (combining two time periods: 25073+5035) | 30,108 | 29 (5.4) and 30.2 (5.4) | 39.1 (2.1) and 39.2 (1.7) | 2009-2013 Black: 3401 (13.6%)  British/Irish: 2665 (10.6%)  White other: 4964 (19.8%)  Indian subcontinent: 11811 (47.1%)  Other: 2232 (8.9%)   2016-2017 Black: 419 (8.3%)  British/Irish: 408 (8.1%) White other: 1538 (30.6%) Indian subcontinent: 2260 (44.9%) Other: 407 (8.1%) | NR | NR | NR |
| Gosling 2002 | Determine group B streptococcus (GBS) prevention protocols | NR | NR | NR | NS O | No strategy | NR | NR | NR | NR | NR | NR | NR | NR |
|  |  |  |  |  |  | Other: Any strategy | NR | NR | NR | NR | NR | NR | NR | NR |
| Hafner 1998 | To evaluate whether the rate of neonatal group B streptococcal infection could be reduced by screening for group B streptococci during the third trimester of pregnancy. | All women were recalled for weeks 33 through 35. When they showed up at this time, informed consent was obtained and swabs were taken from the distal third of the vagina and the rectum. | NR | NR | SR | Risk based | 3623 | 3700 | NR | NR | NR | NR | 432 (11.9%) | NR |
|  |  |  |  |  |  | Screening/Universal | 3569 | 3648 | NR | GBS pos: 40.50 weeks GBS neg: 40.24 weeks | NR | 520 (14.6%) | NR | NR |
| Hakansson 2017 | To investigate the incidence of neonatal early onset group B streptococcal (GBS) infection in Sweden after promulgation of guidelines (2008) for risk factor-based intrapartum antibiotic prophylaxis, and evaluate the presence of risk factors and obstetric management in mothers. | In infants with a purported diagnosis of early-onset GBS infection, medical records from the relevant admission were retrieved from the local hospitals. | NR | NR | NS R | No strategy | NR | 317,281 | NR | NR [Fig 4 shows overall distribution] | NR | NR | NR 127/231 with neonates who had EOGBS | NR |
|  |  |  |  |  |  | Risk based | NR | 334,593 | NR | NR [Fig 4 shows overall distribution] | NR | NR | NR 74/164 with neonates who had EOGBS | NR |
| Hong 2019 | To compare the rates of early onset neonatal sepsis (EONS) according to two different antenatal GBS screening methods- risk-based versus universal screening | A total 2,206 of pregnant women were included in the study: period 1 of risk-based approach (n=1,293; from January 2014 to April 2015) and period 2 of universal screening (n=913; from  January 2016 to April 2017). In period 2, 795 out of 913 (87%) women underwent universal GBS screening | Women with preterm delivery were excluded from this study | NR | SR | Risk based | 1293 | 1301 | 32.7(3.6) | 39.4(6.9) | NR | NR | History of neonatal GBS disease: 3/1293 (0.2) GBS bacteriuria: 1/1293 (0.1) Intrapartum temp >38 degrees C: 145/1293 (11.2) ROM>18hr before delivery: 33/1293 (2.6) | Birth weight: 3.2 (0.4) kg |
|  |  |  |  |  |  | Screening/Universal | 913 | 924 | 33.1 (3.6) | 40.6 (0.1) | NR | 7.90% | History of neonatal GBS disease: 0/913 (0) GBS bacteriuria: 0/913 (0) Intrapartum temp >38oC: 181/193 (19.8) ROM>18hr before delivery: 19/193 (2.1) | Birth weight: 3.3 (0.4) kg |
| Horvath 2013 | To assess the benefits of a chemoprophylaxis program based on screening women for group B streptococcus (GBS) infection between 30 and 32 weeks of pregnancy in a population with a high rate of premature births. | All pregnant women attending the hospital's prenatal clinic | NR | NR | NS O | No strategy | 19,722 | 19,722 | NR | NR | NR | NR | NR | 97/149 cases of neonatal GBS infection occurred in preterm new-borns |
|  |  |  |  |  |  | Other: Modified screening-based protocol (screened 30-32 weeks; IAP to GBS+ or at risk of premature) | 24,950 | 25,857 | NR | NR | NR | 23.20% | NR (overall  55/63 (87%) of women whose new-borns were infected presented 2 or more factors predicting severe neonatal GBS disease, and these included 23 women not found positive at screening) | 1/63 premature new-born was infected at birth |
| Hung 2018 | We examined the risk for Group B streptococcus (GBS)-related diseases in new-borns born to mothers who participated in a universal GBS screening program and to determine whether differences are observed in factors affecting the morbidity for neonatal early-onset GBS-related diseases | Women who had undergone GBS screening and who gave birth naturally and their new-borns between April 15, 2012 and December 31, 2013. | Women who had cesarean birth | NR | SO | Screening/Universal | 154,088 | NR | 30.89 (4.51) | NR | Taiwan: 151,899 (98.58%) Foreign: 2189 (1.42%) Aboriginal: yes: 4800 (3.12%) [unclear why this reported separately to maternal nationality] Aboriginal: no: 149,288 (96.88%) | 19.58% | NR | Preterm birth Yes: 4011 (2.60%) No: 150,077 (97.40%) |
|  |  |  |  |  |  | Other | NR | NR | NR | NR | NR | 20% | NR | NR |
| Isaacs 1999 | To determine the incidence of early onset infections caused by group B streptococcus (GBS) and other organisms in Australia and to assess intrapartum antibiotic use | Babies with systemic sepsis | NR | NR (LO sepsis defined as occurring after 48 hours) | NS O | No strategy | NR | 24,535 | NR | NR | 1991-1993 live births Non-Aboriginal: 22,819 Aboriginal: 1716 | NR | NR | NR |
|  |  |  |  |  |  | Other: Assume this means combining 1993-1995 with 1995-1997. | NR | 1993-1995:83,520 (79,240+4280) 1995-1997: 74225 (70,888+3337) Combined: 157,745 | NR | NR | 1993-1995 live births Non-Aboriginal: 79,240 Aboriginal: 4280 1995-1997 live births Non-Aboriginal: 70,888 Aboriginal: 3337 Combined live births Non-Aboriginal: 150,128 Aboriginal: 7617 | NR | NR | NR |
| Jeffery 1998 | To report the outcome of intervention to reduce early-onset group B streptococcal disease (EOGBSD) at a tertiary maternity hospital in Sydney and to review all cases of EOGBSD since intervention to improve outcomes further. | The study population included all women who booked at KGVH for delivery or who were referred to KGVH because of a complication with the pregnancy or with labor. | NR | NR | NS S | No strategy | NR | 5732 | NR | NR | General reporting (At KGVH, 2% of mothers are Aboriginal) | 12% | NR | NR |
|  |  |  |  |  |  | Screening/Universal | NR | 36,342 | NR | NR | NR (At KGVH, 2% of mothers are Aboriginal) | 10.50% | NR | NR |
| Johansson Gudjónsdóttir 2019 | The objective of the study was to evaluate data on early-onset neonatal invasive infections in western Sweden for the period 1997-2017. To identify changes in incidence, etiology and mortality and compare to previous studies from the same area starting from 1975. | All infants, within a week after birth, from whom a pathogenic organism was isolated from blood or cerebrospinal fluid (CSF) during the years 1997-2017 were included in the study | Cultures identifying commensal bacteria or species of less clear clinical significance without fulfilling the above criteria were excluded from the study | NR | NS R | Risk based | 90 (calculated from characteristics) | 90 infants with EO neonatal invasive infection | NR | <28 weeks: 25 (28%) 28-36 weeks: 23 (26%) >= 37 weeks: 42 (47%) | NR | NR | PPROM: 10 (11%) Febrile mother: 21 (23%) | NR |
|  |  |  |  |  |  | No strategy | 119 (calculated from characteristics: 119) | 119 infants with EO neonatal invasive infection | NR | <28 weeks: 12 (10%) 28-36 weeks: 37 (31%) >= 37 weeks: 70 (59%) | NR | NR | PPROM: 23 (19%) Febrile mother: 25 (21%) | NR |
| Katz 1994 | Our purpose was to evaluate and report the results of a protocol for the identification and treatment of all group B streptococcal carriers. | All pregnant women who attended clinics at the University of North Carolina Hospitals. | NR | NR | NS S | No strategy | 1977 | NR | NR | NR | NR | NR | NR (Five mothers had positive amniotic fluid cultures for group B streptococci, obtained after temperature elevation in labor.) | NR |
|  |  |  |  |  |  | Screening/Universal | 1681 | NR | NR | NR (Delivery at <37 weeks was 10% versus 13% in carriers versus noncarriers, respectively) | Black: 32% | 14% | NR (Four mothers who were not identified as group B streptococcal carriers had temperature elevations in labor, and amniotic fluid cultures grew group B streptococci. These women received antibiotics in labor because of their elevated temperatures" Preterm labor was 5.9%versus 7.7% in carriers versus noncarriers Premature rupture of the membranes was 18% versus 13% carriers versus noncarriers (p < 0.05) | GBS+: birth weight 7.2% <2500 gm GBS neg: birth weight 11% <2500 gm |
| Katz 1999 | To determine if universal Group B Streptococcus (GBS) culturing and antibiotic prophylaxis of obstetric patients decreased the incidence of neonatal early-onset GBS sepsis and mortality and maternal chorioamnionitis | We included all mothers and their infants delivering at our hospital during these years and divided them into two groups: before January 1, 1994, when there was no GBS surveillance policy in effect, and after January 1, 1994, when a formal GBS universal culturing and antibiotic prophylaxis policy was instituted | NR | 11-47 days | NS O | No strategy | 15,620 | 16,272 | NR | NR | General reporting (The University of Chicago is an urban tertiary center serving a population that is predominantly African American and indigent | NR | NR (60% of gestations with early-onset GBS sepsis had risk factors for the disease, but only one quarter of these mothers received antibiotic prophylaxis (15% of the total early-onset GBS septic infants) |  |
|  |  |  |  |  |  | Other | 8748 | 9130 | NR | NR | NR (The University of Chicago is an urban tertiary center serving a population that is predominantly African American and indigent) | 26% | NR (55% of the neonates with early-onset GBS sepsis, were noted to have risk factors, but only 45.5% of this group received antibiotic prophylaxis (30% of the total early-onset GBS septic infants)) | NR |
| Ko 2021 | Nationwide group B Streptococcus agalactiae (GBS) antepartum screening was instituted in Taiwan in 2012. The impact of the policy on early-onset sepsis (EOS) has not been evaluated. This study aimed to examine the impact of the policy on the incidence of neonatal EOS | We included neonates with culture-proven sepsis or meningitis within the first 72 h of life. | Stillborn infants and out born infants were excluded. Infants whose cultures yielded more than 2 organisms or organisms considered to be contaminants were also excluded | NR | SR | Risk based | NR | NR | NR | NR Overall: 32.7 weeks (range 22-41 weeks) | NR | NR | NR | NR |
|  |  |  |  |  |  | Screening/Universal | NR | NR | NR | NR Overall: 32.7 weeks (range 22-41 weeks) | NR | NR | NR | NR |
| Kolkman 2020 | Our study was conducted to determine adherence to three guideline-based group B streptococcus (GBS) preventive strategies | Pregnant women from 30 weeks gestation onwards were eligible for the study | Exclusion criteria for the study were age younger than 18 years, planned birth outside the participating region or language barrier | NR | ROO | Risk based | 565 | NR | Midwifery Table 2: during implementation (n=300) 28.9 (4.5) | NR | Dutch (%) 296/300 (99.0) (Midwifery Table 2) | NR | NR | NR |
|  |  |  |  |  |  | Other: Combination | 517 | NR | Midwifery Table 2: (n=256) 30.4 (4.6) | NR | Midwifery Table 2: Dutch (%) 238/256 (93.3) | NR | NR | NR |
|  |  |  |  |  |  | Other: Dutch strategy | 480 | NR | Midwifery Table 2: (n=294) 29.9 (4.5) | NR | Midwifery Table 2: Dutch (%) 285/294 (96.9) | NR | NR | NR |
| Lee 2021 | This retrospective review aimed to delineate the trend in antenatal screening for GBS colonisation at our hospital and its impact on EO-GBS sepsis rates in infants over a 15-year period | Anonymised clinical details of EO-GBS sepsis (blood-culture positive) in infants were obtained from the neonatal intensive care unit patient database | NR | NR | SR | Risk based | NR | 2001-2005: 11992 | NR | NR | General reporting on ethnicity [GBS colonisation rates among the different ethnic groups during  2010-2014 were: Chinese 22.1% (919/4,149 infants), Malay  30.8% (690/2,241 infants), Indian 29.2% (675/2,314 infants)  and other ethnicities 20.7% (338/1,631 infants). | NR | NR | NR |
|  |  |  |  |  |  | Screening/Universal | NR | 2014 and 2015: 7621 | NR | NR | NR [see above] | 2014: 26.9% 2015: 26.0% | NR | NR |
| Levine 1999 | To investigate the influence of the increased use of intrapartum chemoprophylaxis on the incidence of vertically transmitted neonatal sepsis. | Neonatal morbidity and mortality statistics and maternal data were obtained by querying our proprietary computerized perinatal database of 20,981 consecutive live births from January 1, 1992, through December 31, 1997 | NR | NR | NS O | No strategy | NR | 17,251 | NR | NR | NR | NR | NR | NR |
|  |  |  |  |  |  | Other: post CDC guidelines | NR | 3730 | NR | NR | NR | NR | NR | NR |
| Lin 2011 | To evaluate the changes of pathogens and its antibiotic susceptibility, we reviewed the incidence of EOS for the period before screening and IAP were implemented, and since 2004, after screening and IAP were implemented | All live born infants delivered at TPMMH with a birth weight more than 400 g were enrolled in this study. | NR | NR | NS S | No strategy | NR | NR (Total between 2001-2008: 32,614 2004-2008: 17,405 2001-2004: 5,209) | NR | NR | NR | NR | NR | NR |
|  |  |  |  |  |  | Screening/Universal | NR | NR Table 1: 17,405 | NR | NR | NR | Text: "culture-positive GBS rate remained at around 22% from October 2004 to November 2008" Table 1 2004: 20.54% 2005: 20.28% 2006: 19.9% 2007: 20.3% 2008: 22.9% | NR | NR |
| Locksmith 1999 | The objectives of this study were to compare maternal and neonatal infection rates under 3 different group B streptococcal prevention protocols, to determine which sub-groups of mothers and neonates benefited most from these strategies, and to evaluate the reasons for each protocol-failures in preventing neonatal disease. | We conducted a retrospective time series study including all women who were delivered of live infants at the University of Florida‚ Shands Hospital between August 1, 1991, and April 30, 1998. | NR | NR | SOO | Other: Selective/1 | 7810 | Deliveries: 7810 | <=20: 2836 (36%) 21-35: 4634 (59%) >=36: 340 4%) | NR | White: 4452 (57%) African American: 3046 (39%) Other: 312 (4%) | NR | 2082 (27%) Delivery < 37 weeks: 1561 (20%) | Chorioamnionitis:7.36% Preterm deliveries: 6.90% Term deliveries:7.57% Prolonged rupture of membranes: 23.5% Endometritis: 4.02% |
|  |  |  |  |  |  | Other: ACOG/2 | 7917 | Deliveries: 7917 | <=20: 2769 (35%) 21-35: 4738 (60%) >=36: 410 (5%) | NR | White: 4643 (59%) African American: 2797 (35%) Other: 477 (6%) | NR | 2323 (29%) Delivery < 37 weeks: 1760 (22%) | Chorioamnionitis: 7.67% Preterm deliveries: 9.30% Term deliveries:7.41% Prolonged rupture of membranes:20.3% Endometritis: 4.62% |
|  |  |  |  |  |  | Screening/Universal | 4453 | Deliveries: 4453 | <=20: 1501 (34%) 21-35: 2683 (60%) >=36: 269 (6%) | NR | White: 2622 (59%) African American: 1474 (33%) Other: 357 (8%) | NR | 1277 (29%) Delivery < 37 weeks: 1087 (24%) | Chorioamnionitis: 5.23% Preterm deliveries: 5.61% Term deliveries: 5.11% Prolonged rupture of membranes: 12.4% Endometritis: 2.76% |
| Lopez Sastre 2005 | To assess trends in the epidemiology of culture-proven and clinical neonatal sepsis of vertical transmission in the era of intrapartum antibiotic prophylaxis. | NR | NR | NR | NS S | No strategy | NR | NR | NR | NR | NR | NR | NR | NR |
|  |  |  |  |  |  | Screening/Universal | NR | NR | NR | NR | NR | NR | NR | NR |
| Lu 2022 | We aimed to explore the epidemiology and evolution of pathogens, antibiotic susceptibility, and mortality rate in cases of neonatal early-onset sepsis (EOS) reported over a period of 12 years in a level III neonatal center in Central Taiwan. | We conducted a retrospective cohort study from January 2007 to December 2018 including all infants admitted to the new-born units at China Medical University Hospital, a 90-bed level III neonatal center located in Taichung, Taiwan | We excluded stillborn infants, infants with < 23 weeks of gestation. We also excluded those with contaminated samples, including those with coagulase-negative staphylococci, micrococci, propionibacterium, corynebacterium, and diphtheroids, with a negative result on testing the second set of blood or CSF cultures drawn before antibiotic agents were administered and patients who had recovered without antibiotic use or clinical symptoms of infection | NR | NS S | No strategy | NR | NR | NR | NR | NR | NR | NR | NR |
|  |  |  |  |  |  | Screening/Universal | NR | NR | NR | NR | NR | NR | NR | NR |
| Lukacs 2012 | To describe the burden and characteristics of clinical neonatal sepsis in the United States and evaluate incidence rates after the issuance of intrapartum antibiotic prophylaxis (IAP) guidelines. | The study sample comprised hospital discharge records for hospital-born new-borns (99% of all births) and infants admitted at age <3 months. | Infants with an ICD-9-CM code of V29.0 (observation for suspected infection condition in the first 28 days of life) were excluded | 7-90 days | NS SO | No strategy | NR | NR (Sepsis hospitalisation< 3 months: 1,116,047) | NR | NR Sepsis hospitalisation< 3 months: 1,116,047 Preterm: 29.7% Preterm <28 weeks: 3.9% Preterm 28-36 weeks: 25.8% Term: 70.3% EO subgroup: 36.2% | NR | NR | NR | NR |
|  |  |  |  |  |  | Other: 1996-2001 | NR | NR Sepsis hospitalisation< 3 months: 796,633 | NR | NR Sepsis hospitalisation< 3 months: 796,633: Preterm: 35.4% Preterm <28 weeks: 6.1% Preterm 28-36 weeks: 29.3% Term: 64.6% EO subgroup: 29.9% | NR | NR | NR | NR |
|  |  |  |  |  |  | Screening/Universal | NR | NR Sepsis hospitalisation< 3 months: 608,569 | NR | NR Sepsis hospitalisation< 3 months: 608,569 Preterm: 35.8% Preterm <28 weeks: 6.0% Preterm 28-36 weeks:29.9% Term: 64.2% EO subgroup: 30.4% | NR | NR | NR | NR |
| Ma 2018 | To determine the prevalence of maternal colonization with group B streptococcus (GBS), and early onset GBS disease (EOGBSD) after implementation of universal screening. | NR | NR | NR | SR | Risk based | NR | NR | NR | NR | NR | NR | NR | NR |
|  |  |  |  |  |  | Screening/Universal | 113,989 | 122,139 | NR | NR | NR | 21.80% | NR | NR |
| Main 2000 | We compared the two different approaches to group B streptococcal screening and intrapartum chemoprophylaxis suggested by The American College of Obstetricians and Gynecologists, the American Academy of Pediatrics, and the Centers for Disease Control and Prevention: risk factor‚ based protocol and culture-based protocol. | NR | NR | NR | NS SR | No strategy | 6719 | 6829 | <20: 169 (2.5 %) 20-35: 5024 (74.8%) >35: 1526 (22.7 %) | NR <37 wks.: 704 (10.3%) | White: 2964 (44.1%) Asian: 3053(45.4%) African American: 450 (6.7%) Other: 262 (3.9%) | NR | NR | NR |
|  |  |  |  |  |  | Risk based | 12,960 | 13,270 | <20: 197 (1.5%) 20-35: 9,555 (73.7%) >35: 3208 (24.8%) | NR <37 wks.: 1289 (9.7%) | White: 7102 (54.8%) Asian: 5108 (40.6%) African American: 536 (4.1%) Other: 56 (0.5%) | NR | NR | NR |
|  |  |  |  |  |  | Screening/Universal | 9078 | 9304 | <20: 120 (1.3 %) 20-35: 6563 (72.3%) >35: 2395 (26.4%) | NR <37 wks.: 951 (10.2%) | White: 5047 (55.6%) Asian: 3651 (40.2%) African American: 318 (3.5%) Other: 62 (0.7%) | NR | NR | NR |
| Matsubara 2007 | To explore clinical protocols for the prevention of EOGBS disease of the new-born in Japan, we conducted a multicenter questionnaire survey | NR | NR | NR | NS S | No strategy | NR | 18,087 | NR | NR | NR | NR | NR | NR |
|  |  |  |  |  |  | Screening/Universal | NR | 48,891 | NR | NR | NR | NR | NR | NR |
| Matsubara 2013 | To clarify the incidence and prognosis of early-onset (EOD) and late-onset (LOD) GBS disease in Japan. To evaluate the influence of national guidelines issued in 2008 on the epidemiology of GBS disease. | NR | NR | 7-89 days | NS S | No strategy | NR | NR | NR | NR | NR | NR | NR | NR |
|  |  |  |  |  |  | Screening/Universal | NR | NR | NR | NR | NR | NR | NR | NR |
| Mirsky 2020 | Our objectives were to investigate the rate of conversion from negative to positive results in women rescreened after appropriate screening at 35-37-week gestation and to examine the impact of rescreening on the use of intrapartum antibiotics. Additionally, we examined cases of early-onset group B streptococcal sepsis (early-onset GBS) in term neonates. | Women who delivered a liveborn infant between 1 January, 2010 and 31 December, 2014 were identified. | Women were excluded from the study for the following reasons: missing date of delivery or gestational Age at birth, delivery at <22-week gestation, or delivery at one of two contract sites at which laboratory tests are not routinely captured | NR | SS | Screening/Universal | 130,906 | NR | 31 (IQR 27-34) | 39w3d, IQR 38w5d-40w2d | Asian: 7538 (24.1%) Black: 2961 (9.5%) Hispanic: 7129 (22.8%) White, non-Hispanic: 12,612 (40.4%) Unknown/other: 1006 (3.2%) | 31,246/130,906 (23.9%) | NR | NR |
|  |  |  |  |  |  | Screening/Universal | 4511 | NR | neg/pos: 29 (IQR 25-33) pos/neg: 31 (IQR 28-35) neg/neg: 31 (IQR 27-34) pos/pos: 31 (IQR 27-34) | median (IQR) neg/pos: 41w0d (40w4d-41w3d) pos/neg: 41w0d (40w3.5d-41w2d) neg/neg: 41w1d (40w4d-41w3d) pos/pos: 40w6d (40w2d-41w2d) | Neg/pos Asian: 35 (16.1%) Black: 25 (11.5%) Hispanic: 40 (18.3%) White, non-Hispanic: 114 (52.3%) Unknown/other: 4 (1.8%) Pos/neg Asian: 19 (15.8%) Black: 7 (5.8%) Hispanic: 25 (20.8%) White, non-Hispanic: 64 (53.4%) Unknown/other: 5 (4.2%) Neg/neg Asian: 542 (14.9%) Black: 203 (5.6%) Hispanic: 736 (20.2%) White, non-Hispanic: 2049 (56.3%) Unknown/other: 112 (3%) Pos/pos Asian: 90 (16.9%) Black: 32 (6%) Hispanic: 91 (17.1%) White, non-Hispanic: 301 (56.7%) Unknown/other: 17 (3.3%) | 749/4511 (16.6%) | NR | NR |
| O'Sullivan 2019 | We aimed to define the burden and clinical features of invasive group B streptococcal disease in infants younger than 90 days in the UK and Ireland, together with the characteristics of disease-causing isolates. | NR | NR | 7-89 days | NS R | Risk based | NR | 914,132 | NR | NR | NR | NR | NR [152/429 (35%) had one RCOG risk factor. This number is from 517 with EOGBS. 429/517 had additional clinical info] | NR |
|  |  |  |  |  |  | No strategy | NR | NR | NR | NR | NR | NR | NR | NR |
| Petersen 2014 | The present retrospective cohort study determines the trend over time in the rates of GBS and in demographic risk factors for GBS among pregnant women delivering at Rigshospitalet | We conducted a retrospective cohort study on all deliveries at the RH from 2002 to 2010 | NR | NR | NS R | No strategy | 125,127 (not reported directly -deliveries during 2002 and 2003 (IAP introduced 2004) | NR | NR | NR | NR | 2002: 3.3% 2003: 2.8% | NR | NR by year/strategy |
|  |  |  |  |  |  | Risk based | 437,058 (calculated - deliveries during 2004 to 2010 (IAP introduced 2004) | NR | NR | NR | NR | 2004: 3.5% 2005: 3.2% 2006: 3.2% 2007: 4.0% 2008: 4.6% 2009: 5.8% 2010: 5.8% | NR | NR by year/strategy |
| Phares 2008 | To describe disease trends among populations that might benefit from vaccination and among new-borns during a period of evolving prevention strategies. | Laboratory-confirmed invasive group B streptococcal disease identified by population-based, multistate surveillance over a recent 7-year period (1999-2005) | NR | 7-89 days | SO | Other: 1999-2001 | NR | NR | NR | NR | General ethnicity reporting: Incidence and case fatality of EOGBS by black/white reported in Fig 1 | NR | NR | NR |
|  |  |  |  |  |  | Screening/Universal | NR | NR | NR | NR | NR Incidence and case fatality of EOGBS by black/white reported in Fig 1 | NR | NR | NR |
| Poulain 1997 | To investigate the efficacy of a selective intrapartum prophylaxis of group B streptococci (GBS) infection of the neonates | NR | NR | NR | NS O | Other | 1098 | 2454 | NR |  | NR | 11% (75/668) | 11/75 GBS+: PROM 6/75: premature (no definition) 8/75: temp 38 degrees C or more 2/75: prolonged labour > 12 hours with rupture of membranes 1/75: previous GBS+ neonate infection | NR |
|  |  |  |  |  |  | No strategy | 1338 | 1338 | NR | NR | NR | NR | NR | NR |
| Puopolo 2010 | To assess the effect of maternal antibiotic exposure on neonatal early-onset sepsis (EOS) rates over an 18-year period | All infants born at the Brigham and Women Hospital (BWH) (Boston, MA) between January 1, 1990, and December 31, 2007 | NR | NR | NS SR | No strategy | NR | 25,934 | NR | NR | NR | NR | NR | Birth weight < 1500g: 890 |
|  |  |  |  |  |  | Risk based | NR | 34,934 | NR | NR | NR | NR | NR | Birth weight < 1500g: 923 |
|  |  |  |  |  |  | Screening/Universal | NR | 101,267 | NR | NR | NR | NR | NR | Birth weight < 1500g: 2293 |
| Reisner 2000 | To evaluate a group B streptococcal protocol in a large community hospital that combined treatment of high-risk patients with rapid screening of low-risk patients. | NR | NR | NR | SR | Risk based | 8188 (deliveries) | 8188 | NR | NR | NR | NR | NR | NR |
|  |  |  |  |  |  | Screening/Universal | 9932 (deliveries 1994-6) Demographics are for 1994 (3469) | 9932 | 1994: age <19: 3% of 3469 | NR | Non-white: 28.0% of 3469 | 1994: 16.6%  During study: 18% | 21.1% of 3469 | NR |
| Renner 2006 | To evaluate of the efficacy of our screening and risk-factor directed strategy | All live births at the University Womens Hospital Basel between 1997 and 2002 | NR | NR | NS O | Other: screening | NR | 9385 | NR | NR For GBS sepsis cases: 40+2 | NR | NR | NR | NR |
|  |  |  |  |  |  | No strategy | NR | 16,126 | NR | NR For GBS sepsis cases: 37 | NR | NR | NR | NR |
| Riley 2003 | To evaluate the intrapartum compliance. For the culture-based protocol, compliance was defined as culture positive receiving antibiotics or culture unavailable but risk factor positive receiving antibiotics. For the risk-based protocol, compliance was defined as risk factor positive receiving antibiotics. | We retrospectively collected data from prenatal and labor and delivery records on maternal colonization status, antepartum and intrapartum risk factors for group B streptococcal disease such as group B streptococcal bacteriuria, fever in labor, and rupture of membranes >18 hours, intrapartum antibiotic prophylaxis, and adverse drug reactions of all full-term women who labored at the academic institutions between January and February 1998. We collected the same information at the community hospital between January and March 1998 over a longer time frame because of a lower delivery volume. | We chose only full-term laboring women to avoid assessing compliance with intrapartum antibiotic prophylaxis for cesarean delivery where the guidelines are unclear. | NR | SR | Screening/Universal | 415 | NR | NR | NR | NR | 70 (16.9%) | Of 39 with unknown GBS status 8 had risk factors | NR |
|  |  |  |  |  |  | Risk based | 505 | NR | NR | NR | NR | NR | 79 (15.6%) Fever >= 100.4: 19/79 Rupture of membranes > 18 hours: 37/79 GBS bacteriuria: 28/79 Prior GBS+ infant: 1/79 | NR |
| Rottenstreich 2019 | To assess the maternal group B streptococcal (GBS) colonization rate and neonatal early-onset GBS (EOGBS) disease in term deliveries, a decade apart. | All term deliveries (37-42 weeks gestation) in SZMC  between August 2005 and December 2016 | NR | NR | SR | Screening/Universal | Not reported by epoch (overall provided in separate entry) | NR | NR | NR | NR | 2010: 36% 2011: 35% 2012: 33% 2013: 33% 2014: 31% 2015: 31% 2016: 32% | NR | NR |
|  |  |  |  |  |  | Risk based | NR by epoch (overall provided in separate entry) | NR | NR | NR | NR | 2005: 50% 2006: 53% 2007: 51% 2008: 43% 2009: 43% | NR | NR |
|  |  |  |  |  |  | Other: Overall demographics 2005-16 | 149,910 | NR | Screened: 28.51 (5.6)  Not screened: 28.97(5.8) | NR | Jewish ethnicity  Screened: 52,442 (97.3%)  Not screened: 85,221 (88.7%) | NR | NR | NR |
| Sakata 2012 | We conducted a screening study of GBS infection at our hospital over a period of 3 years from 2009 through 2011 | Medical records of pregnant women, who delivered at Asahikawa Kosei General Hospital during the 3-year period from January 2009 through December 2011, and their neonates. | NR | >3 days | NS S | Screening/Universal | 2399 | 2499 | NR | NR | NR | NR | NR | NR |
|  |  |  |  |  |  | No strategy | 2097 | 2166 | NR | NR | NR | NR | NR | NR |
| Schrag 2002 | To evaluate the effectiveness of the screening approach relative to the risk-based approach in preventing early-onset group B streptococcal disease | Infants born in 1998 and 1999 to residents of selected areas (see the Appendix) of the Active Bacterial Core Surveillance program of the Emerging Infections Program Network of the CDC. | NR | NR | SR | Screening/Universal | 2628 | 2628 | < 20: 7.6% >= 20: 92.4% | NR | Black: 22.5% White: 71.6% Other: 5.9%  Non-Hispanic: 91.3% Hispanic: 8.7% | 24% | Preterm: 7.1% Membrane rupture >=18: 8.0% Temp >=38 degrees C: 4.1% GBS bacteriuria: 3.4% GBS+ previous infant: 0.5% | NR |
|  |  |  |  |  |  | Risk based | 2515 | 2515 | < 20: 8.4% >= 20: 91.6% | NR | Black: 22.2% White: 69.0% Other: 8.8% Non-Hispanic: 87.5% Hispanic: 12.5% | NR | Preterm: 14% Membrane rupture >=18: 8.3% Temp >=38oC: 3.3% GBS bacteriuria: 1.9% GBS+ previous infant: 0.2% | NR |
| Schuchat 2002 | To determine levels of prenatal screening for several infections, intrapartum recognition of risk factors, and prophylaxis against mother-to-child transmission of group B streptococcus | A stratified random sample of 992 births was selected using the power allocation method, requiring a minimum sample of 20 births per hospital from all singleton births during 1996 in the state (n = 43,109) | NR | NR | SR | Screening/Universal | NR | NR | 7.5% of deliveries occurred to women less than 20 years of age | 9.0% of deliveries occurred preterm | Overall:  White: 79% African American: 12% Unknown: 7% Hispanic: 13% | 17% <37weeks: 31.4%; >37 weeks: 15.7% | GBS bacteriuria during the current pregnancy: 1.7% Previous infant with GBS disease: 0.31% | NR |
|  |  |  |  |  |  | Risk based | NR | NR | NR | NR | NR | NR | Delivery at <37 weeks: 9.0% ROM >18 h: 9.2% Intrapartum temperature > 38 ^o^C: 4.4% GBS bacteriuria during the current pregnancy: 1.7% Previous infant with GBS disease: 0.31% | NR |
| Share 2001 | To determine the incidence of early onset GBS in inborn babies at University Macdonald Women's Hospital before and after the implementation of the CDC/AAP/ACOG guidelines; and 2) To document compliance with the guidelines and measure the impact of the guidelines on the evaluation and treatment of neonates born to GBS colonized  women | Women identified by the Department of Reproductive Biology (at Macdonald Women's Hospital/University Hospitals of Cleveland) as GBS colonized | NR | NR | NS O | No strategy | 7531 (total deliveries) Demographics for 576 (GBS+) | NR | < 20 years: 62/576 (10.8%) | NR | Black: 213 (37.0%) White: 347 (60.2%)  Other: 9 (1.6%)  Unreported: 7 (1.2%) | 7.90% | Fever: 49 (8.5%)  UTI: 70 (12.2%) Chorioamnionitis: 43 (7.5%)  PROM: 59 (10.2%) Previously infected infant: 3 (0.3%) | NR |
|  |  |  |  |  |  | Other: July 1996-1997 | 7180 total (deliveries GBS+: 1135) | NR | < 20 years: 131/1115 (11.8%) | NR | Black: 498 (44.7%) White: 596 (53.4%)  Other: 10 (1.0%) Unreported: 11 (0.98%) | 16.1% (1996-97) | Fever: 43 (3.9%) UTI: 116 (10.4%) Chorioamnionitis: 27 (2.4%) PROM: 88 (7.9%) Previously infected infant: 4 (0.4%) | NR |
| Sutkin 2005 | To assess the effect of increased use of intravenous penicillin for group B streptococcus (Streptococcus agalactiae, GBS) antibiotic prophylaxis on non-GBS neonatal sepsis and antibiotic resistance. | All microbiology cultures originating from our NICU from 1992 to 1999 were reviewed. | NR | NR | NS S | No strategy | 30,615 | NR | NR Teen: 2478 (8.1%) | NR | African American: 5970 (19.5%) | NR | Preterm < 37 weeks: 3970 (13%) | Birth weight < 2500g: 3529 (11.5%) |
|  |  |  |  |  |  | Screening/Universal | 29,839 | NR | Teen: 2169 (7.3%) | NR | African American: 5611 (18.8%) | NR | Preterm < 37 weeks: 3496 (11.7%) | Birth weight < 2500g: 2908 (9.7%) |
| Towers 2002 | To analyze the incidence of early-onset neonatal sepsis and the presence of antibiotic resistance of the isolated bacteria and its relationship to antibiotic chemoprophylaxis that occurred during the 4 years that followed the publication of the most recent group B streptococcal guidelines | All cases of blood culture approved early-onset neonatal sepsis were collected prospectively from the nursery at Long Beach Memorial Women's Hospital. | NR | NR | NS SR | No strategy | 19891 (total deliveries) | NR | NR | NR | NR | NR | NR | NR |
|  |  |  |  |  |  | Risk based | NR | NR | NR | NR | NR | NR | NR | NR |
|  |  |  |  |  |  | Screening/Universal | NR | NR | NR | NR | NR | NR | NR | NR |
| Trijbels-Smeulders 2006 | We studied the characteristics of strains isolated from neonates with group B streptococci sepsis and meningitis, before and after the introduction of antibiotic prophylaxis in The Netherlands | Basic clinical data of 198 neonates hospitalized for GBS sepsis and/or meningitis in the Netherlands were obtained during the period 1997-199 | NR | >7 days | NS O | No strategy | NR | NR | NR | NR | NR | NR | NR | NR |
|  |  |  |  |  |  | Other | NR | NR | NR | NR | NR | NR | NR | NR |
| Trijbels-Smeulders 2007 | To describe the epidemiology of neonatal group B streptococcal (GBS) disease over five years (1997-2001) in the Netherlands, stratified for proven and probable sepsis and for very early (<12 h), late early (12 h-<7 days) and late (7-90 days) onset sepsis. (2) To evaluate the effect of the introduction in January 1999 of guidelines for prevention of early onset GBS disease based on risk factors | Proven and probable cases of GBS disease during the first three months of life, diagnosed in the preceding month. | NR | 7-90 days | NS O | No strategy | NR | 378 with proven/probable GBS sepsis | NR | NR | NR | NR | preterm delivery: 54/128; 42% | NR |
|  |  |  |  |  |  | Other: Dutch strategy | NR | 564 with proven/probable GBS sepsis | NR | NR | NR | NR | preterm delivery: 40/140; 29% | NR |
| Trollfors 2022 | To evaluate the total burden of invasive GBS infections in terms of incidence, hospitalizations, manifestations, risk factors, and short-term mortality. | All patients from whom GBS was isolated in blood, cerebrospinal fluid (CSF), synovial fluid, pleural fluid, peritoneal fluid, pericardial fluid, and corpus vitreum during 2003-2016, in the region of Vastra Gataland, southwest Sweden, during 2003-2016 | NR | 7-27 days | NS R | No strategy | NR | NR Overall: 196 neonates with GBS | NR | NR | NR | NR | NR Overall: Preterm: 68/196 PROM: 15/196 Febrile mother at birth: 10/196 | Down syndrome: 3/196 Congenital malformation: 2/196 |
|  |  |  |  |  |  | Risk based | NR | NR Overall: 196 neonates with GBS | NR | NR | NR | NR | NR Overall: Preterm: 68/196 PROM: 15/196 Febrile mother at birth: 10/196 | Down syndrome: 3/196 Congenital malformation: 2/196 |
| Uy 2002 | To determine the incidence of EOGBS infection and the association between changes in the incidence and intrapartum antibiotic prophylaxis (IAP). | All infants delivered at SMH from January 1, 1985 to December 31, 1998 with GBS isolated from blood and/or CSF at less than 7 days of age were included in this retrospective study. | NR | NR | NS RO | No strategy | 320 | NR | NR | NR Overall in 56 cases >= 37 weeks: 29 (52%) < 37 weeks: 27 (48%) | Overall in 56 cases: Black: 23 (41%) White: 30 (54%) Hispanic: 2 (4%) Asian: 1 (2%) | NR | 64/320 | Chorioamnionitis: 10/300, 3.3% |
|  |  |  |  |  |  | Other: 1993-95 AAP | 151 | NR | NR | NR Overall in 56 cases >= 37 weeks: 29 (52%) < 37 weeks: 27 (48%) | NR Overall in 56 cases: Black: 23 (41%) White: 30 (54%) Hispanic: 2 (4%) Asian: 1 (2%) | NR | 30/151 | Chorioamnionitis: 4/150, 2.7% |
|  |  |  |  |  |  | Risk based | 150 | NR | NR | NR Overall in 56 cases >= 37 weeks: 29 (52%) < 37 weeks: 27 (48%) | NR Overall in 56 cases: Black: 23 (41%) White: 30 (54%) Hispanic: 2 (4%) Asian: 1 (2%) | NR | 41/150 | Chorioamnionitis: 6/150 4.0% |
| Van den Hoogen 2010 | To identify longitudinal trends in causative microorganisms for neonatal sepsis and analyze antibiotic susceptibility of all blood isolates of infants with sepsis | Data of all infants admitted to the NICU of the Wilhelmina Children's Hospital, University Medical Centre, Utrecht, the Netherlands, between January 1, 1978, and December 31, 2006, were studied retrospectively | NR | NR (LOS was defined as clinical signs of infection and a positive blood culture obtained after 48 h of life) | NS O | No strategy | NR | NR Number of GBS positive cases of early onset sepsis in infants admitted to NICU from 1978-1997: 94/6742 | NR | NR | NR | NR | NR | NR |
|  |  |  |  |  |  | Other: Dutch strategy > 1998 | NR | NR Number of GBS positive cases of early onset sepsis in infants admitted to NICU from 1998-2006: 31/4660 | NR | NR | NR | NR | NR | NR |
| VanDyke 2009 | Assess the implementation of the 2002 screening and chemoprophylaxis guidelines, examine missed opportunities for the prevention of GBS disease, and characterize the remaining burden of early-onset group B streptococcal disease to identify areas that might benefit from additional public health prevention measures. | The target study population was infants born alive to surveillance-area residents who delivered at area hospitals at which there were 10 births per year or more during 2003 and 2004. [All cases of GBS disease that occurred in the birth cohort were included; and random sample of 7737 live births which were GBS neg] | Our study design excluded women with a risk of preterm delivery whose  labor was successfully arrested | NR | SO | Screening/Universal | 7691 | 7691 | < 20: 8.7% (95% CI 7.9 - 9.5) | NR Preterm delivery 11.0% (95% CI 10.0-12.1) | Race White: 67.0% (95% CI 65.7-68.2) Black: 20.3% (95% CI 19.2-21.4) Other: 12.1% (95% CI 11.3-13.0) Unknown:0.6% (95% CI 0.5-0.8)  Ethnic group  Hispanic: 18.0% (95% CI 17.0-18.9) Non-Hispanic: 80.5% (95% CI 79.5-81.5) Unknown:1.6% (95% CI 1.3-1.9) | 24.2% (95% CI 23.0-25.5) | Preterm delivery: 11.0% (95% CI 10.0-12.1) Threatened preterm delivery: 5.3% (95% CI 4.7-6.0) Rupture of membranes 18 hr before delivery: 7.2% (95% CI 6.5-8.0) Intrapartum temperature 38.0 degrees C: 3.3% (95% CI 2.8-3.8) Suspected chorioamnionitis: 3.1% (95% CI 2.6-3.7) Previous infant with invasive group B streptococcal disease: 1.3% (95% CI 1.0-1.7) Group B streptococcal bacteriuria during current pregnancy: 5.5% (95% CI 4.9-6.2) | NR |
|  |  |  |  |  |  | Other: Screening during 1998-1999 (pre-universal) | NR | NR | NR | NR | NR | NR | NR | NR |
| Vergani 2002 | To evaluate the effect of different prevention strategies on the rate of early-onset neonatal group B streptococcus (GBS) disease and mortality. | NR | Cases of clinical suspicion of sepsis in the absence of positive cultures were not included in the analysis. | NR | NS SR | No strategy | 8573 | 8573 | <20: 48(1%) 20-35: 7043 (82%)  >35: 1482 (17%) | NR | NR | General report of colonisation (The yearly prevalence of maternal GBS carriers did not change significantly during the period in which screening was performed, with an average of 18% (range 16 to 19%)) | 1397/8573 (16.3%) | NR |
|  |  |  |  |  |  | Screening/Universal | 13,754 | 13,754 | <20: 82 (1%) 20-35: 10,728 (78%) >35: 2943 (21%) | NR | NR | NR As above | 2182/13,754 (15.9%) | NR |
|  |  |  |  |  |  | Risk based | 10,303 | 10,303 | <20: 62 (1 %) 20-35: 8356 (81 %) >35: 1885(18%) | NR | NR | NR As above | 1732/10,303 (16.8%) | NR |
| Wicker 2019 | To detect and analyze potential changes in the incidence of invasive infant GBS infections before (2001-2003) versus after (2009-2010) the implementation of the revised guidelines for prevention of infant GBS disease in Germany. | We collected and matched data regarding invasive GBS infections from 2 separate, independent sources | NR | 7-90 days | SO | Screening/Universal | NR | NR | NR | NR | NR | NR | NR | NR |
|  |  |  |  |  |  | Other: 2001-2003 (pre-universal screening recommendation) | NR | NR | NR | NR | NR | NR | NR | NR |
| Youden 2005 | To determine the level of compliance with GBS testing recommendations and to determine women's knowledge of, attitudes towards, and beliefs about prenatal screening for this infection. | Women who gave birth to a live infant at the IWK Health Centre during a seven-week period July 12, 2004 to August 23, 2004) were offered enrolment in the study on the day after delivery. | Exclusion criteria included having an infant in the neonatal intensive care unit and an inability to speak English. | NR | SR | Screening/Universal | 67 | 67 | Not detailed by strategy – see below | NR | Not detailed by strategy – see below | 19.4% (95% CI 10.8-30.9) | NR | NR |
|  |  |  |  |  |  | Risk based | 206 | 206 | Not detailed by strategy – see below | NR | Not detailed by strategy – see below | NR | 25.2% (95% CI 19.5-31.7) | NR |
|  |  |  |  |  |  | Other: Overall demographics reported | 273 | 273 | Under 18: 1.4% (95% CI 0.4-3.6)  18-24: 17.6% (95% CI 13.3-22.5)  24-34: 63.8% (95% CI 57.9-69.4)  35-44: 17.2% (95% CI 13.0-22.2) | NR | White: 90.3% (95% CI 86.2-93.5)  Black: 2.5% (95% CI 1.0-5.1)  Asian: 3.2% (95% CI 1.5-6.0)  First Nations: 1.4% (95% CI 0.4-3.6) | NR | NR | NR |

**Abbreviations:** EOGBS: early onset GBS; GBS: Group B streptococcus; IQR: interquartile range, LOGBS: late onset GBS; NI: no information; NR: not reported, PROM: premature rupture of membranes; PTL: preterm labour; RCOG: Obstetricians and Gynaecologists; ROM: rupture of membrane

***Approach / Strategy comparison:** NS: no strategy, S: universal screening, R: risk-based approach; O: other
