# Supplementary material for: Benefits and Harms of Antenatal/Intrapartum Screening for Maternal Group B Streptococcus and Use of Intrapartum Antibiotic Prophylaxis Versus Risk‐Based Protocols or No Intervention: A Rapid Review
Source: Acta Paediatr. 2026 Apr 30;115(8):1598–610. doi: 10.1111/apa.70568 (PMC13371836; doi:10.1111/apa.70568)
Supplement: Supplementary file 16 — Data S16: Strategies compared in primary studies. [file APA-115-1598-s004.docx]

## File 16 (S16) Strategies compared in primary studies

| **No. of studies** | **Strategy 1** | **Strategy 2** | **Strategy 3** | **Studies** |
| --- | --- | --- | --- | --- |
| 21 | Screening/ Universal | Risk Based |  | Abdelmaaboud 2011, AlLuhidan 2019, Bizzarro 2005, Bjorklund 2017, Chan 2023, Coco 2002, Edwards 2003, Eisenberg 2005, Gilson 2000, GopalRao 2017, Hafner 1998, Hong 2019, Ko 2021, Lee 2021, Ma 2018, Reisner 2000, Riley 2003, Rottenstreich 2019, Schrag 2002, Schuchat 2002, Youden 2005 |
| 15 | Screening/ Universal | No Strategy |  | Bauserman 2013, Brozanski 2000, Clemens 2002, Cho 2019, Garland 1991, Gibbs 1994, Jeffery 1998, Katz 1994, Lin 2011, LopezSastre 2005, Lu 2022, Matsubara 2007, Matsubara 2013, Sakata 2012, Sutkin 2005 |
| 8 | Risk Based | No Strategy |  | Bjornsdottir 2019, Chen 2001, Factor 1998, Hakansson 2017, JohanssonGudjónsdóttir 2019, O'Sullivan 2019, Petersen 2014, Trollfors 2022 |
| 4 | Screening/ Universal | Other Strategy |  | Hung 2018, Phares 2008, VanDyke 2009, Wicker 2019 |
| 3 | Risk Based | Other Strategy |  | Angstetra 2007, Daniels 2022, Davis 2001 |
| 2 | Screening/ Universal (1) | Screening/ Universal (2) |  | ElHelali 2019, Mirsky 2020 |
| 13 | No Strategy | Other Strategy |  | Bekker 2014, Darlow 2014, Gosling 2002, Horvath 2013, Isaacs 1999, Katz 1999, Levine 1999, Poulain 1997, Renner 2006, Share 2001, Trijbels-Smeulders 2006, Trijbels-Smeulders 2007, vandenHoogen 2010 |
| 8 | Screening/Universal | Risk Based | No Strategy | Alarcon 2004, Chen 2005, Eberly 2009, Ecker 2013, Main 2000, Puopolo 2010, Towers 2002, Vergani 2002 |
| 1 | Screening/Universal | Other Strategy | No Strategy | Lukacs 2012 |
| 1 | Risk Based | Other Strategy | No Strategy | Uy 2002 |
| 1 | Risk Based | Other   Strategy (1) | Other Strategy (2) | Kolkman 2020 |
| 1 | Screening/Universal | Other   Strategy (1) | Other Strategy (2) | Locksmith 1999 |
