# Supplementary material for: Benefits and Harms of Antenatal/Intrapartum Screening for Maternal Group B Streptococcus and Use of Intrapartum Antibiotic Prophylaxis Versus Risk‐Based Protocols or No Intervention: A Rapid Review
Source: Acta Paediatr. 2026 Apr 30;115(8):1598–610. doi: 10.1111/apa.70568 (PMC13371836; doi:10.1111/apa.70568)
Supplement: Supplementary file 17 — Data S17: IAP exposure: primary study level. [file APA-115-1598-s009.docx]

## Supplementary materials File 17 (S17). IAP exposure: primary study level

Table showing percentage of pregnant women receiving intrapartum antibiotic exposure by strategy

| **Review** | **Authors/primary study** | **Percentage of pregnant women receiving IAP (%, 95% CI)** | | | |
| --- | --- | --- | --- | --- | --- |
|  |  | **No strategy** | **Risk based** | **Screening / Universal strategy** | **Other strategy** |
| Panneflek 2024 | Björklund 2017 |  | 27.1 (25.5-28.7) | 27.9 (26.5-29.4) |  |
| Panneflek 2024 | Chan 2023^1^ |  | 8.1 (8.0-8.2) | 24.0 (23.8-24.1) |  |
| Panneflek 2024 | Coco 2002 |  | 18.5 (13.5-24.9) | 20.5 (15.1-27.2) |  |
| Newly identified | Daniels 2022 |  | 36 (no data) |  | 41 (no data) |
| Panneflek 2024 | Davis 2001 |  |  | 26.7 (25.0-28.5) | 12.8 (11.1-14.7) |
| Panneflek 2024 | Hafner 1998 |  | 11.9 (10.9-13.0) | 13.5 (12.5-14.6) |  |
| Panneflek 2024 | Hong 2019 |  | 10.9 (9.3-12.7) | 21.5 (18.9-24.3) |  |
| Panneflek 2024 | Katz 1999 | 15.7 (15.1-16.3) |  |  | 30 (no data)^2^ |
| Panneflek 2024 | Levine 1999 | 3.0 (27.6-32.7) |  |  | 16.0 (14.9-17.2) |
| Panneflek 2024 | Locksmith 1999 (i) |  |  | 21.0 (19.8-22.2) | 9.4 (8.8-10.1) |
| Panneflek 2024 | Locksmith 1999 (ii) |  |  | 21.0 (19.8-22.2) | 12.9 (12.2-13.7) |
| Hasperhoven 2020 | Main & Slagle 2000 |  | 25 (% all pregnant women) | 26 (% all pregnant women) |  |
| Newly identified | Riley 2003^3^ |  | 14.3 (no data) | 16.1 (no data) |  |
| Hasperhoven 2020 | Schrag 2002 |  | 29 (% all pregnant women) | 31 (% all pregnant women) |  |
| Panneflek 2024 | Schrag 2002 |  | 14.6 (13.3-16.1) | 21.4 (19.8-23.0) |  |
| Panneflek 2024 | Schushat 2002 |  | 9.8 (6.9-13.8) | 13.3 (10.0-17.3) |  |
| Panneflek 2024 | Uy 2002 | 10.0 (7.2-13.8) | 20.7 (14.9-27.9) |  | 7.3 (4.1-12.7) |
| Panneflek 2024 | van Dyke 2009 |  |  | 31.7 (30.7-32.8) | 26.8 (25.4-28.2) |
| Hasperhoven 2020 | Vergani 2002 |  | 16.8 (% all pregnant women) ^2^ | 28 (% all pregnant women) |  |
| Panneflek 2024 | Vergani 2002 |  | 16.8 (16.1-17.6) |  | 35.8 (35.0-36.6) |
| Panneflek 2004 | Youden 2005 |  | 23.8 (18.5-30.1) | 14.9 (8.2-25.6) |  |

**Abbreviations:** CI: confidence interval, IAP: intrapartum antibiotic anaphylaxis

^1^Reported as Wang 2023 in Panneflek 2024
^2^No data reported in review, extracted from original paper for GBS strategy period
^3^Data newly extracted, excluded from Panneflek 2024 (no outcomes of interest)
