# Supplementary material for: Benefits and Harms of Antenatal/Intrapartum Screening for Maternal Group B Streptococcus and Use of Intrapartum Antibiotic Prophylaxis Versus Risk‐Based Protocols or No Intervention: A Rapid Review
Source: Acta Paediatr. 2026 Apr 30;115(8):1598–610. doi: 10.1111/apa.70568 (PMC13371836; doi:10.1111/apa.70568)
Supplement: Supplementary file 18 — Data S18: Timing of determination: summary of meta‐analysis and GRADE judgements. [file APA-115-1598-s014.docx]

## Supplementary materials File 18 (S18). Timing of determination: summary of meta-analysis and GRADE judgements

### File 18.1. Timing of determination (screening timepoint)

Timing of screening as reported by the included high-quality systematic reviews to compare late versus early antenatal universal screening or antepartum versus intrapartum universal screening.

| **Review (Author, year)** | **Comparison** | **Population** | **Outcome** | **Number of studies (primary studies included in MA)** | **n (total)** | **Effect size (95% confidence interval)** | **Direction of effect** | **GRADE LEVEL (as reported by SR authors)** | **a. ROB, b. Inconsistency, c. Indirectness, d. Imprecision, e. Publication bias (Report downgrades applied by the SR authors)** | **Finding as reported by review authors (verbatim)** |
| --- | --- | --- | --- | --- | --- | --- | --- | --- | --- | --- |
| Panneflek 2024 | Late antenatal universal screening strategy vs early antenatal universal screening strategy | Pregnant women | EOGBS timing of determination (early vs. late antenatal*) | 1 study  (Vergani 2002) | 13,754 | RR 1.24, 95% CI 0.25 to 6.12    Heterogeneity (I^2^) = NA | No harm or benefit | Very-low | a. Study at serious risk of bias. b. Only one study c. A combination strategy was used instead of a universal strategy. d. The 95%-CI is wide and includes a RR of 1.0. Sample size is not sufficiently large to detect a precise effect e. Only one study. | Vergani et al reported no significant differences in  EOGBS infection between early and late antepartum  determination |
| Panneflek 2024 | Intrapartum universal screening strategy vs Antepartum universal screening strategy | Pregnant women | EOGBS timing of determination (intrapartum vs. antepartum) | 1 study  (El Helali 2019) | 30,798 | RR 0.21, 95% CI 0.07 to 0.64    Heterogeneity (I^2^) = NA | Favours intrapartum determination | Moderate | a. Study at moderate risk of bias. b. Only one study c. Not applicable in this review, because all studies include report a direct comparison. d. The 95%-CI is relatively narrow and excludes a RR of 1.0. Although small sample size, no downgrade, because effect large enough. e. Only one study. | El Helali  et al reported that intrapartum determination significantly reduced EOGBS infection compared to antepartum determination |

Abbreviations: EOS: early-onset sepsis, EOS-GBS: early-onset sepsis Group B Streptococcus, NOS: Newcastle Ottawa Scale, NS: not significant, ROB: risk of bias, RR: risk ratio, SR: systematic review
* early screening: 26-28 weeks, late screening: 35-37 weeks

**GRADE Working Group grades of evidence**
High quality: Further research is very unlikely to change our confidence in the estimate of effect.
Moderate quality: Further research is likely to have an important impact on our confidence in the estimate of effect and may change the estimate.
Low quality: Further research is very likely to have an important impact on our confidence in the estimate of effect and is likely to change the estimate.
Very low quality: We are very uncertain about the estimate.
