# Supplementary material for: Benefits and Harms of Antenatal/Intrapartum Screening for Maternal Group B Streptococcus and Use of Intrapartum Antibiotic Prophylaxis Versus Risk‐Based Protocols or No Intervention: A Rapid Review
Source: Acta Paediatr. 2026 Apr 30;115(8):1598–610. doi: 10.1111/apa.70568 (PMC13371836; doi:10.1111/apa.70568)
Supplement: Supplementary file 19 — Data S19: Timing of maternal GBS screening: primary study level. [file APA-115-1598-s019.docx]

## Supplementary materials File 19 (S19). Timing of maternal GBS screening at primary study level

Table showing timing of maternal GBS screening as reported by primary studies. Guidelines cited are reported when no specific time in weeks was described. Data were extracted from the included high-quality systematic reviews when available.

| **Review** | **Primary study** | **Maternal GBS screening strategy timing (GA weeks)** | | |
| --- | --- | --- | --- | --- |
|  |  | **Universal screening (1)** | **Universal screening (2)** | **Other screening** |
| Li 2020 | Abdelmaaboud 2011 | 35–37 |  |  |
| Panneflek 2024 | Abdelmaaboud 2011 | 35–37 |  |  |
| Panneflek 2024 | Alarcon 2004 | CDC 1996 |  |  |
| Panneflek 2024 | Al Luhidan 2019 | 35–37 |  |  |
| Li 2020 | Angstetra 2007 | 34-37 |  |  |
| Panneflek 2024 | Angstetra 2007 |  |  | 34–37 |
| Hasperhoven 2020 | Angstetra 2007 | 34–37 |  |  |
| Panneflek 2024 | Bauserman 2013 | CDC 2002 |  |  |
| Li 2020 | Bjorklund 2017 | IP PCR |  |  |
| Panneflek 2024 | Björklund 2017 | IP PCR |  |  |
| Panneflek 2024 | Brozanski 2000 | CDC 1996 |  | 26-28 |
| Panneflek 2024 | Chan 2023 | 35-37 |  |  |
| Hasperhoven 2020 | Chen 2005 | 35-37 |  |  |
| Panneflek 2024 | Chen 2005 | 35-37 |  |  |
| Li 2020 | Chen 2005 | 35-37 |  |  |
| Panneflek 2024 | Cho 2019 | CDC 2010 |  |  |
| Panneflek 2024 | Clemens 2002 | CDC 1996 |  |  |
| Panneflek 2024 | Coco 2002 | 35+ |  |  |
| Newly identified | Daniels 2022 |  |  | IP PCR |
| Panneflek 2024 | Davis 2001 | CDC 1996 |  | AAP 1992 |
| Panneflek 2024 | Eberly 2009 | CDC 2002 |  | 35-37 |
| Panneflek 2024 | Ecker 2013 | CDC 2002 |  |  |
| Hasperhoven 2020 | Edwards 2003 | CDC 1996 |  |  |
| Panneflek 2024 | Edwards 2003 | CDC 1996 |  |  |
| Li 2020 | Eisenberg 2005 | 35-37 |  |  |
| Panneflek 2024 | El Helali 2019 | 35-37 | IP PCR |  |
| Panneflek 2024 | Garland 1991 | 32 |  |  |
| Panneflek 2024 | Gibbs 1994 | 26-28 |  |  |
| Li 2020 | Gilson 2000 | 35-37 |  |  |
| Panneflek 2024 | Gilson 2000 | 35-37 |  |  |
| Hasperhoven 2020 | Gopal Rao 2017 | 35-37 |  |  |
| Panneflek 2024 | Gopal Rao 2017 | 35-37 |  |  |
| Li 2020 | Hafner 1998 | 33-35 |  |  |
| Panneflek 2024 | Hafner 1998 | 33-35 |  |  |
| Panneflek 2024 | Hong 2019 | CDC 2010 |  |  |
| Panneflek 2024 | Horváth 2013 |  |  | 30-32 |
| Hasperhoven 2020 | Hung 2018 | 35-37 |  |  |
| Panneflek 2024 | Hung 2018 | 35-37 |  |  |
| Panneflek 2024 | Jeffery 1998 | 28 |  |  |
| Panneflek 2024 | Katz 1994 | 24-28 |  |  |
| Panneflek 2024 | Katz 1999 |  |  | 28 |
| Panneflek 2024 | Ko 2021 | 35-37 |  |  |
| Newly identified | Kolkman 2020 |  |  | 35-37 |
| Panneflek 2024 | Lee 2021 | 35-37 |  |  |
| Panneflek 2024 | Lin 2011 | 35-37 |  |  |
| Li 2020 | Locksmith 1999 | 35-37 |  |  |
| Panneflek 2024 | Locksmith 1999 | CDC 1996 |  |  |
| Panneflek 2024 | López Sastre 2005 | 35-37 |  |  |
| Panneflek 2024 | Lu 2022 | 35-37 |  |  |
| Panneflek 2024 | Lukacs 2012 | 35-37 |  | 35-37 |
| Hasperhoven 2020 | Ma 2018 | 35-37 |  |  |
| Panneflek 2024 | Ma 2018 | 35-37 |  |  |
| Hasperhoven 2020 | Main 2000 | 35-37 |  |  |
| Li 2020 | Main 2000 | 35-37 |  |  |
| Panneflek 2024 | Main 2000 | 35-37 |  |  |
| Panneflek 2024 | Matsubara 2013 | 33-37 |  |  |
| Panneflek 2024 | Matsubara 2007 | 33-37 |  |  |
| Newly identified | Mirsky 2020 | 35-37 | 36-38 (rescreen) |  |
| Hasperhoven 2020 | Phares 2008 | CDC |  |  |
| Panneflek 2024 | Phares 2008 | CDC 2002 |  | CDC 1996 |
| Panneflek 2024 | Poulain 1997 |  |  | 28 |
| Panneflek 2024 | Puopolo 2010 | 35-37 |  |  |
| Li 2020 | Reisner 2000 |  |  |  |
| Panneflek 2024 | Renner 2006 |  |  | 35-37 |
| Panneflek 2024* | Riley 2003 | CDC 1996 |  |  |
| Panneflek 2024 | Rottenstreich 2019 | 35-37 |  |  |
| Panneflek 2024 | Sakata 2012 | 33-37 |  |  |
| Li 2020 | Schrag 2002 | 35-37 |  |  |
| Panneflek 2024 | Share 2001 |  |  | ACOG 1996 |
| Panneflek 2024 | Sutkin 2005 | CDC 1996 |  | 26-28 |
| Panneflek 2024 | Towers 2002 |  |  | 35-37 |
| Panneflek 2024 | Trijbels-Smeulders 2007 |  |  | Dutch guidelines 1999 |
| Panneflek 2024 | Trijbels-Smeulders 2006 |  |  | Dutch guidelines 1999 |
| Panneflek 2024 | Uy 2002 |  |  | ACOG 1992 |
| Panneflek 2024 | van den Hoogen 2010 |  |  | Dutch guidelines 1999 |
| Hasperhoven 2020 | Vergani 2002 | 26-28 and 35-37 |  |  |
| Li 2020 | Vergani 2002 | 26-28 and 35-37 |  |  |
| Panneflek 2024 | Vergani 2002 |  |  | 26-28 and 35-37 |
| Panneflek 2024 | Wicker 2019 | 35-37 |  | 35-37 |
| Panneflek 2024 | Youden 2005 | 35-37 |  |  |

**Abbreviations:** ACOG: American College of Obstetricians and Gynecologists; AAP: American Academy of Pediatrics, CDC: Centers for Disease Control and Prevention, GA: gestational age, IP: intrapartum, PCR: polymerase chain reaction test

*Excluded from Panneflek 2024 (no outcomes of interest)
