# Supplementary material for: Benefits and Harms of Antenatal/Intrapartum Screening for Maternal Group B Streptococcus and Use of Intrapartum Antibiotic Prophylaxis Versus Risk‐Based Protocols or No Intervention: A Rapid Review
Source: Acta Paediatr. 2026 Apr 30;115(8):1598–610. doi: 10.1111/apa.70568 (PMC13371836; doi:10.1111/apa.70568)
Supplement: Supplementary file 20 — Data S20: Meningitis: Neonatal health outcomes. [file APA-115-1598-s002.docx]

## Supplementary materials File 20 (S20). Septicaemia and meningitis: neonatal health outcomes at primary study level

Tables describe meningitis as reported by primary study and presented by strategy. Studies may appear in more than one table if multiple strategies were used.

### File 20.1 Universal screening versus no strategy: septicaemia and meningitis

| **Review** | **Authors** | **Country** | **Is the outcome reported for separate screening groups?** | **No policy strategy** | **Risk based** | **Screening / Universal strategy** | **Other strategy** | **If outcome data not reported separately, provide details here** | **Is the outcome reported at the level of the neonate / infant / child or maternal.** | **Other details about time frames** | **Is the outcome reported at short-term, medium-term or longer term?** | **Comments** |
| --- | --- | --- | --- | --- | --- | --- | --- | --- | --- | --- | --- | --- |
| Panneflek 2024 | Eberly 2009 | USA | No |  |  |  |  | Meningitis: With bacteremia or sepsis: 34/860 cases of early onset GBS; Meningitis alone: 16/860 cases of early onset GBS | Neonate <7 days |  | Short-term outcome |  |
| Panneflek 2024 | Garland 1991 | Australia | No |  |  |  |  | From Garland "of the 16 GBS infections, 8 were cases born at or before 32 weeks gestation. Of these 5 had septicaemia and 3 were pneumonia. Of the 8 infections occurring after 32 weeks, 3 cases had septicaemia, 4 cases had pneumonia, and 1 case had speticaemia/pneumonia). Of the 8 infections occurring after 32 weeks' gestation, 5 resulted from failure to follow the preventative protocol and the remained resulted from maternal sepsis" | Neonate <7 days |  |  |  |
| Panneflek 2024 | Lu 2022 | Taiwan | No |  |  |  |  | "most infants with EOS/ early onset meningitis survived to discharge, 23.9% died (17/71); of those, 82% (14/17) died within the first 7 days of life" | Neonate <7 days |  |  |  |
| Panneflek 2024 | Lukacs 2012 | USA | Yes | Bacterial meningitis: 0.6% (n=1116047) |  | Bacterial meningitis: 0.9% (n=608569) | Bacterial meningitis: 0.9% (n=796633) | Bacterial meningitis based on sepsis hospitalisation in infants aged < 3 months | Other (please specify) | Characteristics of sepsis hospitalisation in infants <3 months | Other | Among the 12% of sepsis hospitalizations that involved a pathogen-specific ICD-9-CM code (Table I and Appendix), 38% were due to Streptococcus spp (9% identified as GBS). There were no significant differences in the percentage of hospitalizations with meningitis or respiratory failure, the percentage of fatal cases, or geographic distribution.  LOGBS definition: late-onset sepsis: up to 90 days of age and after first week of life. Authors note that "we could not determine the date of onset of sepsis to distinguish early-onset sepsis from late-onset sepsis, if more than one episode of sepsis occurred during a single newborn hospital course or if an infant was hospitalized more than once" |
| Panneflek 2024 | Matsubara 2013 | Japan | No |  |  |  |  | 24/88 cases of EOD | Neonate <7 days |  |  |  |
| Panneflek 2024 | Matsubara 2013 | Japan | No |  |  |  |  | 85/162 cases of LOD with meningitis | Other (please specify) | Late-onset disease |  |  |

**Abbreviations**: EOD: early onset disease, EOGBS: Early-Onset Group B Streptococcal Disease, EOS: early onset sepsis, LOD: late onset disease, LOGBS: late onset Group B Streptococcal Disease
Studies may be reported in multiple tables

### File 20.2 Risk based versus no strategy: septicaemia and meningitis

| **Review** | **Authors** | **Country** | **Is the outcome reported for separate screening groups?** | **No policy strategy** | **Risk Strategy** | **Screening / Universal strategy** | **Other strategy** | **If outcome data not reported separately, provide details here** | **Is the outcome reported at the level of the neonate / infant / child or maternal.** | **Other details about time frames** | **Is the outcome reported at short-term, medium-term or longer term?** | **Comments** |
| --- | --- | --- | --- | --- | --- | --- | --- | --- | --- | --- | --- | --- |
| Panneflek 2024 | Eberly 2009 | USA | No |  |  |  |  | Meningitis: With bacteremia or sepsis: 34/860 cases of early onset GBS; Meningitis alone: 16/860 cases of early onset GBS | Neonate <7 days |  | Short-term outcome |  |
| Hasperhoven 2020, Panneflek 2024 | Håkansson 2017 | Sweden | Yes | Early-onset group B streptococcal (GBS) infection cases per time period: verified sepsis/meningitis = 127 | Early-onset group B streptococcal (GBS) infection cases per time period: verified sepsis/meningitis = 100 |  |  |  | Neonate <7 days |  | Short-term outcome | Infants with culture verified sepsis/meningitis: 22 |
| Hasperhoven 2020, Panneflek 2024 | O’Sullivan 2019 | UK and Ireland | Unclear | previous group B streptococcus surveillance in 2000–01 (0·15 per 1000 livebirths) | The incidence of group B streptococcal meningitis in 2014–15 (0·17 per 1000 livebirths) |  |  | "The most common clinical syndrome was septicaemia (449 [62%] of 719 infants), followed by meningitis (155 [22%]), bacteraemic pneumonia (107 [15%]), and focal infections (six [<1%]). A lumbar puncture was done in 600 (84%) of 716 infants; of the remaining 116 (16%), either the procedure was unsuccessful or the baby was deemed too ill for lumbar puncture. Infants with late- onset disease were more likely to present with meningitis than those with early-onset disease (98 [29%] of 339 vs 57 [11%] of 517, respectively). The overall incidence of meningitis was 0·17 per 1000 livebirths. ...The incidence of group B streptococcal meningitis in 2014–15 (0·17 per 1000 livebirths) was similar to that found in our national meningitis surveillance study in 2010–11 (0·16 per 1000 livebirths) 28 and in the previous group B streptococcus surveillance in 2000–01 (0·15 per 1000 livebirths).12 Therefore, the burden of group B streptococcal meningitis in young infants has changed little over the past 15 years. | Other (please specify) |  | Short-term outcome | The incidence of GBS per 1000 live births (95% CI) was: Early onset: 0·57 (0·52–0·62) and late onset: 0·37 (0·33–0·41). The paper refers to supplementary data presented in an Appendix but having accessed it multiple times this is an empty document. Authors have been contacted to obtain the Appendix document. |
| Panneflek 2024 | Uy 2002 | USA | No |  |  |  |  | Meningitis. One infant developed meningitis with a negative blood culture. | Other (please specify) | No timeframe given | Short-term outcome |  |

**Abbreviations**: EOD: early onset disease, EOGBS: Early-Onset Group B Streptococcal Disease, LOD: late onset disease, LOGBS: late onset Group B Streptococcal Disease
Studies may be reported in multiple tables

### File 20.3 Universal screening strategy versus risk based: septicaemia and meningitis

| **Review** | **Authors** | **Country** | **Is the outcome reported for separate screening groups?** | **No policy strategy** | **Risk Strategy** | **Screening / Universal strategy** | **Other strategy** | **If outcome data not reported separately, provide details here** | **Is the outcome reported at the level of the neonate / infant / child or maternal.** | **Other details about time frames** | **Is the outcome reported at short-term, medium-term or longer term?** | **Comments** |
| --- | --- | --- | --- | --- | --- | --- | --- | --- | --- | --- | --- | --- |
| Li 2020, Panneflek 2024 | Abdelmaaboud 2011 | Qatar | No |  | 2006 to 2009 (a risk-based approach) | 2003 to 2006 (universal screening approach) |  | Out of 45 neonates with EOGBS disease, 34 (75.5%) had signs of illness before 24 h of life. Twelve well-appearing term infants were evaluated shortly after birth because of intrapartum risk factors for sepsis according to our hospital’s guidelines. Nine term neonates and one preterm neonate appeared well at birth but were evaluated for infection after developing signs of illness in the first 24 h of life. In six of these cases, the mothers were screened GBS negative and there were no other intrapartum risk factors for sepsis. The other four cases have been evaluated for infection because of intrapartum risk factors for sepsis and their mothers had unknown GBS status. Lumber puncture was done in 38 cases (84.4%) and 4 (8.9%) cases were proved to be meningitis. | Neonate i.e. 1-28 days |  | Short-term outcome | Separate data not reported for screening strategy. Authors report characteristics of neonates comparing term babies with pre-term babies.  In this study universal screening was initially implemented and then they introduced risk-based screening. |
| Panneflek 2024 | Al Luhidan 2019 | Saudi Arabia | No |  |  |  |  | 3 cases of LOD with meningitis. Authors state that "Meningitis was significantly associated with the onset of the disease (P < 0.008) and was observed only in LOD patients" | Other (please specify) | "LOGBS definition: GBS from a sterile body site after 7 days of age but before 90 days of age" | Medium-term outcome | "Meningitis was exclusively seen in LOD, constituting 17.7% of the LOD presentation". Data for each strategy is not reported separately but presented according to whether it is EOD or LOD. |
| Panneflek 2024 | Eberly 2009 | USA | No |  |  |  |  | Meningitis: With bacteremia or sepsis: 34/860 cases of early onset GBS; Meningitis alone: 16/860 cases of early onset GBS | Neonate <7 days |  | Short-term outcome |  |
| Li 2020, Hasperhoven 2020, Panneflek 2024 | Gopal Rao 2017 | UK | Yes |  | Meningitis: 0 cases | Meningitis: 1 case |  |  |  |  |  |  |
| Panneflek 2024 | Ko 2021 | Taiwan | No |  |  |  |  | "The predominant pathogen of EOS in preterm and term infants was E. coli (60%) and GBS (43%), respectively. All patients had bacteremia. CSF was collected in 48 patients, of whom 11 had culture-proven meningitis. Pathogens isolated from the CSF were E. coli (6 cases), GBS (4 cases), and GDS (1 case)" | Unclear / not specified |  | Short-term outcome |  |
| Panneflek 2024 | Lee 2021 | Singapore | No |  |  |  |  | Between 2001 and 2015, nine neonates were diagnosed with GBS sepsis. Reported postnatal co-morbidities included: one case with meningitis and seizures, one with pneumonia and persistent pulmonary hypertension of the newborn, and one with pneumonia alone. The remaining six cases had no additional postnatal co-morbidities. | Other (please specify) | Neonate - no age given | Short-term outcome |  |

**Abbreviations**: EOD: early onset disease, EOGBS: Early-Onset Group B Streptococcal Disease, LOD: late onset disease, LOGBS: late onset Group B Streptococcal Disease
Studies may be reported in multiple tables

### File 20.4 Universal screening versus universal screening (timing of screening): septicaemia and meningitis

| **Review** | **Authors** | **Country** | **Is the outcome reported for separate screening groups?** | **No policy strategy** | **Risk Strategy** | **Screening / Universal strategy** | **Other strategy** | **If outcome data not reported separately, provide details here** | **Is the outcome reported at the level of the neonate / infant / child or maternal.** | **Other details about time frames** | **Is the outcome reported at short-term, medium-term or longer term?** | **Comments** |
| --- | --- | --- | --- | --- | --- | --- | --- | --- | --- | --- | --- | --- |
| Panneflek 2024 | El Helali 2019 | France | Yes |  |  | Timing of GBS determination i.e., Antenatal vs Intrapartum |  | "The number of proven (bacteremia and meningitis) and probable early-onset GBS disease cases was 60% lower in the intrapartum PCR period, 3.8/1,000 (95% CI 2.3–7.4) compared with 0.9/1,000 (95% CI 0.6–1.5) (P<001). Further data reported in Table 2 stratified by positive screening, negative screening and unknown screening | Neonate <7 days |  | Short-term outcome | Two time periods are compared: (a) 2006-2009: 4-Year Antenatal Culture Screening Strategy with (b) 2010–2015: 6-Year Intrapartum PCR Screening Strategy |

**Abbreviations**: EOD: early onset disease, EOGBS: Early-Onset Group B Streptococcal Disease, LOD: late onset disease, LOGBS: late onset Group B Streptococcal Disease
Studies may be reported in multiple tables

### File 20.5 Universal screening versus other strategy: septicaemia and meningitis

| **Review** | **Authors** | **Country** | **Is the outcome reported for separate screening groups?** | **No policy strategy** | **Risk Strategy** | **Screening / Universal strategy** | **Other strategy** | **If outcome data not reported separately, provide details here** | **Is the outcome reported at the level of the neonate / infant / child or maternal.** | **Other details about time frames** | **Is the outcome reported at short-term, medium-term or longer term?** | **Comments** |
| --- | --- | --- | --- | --- | --- | --- | --- | --- | --- | --- | --- | --- |
| Panneflek 2024 | Lukacs 2012 | USA | Yes | Bacterial meningitis: 0.6% (n=1116047) |  | Bacterial meningitis: 0.9% (n=608569) | Bacterial meningitis: 0.9% (n=796633) | Bacterial meningitis based on sepsis hospitalisation in infants aged < 3 months | Other (please specify) | Characteristics of sepsis hospitalisation in infants <3 months | Other | Among the 12% of sepsis hospitalizations that involved a pathogen-specific ICD-9-CM code (Table I and Appendix), 38% were due to Streptococcus spp (9% identified as GBS)...There were no significant differences in the percentage of hospitalizations with meningitis or respiratory failure, the percentage of fatal cases, or geographic distribution.  LOGBS definition: late-onset sepsis: up to 90 days of age and after first week of life. Authors note that "we could not determine the date of onset of sepsis to distinguish early-onset sepsis from late-onset sepsis, if more than one episode of sepsis occurred during a single newborn hospital course or if an infant was hospitalized more than once" |
| Hasperhoven 2020, Panneflek 2024 | Phares 2008 | USA | No |  |  |  |  | When compared with early onset cases, a higher proportion of late-onset cases manifested as meningitis (27% vs 7% chi-squared test, P<0.001) | Other (please specify) | late onset (7–90 days) | Short-term outcome | LOGBS definition: 7 - 89 days old |
| Hasperhoven 2020, Panneflek 2024 | Phares 2008 | USA | No |  |  |  |  | "The most commonly identified syndromes were bacteremia without focus (83%), pneumonia (9%), and meningitis (7%). Overall, among 1224 infants for whom outcome was known, 83 (6.8%) died. The proportion who died varied by year (range, 5%-9%), although no trend over time was observed. It also varied by syndrome, from 9% (10/114) for pneumonia to 4% (3/ 81) for meningitis" | Neonate <7 days |  | Short-term outcome |  |

**Abbreviations**: EOD: early onset disease, EOGBS: Early-Onset Group B Streptococcal Disease, LOD: late onset disease, LOGBS: late onset Group B Streptococcal Disease
Studies may be reported in multiple tables

### File 20.6 Risk based verses other strategy: septicaemia and meningitis

| **Review** | **Authors** | **Country** | **Is the outcome reported for separate screening groups?** | **No policy strategy** | **Risk Strategy** | **Screening / Universal strategy** | **Other strategy** | **If outcome data not reported separately, provide details here** | **Is the outcome reported at the level of the neonate / infant / child or maternal.** | **Other details about time frames** | **Is the outcome reported at short-term, medium-term or longer term?** | **Comments** |
| --- | --- | --- | --- | --- | --- | --- | --- | --- | --- | --- | --- | --- |
| Panneflek 2024 | Uy 2002 | USA | No |  |  |  |  | Meningitis. One infant developed meningitis with a negative blood culture. | Other (please specify) | No timeframe given | Short-term outcome |  |

**Abbreviations**: EOD: early onset disease, EOGBS: Early-Onset Group B Streptococcal Disease, LOD: late onset disease, LOGBS: late onset Group B Streptococcal Disease
Studies may be reported in multiple tables

### File 20.7: No strategy versus other strategy: septicaemia and meningitis

| **Review** | **Authors** | **Country** | **Is the outcome reported for separate screening groups?** | **No policy strategy** | **Risk Strategy** | **Screening / Universal strategy** | **Other strategy** | **If outcome data not reported separately, provide details here** | **Is the outcome reported at the level of the neonate / infant / child or maternal.** | **Other details about time frames** | **Is the outcome reported at short-term, medium-term or longer term?** | **Comments** |
| --- | --- | --- | --- | --- | --- | --- | --- | --- | --- | --- | --- | --- |
| Panneflek 2024 | Alarcon 2004 | Spain | Yes | Neonates with early onset E Coli: Meningitis: 2/16 |  |  | Neonates with early onset E Coli: Meningitis (data combined from period 2 and 3): 4/25 | Neonates with early onset E Coli: Meningitis: Period 1: 2/16; Period 2: 0/5; Period 3: 4/20 | Neonate <7 days |  | Short-term outcome | Note: this is the clinical characteristics of 41 infants with early onset *Escherichia coli* infections |
| Hasperhoven 2020, Panneflek 2024 | Darlow 2016 | New Zealand | No |  |  |  |  | Meningitis. "29 cases of confirmed early-onset GBS sepsis, including one case of meningitis" ((Two further cases of GBS sepsis presented after the first 48 h, at 62 and 96 h of age, the latter with confirmed meningitis, and both survived.) | Neonate <7 days |  | Short-term outcome | "Our retrospective laboratory survey identified two further cases, and we believe we will have missed few others because we have focussed upon early-onset disease, although we acknowledge that our survey method does not guarantee all cases will be detected."  Authors report "Data on causes of early-onset neonatal sepsis other than GBS and on sepsis presenting from days 3 to 6 of life will be reported elsewhere." No reference is provided. |
| Panneflek 2024 | Isaacs 1999 | Australia | Unclear |  |  |  |  | Total number of meningitis cases:21/ total number of EOGBS sepsis cases: 219 | Neonate <7 days |  | Short-term outcome | According to Isaacs 1999, data presented from three time periods: 1991-1993, 1993-1995 and 1995-1997. Table 5 in that publication shows that only 3 out of 9 hospitals had a a formal policy on IAP use. One hospital policy was to screen women for GBS at 28 weeks of gestation, and give IAP only for women with risk factors in labour; The second hospital policy recommended screening for GBS at 28 weeks and giving IAP to all GBS carriers and the third hospital policy stated no screening and giving IAP to any women with risk factors. Six hospitals in 1991 did not have a formal IAP policy. By 1997 all hospitals had a policy, "4 were based on maternal screening and 7 were based on treatment of women with risk factors for neonatal sepsis without screening". Due to the variation in screening approaches across time and hospital, we have extracted the data for total number of cases. |
| Panneflek 2024 | Lukacs 2012 | USA | Yes | Bacterial meningitis: 0.6% (n=1116047) |  | Bacterial meningitis: 0.9% (n=608569) | Bacterial meningitis: 0.9% (n=796633) | Bacterial meningitis based on sepsis hospitalisation in infants aged < 3 months | Other (please specify) | Characteristics of sepsis hospitalisation in infants <3 months | Other | Among the 12% of sepsis hospitalizations that involved a pathogen-specific ICD-9-CM code (Table I and Appendix), 38% were due to Streptococcus spp (9% identified as GBS). There were no significant differences in the percentage of hospitalizations with meningitis or respiratory failure, the percentage of fatal cases, or geographic distribution.  LOGBS definition: late-onset sepsis: up to 90 days of age and after first week of life. Authors note that "we could not determine the date of onset of sepsis to distinguish early-onset sepsis from late-onset sepsis, if more than one episode of sepsis occurred during a single newborn hospital course or if an infant was hospitalized more than once" |
| Panneflek 2024 | Poulain 1997 | France | No |  |  |  |  | "We noticed four cases of neonatal bacteraemia of GBS. One case arose from the group of carriers (but no condition of risk of fetal contamination during the labor and no chemoprophylaxy). The three other cases were from women with a negative antepartum screening. There was no case of meningitis and all four babies were in good health at day 10 of life." | Neonate i.e. 1-28 days |  | Short-term outcome |  |
| Panneflek 2024 | Trijbels-Smeulders 2006 | The Netherlands | No |  |  |  |  | "In this study, we typed GBS-strains isolated in the Netherlands in 1997–1999 from patients with neonatal sepsis and meningitis and we studied the relationship between the clinical presentation of the disease and the serotype and/or genotype of GBS strains isolated before (1997–1998) and after introduction of antibiotic prophylaxis (1999)...The 198 neonates with GBS sepsis and/or meningitis included 105 (53%) males….There was no difference in the serotype distribution between the 2 periods (1997–1998 and 1999)." | Unclear / not specified |  | Short-term outcome | There will be overlap reported between Trijbels-Smeulders et al 2006 and Trijbels-Smeulders et al. 2007 paper. Data has been extracted separately for these two publications at present. |
| Panneflek 2024 | Trijbels-Smeulders 2006 | The Netherlands | No |  |  |  |  | Number of cases with early-onset (< /=7 d) meningitis: 5/142 | Neonate <7 days |  | Short-term outcome |  |
| Panneflek 2024 | Trijbels-Smeulders 2006 | The Netherlands | No |  |  |  |  | Number of cases with early-onset (< /=7 d) sepsis + meningitis: 24/142 | Neonate <7 days |  | Short-term outcome |  |
| Panneflek 2024 | Trijbels-Smeulders 2006 | The Netherlands | No |  |  |  |  | Number of cases with late-onset (>7 d) meningitis: 10/56 | Unclear / not specified |  | Unclear/Not reported | In this paper, authors refer to late-onset as > 7days; no further details given. |
| Panneflek 2024 | Trijbels-Smeulders 2006 | The Netherlands | No |  |  |  |  | Number of cases with late-onset (>7 d) sepsis + meningitis: 24/56 | Unclear / not specified |  | Unclear/Not reported | In this paper, authors refer to late-onset as > 7days; no further details given. |
| Panneflek 2024 | Trijbels-Smeulders 2007 | The Netherlands | Yes | Corrected incidence of proven GBS meningitis: 0.14 (95% CI 0.11 to 0.17) per 1000 live births |  |  | Corrected incidence of proven GBS meningitis: 0.17 (95% CI 0.15 to 0.19) per 1000 live births | Incidence of GBS meningitis: "In 1997–8 and 1999–2001, 29 and 57 patients, respectively, with proven GBS meningitis were reported to the reference microbiology laboratory. As 16 and 36 of these were also reported to the DPSU, the DPSU captured only 55% of GBS meningitis cases in 1997–8 and 63% in 1999–2001. Therefore, the corrected incidence of proven GBS meningitis in these two periods was 0.14 (95% CI 0.11 to 0.17) per 1000 live births and 0.17 (95% CI 0.15 to 0.19) per 1000 live births, respectively, and in the total five-year period it was 0.16 (95% CI 0.14 to 0.17) per 1000 live births. The proportion of patients with GBS meningitis was clearly related to the time of onset of GBS disease and increased from 6% if the onset was ,12 h to 53% if the onset was >7 days after birth". Note data shown in relevant Table 2 was not extracted as it presented data from across the total 5 year period. | Other (please specify) | Definitions of timeframes: very early onset (<12 h); late early onset (12 h – <7 days) and late onset (7–90 days). | Other | Note: data shown in relevant Table 2 was not extracted as it presented data from across the total 5 year period and did not report separate data for screening strategies. |
| Panneflek 2024 | Uy 2002 | USA | No |  |  |  |  | Meningitis. One infant developed meningitis with a negative blood culture. | Other (please specify) | No timeframe given | Short-term outcome |  |

**Abbreviations**: EOD: early onset disease, EOGBS: Early-Onset Group B Streptococcal Disease, LOD: late onset disease, LOGBS: late onset Group B Streptococcal Disease
Studies may be reported in multiple tables
