# Supplementary material for: Benefits and Harms of Antenatal/Intrapartum Screening for Maternal Group B Streptococcus and Use of Intrapartum Antibiotic Prophylaxis Versus Risk‐Based Protocols or No Intervention: A Rapid Review
Source: Acta Paediatr. 2026 Apr 30;115(8):1598–610. doi: 10.1111/apa.70568 (PMC13371836; doi:10.1111/apa.70568)
Supplement: Supplementary file 21 — Data S21: Pneumonia: Neonatal health outcomes. [file APA-115-1598-s008.docx]

## Supplementary materials File 21. Pneumonia: neonatal health outcomes

Tables describe pneumonia as reported by primary study and presented by strategy. Studies may appear in more than one table if multiple strategies were used.

File 21.1 Universal screening versus no strategy: pneumonia

| **Review** | **Authors** | **Country** | **Is the outcome reported for separate screening groups?** | **No policy strategy** | **Risk Strategy** | **Screening / Universal strategy** | **Other strategy** | **If outcome data not reported separately, provide details here** | **Is the outcome reported at the level of the neonate / infant / child or maternal.** | **Other details about time frames** | **Is the outcome reported at short-term, medium-term or longer term?** | **Comments** |
| --- | --- | --- | --- | --- | --- | --- | --- | --- | --- | --- | --- | --- |
| Panneflek 2024 | Eberly 2009 | USA | No |  |  |  |  | Pneumonia (without sepsis): 232/860 cases of early onset GBS | Neonate <7 days |  | Short-term outcome |  |
| Hasperhoven 2020, Li 2020, Panneflek 2024 | Main 2000 | USA | Yes | Number of Group B streptococcal early-onset pneumonia cases/total number of births: 3/6829 | Group B streptococcal early-onset pneumonia: 7/13270 | Group B streptococcal early-onset pneumonia: 0/9302 |  |  | Neonate <7 days |  | Short-term outcome | Early-onset pneumonia was defined as clinical pneumonia with a positive aspirate culture result within the first 7 days after birth |
| Panneflek 2024 | Matsubara 2013 | Japan | No |  |  |  |  | 9/88 cases of EOD with pneumonia | Neonate <7 days |  |  |  |
| Panneflek 2024 | Matsubara 2013 | Japan | No |  |  |  |  | 3/162 cases of LOD with pneumonia | Other (please specify) | Late-onset disease |  |  |

**Abbreviations**: EOD: early onset disease, EOGBS: Early-Onset Group B Streptococcal Disease, LOD: late onset disease
Studies may be reported in multiple tables when three strategies were reported

File 21.2 Risk based versus no strategy: pneumonia

| **Review** | **Authors** | **Country** | **Is the outcome reported for separate screening groups?** | **No policy strategy** | **Risk Strategy** | **Screening / Universal strategy** | **Other strategy** | **If outcome data not reported separately, provide details here** | **Is the outcome reported at the level of the neonate / infant / child or maternal.** | **Other details about time frames** | **Is the outcome reported at short-term, medium-term or longer term?** | **Comments** |
| --- | --- | --- | --- | --- | --- | --- | --- | --- | --- | --- | --- | --- |
| Panneflek 2024 | Eberly 2009 | USA | No |  |  |  |  | Pneumonia (without sepsis): 232/860 cases of early onset GBS | Neonate <7 days |  | Short-term outcome |  |
| Hasperhoven 2020, Panneflek 2024 | Håkansson 2017 | Sweden | Yes | Early-onset GBS infection cases per time period: clinical sepsis/pneumonia: 104 | Early-onset GBS infection cases per time period: clinical sepsis/pneumonia: 64 |  |  |  | Neonate <7 days |  | Short-term outcome | Infants with clinical sepsis/pneumonia: 168 "A significant decrease in the number of cases with clinical GBS sepsis/pneumonia was also observed" |
| Hasperhoven 2020, Li 2020, Panneflek 2024 | Main 2000 | USA | Yes | Number of Group B streptococcal early-onset pneumonia cases/total number of births: 3/6829 | Group B streptococcal early-onset pneumonia: 7/13270 | Group B streptococcal early-onset pneumonia: 0/9302 |  |  | Neonate <7 days |  | Short-term outcome | Early-onset pneumonia was defined as clinical pneumonia with a positive aspirate culture result within the first 7 days after birth |

**Abbreviations**: EOD: early onset disease, EOGBS: Early-Onset Group B Streptococcal Disease, LOD: late onset disease
Studies may be reported in multiple tables

File 21.3 Universal screening versus risk based: pneumonia

| **Review** | **Authors** | **Country** | **Is the outcome reported for separate screening groups?** | **No policy strategy** | **Risk Strategy** | **Screening / Universal strategy** | **Other strategy** | **If outcome data not reported separately, provide details here** | **Is the outcome reported at the level of the neonate / infant / child or maternal.** | **Other details about time frames** | **Is the outcome reported at short-term, medium-term or longer term?** | **Comments** |
| --- | --- | --- | --- | --- | --- | --- | --- | --- | --- | --- | --- | --- |
| Panneflek 2024 | Al Luhidan 2019 | Saudi Arabia | No |  |  |  |  | 1 case with pneumonia in EOD | Neonate <7 days |  | Short-term outcome | "Pneumonia was defined as GBS-associated pneumonia." |
| Panneflek 2024 | Eberly 2009 | USA | No |  |  |  |  | Pneumonia (without sepsis): 232/860 cases of early onset GBS | Neonate <7 days |  | Short-term outcome |  |
| Panneflek 2024 | Lee 2021 | Singapore | No |  |  |  |  | Between 2001 and 2015, nine neonates were diagnosed with GBS sepsis. Reported postnatal co-morbidities included: one case with meningitis and seizures, one with pneumonia and persistent pulmonary hypertension of the newborn, and one with pneumonia alone. The remaining six cases had no additional postnatal comorbidities. | Other (please specify) | Neonate - no age given | Short-term outcome |  |
| Hasperhoven 2020, Li 2020, Panneflek 2024 | Main 2000 | USA | Yes | Number of GBS early-onset pneumonia cases/total number of births: 3/6829 | Group B streptococcal early-onset pneumonia: 7/13270 | Group B streptococcal early-onset pneumonia: 0/9302 |  |  | Neonate <7 days |  | Short-term outcome | Early-onset pneumonia was defined as clinical pneumonia with a positive aspirate culture result within the first 7 days after birth |

**Abbreviations**: EOD: early onset disease, EOGBS: Early-Onset Group B Streptococcal Disease, LOD: late onset disease
Studies may be reported in multiple tables

File 21.4 Universal screening versus other strategy: pneumonia

| **Review** | **Authors** | **Country** | **Is the outcome reported for separate screening groups?** | **No policy strategy** | **Risk Strategy** | **Screening / Universal strategy** | **Other strategy** | **If outcome data not reported separately, provide details here** | **Is the outcome reported at the level of the neonate / infant / child or maternal.** | **Other details about time frames** | **Is the outcome reported at short-term, medium-term or longer term?** | **Comments** |
| --- | --- | --- | --- | --- | --- | --- | --- | --- | --- | --- | --- | --- |
| Hasperhoven 2020, Panneflek 2024 | Phares 2008 | USA | No |  |  |  |  | Other disease manifestations included bacteremia without focus (65%), bacteremic cellulitis (3%), and pneumonia (3%). | Other (please specify) | late onset (7–90 days) | Short-term outcome | LOGBS definition: 7 - 89 days old |
| Hasperhoven 2020, Panneflek 2024 | Phares 2008 | USA | No |  |  |  |  | "The most commonly identified syndromes were bacteremia without focus (83%), pneumonia (9%), and meningitis (7%). Overall, among 1224 infants for whom outcome was known, 83 (6.8%) died. The proportion who died varied by year (range, 5%-9%), although no trend over time was observed. It also varied by syndrome, from 9% (10/114) for pneumonia to 4% (3/ 81) for meningitis" | Neonate <7 days |  | Short-term outcome |  |

**Abbreviations**: EOD: early onset disease, EOGBS: Early-Onset Group B Streptococcal Disease, LOD: late onset disease, LOGBS: late onset Group B Streptococcal Disease
Studies may be reported in multiple tables

File 21.5 Other strategy versus no strategy: pneumonia

| **Review** | **Authors** | **Country** | **Is the outcome reported for separate screening groups?** | **No policy strategy** | **Risk Strategy** | **Screening / Universal strategy** | **Other strategy** | **If outcome data not reported separately, provide details here** | **Is the outcome reported at the level of the neonate / infant / child or maternal.** | **Other details about time frames** | **Is the outcome reported at short-term, medium-term or longer term?** | **Comments** |
| --- | --- | --- | --- | --- | --- | --- | --- | --- | --- | --- | --- | --- |
| Panneflek 2024 | Horváth 2013 | Hungary | Yes | 88 neonates |  |  | 19 neonates |  | Neonate <7 days |  | Short-term outcome | P = 0.001; OR, 0.19; 95% CI, 0.11–0.32. P values were calculated by the χ2 test |

**Abbreviations**: CI: confidence intervals, EOD: early onset disease, EOGBS: Early-Onset Group B Streptococcal Disease, LOD: late onset disease, OR: odds ratio
Studies may be reported in multiple tables
