# Supplementary material for: Benefits and Harms of Antenatal/Intrapartum Screening for Maternal Group B Streptococcus and Use of Intrapartum Antibiotic Prophylaxis Versus Risk‐Based Protocols or No Intervention: A Rapid Review
Source: Acta Paediatr. 2026 Apr 30;115(8):1598–610. doi: 10.1111/apa.70568 (PMC13371836; doi:10.1111/apa.70568)
Supplement: Supplementary file 22 — Data S22: Other: Neonatal health outcomes. [file APA-115-1598-s018.docx]

## Supplementary materials file 22 (S22): Other neonatal health outcomes reported by strategy

Tables describe other neonatal health outcomes as reported by primary study and presented by strategy. These include urinary tract infection. Studies may appear in more than one table if multiple strategies were used.

### File 22.1 Universal screening versus no strategy: other neonatal health outcomes

| **Review** | **Authors** | **Country** | **Is the outcome reported for separate screening groups?** | **No policy strategy** | **Risk Strategy** | **Screening / Universal strategy** | **Other strategy** | **If outcome data not reported separately, provide details here** | **Is the outcome reported at the level of the neonate / infant / child or maternal.** | **Other details about time frames** | **Is the outcome reported at short-term, medium-term or longer term?** | **Comments** |
| --- | --- | --- | --- | --- | --- | --- | --- | --- | --- | --- | --- | --- |
| Panneflek 2024 (1) | Alarcon et al. 2004 (2) | Spain | No | 1992-1995: not clear what the policy is but it predates the CDC 1996 guidelines so likely this is no policy |  |  | Authors refer to implementation of CDC 1996 in period 2 and the refer directly to CDC 1996 in period 3 | 27/41 cases had early onset E Coli infection. Of these 40/41 had "known duration of rupture of membranes and 28/41 had information about IAP use available for review" |  |  |  | Only report data for Early-onset E Coli sepsis and antimicrobial resistance. No EOGBS data reported |
| Panneflek 2024 (1) | Bauserman 2013 (3) | USA | Yes | 4.2 (0.9-11.9) |  | 7.4 (5.3 - 10.0) |  | Percentage of LOGBS case-fatality (95%-CI) | Other (please specify) | LOGBS - from positive culture from 4 - 120 postnatal days | Other |  |
| Panneflek 2024 (1) | Cho 2019 (4) | Taiwan | No |  |  |  |  | CNS involvement/neurologic sequelae reported for EOD: 1 case, n=31 and LOD: 5 cases, n=12 | Other (please specify) | Neonate - data compared EOD and LOD |  | "Six babies had central nervous system involvement including meningitis, intraventricular or intracranial hemorrhage, and seizures. Two of these babies suffered from neurologic sequelae including epileptic events, speech delay, and brain infarction that required rehabilitation therapy." |
| Panneflek 2024 (1) | Cho 2019 (4) | Taiwan | No |  |  |  |  | EOD group oxygen support (n=31): Nasal cannula/ nasal prong continuous positive airway pressure: 12 cases, Intubation: 3 cases. LOD group oxygen support (n=12): Nasal cannula/ nasal prong continuous positive airway pressure: 0 cases, Intubation: 1 case. | Other (please specify) | Neonate - data compared EOD and LOD |  | "The mean hospital stay of the sick babies was 8.7 days (ranged 3-31 days). Sixteen (37.2%) cases were admitted to the NICU for intensive monitoring and treatment (ranged 3-13 days). Most of these cases were in EOD group, and they had a longer intensive ward stay compared to those in LOD group. Sixteen infants required ventilator support during the hospital stay. The majority of these infants required only nasal cannula or nasal prong assistance, and only four babies were intubated (three in the EOD group and one in the LOD group). The requirement of oxygen assistance was significantly greater in the EOD group." |
| Panneflek 2024 (1) | Eberly 2009 (5) | USA | No |  |  |  |  | Isolated urinary tract infection: 22/860 cases of early onset GBS | Neonate <7 days |  | Short-term outcome |  |
| Panneflek 2024 (1) | Eberly 2009 (5) | USA | No |  |  |  |  | Cellulitis/abscess: 4/860 cases of early onset GBS | Neonate <7 days |  | Short-term outcome |  |
| Panneflek 2024 (1) | Eberly 2009 (5) | USA | No |  |  |  |  | Osteomyelitis: 1/860 cases of early onset GBS | Neonate <7 days |  | Short-term outcome |  |
| Panneflek 2024 (1) | Gibbs1994 (6) | USA | No |  |  |  |  | "There were no adverse effects of prophylatic antibiotic administration on the mothers or infants" | Other (please specify) | Mother and neonate | Short-term outcome |  |
| Panneflek 2024 (1) | Jeffery 1998 (7) | Australia | Yes | 12 neonates ventilated / total number of live births: 5732 |  | 13 neonates ventilated / total number of live births: 36342 |  | "There was a statistically significant reduction in neonatal morbidity outcomes after intervention compared with the preintervention group, including requirement for admission and treatment in a neonatal unit and the need for ventilation" | Neonate <7 days |  | Short-term outcome | Data extracted from Panneflek 2024. According to Jeffery 1998, the "background rate of EOGBSD was determined prospectively over a 16-month period (November 1986 to February 1988) before the intervention, which was introduced as protocol in March 1988. After 3 months of in-service education for the staff, data were collected prospectively on all infants with EOGBSD from June 1988" |
| Panneflek 2024 (1) | Katz 1994 (8) | USA | Yes | 4/1977 had GBS. Of these, one was premature and died; one had no complications and was discharged at day 7, one case had a 'complicated course in the NICU' and was discharged without long-term sequalae, and one case had multisystem organ failure with severe neurological sequelae including seizures, hydrocephalus which required shunting and subsequent developmental delay. |  | 0 |  | According to Katz 1994, "During the 2 years of evaluation there were no neonatal deaths in the study population among infants of GBS carriers related to infection from other organisms. There was also no superinfection or morbidity from other organisms in this group of mothers or their infants. In the comparison group, 4/1977 had GBS. Of these, one was premature and died; one had no complications and was discharged at day 7, one case had a 'complicated course in the NICU' and was discharged without long-term sequalae, and one case had multisystem organ failure with severe neurological sequelae including seizures, hydrocephalus which required shunting and subsequent developmental delay. | Neonate <7 days |  | Short-term outcome |  |
| Panneflek 2024 (1) | Lukacs 2012 (9) | USA | Yes | CFR: 2.3% (n=1116047) |  | CFR: 3.2% (n=608569) | CFR: 3.1% (n=796633) | Case fatality rate based on sepsis hospitalisation in infants aged < 3 months | Other (please specify) | Characteristics of sepsis hospitalisation in infants <3 months | Other | Among the 12% of sepsis hospitalizations that involved a pathogen-specific ICD-9-CM code (Table I and Appendix), 38% were due to Streptococcus spp (9% identified as GBS). There were no significant differences in the percentage of hospitalizations with meningitis or respiratory failure, the percentage of fatal cases, or geographic distribution.  LOGBS definition: late-onset sepsis: up to 90 days of age and after first week of life. Authors note that "we could not determine the date of onset of sepsis to distinguish early-onset sepsis from late-onset sepsis, if more than one episode of sepsis occurred during a single newborn hospital course or if an infant was hospitalized more than once" |
| Panneflek 2024 (1) | Lukacs 2012 (9) | USA | Yes | Respiratory failure: 0.8% (n=1116047) |  | Respiratory failure: 1.2% (n=608569) | Respiratory failure: 1.2% (n=796633) | Respiratory failure based on sepsis hospitalisation in infants aged < 3 months | Other (please specify) | Characteristics of sepsis hospitalisation in infants <3 months | Other | Among the 12% of sepsis hospitalizations that involved a pathogen-specific ICD-9-CM code (Table I and Appendix), 38% were due to Streptococcus spp (9% identified as GBS). There were no significant differences in the percentage of hospitalizations with meningitis or respiratory failure, the percentage of fatal cases, or geographic distribution.  LOGBS definition: late-onset sepsis: up to 90 days of age and after first week of life. Authors note that "we could not determine the date of onset of sepsis to distinguish early-onset sepsis from late-onset sepsis, if more than one episode of sepsis occurred during a single newborn hospital course or if an infant was hospitalized more than once" |
| Panneflek 2024 (1) | Matsubara 2013 (10) | Japan | No |  |  |  |  | Survived with sequelae: 12/88 (EOD) | Neonate <7 days | EOD |  | A high rate of neurological sequelae was noted in meningitis in EOD (33.3%, 8/24) and LOD (34.1%, 29/85; p < 0.001 vs. non-meningitis LOD (6.6%, 5/76)) cases. Speech or mental delay (n = 23), epilepsy (n = 13), cerebral palsy (n = 9), brain atrophy (n = 7), hydrocephalus (n = 4), visual impairment (n = 3), and deafness (n = 2) were documented. |
| Panneflek 2024 (1) | Matsubara 2013 (10) | Japan | No |  |  |  |  | Survived with sequelae: 34/162 (LOD) | Other (please specify) | LOD |  | A high rate of neurological sequelae was noted in meningitis in EOD (33.3%, 8/24) and LOD (34.1%, 29/85; p < 0.001 vs. non-meningitis LOD (6.6%, 5/76)) cases. Speech or mental delay (n = 23), epilepsy (n = 13), cerebral palsy (n = 9), brain atrophy (n = 7), hydrocephalus (n = 4), visual impairment (n = 3), and deafness (n = 2) were documented. |
| Panneflek 2024 (1) | Matsubara 2013 (10) | Japan | No |  |  |  |  | Arthritis: 4/162 (LOD) [no neonates with EOD were diagnosed with arthritis] | Other (please specify) | LOD |  | "Cellulitis and arthritis had an excellent prognosis without any fatality or complications." |
| Panneflek 2024 (1) | Matsubara 2013 (10) | Japan | No |  |  |  |  | Cellulitis: 5/162 (LOD) [no neonates with EOD were diagnosed with arthritis] | Other (please specify) | LOD |  | "Cellulitis and arthritis had an excellent prognosis without any fatality or complications." |
| Panneflek 2024 (1) | Sutkin 2005 (11) | USA | Yes |  | 0.61 |  | 0.14 | Early onset prevalence (per 1000 live births for GBS): RR 0.23 (0.08–0.67) | Neonate <7 days |  | Short-term outcome | Authors state "Our institution has experienced a substantial (87%) decrease in the rate of GBS early-onset neonatal sepsis since the initiation of our guidelines - cite a reference to Brozanski BS, Jones JG, Krohn MA, Sweet RL. Effect of a screening-based prevention policy on prevalence of early- onset group B streptococcal sepsis. Obstet Gynecol 2000; 95:496–501. This study is also included in our review. |

**Abbreviations**: CFR: case fatality rate, CNS: central nervous system; EOD: early onset disease, EOGBS: Early-Onset Group B Streptococcal Disease, LOD: late onset disease, LOGBS: late onset Group B Streptococcal Disease
Studies may be reported in multiple tables

### File 22.2 Risk based versus no strategy: other neonatal health outcomes

| **Review** | **Authors** | **Country** | **Is the outcome reported for separate screening groups?** | **No policy strategy** | **Risk Strategy** | **Screening / Universal strategy** | **Other strategy** | **If outcome data not reported separately, provide details here** | **Is the outcome reported at the level of the neonate / infant / child or maternal.** | **Other details about time frames** | **Is the outcome reported at short-term, medium-term or longer term?** | **Comments** |
| --- | --- | --- | --- | --- | --- | --- | --- | --- | --- | --- | --- | --- |
| Panneflek 2024 (1) | Alarcon 2004 (2) | Spain | No | 1992-1995: not clear what the policy is but it predates the CDC 1996 guidelines so likely this is no policy |  |  | Authors refer to implementation of CDC 1996 in period 2 and the refer directly to CDC 1996 in period 3 | 27/41 cases had early onset E Coli infection. Of these 40/41 had "known duration of rupture of membranes and 28/41 had information about IAP use available for review" |  |  |  | Three time periods. Only report data for Early-onset E Coli sepsis and antimicrobial resistance. No EOGBS data reported |
| Panneflek 2024 (1) | Björnsdóttir 2019 (12) | Iceland | Yes | 0.6 (no data) | 0.4 (no data) |  |  | "Both EOD and LOD increased during the early years, but while EOD subsequently decreased from 0.7/1000 live births in 1991–1995 to 0.2/1000 in 2011–2015, LOD showed a nonsignificant decrease from its peak value of 0.6/1000 in 2001–2005 to 0.4/1000 in 2006–2015 " | Other (please specify) | Late-onset disease (LOD; 7 days to 3 months). | Other | Authors concluded: "The risk-based chemoprophylaxis adopted in Iceland possibly contributed to the decline of EOD but has had limited effect on LOD."  LOGBS definition: 7-90 days; Ultra-LOD (ULOD) >3 months of age. |
| Panneflek 2024 (1) | Eberly 2009 (5) | USA | No |  |  |  |  | Isolated urinary tract infection: 22/860 cases of early onset GBS | Neonate <7 days |  | Short-term outcome |  |
| Panneflek 2024 (1) | Eberly 2009 (5) | USA | No |  |  |  |  | Cellulitis/abscess: 4/860 cases of early onset GBS | Neonate <7 days |  | Short-term outcome |  |
| Panneflek 2024 (1) | Eberly 2009 (5) | USA | No |  |  |  |  | Osteomyelitis: 1/860 cases of early onset GBS | Neonate <7 days |  | Short-term outcome |  |
| Hasperhoven 2020 (13), Panneflek 2024 (1) | O’Sullivan 2019 (14) | UK and Ireland | Unclear |  |  |  |  | "More than half the cases (517 [60%]) presented at 0–6 days (early-onset disease), and 339 (40%) presented at 7–89 days (late-onset disease). 193 (57%) cases of late- onset disease presented at 7–28 days and 130 (38%) presented at 29–89 days (the specific day was not reported for 16 cases of late-onset disease; appendix). The median age at presentation for infants with late-onset disease was 23 days (IQR 15–38); infants born at term presented at a median age of 20 days (14–30) and those born prematurely (<37 weeks’ gestation; 107 [39%] of 323) presented at a median of 35 days (21–55)" | Other (please specify) |  | Short-term outcome | The incidence of GBS per 1000 live births (95% CI) was: Early onset: 0·57 (0·52–0·62) and late-onset: 0·37 (0·33–0·41). The paper refers to supplementary data presented in an Appendix but having accessed it multiple times this is an empty document. Authors have been contacted to obtain the Appendix document. |
| Panneflek 2024 (1) | Trollfors 2022 (15) | Sweden | No |  |  |  |  | "Only two neonates had persistent sequelae,involving cerebral paresis and epilepsy" | Other (please specify) | Some long-term neurological sequelae reported. | Medium-term outcome | Data not reported separately for each screening strategy |
| Panneflek 2024 (1) | Trollfors 2022 (15) | Sweden | No |  |  |  |  | "Of the 196 neonates, 156 were very early onset (< 2days after birth), 10 were early onset (3–6 days after birth) and 30 were late onset (7–27 days after birth)" | Other (please specify) | See outcome description |  | Data not reported separately for each screening strategy |
| Panneflek 2024 (1) | Trollfors 2022 (15) | Sweden | No |  |  |  |  | LOS: The median time spent in hospital for all neonates was 10 days (range 0–128). | Other (please specify) |  | Other | Data not reported separately for each screening strategy |
| Panneflek 2024 (1) | Trollfors 2022 (15) | Sweden | No |  |  |  |  | "There were 60 GBS infections in children aged 28 days to 17 years (median age 1.3 years). The incidence was 1.31 cases/100000 population/year, with no changes over the time period. One child, who died via sudden infant death, had GBS isolated from blood postmortem. Twenty-eight cases were very late-onset infections.All of them had complicated births and neonatal periods due to low gestational age (< 30 weeks) involving asphyxia during birth or congenital malformations. They had not left the hospital when they were infected. All infections were considered as hospital acquired. | Other (please specify) | Children aged 28 days to 17 years (median age 1.3 years) | Other | Data not reported separately for each screening strategy. Additional information: "The median time spent in hospital was 8 days (range 0–150). Thirteen children died, in most cases due to the infection itself without known contributing factors. Sixteen children needed assisted ventilation in the intensive care unit. Four children were later diagnosed with intellectual disability, one had a subarachnoidal hemorrhage,and one with arthritis required a hip prosthesis.  "The incidence rates in children (28 days to 17 years), adults (18–64 years) and elderly patients (≥ 65 years) were 1.3, 3.6, and 12.9 per 100000 per year,respectively. The majority of children and adults had severe underlying diseases, but severe infections were also seen in individuals with no risk factors." |

**Abbreviations**: EOD: early onset disease, EOGBS: Early-Onset Group B Streptococcal Disease, LOD: late onset disease, LOGBS: late onset Group B Streptococcal Disease
Studies may be reported in multiple tables

File 22.3 Universal screening versus risk based: other neonatal health outcomes

| **Review** | **Authors** | **Country** | **Is the outcome reported for separate screening groups?** | **No policy strategy** | **Risk Strategy** | **Screening / Universal strategy** | **Other strategy** | **If outcome data not reported separately, provide details here** | **Is the outcome reported at the level of the neonate / infant / child or maternal.** | **Other details about time frames** | **Is the outcome reported at short-term, medium-term or longer term?** | **Comments** |
| --- | --- | --- | --- | --- | --- | --- | --- | --- | --- | --- | --- | --- |
| Panneflek 2024 (1) | Al Luhidan 2019 (16) | Saudi Arabia | No |  | Universal screening was discontinued in late 2014, and then hospital policy followed an adaptation of the UK RCOG guidelines, giving IAP based on risk factors only.RCOG 2012: IAP to: PTL, PROM, fever, previous infant with invasive GBS infection, and GBS bacteriuria | Between 2004 and 2014, universal screening was carried out between 35 and 37 weeks of gestation. CDC 2002: IAP to: i) carriers of rectovaginal GBS colonisation at 35–37 weeks’ gestation, ii) previous baby with invasive GBS infection, iii) GBS bacteriuria, and iv) if unknown carrier state, presence of PTL, PROM and fever. |  | Total number of early-onset EOD cases with sepsis: 38/55; | Neonate <7 days |  | Short-term outcome | Universal screening conducted from 2004 and 2014 (usually between 35 and 37 weeks of gestation). Universal screening was discontinued in late 2014, and then hospital policy followed an adaptation of the UK RCOG guidelines, giving IAP based on risk factors only. Data not reported separately.  LOGBS definition: GBS from a sterile body site after 7 days of age but before 90 days of age |
| Panneflek 2024 (1) | Al Luhidan 2019 (16) | Saudi Arabia | No |  | Universal screening was discontinued in late 2014, and then hospital policy followed an adaptation of the UK RCOG guidelines, giving IAP based on risk factors only.RCOG 2012: IAP to: PTL, PROM, fever, previous infant with invasive GBS infection, and GBS bacteriuria | Between 2004 and 2014, universal screening was carried out between 35 and 37 weeks of gestation. CDC 2002: IAP to: i) carriers of rectovaginal GBS colonisation at 35–37 weeks’ gestation, ii) previous baby with invasive GBS infection, iii) GBS bacteriuria, and iv) if unknown carrier state, presence of PTL, PROM and fever. |  | Total number of late-onset disease (LOD) cases with sepsis: 6/55; | Infant i.e. from the first month after birth to approximately 12 months of age |  | Other | The age at diagnosis ranged from 0 to 54 days, with a mean of 7.95 (±12.4) days and a median of 1 day. Of those diagnosed with GBS, 69.1% (n = 38) presented with EOD and 30.1% (n = 17) presented with LOD. Data for each strategy is not reported separately but presented according to whether it is EOD or LOD. |
| Panneflek 2024 (1) | Al Luhidan 2019 (16) | Saudi Arabia |  |  | Universal screening was discontinued in late 2014, and then hospital policy followed an adaptation of the UK RCOG guidelines, giving IAP based on risk factors only.RCOG 2012: IAP to: PTL, PROM, fever, previous infant with invasive GBS infection, and GBS bacteriuria | Between 2004 and 2014, universal screening was carried out between 35 and 37 weeks of gestation. CDC 2002: IAP to: i) carriers of rectovaginal GBS colonisation at 35–37 weeks’ gestation, ii) previous baby with invasive GBS infection, iii) GBS bacteriuria, and iv) if unknown carrier state, presence of PTL, PROM and fever. |  | Septic shock. 1 case of EOD with shock. | Neonate <7 days |  | Short-term outcome | Septic shock was documented in 5.5% (n = 3) of patients. Data for each strategy is not reported separately but presented according to whether it is EOD or LOD. |
| Panneflek 2024 (1) | Al Luhidan 2019 (16) | Saudi Arabia |  |  | Universal screening was discontinued in late 2014, and then hospital policy followed an adaptation of the UK RCOG guidelines, giving IAP based on risk factors only.RCOG 2012: IAP to: PTL, PROM, fever, previous infant with invasive GBS infection, and GBS bacteriuria | Between 2004 and 2014, universal screening was carried out between 35 and 37 weeks of gestation. CDC 2002: IAP to: i) carriers of rectovaginal GBS colonisation at 35–37 weeks’ gestation, ii) previous baby with invasive GBS infection, iii) GBS bacteriuria, and iv) if unknown carrier state, presence of PTL, PROM and fever. |  | Septic shock. 2 cases of EOD with shock. | Other (please specify) | "LOGBS definition: GBS from a sterile body site after 7 days of age but before 90 days of age" | Medium-term outcome | Septic shock was documented in 5.5% (n = 3) of patients. Data for each strategy is not reported separately but presented according to whether it is EOD or LOD. |
| Panneflek 2024 (1) | Al Luhidan 2019 (16) | Saudi Arabia | No |  |  |  |  | UTI. 15 cases with UTI in EOD | Neonate <7 days |  | Short-term outcome |  |
| Panneflek 2024 (1) | Al Luhidan 2019 (16) | Saudi Arabia | No |  |  |  |  | UTI. 11 cases with UTI in LOD | Other (please specify) | "LOGBS definition: GBS from a sterile body site after 7 days of age but before 90 days of age" | Medium-term outcome |  |
| Panneflek 2024 (1) | Al Luhidan 2019 (16) | Saudi Arabia | Yes |  | 10/26 UTI cases took place between 2015-2016 | 16/26 UTI cases took place between 2004-2014 |  |  | Other (please specify) | Onset not reported | Unclear/Not reported | The authors state: "Urinary tract infection (UTI) is often a challenging diagnosis in neonates, as the signs are not specific. GBS UTI is not commonly reported in the literature, and UTI rates are high in our study compared with the literature. Most of the cases identified [in the literature] had preexisting renal anomalies.…Although UTI cases are high in our study, the increase in incidence during 2015–2016 cannot be attributed to that. Of the 26 UTI cases, 16 of them took place between 2004 and 2014. Of those, 13 had no other coexisting diagnosis. Ten of the cases were diagnosed between 2015 and 2016, and none of them had another diagnosis. If those cases were to be eliminated, the incidence will drop to 0.15 (originally 0.29) for the period between 2004 and 2014, and to 1.15 (originally 1.79), marking a 7-folds increase in incidence after the universal screening discontinuation" |
| Panneflek 2024 (1) | Al Luhidan 2019 (16) | Saudi Arabia | No |  |  |  |  | "The majority of the study population (94.6%, n = 52) survived without any long-term complication, while 1 neonate (1.8%) had hearing loss, resolved cranial vein thrombosis and developmental delay". Not clear if this neonate was EOD or LOD or which screening strategy. |  |  | Longer-term outcome | Long-term |
| Panneflek 2024 (1) | Alarcon et al. 2004 (2) | Spain | No | 1992-1995: not clear what the policy is but it predates the CDC 1996 guidelines so likely this is no policy |  |  | Authors refer to implementation of CDC 1996 in period 2 and the refer directly to CDC 1996 in period 3 | 27/41 cases had early onset E Coli infection. Of these 40/41 had "known duration of rupture of membranes and 28/41 had information about IAP use available for review" |  |  |  | Three time periods  No EOGBS data reported |
| Li 2020 (17), Panneflek 2024 (1) | Björklund 2017 (18) | Finland | Yes |  | Length of stay of the newborns identified with suspected or confirmed GBS-EOD by ward and phase, mean (SD): Pediatric care unit: 3.82 (3.34) days, n=65; Mother-and-baby ward: 3.48 (1.34) days, n=63; NICU: 6.83 (4.45), n=6. |  | Length of stay of the newborns identified with suspected or confirmed GBS-EOD by ward and phase, mean (SD): Pediatric care unit: 2.69 (2.3) days, n=84; Mother-and-baby ward: 4.59 (1.85) days, n=76; NICU: 5 (1.41), n=2. | "The mean lengths of stay (LOS) in the pediatric care unit were 3.82 days in phase 1 and 2.69 days in phase 2 (p¼ 0.02), and on the mother-and-baby ward, mean LOS were 3.48 days in phase 1 and 4.59 days in phase 2 (p50.01). Some babies were only on one of the wards, and others were on more than one ward during their stay. Eight babies (6 in Phase 1 and 2 in Phase 2) were also transferred to the NICU in the nearby University Hospital." |  |  |  |  |
| Panneflek 2024 (1) | Chan 2023 (19) | Hong Kong, PRC | Yes |  | 92 GBS late-onset cases / 130498 live births (i.e. incidence of late onset GBS sepsis: 0.71, 95% CI 0.57-0.86 per 1000 live births | 131 GBS late-onset cases/ 337081 |  |  |  |  |  | "Clinical-­ risk based screening period (2009–­ 2011) and during the universal culture-­ based screening period (2012–­ 2020)"  LOGBS definition: day 8 to day 120 of life |
| Panneflek 2024 (1) | Chan 2023 (19) | Hong Kong, PRC | Yes |  | Fatality: 28 cases/328 confirmed EOS | Fatality: 21 cases/194 confirmed EOS |  | Cases reported here are confirmed EOS. Data extracted from linked paper - Wang 2023 Epidemiologic Changes of Neonatal Early-onset Sepsis After the Implementation of Universal Maternal Screening for Group B Streptococcus in Hong Kong Pediatric Infectious Disease Journal 2023;42(10):914-920 | Neonate <7 days |  | Short-term outcome | Authors conclude that "Pathogen profile of EOS changed with the implementation of universal GBS screening. S. bovis has emerged as a more common pathogen associated with the risk of meningitis. IAP may not be as effective in reducing EOS rate among infants born <34 weeks as compared with those ≥34 weeks, and newer strategies may be needed." |
| Panneflek 2024 (1) | Coco 2002 (20) | USA | Yes |  | Newborn length of stay (SD): 52.4 (26.8) hours | Newborn length of stay (SD): 53.5 (24.8) hours |  | Newborn Length of Stay by Prenatal Screening (n=154) and Risk Factor (n=155) Groups. Authors report no significant difference between groups (P=0.71) | Neonate <7 days |  | Short-term outcome |  |
| Panneflek 2024 (1) | Coco 2002 (20) | USA | Yes |  | Newborn charges (SD): $1061 (1491) US dollars | Newborn charges (SD): $1186 (1406) US dollars |  | Newborn Hospital Charges, by Prenatal Screening (n=154) and Risk Factor (n=155) Groups. Authors report no significant difference between groups (P=0.45) | Neonate <7 days |  | Short-term outcome |  |
| Panneflek 2024 (1) | Eberly 2009 (5) | USA | No |  |  |  |  | Isolated urinary tract infection: 22/860 cases of early onset GBS | Neonate <7 days |  | Short-term outcome |  |
| Panneflek 2024 (1) | Eberly 2009 (5) | USA | No |  |  |  |  | Cellulitis/abscess: 4/860 cases of early onset GBS | Neonate <7 days |  | Short-term outcome |  |
| Panneflek 2024 (1) | Eberly 2009 (5) | USA | No |  |  |  |  | Osteomyelitis: 1/860 cases of early onset GBS | Neonate <7 days |  | Short-term outcome |  |
| Li 2020 (17), Hasperhoven 2020 (13), Panneflek 2024 (1) | Gilson 2000 (21) | USA | Yes |  | Overall length of stay (SD): 2 (2) days | Overall length of stay (SD): 2.3 (3.9) days |  |  |  |  |  | Gilson 2020 report that "length of stay was not different between groups, except for those infants in the control group with documented GBS sepsis" Gilson 2020 also points out that this is the "first study to compare a cohort of patients managed under the two protocols concomitantly" |
| Li 2020 (17), Hasperhoven 2020 (13), Panneflek 2024 (1) | Gilson 2000 (21) | USA | Yes |  | Overall length of stay (SD) for culture +ve for GBS: 0 days (n=0) | Overall length of stay (SD) for culture +ve for GBS: 9.8 (5.5) days (n=4) |  |  |  |  |  | Gilson 2020 report that "length of stay was not different between groups, except for those infants in the control group with documented GBS sepsis, p<0.001". Gilson 2020 also points out that this is the "first study to compare a cohort of patients managed under the two protocols concomitantly" |
| Li 2020 (17), Hasperhoven 2020 (13), Panneflek 2024 (1) | Gilson 2000 (21) | USA | No |  |  |  |  | No cases of adverse reactions to antibiotics were noted | Unclear / not specified |  | Short-term outcome |  |
| Li 2020 (17), Panneflek 2024 (1) | Hafner 1998 (22) | Austria | Yes |  | In period A, 2 infants had to be transferred to another center for extracorporeal membrane oxygenation; another 3 required conventional respirator treatment for several days. | In period B, only 2 infants needed respirator treatment |  | Respiratory disease | Neonate <7 days |  |  | In period A (risk screening) "Five infants needed respirator treatment. Two of them did not respond to conventional respirator treatment and had to be transferred to a specialized center for extracorporeal membrane oxygenation, which kept them alive." In period B, Both infants needed respirator treatment for several days. Although the mother of 1 of the 2 had been given an antibiotic because of fever, severe sepsis occurred. This baby made a good recovery and had normal findings at the follow-up neurodevelopmental examinations. The mother of the other preterm infant with extremely severe sepsis had not received an antibiotic. Bilateral paraventricular leucomalacia and subsequent moderate hemiplegia developed in this baby. |
| Li 2020 (17), Panneflek 2024 (1) | Hafner 1998 (22) | Austria | Yes |  | Survivors with neurological abnormalities: 2 | Survivors with neurological abnormalities: 1 |  | Neurological outcomes | Child i.e. from 1-18 years of age |  | Longer-term outcome | In period A (risk screening) "There were no pathologic findings in 18 of the infected infants when follow-up neurodevelopmental examinations were performed at the end of the second year of life. One patient from the extracorporeal membrane oxygenation group had slight hemiparesis of the foot and 1 had epileptiform attacks, but there was no causal relationship between these problems and the group B streptococcal infection." In period B, In period B, Both infants needed respirator treatment for several days. Although the mother of 1 of the 2 had been given an antibiotic because of fever, severe sepsis occurred. This baby made a good recovery and had normal findings at the follow-up neurodevelopmental examinations. The mother of the other preterm infant with extremely severe sepsis had not received an antibiotic. Bilateral paraventricular leucomalacia and subsequent moderate hemiplegia developed in this baby |
| Panneflek 2024 (1) | Lee 2021 (23) | Singapore | No |  |  |  |  | Nine neonates were diagnosed with GBS sepsis between 2001 and 2015. All of these cases were followed up (aged 4–11 years), with two experiencing long-term complications: one with profound hearing loss and another with intellectual impairment. The remaining 7 cases were reported as 'well'. Outcomes were not reported separately by screening strategy. |  |  |  |  |
| Newly identified | Riley 2003 (24) * | USA | No |  |  |  |  | "There was one adverse drug reaction, which was a rash to penicillin. | Unclear / not specified |  | Other | "At the academic hospital using the risk-based strategy, there is one physician group and one midwifery group providing care to the entire population. At the community hospital, women were counted in the culture- or risk-based strategy based on the intended strategy for their provider practice" |

**Abbreviations**: EOD: early onset disease, EOGBS: Early-Onset Group B Streptococcal Disease, LOD: late onset disease, LOGBS: late onset Group B Streptococcal Disease
* Study excluded in Panneflek 2024 as outcomes not reported
Studies may be reported in multiple tables

File 22.4 Universal screening versus other: other neonatal health outcomes

| **Review** | **Authors** | **Country** | **Is the outcome reported for separate screening groups?** | **No policy strategy** | **Risk Strategy** | **Screening / Universal strategy** | **Other strategy** | **If outcome data not reported separately, provide details here** | **Is the outcome reported at the level of the neonate / infant / child or maternal.** | **Other details about time frames** | **Is the outcome reported at short-term, medium-term or longer term?** | **Comments** |
| --- | --- | --- | --- | --- | --- | --- | --- | --- | --- | --- | --- | --- |
| Hasperhoven 2020 (17), Panneflek 2024 (1) | Hung 2018 (25) | Taiwan | No |  |  |  |  | "A total of 154,088 pregnant women underwent GBS screening and delivered naturally. Among them, 30,176 had a positive screening result, with a GBS prevalence of 19.58% (Table 1). In terms of morbidity risk, the percentage of newborns contracting early-onset GBS related diseases (including sepsis, meningitis, and pneumonia) and delivered by women who underwent screening and delivered naturally was 0.02%. Of these, the neonatal morbidity rate for GBS-positive mothers was 0.03%, whereas that for GBS-negative mothers was 0.01%, and the difference was statistically significant. |  |  |  | Data regarding EOGBS related diseases (including sepsis, meningitis and pneumonia) is not reported separately. |
| Li 2020 (17), Panneflek 2024 (1) | Locksmith 1999 (26) | USA | No |  |  |  |  | Adverse events: The incidence of major dermatologic reactions, such as Stevens-Johnson syndrome and toxic epidermal necrolysis, was also zero. | Unclear / not specified |  | Short-term outcome |  |
| Panneflek 2024 (1) | Lukacs 2012 (9) | USA | Yes | CFR: 2.3% (n=1116047) |  | CFR: 3.2% (n=608569) | CFR: 3.1% (n=796633) | Case fatality rate based on sepsis hospitalisation in infants aged < 3 months | Other (please specify) | Characteristics of sepsis hospitalisation in infants <3 months | Other | Among the 12% of sepsis hospitalizations that involved a pathogen-specific ICD-9-CM code (Table I and Appendix), 38% were due to Streptococcus spp (9% identified as GBS). There were no significant differences in the percentage of hospitalizations with meningitis or respiratory failure, the percentage of fatal cases, or geographic distribution.  LOGBS definition: late-onset sepsis: up to 90 days of age and after first week of life. Authors note that "we could not determine the date of onset of sepsis to distinguish early-onset sepsis from late-onset sepsis, if more than one episode of sepsis occurred during a single newborn hospital course or if an infant was hospitalized more than once" |
| Panneflek 2024 (1) | Lukacs 2012 (9) | USA | Yes | Respiratory failure: 0.8% (n=1116047) |  | Respiratory failure: 1.2% (n=608569) | Respiratory failure: 1.2% (n=796633) | Respiratory failure based on sepsis hospitalisation in infants aged < 3 months | Other (please specify) | Characteristics of sepsis hospitalisation in infants <3 months | Other | Among the 12% of sepsis hospitalizations that involved a pathogen-specific ICD-9-CM code (Table I and Appendix), 38% were due to Streptococcus spp (9% identified as GBS).There were no significant differences in the percentage of hospitalizations with meningitis or respiratory failure, the percentage of fatal cases, or geographic distribution.  LOGBS definition: late-onset sepsis: up to 90 days of age and after first week of life. Authors note that "we could not determine the date of onset of sepsis to distinguish early-onset sepsis from late-onset sepsis, if more than one episode of sepsis occurred during a single newborn hospital course or if an infant was hospitalized more than once" |
| Hasperhoven 2020 (13), Panneflek 2024 (1) | Phares 2008 (27) | USA | No |  |  |  |  | "Childhood Disease. Surveillance identified 233 cases of invasive group B streptococcal disease in children aged 90 days through 14 years. Incidence of pediatric disease was 0.56 per 100 000 (yearly range, 0.37-0.73 per 100 000), and no sustained change over time was observed. Sixty-one percent (143/233) of these cases occurred in children aged 90 days through 12 months, and the remaining third (90/233) were evenly distributed among children aged 1 through 14 years. Among all 233 children, bacteremia without focus was the most common syndrome, accounting for 58% of the cases, followed by meningitis (19%), pneumonia (7%), septic arthritis (5%), and peritonitis (4%). The proportion with known outcome who died was lower among children aged 90 days through 12 months (4/142 [3%]) than among those aged 1 through 14 years (11/85 [13%]). Only 11% (16/143) of children aged 90 days through 12 months had an underlying condition (excluding preterm birth), but 44% (40/90) of children aged 1 through 14 years had at least 1 condition. Among these 40 older children, the most common underlying factors were neurologic disorders (25%), immunosuppressive conditions (23%), asthma (23%), malignancy (15%), and renal disease (13%)" | Other (please specify) | Infant and Child data presented | Other | Data not reported for separate screening strategies. |
| Hasperhoven 2020 (13), Panneflek 2024 (1) | Phares 2008 (27) | USA | Unclear |  |  |  |  | "Surveillance identified 1036 cases of late-onset disease. Incidence remained generally stable, averaging 0.34 per 1000 live births. Similar to early onset disease, incidence of late-onset disease increased slightly during 2003-2005 [after the introduction of the 2002 guidelines] |  | Late-onset disease | Short-term outcome | LOGBS definition: 7 - 89 days old |
| Panneflek 2024 (1) | Wicker 2019 (28) | Germany | No |  |  |  |  | Paediatic reporting: "Data regarding age at disease onset was available for 214 out of 226 cases (94.7%). Among those, 92 (43%) patients were below 7 days of age and 122 (57%) patients were 7–90 days old. The ratio of EOD to LOD was 0.75 (92:122)....Calculation based on the pediatric reporting gives an incidence of 0.15 for EOD and 0.19 for LOD per 1000 live births, respectively. From this, we estimate detection sensitivity to be 50% for pediatric reporting (95% CI, 43%–58%) and 38% for laboratory reporting (95% CI, 32%–44%). | Other (please specify) | Based on the definition of late-onset disease: "late-onset disease (LOD) occurring at 7–90 days of age" | Other | Late onset incidence reported. Data in this study compared paediatric reporting from ESPED system with lab based reporting system (RKI). The authors report that they "compared our results with those from a previous study by employing an equivalent design (2001–2003). We detected a 32% reduction in GBS incidence, from 0.47 per 1000 live births (n = 679) in 2001–2003 to 0.34 per 1000 live births (n = 450) in 2009–2010. This decline primarily is tied to a reduced number of GBS cases in children under 1 week of age. In 2009–2010, the ratio of early-onset disease to late-onset disease reversed from 1.52 (206:136), as determined in 2001–2003, to 0.75 (92:122). This study is the first to assess changes in the incidence of invasive GBS in Germany after the implementation of the guidelines for intrapartum prophylaxis for pregnant women colonized with GBS". Authors also note that "A separate computation of either EOD- or LOD-specific incidence by means of the CRC analysis was not possible because RKI only was allowed to receive pseudonymized data. Unfortunately, these data did not include the exact age at onset of infection."  LOGBS definition: 7- 90 days of age. |

**Abbreviations**: EOD: early onset disease, EOGBS: Early-Onset Group B Streptococcal Disease, LOD: late onset disease, LOGBS: late onset Group B Streptococcal Disease
Studies may be reported in multiple tables

### File 22.5 Risk based versus other: other neonatal health outcomes

| **Review** | **Authors** | **Country** | **Is the outcome reported for separate screening groups?** | **No policy strategy** | **Risk Strategy** | **Screening / Universal strategy** | **Other strategy** | **If outcome data not reported separately, provide details here** | **Is the outcome reported at the level of the neonate / infant / child or maternal.** | **Other details about time frames** | **Is the outcome reported at short-term, medium-term or longer term?** | **Comments** |
| --- | --- | --- | --- | --- | --- | --- | --- | --- | --- | --- | --- | --- |
| Li 2020 (17), Hasperhoven 2020 (13), Panneflek 2024 (1) | Angstetra 2007 (29) | Australia | Yes |  |  | 3.2 days | 3.2 days | Length of stay (days) |  |  |  | In 2004–2006 all women were offered rectovaginal swab for GBS detection at 34-37 weeks gestation. IAP was offered if culture was positive. Limited information about the strategy. Between 1994–2002. IAP offered if previous infant with GBS-EOS, GBS bacteriuria in current pregnancy, preterm labor of less than 37 weeks spontaneous or induced (unless known GBS status within last 2 weeks), intrapartum fever 38°C or more on 2 occasions 2 h apart regardless of GBS status and prolonged rupture of membranes of 18 h or more where GBS status is unknown. Note: screening-based protocol was introduced in mid-2003. Data from Panneflek have categorised these as Universal vs other screening; Hasperhoven 2020 and Li 2020 have compared the two strategies as Universal vs risk-based screening. |
| Newly identified | Daniels 2022 (30) | England, UK | No |  |  |  |  | No serious adverse events were reported. | Other (please specify) | Outcomes included SAE in the mother or newborn. None reported. |  |  |

**Abbreviations**: EOD: early onset disease, EOGBS: Early-Onset Group B Streptococcal Disease, LOD: late onset disease, LOGBS: late onset Group B Streptococcal Disease
Studies may be reported in multiple tables

### File 22.6 Universal screening versus universal screening (timing): other neonatal health outcomes

| **Review** | **Authors** | **Country** | **Is the outcome reported for separate screening groups?** | **No policy strategy** | **Risk Strategy** | **Screening / Universal strategy** | **Other strategy** | **If outcome data not reported separately, provide details here** | **Is the outcome reported at the level of the neonate / infant / child or maternal.** | **Other details about time frames** | **Is the outcome reported at short-term, medium-term or longer term?** | **Comments** |
| --- | --- | --- | --- | --- | --- | --- | --- | --- | --- | --- | --- | --- |
| Panneflek 2024 (1) | El Helali 2019 (31) | France | Yes |  |  | Timing of GBS determination i.e., Antenatal vs Intrapartum |  | Average length of stay for all newborns (days): Antenatal group: 5.37 (4.94) days compared with Intrapartum group: 4.38 (3.83) days. Authors note: "The average length of stay for delivery declined one day between the two periods owing to changes in postnatal practices of newborns’ surveillance" | Other (please specify) | Maternal and neonate | Short-term outcome |  |
| Panneflek 2024 (1) | El Helali 2019 (31) | France | Yes |  |  | Timing of GBS determination i.e., Antenatal vs Intrapartum |  | Average length of stay for all newborns (days): Antenatal group: 10.0 (6.1) days compared with Intrapartum group: 9.2 (3.5) days. |  |  |  |  |

**Abbreviations**: CNS: central nervous system; EOD: early onset disease, EOGBS: Early-Onset Group B Streptococcal Disease, LOD: late onset disease, LOGBS: late onset Group B Streptococcal Disease
Studies may be reported in multiple tables

File 22.7 Other strategies versus no strategy: other neonatal health outcomes

| **Review** | **Authors** | **Country** | **Is the outcome reported for separate screening groups?** | **No policy strategy** | **Risk Strategy** | **Screening / Universal strategy** | **Other strategy** | **If outcome data not reported separately, provide details here** | **Is the outcome reported at the level of the neonate / infant / child or maternal.** | **Other details about time frames** | **Is the outcome reported at short-term, medium-term or longer term?** | **Comments** |
| --- | --- | --- | --- | --- | --- | --- | --- | --- | --- | --- | --- | --- |
| Hasperhoven 2020 (13) Panneflek 2024 (1) | Darlow 2016 (32) | New Zealand | No |  |  |  |  | 29 cases of GBS sepsis in the first 48 hours; (Two further cases of GBS sepsis presented after the first 48 h, at 62 and 96 h of age, the latter with confirmed meningitis, and both survived.) | Neonate <7 days |  | Short-term outcome | "the incidence of early-onset GBS sepsis in New Zealand appeared to have more than halved from 0.5 per 1000 live births in 1998–1999 when LMCs and obstetric units used various screening and risk-based prevention protocols (see Grimwood et al., 2002, reference number 6) to 0.23 per 1000 in 2009–2011 when a nationally agreed single policy was available." |
| Panneflek 2024 (1) | Horváth 2013 (33) | Hungary | Yes | 31 neonates |  |  | 8 neonates | Sepsis | Neonate <7 days |  | Short-term outcome | P = 0.001; OR, 0.27; 95% CI, 0.12–0.58. P values were calculated by the χ2 test. "Of the 8 women whose newborns incurred sepsis, only 2 tested positive for GBS between 30 and 32 weeks, but 7 presented 2 or more factors predicting GBS disease in their newborns. The prophylactic antibiotic treatment could not be administered in 4 women because of a very short labor and delivery duration. The only newborn who died was severely premature, with a gestational age of only 27 weeks and a very low birth weight at delivery, and its mother was probably heavily infected with GBS. All other affected newborns recovered without lasting adverse effects. The mean recovery period was 15.6 days (range, 10–36 days)." |
| Panneflek 2024 (1) | Horváth 2013 (33) | Hungary | Yes | 0 |  |  | 0 | LOGBS | Other (please specify) | Just refer to LOGBS | Medium-term outcome | Of 8 newborns (0.03%) who incurred neonatal sepsis, the only one who died was markedly premature, "Regarding late-onset GBS infection, no case has occurred at Markusovszky Teaching Hospital in the last 16 years" |
| Panneflek 2024 (1) | Lukacs 2012 (9) | USA | Yes | CFR: 2.3% (n=1116047) |  | CFR: 3.2% (n=608569) | CFR: 3.1% (n=796633) | Case fatality rate based on sepsis hospitalisation in infants aged < 3 months | Other (please specify) | Characteristics of sepsis hospitalisation in infants <3 months | Other | Among the 12% of sepsis hospitalizations that involved a pathogen-specific ICD-9-CM code (Table I and Appendix), 38% were due to Streptococcus spp (9% identified as GBS)...There were no significant differences in the percentage of hospitalizations with meningitis or respiratory failure, the percentage of fatal cases, or geographic distribution.  LOGBS definition: late-onset sepsis: up to 90 days of age and after first week of life. Authors note that "we could not determine the date of onset of sepsis to distinguish early-onset sepsis from late-onset sepsis, if more than one episode of sepsis occurred during a single newborn hospital course or if an infant was hospitalized more than once" |
| Panneflek 2024 (1) | Lukacs 2012 (9) | USA | Yes | Respiratory failure: 0.8% (n=1116047) |  | Respiratory failure: 1.2% (n=608569) | Respiratory failure: 1.2% (n=796633) | Respiratory failure based on sepsis hospitalisation in infants aged < 3 months | Other (please specify) | Characteristics of sepsis hospitalisation in infants <3 months | Other | Among the 12% of sepsis hospitalizations that involved a pathogen-specific ICD-9-CM code (Table I and Appendix), 38% were due to Streptococcus spp (9% identified as GBS). There were no significant differences in the percentage of hospitalizations with meningitis or respiratory failure, the percentage of fatal cases, or geographic distribution.  LOGBS definition: late-onset sepsis: up to 90 days of age and after first week of life. Authors note that "we could not determine the date of onset of sepsis to distinguish early-onset sepsis from late-onset sepsis, if more than one episode of sepsis occurred during a single newborn hospital course or if an infant was hospitalized more than once" |
| Panneflek 2024 (1) | Poulain 1997 (34) | France | No |  |  |  |  | The chemoprophylaxis was really done in only 12 of the 19 (63%). When ampicillin was not administrated, this was due to protocol violation or because the obstetrical risk of transmission was noted nearly the time of delivery. There was no adverse effect of amoxicillin. | Neonate <7 days |  | Short-term outcome |  |
| Panneflek 2024 (1) | Trijbels-Smeulders 2006 (35) | The Netherlands | No |  |  |  |  | Number of cases with early-onset (< /=7 d) sepsis: 113/142 | Neonate <7 days |  | Short-term outcome |  |
| Panneflek 2024 (1) | Trijbels-Smeulders 2006 (35) | The Netherlands | No |  |  |  |  | Number of cases with late-onset (>7 d) sepsis: 22/56 | Unclear / not specified |  | Unclear/Not reported | In this paper, authors refer to late-onset as > 7days; no further details given. |
| Panneflek 2024 (1) | Trijbels-Smeulders 2007 (36) | The Netherlands | Yes |  |  |  |  | Proven early onset GBS sepsis incidence: 157 cases in 1997/98 and 196 in 1999-2001. Corrected incidence (corrected for under-reporting to the Dutch Paediatric Surveillance Unit by using the capture-recapture technique which the authors note may lead to discrepancies in the data) (95% CI): 0.54 (0.42 to 0.67) in 1997/98 and 0.36 (0.32 to 0.41) in 1999-2001. Risk ratio (min-max) (calculated with the lowest and highest estimates of incidence): 0.67 (0.48 to 0.98) | Neonate <7 days |  | Short-term outcome | Definitions of timeframes: very early onset (<12 h); late early onset (12 h – <7 days) and late onset (7–90 days).  LOGBS definition: 7 - 27 days after birth |
| Panneflek 2024 (1) | Trijbels-Smeulders 2007 (36) | The Netherlands | Yes |  |  |  |  | Proven very early onset GBS sepsis incidence: 128 cases in 1997/98 and 140 in 1999-2001. Corrected incidence (corrected for under-reporting to the Dutch Paediatric Surveillance Unit by using the capture-recapture technique which the authors note may lead to discrepancies in the data) (95% CI): 0.38 (0.30 to 0.46) in 1997/98 and 0.28 (0.22 to 0.34) in 1999-2001. Risk ratio (min-max) (calculated with the lowest and highest estimates of incidence): 0.74 (0.48 to 1.13) | Neonate <7 days | very early onset (<12 h) | Short-term outcome |  |
| Panneflek 2024 (1) | Trijbels-Smeulders 2007 (36) | The Netherlands | Yes |  |  |  |  | Proven late early onset GBS sepsis incidence: 29 cases in 1997/98 and 56 in 1999-2001. Corrected incidence (corrected for under-reporting to the Dutch Paediatric Surveillance Unit by using the capture-recapture technique which the authors note may lead to discrepancies in the data) (95% CI): 0.13 (0.07 to 0.19) in 1997/98 and 0.09 (no under-reporting) in 1999-2001. Risk ratio (min-max) (calculated with the lowest and highest estimates of incidence): 0.69 (0.47 to 1.30) | Neonate <7 days | late early onset (12 h – <7 days) | Short-term outcome |  |
| Panneflek 2024 (1) | Trijbels-Smeulders 2007 (36) | The Netherlands | Yes |  |  |  |  | Proven late onset GBS sepsis incidence: 27 cases in 1997/98 and 50 in 1999-2001. Corrected incidence (corrected for under-reporting to the Dutch Paediatric Surveillance Unit by using the capture-recapture technique which the authors note may lead to discrepancies in the data) (95% CI): 0.14 (0.07 to 0.27) in 1997/98 and 0.14 (0.06 to 0.23) in 1999-2001. Risk ratio (min-max) (calculated with the lowest and highest estimates of incidence): 1.0 (0.22 to 32.9) | Other (please specify) | late onset (7–90 days) | Other | late onset (7–90 days) |
| Panneflek 2024 (1) | Trijbels-Smeulders 2007 (36) | The Netherlands | Yes |  |  |  |  | Probable early onset GBS sepsis incidence: 190 cases in 1997/98 and 313 in 1999-2001. Corrected incidence (corrected for under-reporting to the Dutch Paediatric Surveillance Unit by using the capture-recapture technique which the authors note may lead to discrepancies in the data) (95% CI): 1.3 (0.61 to 2.1) in 1997/98 and 1.4 (0.9 to 1.9) in 1999-2001. Risk ratio (min-max) (calculated with the lowest and highest estimates of incidence): 1.08 (0.43 to 3.11) | Neonate <7 days |  | Short-term outcome |  |
| Panneflek 2024 (1) | Trijbels-Smeulders 2007 (36) | The Netherlands | Yes |  |  |  |  | Probable very early onset GBS sepsis incidence: 159 cases in 1997/98 and 257 in 1999-2001. Corrected incidence (corrected for under-reporting to the Dutch Paediatric Surveillance Unit by using the capture-recapture technique which the authors note may lead to discrepancies in the data) (95% CI): 0.99 (0.44 to 1.53) in 1997/98 and 1.1 (0.65 to 1.52) in 1999-2001. Risk ratio (min-max) (calculated with the lowest and highest estimates of incidence): 1.11 (0.42 to 3.45) | Neonate <7 days | Very early onset (<12 h) | Short-term outcome |  |
| Panneflek 2024 (1) | Trijbels-Smeulders 2007 (36) | The Netherlands | Yes |  |  |  |  | Probable late early onset GBS sepsis incidence: 31 cases in 1997/98 and 56 in 1999-2001. Corrected incidence (corrected for under-reporting to the Dutch Paediatric Surveillance Unit by using the capture-recapture technique which the authors note may lead to discrepancies in the data) (95% CI): 0.4 (0.0 to 1.1) in 1997/98 and 0.3 (0.06 to 0.53) in 1999-2001. Risk ratio (min-max) (calculated with the lowest and highest estimates of incidence): 0.75 (0.05 to 53.0) | Neonate <7 days | Late early onset (12 h – <7 days) | Short-term outcome |  |
| Panneflek 2024 (1) | Trijbels-Smeulders 2007 (36) | The Netherlands | Yes |  |  |  |  | Probable late onset GBS sepsis incidence: 4 cases in 1997/98 and 5 in 1999-2001. Corrected incidence (corrected for under-reporting to the Dutch Paediatric Surveillance Unit by using the capture-recapture technique which the authors note may lead to discrepancies in the data) (95% CI): 0.01 in 1997/98 and 0.02 (0.0 to 0.04) in 1999-2001. Risk ratio (min-max) (calculated with the lowest and highest estimates of incidence): 2.0 (0.0 to 4.0) | Other (please specify) | Late onset (7–90 days) | Other |  |

**Abbreviations**: EOD: early onset disease, EOGBS: Early-Onset Group B Streptococcal Disease, LOD: late onset disease, LOGBS: late onset Group B Streptococcal Disease
Studies may be reported in multiple tables

References

1. Panneflek TJR, Hasperhoven GF, Chimwaza Y, Allen C, Lavin T, Te Pas AB, et al. Intrapartum antibiotic prophylaxis to prevent Group B streptococcal infections in newborn infants: a systematic review and meta-analysis comparing various strategies. *EClinicalMedicine* 2024; 74:102748.

2. Alarcon A, Pena P, Salas S, Sancha M, Omenaca F. Neonatal early onset Escherichia coli sepsis: trends in incidence and antimicrobial resistance in the era of intrapartum antimicrobial prophylaxis. *Pediatr Infect Dis J* 2004; 23 4:295-9.

3. Bauserman MS, Laughon MM, Hornik CP, Smith PB, Benjamin DK, Jr., Clark RH, et al. Group B Streptococcus and Escherichia coli infections in the intensive care nursery in the era of intrapartum antibiotic prophylaxis. *Pediatr Infect Dis J* 2013; 32 3:208-12.

4. Cho CY, Tang YH, Chen YH, Wang SY, Yang YH, Wang TH, et al. Group B Streptococcal infection in neonates and colonization in pregnant women: An epidemiological retrospective analysis. *J Microbiol Immunol Infect* 2019; 52 2:265-72.

5. Eberly MD, Rajnik M. The effect of universal maternal screening on the incidence of neonatal early-onset group B streptococcal disease. *Clin Pediatr (Phila)* 2009; 48 4:369-75.

6. Gibbs RS, McDuffie RS, Jr., McNabb F, Fryer GE, Miyoshi T, Merenstein G. Neonatal group B streptococcal sepsis during 2 years of a universal screening program. *Obstet Gynecol* 1994; 84 4:496-500.

7. Jeffery HE, Moses Lahra M. Eight-year outcome of universal screening and intrapartum antibiotics for maternal group B streptococcal carriers. *Pediatrics* 1998; 101 1:E2.

8. Katz VL, Moos MK, Cefalo RC, Thorp JM, Jr., Bowes WA, Jr., Wells SD. Group B streptococci: results of a protocol of antepartum screening and intrapartum treatment. *Am J Obstet Gynecol* 1994; 170 2:521-6.

9. Lukacs SL, Schrag SJ. Clinical sepsis in neonates and young infants, United States, 1988-2006. *J Pediatr* 2012; 160 6:960-5 e1.

10. Matsubara K, Hoshina K, Suzuki Y. Early-onset and late-onset group B streptococcal disease in Japan: a nationwide surveillance study, 2004-2010. *Int J Infect Dis* 2013; 17 6:e379-84.

11. Sutkin G, Krohn MA, Heine RP, Sweet RL. Antibiotic prophylaxis and non-group B streptococcal neonatal sepsis. *Obstet Gynecol* 2005; 105 3:581-6.

12. Bjornsdottir ES, Martins ER, Erlendsdottir H, Haraldsson G, Melo-Cristino J, Ramirez M, et al. Group B Streptococcal Neonatal and Early Infancy Infections in Iceland, 1976-2015. *Pediatr Infect Dis J* 2019; 38 6:620-4.

13. Hasperhoven GF, Al-Nasiry S, Bekker V, Villamor E, Kramer B. Universal screening versus risk-based protocols for antibiotic prophylaxis during childbirth to prevent early-onset group B streptococcal disease: a systematic review and meta-analysis. *BJOG* 2020; 127 6:680-91.

14. O'Sullivan CP, Lamagni T, Patel D, Efstratiou A, Cunney R, Meehan M, et al. Group B streptococcal disease in UK and Irish infants younger than 90 days, 2014-15: a prospective surveillance study. *Lancet Infect Dis* 2019; 19 1:83-90.

15. Trollfors B MF, Gudjonsdottir MJ, et al. . Group B streptococcus - a pathogen not restricted to neonates. *IJID Reg* 2022; 4:171-5.

16. Al Luhidan L, Madani A, Albanyan EA, Al Saif S, Nasef M, AlJohani S, et al. Neonatal Group B Streptococcal Infection in a Tertiary Care Hospital in Saudi Arabia: A 13-year Experience. *Pediatr Infect Dis J* 2019; 38 7:731-4.

17. Li QY, Wang DY, Li HT, Liu JM. Screening-based and Risk-based Strategy for the Prevention of Early-onset Group B Streptococcus/Non-group B Streptococcus Sepsis in the Neonate: A Systematic Review and Meta-analysis. *Pediatr Infect Dis J* 2020; 39 8:740-8.

18. Bjorklund V, Nieminen T, Ulander VM, Ahola T, Saxen H. Replacing risk-based early-onset-disease prevention with intrapartum group B streptococcus PCR testing. *J Matern Fetal Neonatal Med* 2017; 30 3:368-73.

19. Chan YTV, Lau SYF, Hui SYA, Ma T, Kong CW, Kwong LT, et al. Incidence of neonatal sepsis after universal antenatal culture-based screening of group B streptococcus and intrapartum antibiotics: A multicentre retrospective cohort study. *BJOG* 2023; 130 1:24-31.

20. Coco AS. Comparison of two prevention strategies for neonatal group B streptococcal disease. *J Am Board Fam Pract* 2002; 15 4:272-6.

21. Gilson GJ, Christensen F, Romero H, Bekes K, Silva L, Qualls CR. Prevention of group B streptococcus early-onset neonatal sepsis: comparison of the Center for Disease Control and prevention screening-based protocol to a risk-based protocol in infants at greater than 37 weeks' gestation. *J Perinatol* 2000; 20 8 Pt 1:491-5.

22. Hafner E, Sterniste W, Rosen A, Schuchter K, Plattner M, Asboth F, et al. Group B streptococci during pregnancy: a comparison of two screening and treatment protocols. *Am J Obstet Gynecol* 1998; 179 3 Pt 1:677-81.

23. Lee J, Naiduvaje K, Chew KL, Charan N, Chan YH, Lin RT, et al. Preventing early-onset group B streptococcal sepsis: clinical risk factor-based screening or culture-based screening? *Singapore Med J* 2021; 62 1:34-8.

24. Riley L, Appollon K, Haider S, Chan-Flynn S, Cohen A, Ecker J, et al. "Real World" compliance with strategies to prevent early-onset group B streptococcal disease. *J Perinatol* 2003; 23 4:272-7.

25. Hung LC, Kung PT, Chiu TH, Su HP, Ho M, Kao HF, et al. Risk factors for neonatal early-onset group B streptococcus-related diseases after the implementation of a universal screening program in Taiwan. *BMC Public Health* 2018; 18 1:438.

26. Locksmith GJ, Clark P, Duff P. Maternal and neonatal infection rates with three different protocols for prevention of group B streptococcal disease. *Am J Obstet Gynecol* 1999; 180 2 Pt 1:416-22.

27. Phares CR, Lynfield R, Farley MM, Mohle-Boetani J, Harrison LH, Petit S, et al. Epidemiology of invasive group B streptococcal disease in the United States, 1999-2005. *JAMA* 2008; 299 17:2056-65.

28. Wicker E, Lander F, Weidemann F, Hufnagel M, Berner R, Krause G. Group B Streptococci: Declining Incidence in Infants in Germany. *Pediatr Infect Dis J* 2019; 38 5:516-9.

29. Angstetra D, Ferguson J, Giles WB. Institution of universal screening for Group B streptococcus (GBS) from a risk management protocol results in reduction of early-onset GBS disease in a tertiary obstetric unit. *Aust N Z J Obstet Gynaecol* 2007; 47 5:378-82.

30. Daniels JP, Dixon E, Gill A, Bishop J, Wilks M, Millar M, et al. Rapid intrapartum test for maternal group B streptococcal colonisation and its effect on antibiotic use in labouring women with risk factors for early-onset neonatal infection (GBS2): cluster randomised trial with nested test accuracy study. *BMC Medicine* 2022; 20 1:9.

31. El Helali N, Habibi F, Azria E, Giovangrandi Y, Autret F, Durand-Zaleski I, et al. Point-of-Care Intrapartum Group B Streptococcus Molecular Screening: Effectiveness and Costs. *Obstetrics & Gynecology* 2019; 133 2:276-81.

32. Darlow BA, Voss L, Lennon DR, Grimwood K. Early-onset neonatal group B streptococcus sepsis following national risk-based prevention guidelines. *Aust N Z J Obstet Gynaecol* 2016; 56 1:69-74.

33. Horvath B, Grasselly M, Bodecs T, Boncz I, Bodis J. Screening pregnant women for group B streptococcus infection between 30 and 32 weeks of pregnancy in a population at high risk for premature birth. *Int J Gynaecol Obstet* 2013; 122 1:9-12.

34. Poulain P, Betremieux P, Donnio PY, Proudhon JF, Karege G, Giraud JR. Selective intrapartum anti-bioprophylaxy of group B streptococci infection of neonates: a prospective study in 2454 subsequent deliveries. *Eur J Obstet Gynecol Reprod Biol* 1997; 72 2:137-40.

35. Trijbels-Smeulders MA, Kimpen JL, Kollee LA, Bakkers J, Melchers W, Spanjaard L, et al. Serotypes, genotypes, and antibiotic susceptibility profiles of group B streptococci causing neonatal sepsis and meningitis before and after introduction of antibiotic prophylaxis. *Pediatr Infect Dis J* 2006; 25 10:945-8.

36. Trijbels-Smeulders M, de Jonge GA, Pasker-de Jong PC, Gerards LJ, Adriaanse AH, van Lingen RA, et al. Epidemiology of neonatal group B streptococcal disease in the Netherlands before and after introduction of guidelines for prevention. *Arch Dis Child Fetal Neonatal Ed* 2007; 92 4:F271-6.
