# Supplementary material for: Benefits and Harms of Antenatal/Intrapartum Screening for Maternal Group B Streptococcus and Use of Intrapartum Antibiotic Prophylaxis Versus Risk‐Based Protocols or No Intervention: A Rapid Review
Source: Acta Paediatr. 2026 Apr 30;115(8):1598–610. doi: 10.1111/apa.70568 (PMC13371836; doi:10.1111/apa.70568)
Supplement: Supplementary file 23 — Data S23: Maternal health outcomes. [file APA-115-1598-s015.docx]

## Supplementary materials File 23 (S23): Maternal health outcomes

### File 23.1: Chorioamnionitis: maternal health outcome

| **Review** | **Authors** | **Country** | **Is the outcome reported for separate screening groups?** | **Type of outcome** | **No policy strategy** | **Risk Strategy** | **Screening / Universal strategy** | **Other strategy** | **If outcome data not reported separately, provide details here** | **Is the outcome reported at the level of the neonate / infant / child or maternal.** | **Other details about time frames** | **Is the outcome reported at short-term, medium-term or longer term?** | **Comments** |
| --- | --- | --- | --- | --- | --- | --- | --- | --- | --- | --- | --- | --- | --- |
| Panneflek 2024 | Al Luhidan 2019 | Saudi Arabia | No | Maternal health outcome: infection e.g. UTI and chorioamnionitis |  |  |  |  | "no documented cases of chorioamnionitis" | Maternal |  | Short-term outcome |  |
| Li 2020 | Bizzarro 2005 | USA | No | Maternal health outcome: infection e.g. UTI and chorioamnionitis |  |  |  |  | "This group included 105 inborn neonates with mean gestational age of 34 (6) weeks and birth weight of 2388 (1257) g. The median onset of infection was 1 day (range: 1– 4 days). GBS was the predominant organism cultured (47%), followed by E coli (23%), Staphylococcus species (13%), and aerobic Gram-negative rods other than E coli (8%). The maternal antenatal histories of these patients were reviewed in an attempt to identify possible risk factors for EOS. Forty-three percent of mothers had preterm labor, 26% had fever before delivery, 46% had prolonged rupture of membranes, and 20% had documented or suspected chorioamnionitis" | Maternal |  | Short-term outcome | Focus of this paper is to identify neonatal sepsis, so the data reported are not limited to GBS. Late onset sepsis and ulitra-late onset sepsis is reported by not by screening strategy. Group B strep identified in 53 cases (0-4 days when cultured), 12 cases (5-30 days when cultured), 7 cases (when > 30 days age when cultured) and 14 cases (in transported neonates). |
| Panneflek 2024 | Davis 2001 | USA | Yes | Maternal health outcome: infection e.g. UTI and chorioamnionitis |  |  | Maternal peripartum infection incidence (%): Amnionitis: 44/2438 (1.8%) | Maternal peripartum infection incidence (%): Amnionitis: 26/1337 (1.9%) |  | Maternal |  | Unclear/Not reported | Data extracted from Panneflek 2024 |
| Li 2020, Hasperhoven 2020, Panneflek 2024 | Gilson 2000 | USA | Yes | Maternal health outcome: infection e.g. UTI and chorioamnionitis |  | Maternal peripartum infection incidence (%): Chorioamnionitis: 23/407 (5.7%) | Maternal peripartum infection incidence (%): Chorioamnionitis: 28/420 (6.7%) |  |  | Maternal |  | Short-term outcome | Data extracted from Panneflek 2024. Gilson 2000 defined intrapartum chorioamnionitis as a temperature > or equal to 38 degrees in labour accompanied by at least one of the following: maternal or foetal tachycardia, uterine tenderness or malodorous amniorrhoea. They report "no significant difference between the groups [screened vs unscreened] in the incidence of chorioamnionitis or endometritis, or prevalence of STI (chlamydia, gonorrhoea, syphhilis)". Gilson 2020 also points out that this is the "first study to compare a cohort of patients managed under the two protocols concomitantly" |
| Panneflek 2024 | Katz 1999 | USA | Yes | Maternal health outcome: infection e.g. UTI and chorioamnionitis | Maternal peripartum infection incidence (%): Chorioamnionitis: 578/15620 (3.7%)* |  |  | Maternal peripartum infection incidence (%): Chorioamnionitis: 271/8748 (3.1%)* |  | Maternal |  | Unclear/Not reported | Data extracted from Panneflek 2024. Katz 1999 defined chorioamnionitis as "intrapartum maternal temperature 37.8 ° C and two or more of the following conditions: maternal tachycardia 100 beats per minute, fetal tachycardia 160 beats per minute, uterine tenderness, foul odor of the amniotic fluid, or maternal leukocytosis 15,000 cells/mm3". LOGBS definition: "All other positive GBS cultures arose in infants with late-onset GBS sepsis, with cultures positive on the 11th to 47th days of life and were not considered further in this study" |
| Li 2020, Panneflek 2024 | Locksmith 1999 (i) | USA | Yes | Maternal health outcome: infection e.g. UTI and chorioamnionitis |  |  | Maternal peripartum infection incidence (%): Chorioamnionitis: 233/4453 (5.2%)* | Maternal peripartum infection incidence (%): Chorioamnionitis: 575/7810 (7.4%)* |  | Maternal |  | Short-term outcome | Data extracted from Panneflek 2024. During the study period, women in labour were managed under three different GBS prevention strategies: Aug 1991–Aug 1993: Selective screening for women with preterm labor or preterm premature rupture of membranes; IAP was given only if both a risk factor and a positive culture were present. Sept 1993–June 1996: ACOG-guided approach; IAP was offered to women with risk factors and unknown colonization status. Cultures continued for preterm cases. From July 1996 onward: Universal screening policy endorsed by the CDC was adopted. |
| Li 2020, Panneflek 2024 | Locksmith et al., 1999 (ii) | USA | Yes | Maternal health outcome: infection e.g. UTI and chorioamnionitis |  |  | Maternal peripartum infection incidence (%): Chorioamnionitis: 233/4453 (5.2%)* | Maternal peripartum infection incidence (%): Chorioamnionitis: 599/7917 (7.7%)* |  | Maternal |  | Short-term outcome | Data extracted from Panneflek 2024. During the study period, women in labour were managed under three different GBS prevention strategies: Aug 1991–Aug 1993: Selective screening for women with preterm labor or preterm premature rupture of membranes; IAP was given only if both a risk factor and a positive culture were present. Sept 1993–June 1996: ACOG-guided approach; IAP was offered to women with risk factors and unknown colonization status. Cultures continued for preterm cases. From July 1996 onward: Universal screening policy endorsed by the CDC was adopted. |
| Panneflek 2024 | Lu 2022 | Taiwan | No | Maternal health outcome: infection e.g. UTI and chorioamnionitis |  |  |  |  | Maternal clinical chorioamnionitis: Yes in 2/23 women with GBS neonates |  |  |  |  |
| Hasperhoven 2020, Panneflek 2024 | Phares 2008 | USA | No | Maternal health outcome: infection e.g. UTI and chorioamnionitis |  |  |  |  | A total of 409 invasive group B streptococcal infections occurred in pregnant teenagers and women (0.12 per 1000 live births; yearly range: 0.11- 0.14 per 1000 live births) (Figure 2). The median age at onset was 28 years (first and third quartiles, 22 and 33 years). Half (203/409) of these cases were associated with infection of the upper genital tract, placenta, or amniotic sac, resulting in fetal death. Other manifestations included bacteremia without focus (31%), endometritis without fetal death (8%), chorioamnionitis without fetal death (4%), pneumonia (2%), and puerperal sepsis (2%). Endocarditis was observed in 1 case | Maternal | Pregnancy Associated Disease | Short-term outcome | Data not reported for separate screening strategies. |
| Li 2020, Panneflek 2024 | Puopolo 2010 | USA | No | Maternal health outcome: infection e.g. UTI and chorioamnionitis |  |  |  |  | Maternal complications for Chorioamnionitis were reported: n=103 cases however this data is presented for cases of ampicillin-resistant (n=36) or ampicillin-sensitive infections (n=67). Data not reported separately for screening strategy. | Maternal |  | Short-term outcome | 1990 –1992 (no prophylaxis); 1993–1996 (risk-based); and 1997–2007 (screening-based) |
| Panneflek 2024 | Renner 2006 | Switzerland | Yes | Maternal health outcome: infection e.g. UTI and chorioamnionitis | 7 cases/16 |  |  | 0/5 | Signs of amnionitis. Authors report no significant difference between groups (p=0.12) | Maternal |  | Short-term outcome |  |
| Panneflek 2024 | Renner 2006 | Switzerland | Yes | Maternal health outcome: infection e.g. UTI and chorioamnionitis | 7 cases/16 |  |  | 0/5 | Post-partum infection. Authors report no significant difference between groups (p=0.12) | Maternal |  | Short-term outcome |  |
| Panneflek 2024 | Share 2001 | USA | Yes | Maternal health outcome: infection e.g. UTI and chorioamnionitis | 43/576 women with chorioamnionitis |  |  | 27/1135 women with chorioamnionitis | Chorioamnionitis was reported as significantly different between groups (p<0.001) | Maternal | Not clearly reported | Short-term outcome | Data extracted from Table 2. Maternal risk factors. "Clinical chorioamnionitis was identified from progress and delivery notes written by the obstetrical team including evidence of fever, uterine tenderness, and fetal distress/tachycardia" |
| Panneflek 2024 | Uy 2002 | USA | Yes | Maternal health outcome: infection e.g. UTI and chorioamnionitis | Maternal peripartum infection incidence (%): Chorioamnionitis: 10/300 (3.3%) | Maternal peripartum infection incidence (%): Chorioamnionitis: 6/150 (4.0%) |  | Maternal peripartum infection incidence (%): Chorioamnionitis: 4/150 (2.7%) |  | Maternal |  | Short-term outcome | Data extracted from Panneflek 2024. The authors report that there "was no change in the rate of treatment of chorioamnionitis in surveyed women over the 14-year period" |
| Panneflek 2024 | van Dyke 2009 | USA | No | Maternal health outcome: infection e.g. UTI and chorioamnionitis |  |  |  |  | Suspected chorioamnionitis % (95% CI): 3.1 (2.6-3.7) | Maternal |  | Short-term outcome | From the Obstetrical characteristics in Characteristics of the Cohort from 10 Active Bacterial Core Surveillance Sites, 2003–2004 |
| Li 2020, Hasperhoven 2020, Panneflek 2024 | Vergani 2002 | Italy | Yes | Maternal health outcome: infection e.g. UTI and chorioamnionitis |  |  | Chorioamnionitis was reported in 93/7602 women during the universal screening with cultures at 26-28 weeks. Chorioamnionitis was reported in 59/6152 women during the universal screening with cultures at 35-37 weeks. Authors report no significant difference between these two groups. |  |  | Maternal |  | Short-term outcome |  |

**Abbreviations**: EOGBS: Early-Onset Group B Streptococcal Disease, GBS: Group B Streptococcal Disease, IAP: intrapartum antibiotics prophylaxis, UTI: urinary tract infection, USA: United States of America

### File 25.2: Anaphylaxis: maternal health outcome

| **Review** | **Authors** | **Country** | **Is the outcome reported for separate screening groups?** | **Type of outcome** | **No policy strategy** | **Risk Strategy** | **Screening / Universal strategy** | **Other strategy** | **If outcome data not reported separately, provide details here** | **Is the outcome reported at the level of the neonate / infant / child or maternal.** | **Other details about time frames** | **Is the outcome reported at short-term, medium-term or longer term?** | **Comments** |
| --- | --- | --- | --- | --- | --- | --- | --- | --- | --- | --- | --- | --- | --- |
| Li 2020, Hasperhoven 2020, Panneflek 2024 | Angstetra 2007 | Australia | No | Maternal health outcome: anaphylaxis |  |  |  |  | "It is important to remember that antibiotic use in labour is not without risk. There is the potential of a fatal anaphylaxis event (~0.001% of all treated women) and an increase risk of non-fatal anaphylaxis. We have not had any cases of anaphylactic reaction." | Maternal |  | Short-term outcome | From discussion section. |
| Panneflek 2024 | Davis 2001 | USA | Yes | Maternal health outcome: anaphylaxis |  |  | Postpartum: Anaphylaxis, pseudomembranous colitis: 0/2438 | Postpartum: Anaphylaxis, pseudomembranous colitis: 0/1337 | Postpartum: Anaphylaxis, pseudomembranous colitis: | Maternal |  |  |  |
| Panneflek 2024 | Jeffery 1998 | Australia | Yes | Maternal health outcome: anaphylaxis | Maternal peripartum infection incidence (%): Anaphylaxis: 0/5732 (0%) |  | Maternal peripartum infection incidence (%): Anaphylaxis: 1/36342 (0%) |  |  | Maternal |  | Unclear/Not reported | Data extracted from Panneflek 2024. According to Jeffery & Moses Lahra 1998, the "background rate of EOGBSD was determined prospectively over a 16-month period (November 1986 to February 1988) before the intervention, which was introduced as protocol in March 1988. After 3 months of in-service education for the staff, data were collected prospectively on all infants with EOGBSD from June 1988" |
| Panneflek 2024 | Jeffery 1998 | Australia | Unclear | Maternal health outcome: anaphylaxis |  |  |  |  | "Maternal Morbidity. There was one severe, maternal allergic reaction to ampicillin. This one mother (of 3400 treated carriers) had no history to suggest sensitization and was treated effectively with steroids and antihistamines but did not require epinephrine. There was no fetal distress, and her term baby was well at delivery and successfully breastfed from the first postnatal day." | Maternal |  | Short-term outcome |  |
| Panneflek 2024 | Katz 1994 | USA | Unclear | Maternal health outcome: anaphylaxis |  |  |  |  | "Additionally, there were no adverse antibiotic reactions among treated carriers: no cases of pseuomembranous colitis, antibiotic diarrhoea or allergic reactions". | Maternal |  | Short-term outcome | Narrative description of text and while some details are reported the time-frames for some of the outcomes are unclear. |
| Li 2020, Panneflek 2024 | Locksmith 1999 | USA | No | Maternal health outcome: anaphylaxis |  |  |  |  | No cases of anaphylactic reaction to the prophylactic antibiotics were encountered during the series. | Unclear / not specified |  | Short-term outcome |  |
| Hasperhoven 2020, Li 2020, Panneflek 2024 | Main 2000 | USA | No | Maternal health outcome: anaphylaxis |  |  |  |  | Comment provided in the discussion with reviewers - see Editors note "Incidences of rashes and other adverse effects. We have not had any cases of anaphylactic reaction. Because most patients only receive one to three doses of ampicillin during labor, we have not had significant rashes during labor." | Maternal |  | Short-term outcome | There is an additional case note review of 1000 women during the culture-based period only. No data has been extracted from this as it only represents 3 months and is limited to one screening strategy (see Table V in paper). |
| Li 2020 | Reisner 2000 | USA | Yes | Maternal health outcome: anaphylaxis | NR |  | 0 |  | There were no cases of maternal anaphylaxis during this 36-month period. | Maternal |  | Short-term outcome |  |
| Panneflek 2024 | van Dyke 2009 | USA | No | Maternal health outcome: anaphylaxis |  |  |  |  | Report allergy to penicillin % (95% CI). With low risk of anaphylaxis: 8.1 (7.4–8.9) and with high risk of anaphylaxis: 1.0 (0.7–1.3). However the authors state that "There were no verified episodes of anaphylaxis after chemoprophylaxis" | Maternal |  | Short-term outcome | From the Prenatal care and medical history in Characteristics of the Cohort from 10 Active Bacterial Core Surveillance Sites, 2003–2004 |
| Li 2020, Hasperhoven 2020, Panneflek 2024 | Vergani 2002 | Italy | No | Maternal health outcome: anaphylaxis |  |  |  |  | "There was no occurrence of anaphylactic reaction or major dermatologic reactions due to intrapartum maternal antibiotic prophylaxis during the study" | Maternal |  | Short-term outcome |  |
| Panneflek 2024 | Jeffery 1998 | Australia | Yes | Maternal health outcome: anaphylaxis | Maternal peripartum infection incidence (%): Anaphylaxis: 0/5732 (0%) |  | Maternal peripartum infection incidence (%): Anaphylaxis: 1/36342 (0%) |  |  | Maternal |  | Unclear/Not reported | Data extracted from Panneflek 2024. According to Jeffery 1998, the "background rate of EOGBSD was determined prospectively over a 16-month period (November 1986 to February 1988) before the intervention, which was introduced as protocol in March 1988. After 3 months of in-service education for the staff, data were collected prospectively on all infants with EOGBSD from June 1988" |

**Abbreviations**: EOGBS: Early-Onset Group B Streptococcal Disease, GBS: Group B Streptococcal Disease, IAP: intrapartum antibiotics prophylaxis, UTI: urinary tract infection, USA: United States of America

### File 25.3: Adverse events: maternal health outcome

| **Review** | **Authors** | **Country** | **Is the outcome reported for separate screening groups?** | **Type of outcome** | **No policy strategy** | **Risk Strategy** | **Screening / Universal strategy** | **Other strategy** | **If outcome data not reported separately, provide details here** | **Is the outcome reported at the level of the neonate / infant / child or maternal.** | **Other details about time frames** | **Is the outcome reported at short-term, medium-term or longer term?** | **Comments** |
| --- | --- | --- | --- | --- | --- | --- | --- | --- | --- | --- | --- | --- | --- |
| Newly identified | Daniels 2022 | England, UK | No | Other (please specify) |  |  |  |  | No serious adverse events were reported. | Other (please specify) |  |  | Outcomes included SAE in the mother or newborn. None reported. |
| Panneflek 2024+A55:F55 | Davis 2001 | USA | Yes | Other (please specify) |  |  | Postpartum rash: 7/2438 | Postpartum rash: 3/1337 |  | Maternal |  | Short-term outcome | The rate of rash varied from 0% to 0.29% and did not appear to increase after guideline implementation |
| Panneflek 2024 | Gibbs 1994 | USA | No | Other (please specify) |  |  |  |  | "There were no adverse effects of prophylatic antibiotic administration on the mothers or infants" | Other (please specify) | Mother and neonate | Short-term outcome |  |
| Li 2020, Hasperhoven 2020, Panneflek 2024 | Gilson 2000 | USA | No | Other (please specify) |  |  |  |  | No cases of adverse reactions to antibiotics were noted | Unclear / not specified |  | Short-term outcome |  |
| Li 2020, Hasperhoven 2020, Panneflek 2024 | Gopal Rao 2017 | UK | No | Other (please specify) |  |  |  |  | "We were not aware of any women developing adverse reactions to IAP through our hospital’s adverse event reporting system (Datix) or through departmental reporting systems in the screening and postscreening periods. We did not collect information regarding adverse reaction to IAP in the prescreening period." | Maternal |  | Short-term outcome |  |
| Li 2020, Panneflek 2024 | Locksmith 1999 | USA | No | Maternal health outcome: anaphylaxis |  |  |  |  | No cases of anaphylactic reaction to the prophylactic antibiotics were encountered during the series. | Unclear / not specified |  | Short-term outcome |  |
| Panneflek 2024 | Poulain 1997 | France | No | Other (please specify) |  |  |  |  | The chemoprophylaxis was really done in only 12 of the 19 (63%). When ampicillin was not administrated, this was due to protocol violation or because the obstetrical risk of transmission was noted nearly the time of delivery. There was no adverse effect of amoxicillin. | Maternal |  | Short-term outcome |  |
| Li 2020 | Reisner 2000 | USA | Yes | Other (please specify) | NR |  | 0 |  | There were no maternal complications attributable to prophylactic intravenous antibiotic use. There were no cases of Clostridium difficile infection among patients receiving group B streptococcal prophylaxis. Several minor rashes were treated with antihistamines. | Maternal |  | Short-term outcome |  |
| Newly identified | Riley 2003 * | USA | No | Other (please specify) |  |  |  |  | "There was one adverse drug reaction, which was a rash to penicillin. | Unclear / not specified |  | Other | "At the academic hospital using the risk-based strategy, there is one physician group and one midwifery group providing care to the entire population. At the community hospital, women were counted in the culture- or risk-based strategy based on the intended strategy for their provider practice" |

**Abbreviations**: EOGBS: Early-Onset Group B Streptococcal Disease, GBS: Group B Streptococcal Disease, IAP: intrapartum antibiotics prophylaxis, UTI: urinary tract infection, USA: United States of America

*Excluded from Panneflek 2024 as no outcomes of interest reported

### File 25.4: Other: maternal health outcome

| **Review** | **Authors** | **Country** | **Is the outcome reported for separate screening groups?** | **Type of outcome** | **No policy strategy** | **Risk Strategy** | **Screening / Universal strategy** | **Other strategy** | **If outcome data not reported separately, provide details here** | **Is the outcome reported at the level of the neonate / infant / child or maternal.** | **Other details about time frames** | **Is the outcome reported at short-term, medium-term or longer term?** | **Comments** |
| --- | --- | --- | --- | --- | --- | --- | --- | --- | --- | --- | --- | --- | --- |
| Panneflek 2024 | Davis 2001 | USA | Yes | Maternal health outcome: infection e.g. UTI and chorioamnionitis |  |  | Maternal peripartum infection incidence (%) : Endometritis: 14/2438 (0.6%)* *p<0.05 | Maternal peripartum infection incidence (%) : Endometritis: 23/1337 (1.7%)* *p<0.05 |  | Maternal |  | Unclear/Not reported | Data extracted from Panneflek 2024. According to Davis 2001 "Although the proportion of women with clinically diagnosed amnionitis remained constant across time, the rate of endometritis fell after guideline implementation " |
| Panneflek 2024 | Davis 2001 | USA | Yes | Maternal health outcome: infection e.g. UTI and chorioamnionitis |  |  | Maternal peripartum infection incidence (%) : Sepsis/bacteraemia: 0/2438 (0%)** **p<0.07 | Maternal peripartum infection incidence (%) : Sepsis/bacteraemia: 2/13837 (0.2%)** **p<0.07 |  | Maternal |  | Unclear/Not reported | Data extracted from Panneflek 2024. According to Davis 2001 "The proportion of women with either sepsis or bacteremia did not change" |
| Li 2020, Hasperhoven 2020, Panneflek 2024 | Gilson 2000 | USA | Yes | Maternal health outcome: infection e.g. UTI and chorioamnionitis |  | Maternal peripartum infection incidence (%) Endometritis:15/407 (3.9%) | Maternal peripartum infection incidence (%): Endometritis: 11/420 (2.6%) |  |  | Maternal |  | Short-term outcome | Data extracted from Panneflek 2024. Gilson 2000 defined postpartum endometritis as a temperature > or = to 38 degrees C during the first 72 hours postpartum accompanied by uterine ternderness and/or malodorous lochia. Gilson reports "no significant difference betwen the groups [screened vs unscreened] in the incidence of chorioamnionitis or endometritis, or prevalence of STI (chlamydia, gonorrhoea, syphhilis)". Gilson 2020 also points out that this is the "first study to compare a cohort of patients managed under the two protocols concomitantly" |
| Li 2020, Panneflek 2024 | Locksmith 1999 (i) | USA | Yes | Maternal health outcome: infection e.g. UTI and chorioamnionitis |  |  | Maternal peripartum infection incidence (%): Endometritis: 123/4453 (2.8%)* | Maternal peripartum infection incidence (%): Endometritis: 314/7810 (4.0%)* |  | Maternal |  | Short-term outcome | Data extracted from Panneflek 2024. During the study period, women in labour were managed under three different GBS prevention strategies: Aug 1991–Aug 1993: Selective screening for women with preterm labor or preterm premature rupture of membranes; IAP was given only if both a risk factor and a positive culture were present. Sept 1993–June 1996: ACOG-guided approach; IAP was offered to women with risk factors and unknown colonization status. Cultures continued for preterm cases. From July 1996 onward: Universal screening policy endorsed by the CDC was adopted. |
| Hasperhoven 2020, Panneflek 2024 | Phares 2008 | USA | No | Maternal health outcome: infection e.g. UTI and chorioamnionitis |  |  |  |  | One individual with pregnancy-associated disease died. | Maternal | Pregnancy-Associated Disease | Short-term outcome | Data not reported for separate screening strategies. |
| Hasperhoven 2020, Panneflek 2024 | Phares 2008 | USA | No | Maternal health outcome: infection e.g. UTI and chorioamnionitis |  |  |  |  | A total of 409 invasive group B streptococcal infections occurred in pregnant teenagers and women (0.12 per 1000 live births; yearly range: 0.11- 0.14 per 1000 live births) (Figure 2). The median age at onset was 28 years (first and third quartiles, 22 and 33 years). Half (203/409) of these cases were associated with infection of the upper genital tract, placenta, or amniotic sac, resulting in fetal death. Other manifestations included bacteremia without focus (31%), endometritis without fetal death (8%), chorioamnionitis without fetal death (4%), pneumonia (2%), and puerperal sepsis (2%). Endocarditis was observed in 1 case | Maternal | Pregnancy-Associated Disease | Short-term outcome | Data not reported for separate screening strategies. |
| Panneflek 2024 | Share 2001 | USA | Yes | Maternal health outcome: infection e.g. UTI and chorioamnionitis | 70/576 women with UTI |  |  | 116/1135 women with UTI | Urinary tract infection was reported as significantly different between groups (p<0.001). Note this is reported as maternal urinary tract infection *during pregnancy* | Maternal | Maternal urinary tract infection during pregnancy - timing of UTI unclear | Short-term outcome | Data extracted from Table 2. Maternal risk factors |

**Abbreviations**: EOGBS: Early-Onset Group B Streptococcal Disease, GBS: Group B Streptococcal Disease, IAP: intrapartum antibiotics prophylaxis, UTI: urinary tract infection, USA: United States of America
