# Supplementary material for: Benefits and Harms of Antenatal/Intrapartum Screening for Maternal Group B Streptococcus and Use of Intrapartum Antibiotic Prophylaxis Versus Risk‐Based Protocols or No Intervention: A Rapid Review
Source: Acta Paediatr. 2026 Apr 30;115(8):1598–610. doi: 10.1111/apa.70568 (PMC13371836; doi:10.1111/apa.70568)
Supplement: Supplementary file 24 — Data S24: Harms and benefits. [file APA-115-1598-s024.docx]

## Supplementary File 24 (S24). Harms and benefits reported in primary studies

Abbreviations: **NS O:** no screening policy vs other; **NS R**: no screening policy vs risk-based approach; **NS RO:** no screening vs risk-based vs other; **NS S**: no screening policy vs screening; **NS SO**: no screening policy vs screening vs other; **NS SR**: no screening policy vs screening vs risk-based policy; **O**: other; **RO:** risk-based approach vs other; **ROO**: risk-based policy vs other vs another other approach; **SO:** screening vs other approach; **SOO**: screening vs other vs another other approach **SR**: screening vs risk-based approach; **SRO**: screening vs risk-based policy vs other; **SS:** one screening approach vs another type of screening approach

| **Authors** | **Country** | **Type of screening approach comparisons** | **Harms or benefits of screening?** | **Is the harm or benefit reported at the level of the neonate/ infant/ child or mother** | **Select a category that closely matches the harm or benefit.** | **Specify details of the harm or benefit here (verbatim quote)** |
| --- | --- | --- | --- | --- | --- | --- |
| Abdelmaaboud & Mohammed 2011 (1) | Qatar | SR | Harm | Potentially both neonate/maternal | Harm false negatives | In the present study, some cases of EOGBS disease reported in term neonates delivered to mothers with negative GBS-screening culture results. In addition to the use of effective IAP, the negative prenatal GBS-screening results may have contributed to intra- partum management in a way that increased the risk of sepsis in neonates. It is possible that the negative GBS screens provided a false sense of reassurance to obstetrical providers. If the mothers in these cases had an unknown GBS status, it is likely that antibiotics for IAP in the presence of risk factors, or for treatment for chorioamnionitis, would have been given and perhaps prevented some of the cases of EOGBS disease. Whether these negative cultures were false-negative results or the mothers acquired GBS in the interval between the screening culture and the time of delivery is unknown. |
| Abdelmaaboud & Mohammed 2011 (1) | Qatar | SR | Benefit | Neonate - not specified | Benefit more effective treatment | ‘The annual incidence’ of EOGBS disease from 2003 to 2009 slightly increased after institution of risk-based strategy but this increase was not statistically significant (p=0.28). The authors note that before the implementation of IAP in 2003 that "the overall incidence of EOGBS disease has dramatically declined from an estimated 1.9 cases per 1000 live births before 2003 to 0.51 cases per 1000 live births thereafter" |
| Abdelmaaboud & Mohammed 2011 (1) | Qatar | SR | Neutral | Neonate - not specified | Neutral | The overall incidence of EOGBS disease in total live births and VLBW neonates during institution of the two preventive strategies revealed no obvious change ("there is a statistically significant difference in ‘the overall incidence’ of EOGBS disease between total live births and VLBW (p=0.001). The overall incidence in total live births during the period from 2003 to 2006 (universal screening approach) was 0.53 cases per 1000 live births and among VLBW infants was 4.27 cases per 1000 VLBW births. From 2006 to 2009 (a risk-based approach), the overall incidence was 0.57 cases per 1000 live births and 4.87 cases per 1000 VLBW births") |
| Al Luhidan et al 2019 (2) | Saudi Arabia | O | Harm | Neonate - not specified | Benefit more effective treatment | "The incidence during the period when the universal screening was applied (from 2004 to 2014) was 0.29/1000. By the end of the year 2014, after universal screening was discontinued and risk-based approach was implemented, the incidence of GBS disease increased significantly to 1.8 for the years 2015 and 2016 (P-value <0.0001)." The authors note the limitations of the study design stating: "This study was done retrospectively, and therefore we had no means of insuring the adherence to the risk-based approach by obstetricians and gynecologists, it was also not originally designed to assess the efficacy of the GBS screening. Consequently, this study cannot directly compare the risk-based approach to universal screening, but the numbers are concerning and indicate that further evaluation of the 2 approaches is warranted." |
| Alarcon et al 2004 (3) | Spain | NS SR | Harm | Neonate - not specified | Harm health outcomes of exposure to antibiotics during labour | Authors note that "ampicillin resistant E. coli infections occurred with increasing frequency among preterm infants, suggesting that differences in exposure to maternal antibiotics before delivery between preterm and term neonates may underline this tendency". They note that the incidence of E Coli remained stable across the study period but that antibiotic-antibiotic-resistant E. coli infections increased among preterm infants. They conclude that evaluation of the risks and benefits of prophylaxis in pre-terms is "critical" |
| Angstetra et al 2007 (4) | Australia | RO | Benefit | Neonate - not specified | Benefit more effective treatment | Authors state "The incidence of early-onset GBS disease fell during the screening-based protocol period to 0.00 case per 1000 live births from the baseline prescreening period incidence of 0.84 cases per 1000 live births (χ2 with Yates = 5.75; P=0.016). There was no difference between the incidences of non-GBS disease during prescreening period of 0.94 cases per 1000 live births and screening period of 0.72 cases per 1000 live births (χ2 with Yates = 0.14; P= 0.71).". They state that "In our institution, the use of antibiotics to provide prophylaxis against GBS has significantly decreased the incidence of early-onset GBS disease without increasing the incidence of non-GBS disease." |
| Bauserman et al 2013 (5) | USA | O | Benefit | Neonate - not specified | Benefit more effective treatment | "The incidence of GBS early-onset SBI from 1997–2001 to 2002–2010 decreased from 3.5 to 2.6 per 1000 admissions (Figure 1). On multivariable regression, the odds of developing GBS early-onset SBI were lower (odds ratio [OR]=0.69; 95% confidence interval [CI]: 0.59, 0.80]; P<0.001) in the later time period" |
| Bauserman et al 2013 (5) | USA | O | Harm | Neonate - not specified | Harm Other (please specify) | Over the same time period, the incidence of GBS late-onset SBI increased from 0.8 to 1.1 per 1000 admissions, and incidence of E. coli late-onset SBI increased from 2.2 to 2.5 per 1000 admissions. |
| Bekker et al 2014 (6) | The Netherlands | O | Harm | Neonate - not specified | Benefit more effective treatment | "We showed a 60% increase in the incidence of infant invasive group B streptococcus infection in the Netherlands over the past 25 years despite the introduction in 1999 of guidelines for prevention of neonatal group B streptococcus disease... However, we present evidence that, by contrast with what was anticipated, the incidence of invasive group B streptococcus infection has further increased with the same trend as before the introduction of the guidelines. This increase was mainly a result of a rise in the number of cases caused by group B streptococcus belonging to clonal complex 17." |
| Bizzarro 2005 (7) | USA | O | No harms or benefits reported |  |  |  |
| Björklund et al 2017 (8) | Finland | SR | Benefit | Neonate - not specified | Benefit worthwhile use of resources | "In newborns with suspected infection, the introduction of the rapid test was related to a decreased length of stay on the pediatric care unit by 1.16 days (p= 0.01), and an increase in the length of stay on the mother-and-baby ward by 1.11 days (p<0.001). No increase in antibiotics was noted"..."In conclusion, the availability of an accurate point of care test for GBS is important to improve the accuracy of GBS colonization detection and prevention of intrapartum trans- mission. It could reduce patients’ length of stay, without an increase in antibiotic use" |
| Björnsdóttir et al 2019 (9) | Iceland | NS R | Benefit | Neonate - not specified | Benefit more effective treatment | "The risk-based chemoprophylaxis adopted in Iceland possibly contributed to the decline of EOD but has had limited effect on LOD." |
| Brozanski et al 2000 (10) | USA | NS SR | Unclear | Neonate - not specified | Neutral | "Of the 2174 women who qualified for prophylaxis, 1871 received some form of it, for an overall compliance rate of 86.1%. Approximately 94% of women who met CDC criteria for prophylaxis and who delivered vaginally received some prophylaxis, and 1137 (68.9%) of those women received it at least 4 hours before delivery. The most common reason for delay in or lack of administration of prophylaxis for qualified women was late presentation. Of 193 who delivered by cesarean and met CDC criteria for prophylaxis but received none, 95% were either previously scheduled or emergent deliveries without onset of labor. Prophylaxis was given to 39.9% (126) of women without risk factors in whom colonization was unknown. |
| Brozanski et al 2000 (10) | USA | NS SR | Harm | Potentially both neonate/maternal | Harm Other (please specify) | Almost 40% of women in our study in whom colonization status was unknown, and who did not meet CDC criteria for prophylaxis, received intrapartum antibiotics. In some cases, rupture of membranes had occurred in the preceding 12 hours and antibiotics might have been administered in anticipation of the risk factor of rupture of membranes at or more than 18 hours before delivery. In other instances, cultures were documented as sent from office visits, but results were not noted on hospital medical records and were recorded as unknown in our data. Consequently, we might have overestimated the number of women whose colonization status was unknown. |
| Chan et al 2023 (11) | Hong Kong, PRC | SR | Benefit | Neonate - not specified | Benefit more effective treatment | "The incidence of EOGBSD dropped from 1.03 per 1000 live births (95% CI 0.87–­ 1.22) in 2009–­ 2011, to 0.26 per 1000 live births (95% CI 0.64–0.92) in 2012–­ 2020 (p < 0.05). The incidence of EOGBSD was higher than that of E. coli sepsis before 2012, but be- came persistently lower than that of E. coli after 2012" |
| Chen 2001 (12) | USA | NS R | Benefit | Neonate - not specified | Benefit Other (please specify) | "Given the concern for the emergence of EONS caused by ampicillin-resistant organisms in a period with increased antibiotic use, we investigated the rate of EONS caused by ampicillin-resistant organisms over 2 periods and found no increase" |
| Chen 2005 (13) | USA | NS SR | No harms or benefits reported |  |  |  |
| Cho et al 2019 (14) | Taiwan | O | Benefit | Neonate - not specified | Benefit more effective treatment | "The overall neonatal invasive GBS infection rate was 0.81 per 1000 live births and the vertical transmission rate was 1.2%. After 2012, the invasive neonatal GBS infection rate declined from 1.1-1.6% to 0.6-0.7% in 2014 and thereafter, the GBS EOD incidence rate declined from 2.8% to 0.0-0.6%, but the LOD incidence rate remained approximately 0.7%. Infants with EOD had strong association with obstetric risk factors. Taiwan’s universal GBS screening with IAP program reduced the incidence rate of neonatal GBS EOD to be lower than 1% after 2012. Pediatricians still should pay attention to infants with GBS LOD since its incidence rate remained unchanged." |
| Clemens & Gable 2002 (15) | USA | NS S | Benefit | Neonate - not specified | Benefit Other (please specify) | "Over 90% of mothers at WHG who were colonized with GBS or whose GBS status was unknown received intrapartum antibiotic prophylaxis. Additionally, since the adoption of the CDC guidelines at WHG, the incidence of culture proven EOGBS disease declined at WHG from 1 to 2/1000 before 1996 to less than 0.5 /1000 after 1996" The authors note that implementation of the protocol was in a community hospital and not affiliated with an academic centre. They attribute the "success of our program hinged on a number of factors. First, the policy was developed with the input and ‘‘buy - in’’ of the entire community. A strong and respected physician leader served as the catalyst. Presentation of the CDC guidelines as well as a formal educational seminar followed. Second, a preprinted order for antibiotic treatment of all qualifying women was created. Previous studies have shown that preprinted orders increase compliance rates of interventions such as pneumococcal and influenza vaccine to the institutionalized elderly,9,10 or screening programs such as evaluating the frequency of hepatitis B surface antigen in pregnant women.11 Third, placement of the patient’s GBS status on an electronically scannable clinical pathway allowed excellent quality control of GBS status collection. |
| Coco 2002 (16) | USA | SR | Harm | Potentially both neonate/maternal | Harm Other (please specify) | The major difference between the groups was in the number of eligible women who received no doses of antibiotics. Women in the screening group (8 patients) were more likely to receive no doses of antibiotics when compared with the risk factor group (only 1 patient). This was primarily due to lack of time; of the 8 women, 6 gave birth rapidly with an average time of 43 minutes after admission. The other 2 women in this subset were protocol violations; the providers were unaware of the women’s group B streptococcal culture results even though the results were in the medical records. A large percentage of women in both groups received only one dose of antibiotics. Again, lack of time was the reason. In both groups, labor progressed too quickly to administer a second dose of antibiotics for the 40 women involved in this subset (23 in the screening group and 17 in the risk factor group). |
| Coco 2002 (16) | USA | SR | Unclear | Neonate - not specified | Harm Other (please specify) | There was an increased odds of newborns having a complete blood count ordered using the screening strategy (OR 1.35, 95%; CI 1.01, 1.80). The percentages of newborns having blood cultures drawn, of newborns receiving antibiotics, and of mothers receiving antibiotics were not different between the two study groups |
| Coco 2002 (16) | USA | SR | Benefit | Potentially both neonate/maternal | Benefit worthwhile use of resources | Secondary outcomes of maternal and newborn length of stay and newborn hospital charges. No differences were detected between the study groups (screening vs risk-based) |
| Daniels et al. 2022 (17) | England, UK | RO | Benefit | Potentially both neonate/maternal | Benefit Other (please specify) | "We evaluated whether, in women with clinical risk factors for early neonatal infection, the use of point-of-care rapid intrapartum test to detect maternal GBS colonisation reduces maternal antibiotic exposure compared with usual care, where antibiotics are administered due to those risk factors. There was no evidence of a difference in the rates of intrapartum antibiotic prophylaxis (relative risk 1.16, 95% CI 0.83 to 1.64) between the rapid test (41%, 297/716) and usual care (36%, 328/906) units". The authors report "In pregnant women with risk factors for early-onset GBS infection in their babies, the use of a point-of-care rapid test in labour to diagnose maternal GBS colonisation increased the administration of intrapartum antibiotics to prevent neonatal GBS infection by a small amount, compared with the usual care strategy of risk-factor based antibiotics administration, but with considerable uncertainty. The overall maternal exposure to antibiotics for any reason was not reduced with the use of the rapid test compared with usual care, whilst more women received an adequate duration of intrapartum antepartum prophylaxis in the rapid test units." |
| Daniels et al 2022 (17) | England, UK | RO | Harm | Neonate - not specified | Harm Other (please specify) | "The absence of a reduction in intrapartum antibiotic prophylaxis through the implementation of a rapid testing strategy can be attributed to the following reasons. Firstly, although all women in usual care units should have been offered antibiotics if they had risk factors for neonatal early-onset GBS infection, this was only administered to 36% of women, which was lower than our expected estimate. This highlights the low adherence to the national guidelines, a situation that has changed little since a surveillance study in 2014–2015 where 44% of women with risk factors received IAP" |
| Darlow et al 2016 (18) | New Zealand | O | Harm | Potentially both neonate/maternal | Harm Other (please specify) | In 16 cases (55%), a maternal risk factor qualifying the mother for intrapartum antibiotics was present, but only five (31%) received this intervention |
| Davis 2001 (19) | USA | RO | Benefit | Neonate - not specified | Benefit more effective treatment | "The proportion of infants undergoing evaluation decreased after implementation of the neonatal guidelines; among infants of group B streptococci–negative women, test ordering dropped by almost 40%. CONCLUSIONS: Implementation of the new guidelines is feasible and can be accomplished rapidly. The guidelines were associated with increased maternal intrapartum antibiotic use, particularly among women at highest risk, and with a decrease in laboratory use for infants" |
| Eberly & Rajnik 2009 (20) | USA | NS SR | Benefit | Neonate - not specified | Benefit more effective treatment | "Incidence fell by 63% (1.95 to 0.72) from the pre-prevention to the dual-prevention era, and then fell an additional 35% (0.72 to 0.47) from the dual-prevention to the universal-culture era. w2 analysis of trends indicates these shifts are statistically significant. Thus, universal screening appears to be the superior method for preventing EoGBS." |
| Ecker et al 2013 (21) | USA | NS SR | Benefit | Neonate - not specified | Benefit more effective treatment | "A significant decrease in the incidence of GBS infections occurred over time, with no change in the incidence of other pathogens or the emergence of antibiotic resistance, including the very low-birthweight population". |
| Edwards et al 2003 (22) | USA | SR | No harms or benefits reported |  |  |  |
| Eisenberg 2005 (23) | USA | SR | Harm | Unclear | Harm Other (please specify) | Although only 3% (775) of the women who were screened had no results available on their charts at the time of delivery, 2 babies from this small group developed early onset GBS disease, a rate of 2.58 per 1000 live births. Neither of these 2 mothers with unknown results had received antibiotics before delivery. The authors state "There was less than optimal timing of antibiotic administration in the presence of a positive GBS screening culture. This was most apparent in women who did not have additional risk factors. The rate of early onset GBS disease was low in women who had a negative screening culture; however, there were a small number of cases that could not have been prevented because of a false negative screening result or because these women acquired GBS infection after they were screened. False negative screening can result from improper culture collection technique or failure to use selective broth in the laboratory. In 3% of women screened, results of screening cultures were not available at the time of labor and delivery, and most of these women did not receive optimal intrapartum antibiotics. The importance of having screening culture reports recorded in the prenatal record and having the record at the hospital before labor cannot be overemphasized" |
| El Helali et al 2019 (24) | France | SS | Unclear |  | Neutral | "During the intrapartum PCR screening period, 14.5% of term deliveries screened positive compared with 12.2% during the antenatal culture period (P<001) and 91.8% women who were screened positive received intrapartum antibiotic prophylaxis compared with 89% during the antenatal culture period (P=0.55)" |
| El Helali et al 2019 (24) | France | SS | Harm | Neonate - not specified | Harm Other (please specify) | "During the antenatal culture period, 395 (3.8%) of term deliveries did not undergo GBS testing compared with 23 (0.1%) in the intrapartum PCR period (P<001)" |
| El Helali et al 2019 (24) | France | SS | Benefit | Neonate - not specified | Benefit more effective treatment | The number of proven (bacteraemia and meningitis) and probable early-onset GBS disease cases was 60% lower in the intrapartum PCR period, 3.8/1,000 (95% CI 2.3–7.4) compared with 0.9/1,000 (95% CI 0.6–1.5) (P<001). |
| El Helali et al 2019 (24) | France | SS | Benefit | Neonate - not specified | Benefit more effective treatment | The proportion of newborns with GBS infection in mothers who screened positive decreased from 0.86% to 0.38% (P=.043), and from 1.40% to 0.28% (P=.009) in those with unknown GBS status at delivery. |
| El Helali et al 2019 (24) | France | SS | Benefit | Neonate - not specified | Benefit Other (please specify) | More importantly, in women who screened negative, the proportion of early-onset GBS disease fell from 0.36% to 0.04% (P<001) with a 78.5% decrease in the number of early-onset GBS disease cases during the intrapartum PCR screening period compared with the antenatal culture screening period |
| El Helali et al 2019 (24) | France | SS | Benefit | Neonate - not specified | Benefit worthwhile use of resources | "The yearly cost of delivery and treatment of newborns with GBS infection was reduced from $41,875 ($6,823) to $11,945 (10,303) (P<001). The estimated extra cost to avoid one early-onset GBS disease was $5,819." Additional cost data is presented in detail in the Allan 2024 review. |
| Factor et al 1998 (25) | USA | NS R | Benefit | Neonate - not specified | Benefit more effective treatment | This study compared risk screening vs no screening. The authors report that "The incidence of neonatal group B streptococcal disease declined by 94% at the same time that antibiotic use for women at high risk increased." |
| Garland 1991 (26) | Australia | NS S | Benefit | Neonate - not specified | Benefit more effective treatment | "The implementation of maternal GBS screening at 32 weeks' ge4station results in no neonatal GBS infections of babes born to asymptomatic colonized mothers who received intrapartum penicillin. By contrast in the control group of unscreened untreated patients, there were 27 neonatal GBS infections with 8 deaths" |
| Garland 1991 (26) | Australia | NS S | Harm | Neonate - not specified | Harm Other (please specify) | "Sixteen neonatal infections were documented in the [universal] screening group and half of these were missed by virtue of being < 32 weeks gestation and therefore not identified by the screening protocol.” |
| Garland 1991 (26) | Australia | NS S | Harm | Neonate - not specified | Harm Other (please specify) | "Sixteen neonatal infections were documented in the [universal] screening group and half of these were missed by virtue of being < 32 weeks gestation and therefore not identified by the screening protocol. The remainder were due to failure to follow the protocol (5 cases) or due to the onset of maternal sepsis (3 cases)” |
| Gibbs et al 1994 (27) | USA | NS S | Harm | Potentially both neonate/maternal | Harm Other (please specify) | Authors described that implement a protocol for universal screening and selective prophylaxis …"is complex as revealed by several kinds of protocol errors, including failure to give indicated prophylaxis, failure to use selective media …and administration of prophylaxis when not indicated. It is also possible that some patients who received prenatal care at University Hospital were not screened, but we do not have these data available"....The novel contribution of our report is the practical information on compliance rates, protocol errors, and failures in an American teaching hospital outside a research setting". |
| Gilson et al 2000 (28) | USA | SR | Benefit | Neonate - not specified | Benefit more effective treatment | "Our results demonstrate a decreased incidence of early-onset neonatal GBS sepsis in the infants of the screened women receiving IAP when compared to the infants of the unscreened women treated on the basis of risk factors (p=0.04) |
| Gilson et al 2000 (28) | USA | SR | Benefit | Potentially both neonate/maternal | Benefit worthwhile use of resources | Gilson states that "An increase in the incidence of pediatric septic work-ups has been cited as a 'hidden cost' of the CDC screening-based strategy…..the incidence of both strongly suspected sepsis and low probability sepsis in this population of term infants was low. Length of stay was likewise not significantly different between groups (Table 2). Actual costs were not calculated in this study, the low incidence of extended stays in the infants in the screening-based strategy groups are unlikely to elevate total costs significantly" |
| Gopal Rao et al 2017 (29) | UK | SR | Benefit | Neonate - not specified | Benefit more effective treatment | "This study shows that after adjustment for differences in ethnicity of mothers, there was a fivefold increase in EOGBS rate in the postscreening period compared with the screening period….We believe that this observational study comparing prescreening, screening and postscreening periods provides further evidence that [screening based IAP] SBIAP approach is significantly more effective in prevention of EOGBS in our setting. |
| Gosling et al 2002 (30) | New Zealand | NS O | Benefit | Neonate - not specified | Benefit more effective treatment | "Infants born at these three hospitals [with no screening policy] during the 1999 national study were at significantly greater risk of early onset GBS disease than those born at the 14 centres (1.44 versus 0.46 per 1000 live births) where prevention policies already existed (5/3471 versus 15/32,724; crude OR = 3.16 (95% CI 1.02, 8.38))." |
| Hafner et al 1998 (31) | Austria | SR | Benefit | Neonate - not specified | Benefit more effective treatment | "What appears to be more important is the statistically significant reduction in the incidence of early-onset sepsis from 20 to 4 cases. This intervention not only reduced the expenses for antibiotics and other drugs but—more significantly—reduced the number of neonates in need of respirator treatment."..."We are convinced that the significant reduction in the incidence of early-onset group B streptococcal sepsis obtained with the protocol applied in period B more than outweighs the cost incurred for screening" |
| Hafner et al 1998 (31) | Austria | SR | Harm | Neonate - not specified | Harm Other (please specify) | "In the other cases antibiotics were omitted either because patients were scheduled for caesarean delivery or because the time to entry into the delivery room was too short to allow handling of the antibiotic dose. In 11 instances the attending staff forgot to administer the antibiotic." |
| Håkansson et al 2017 (32) | Sweden | NS R | Harm | Neonate - not specified | Harm Other (please specify) | "several occasions where IAP was indicated but not given". The authors argue that "Several previous reports on the results of preventive strategies state that many opportunities to administer IAP are missed and that a stricter adherence to guidelines could have reduced the incidence of early-onset GBS disease even further (28–30). The current study concurs with this conclusion, showing that in a large proportion of cases where IAP was indicated but not adequately given, there was probably enough time to do so. The most important issue in the mission to reduce early-onset GBS infection is undoubtedly to increase adherence to guidelines regardless of the preferred strategy." |
| Håkansson et al 2017 (32) | Sweden | NS R | Benefit | Neonate - not specified | Benefit more effective treatment | "There was a significant 50% reduction of early-onset group B streptococcal infection in infants of women with one or more defined risk factors at parturition after implementation of risk-based intrapartum antibiotic prophylaxis" |
| Hong et al 2019 (33) | South Korea | SR | Harm | Potentially both neonate/maternal | Harm overtreatment | "Even after the adoption of universal GBS screening, there was no case of proven EONS due to GBS in period 2. In this aspect, the primary goal of GBS screening was achieved during the universal screening period. However, EONS rate (suspected and proven) and the use of IAP increased significantly. Considering the overuse of intra-partum antibiotics, it is unclear whether routine GBS screening would be beneficial or cost-effective with regard to prevention of neonatal infection in Korea" |
| Horváth et al 2013 (34) | Hungary | NS O | Neutral | Potentially both neonate/maternal | Neutral | "prospective study spanned 16 years. It was designed to confirm the expected benefit of instituting a GBS screening and prophylaxis program at Markusovszky Teaching Hospital. It was also designed to evaluate the effectiveness of early screening—between 30 and 32 rather than 35 and 37 weeks, as recommended by the CDC/ACOG guidelines—in women from populations where premature births are common. Premature delivery being known as an important risk factor for neonatal GBS infection, the screening protocol was tailored and implemented to account for the rate of premature deliveries in Hungary, which by some accounts is as high as 10%. Over the 16 years of the study, labor and delivery personnel were helped greatly by being aware of each woman’s presumed GBS status before delivery. They could initiate a prompt antibiotic intervention as part of the preterm labor management protocol...."Screening women early in a population with a high rate of premature births may simplify preterm labor management. It results, however, in a higher incidence of early onset neonatal GBS disease than when screening is done closer to term" |
| Hung et al 2018 (35) | Taiwan | SO | Neutral | Neonate - not specified | Benefit more effective treatment | "The purpose of the universal screening program was to detect pregnant GBS-carrying women early and to provide prompt preventive treatment. However, it did not directly help decrease the GBS prevalence in pregnant women." |
| Hung et al 2018 (35) | Taiwan | SO | Benefit | Neonate - not specified | Benefit more effective treatment | "the morbidity rate for early-onset infections caused by GBS decreased from the original 0.1% to 0.02%, with a decrease of as high as 80%, indicating that after the implementation of the universal screening policy in Taiwan, the rate for neonatal early-onset infection showed a significant downward trend due to the early detection of pregnant women carrying GBS and the intervention of preventive treatment." |
| Isaacs & Royle 1999 (36) | Australia | NS O | Benefit | Neonate - not specified | Benefit more effective treatment | "A steady fall in EOGBS infections in Australia from 1991 - 1997 has been associated with increasing use of IAP. Increased antibiotic use is probably causal in the fall in GBS, because the incidence of early onset infections caused by other organisms has also fallen. |
| Jeffery & Moses Lahra 1998 (37) | Australia | NS S | Harm | Potentially both neonate/maternal | Harm false negatives | "After intervention, there were eight neonates (29%) with EOGBSD whose mothers had a negative swab at 28 weeks’ gestation, but who were positive at delivery, representing either a false-negative result or a later acquisition of the organism" |
| Jeffery & Moses Lahra 1998 (37) | Australia | NS S | Benefit | Neonate - not specified | Benefit more effective treatment | "In conclusion, at KGVH, universal antepartum screening at 28 weeks’ gestation and intrapartum antibiotics for maternal carriers of GBS have coincided with a significant decrease in the incidence of EOGBSD to 0.2 per 1000 live births for blood culture- positive disease and to 0.6 per 1000 live births for urine streptococcal antigen-positive disease. The 84% reduction in EOGBSD has been achieved by treating 244 neonates in labor to prevent disease in 1 neonate" |
| Jeffery & Moses Lahra 1998 (37) | Australia | NS S | Harm | Neonate - not specified | Harm Other (please specify) | "An audit indicated that by the 8th year, 90% of all pregnant women were screened by a low vaginal swab at 28 weeks and 10.5% were carriers. After intervention, of the 28 neonates with EOGBSD, 64% were associated with departure from the protocol. ...Of the 28 neonates with EOGBSD in the 8 years after the introduction of the intervention strategy, 18 (64%) were born to mothers who were not screened and/or treated with intrapartum antibiotics as directed by the protocol. The two neonatal deaths occurred in this group (Table 3). The antenatal screening status of these 18 mothers included 14 who were not swabbed antenatally and three who had a positive swab at 28 weeks and were not treated in labor. One mother had a negative swab but had a previous neonate with EOGBSD and was not treated in labor. Twelve of the 14 mothers in the first group went into preterm labor, and the other 2 mothers went on to deliver at term gestation. Of these 2 mothers, 1 developed a fever in labor and was not treated with intravenous antibiotics. Her neonate developed blood culture-positive EOGBSD. The remaining neonate was born to a mother who was not swabbed and not treated with intrapartum antibiotics. A postnatal vaginal swab was positive for GBS. Additional reduction seems possible by improving staff compliance with the protocol. |
| Johansson Gudjónsdóttir et al 2019 (38) | Sweden | NS R | Benefit | Neonate - not specified | Benefit more effective treatment | "There has been a decrease in the incidence of neonatal early-onset infections compared to previous studies in western Sweden. The incidence of GBS infections was not as low as in other reports. Further studies are needed to assess if screening-based intra partum antimicrobial prophylaxis instead of a risk factor-based approach for identifying candidates for intrapartum antimicrobial prophylaxis would be a better option for this study area" |
| Katz et al 1994 (39) | USA | NS S | Benefit | Neonate - not specified | Benefit more effective treatment | "we found antepartum screening and intrapartum chemoprophylaxis of all group B streptococcal carriers to be an acceptable and effective protocol for reducing perinatal group B streptococcal infections" |
| Katz et al 1999 (40) | USA | NS O | Unclear |  | Benefit more effective treatment | "Before the GBS policy, there were 16,272 deliveries with a 2.24/1000 deliveries rate of early-onset GBS sepsis (n=35); after initiating the GBS policy, 9130 deliveries occurred with an early-onset GBS sepsis rate of 2.29/1000 (n=20). Early-onset GBS sepsis case fatality rates before and after initiation of the policy were 14.3% and 0%, respectively (p=0.09). Antibiotic use almost doubled (relative risk 1.84; confidence interval, 1.74 to 1.93, p 0.001) over the two time periods, and the relative risk of chorioamnionitis decreased to 0.95 (confidence interval, 0.73 to 0.99, p=0.04). Despite universal GBS culturing and very liberal use of antibiotics in labor, we were unable to effect a statistically significant change in the rate of early-onset GBS sepsis or mortality, and there was only a slightly decreased chorioamnionitis rate" |
| Ko et al 2021 (41) | Taiwan | SR | Benefit | Neonate - not specified | Benefit more effective treatment | "The neonatal EOGBS incidence rate was 1.1 per 1000 live births prior to the implementation of GBS screening in Taiwan.6 The incidence of EOGBS has not been evaluated after the institution of a GBS screening policy. The EOGBS incidence at our hospitals was 0.38 per 1000 live births in the era of universal GBS screening, but this decline was not as dramatic as that observed in Western countries"....The authors note that "The incidence rates of EOS, GBS, and Escherichia coli (E. coli) sepsis were similar in Epoch 1 [risk-based] and Epoch 3 [universal]" |
| Kolkman et al 2020 (42) | Netherlands | ROO | Harm | Potentially both neonate/maternal | Harm Other (please specify) | "We found low (maximum of 20%) adherence to the three strategies in women with EOGBS risk factors. This implies that a large proportion of women eligible for preventive treatment do not receive the care needed and are therefore undertreated. A possible explanation for the level of under treatment is non-performance of the core elements by care providers due to imprecise recommendations such as an unclear definition of GBS bacteriuria in the current pregnancy [37]. Clari cation of some recommendations may therefore improve adherence". |
| Kolkman et al 2020 (42) | Netherlands | ROO | Harm | Potentially both neonate/maternal | Harm overtreatment | "This study shows the lowest ‘overall adherence’ occurred in the combination strategy."...."In the combination strategy, a significant proportion of women without EOGBS risk factors were overtreated mainly by performing another screening during labour, while screening was also performed during pregnancy. This may result in unnecessary interventions for example IAP or longer observation of the newborn. However, a low level of ‘overall adherence’ to the combination strategy is primarily determined by under-treatment, rather than by overtreatment." |
| Lee et al 2021 (43) | China | SR | Benefit | Neonate - not specified | Benefit more effective treatment | "The incidence of early-onset GBS sepsis among infants of women who were not screened was 0.41 per 1,000 live births (95% confidence interval [CI] 0.19–0.77) when compared to infants of women who were screened, for whom the sepsis incidence was zero per 1,000 live births (95% CI 0–0.19; p = 0.005). CONCLUSION: Our data suggests that routine culture-based screening of pregnant women for GBS colonisation is a better preventive strategy for early-onset GBS sepsis in neonates when compared to clinical risk factor-based screening." |
| Levine et al 1999 (44) | USA | NS O | Benefit | Neonate - not specified | Benefit more effective treatment | "We found a significant increase in intrapartum chemoprophylaxis between the first and fourth quarters of 1997 corresponding to the increased physician awareness of published guidelines. As expected, the incidence of neonatal GBS sepsis was drastically reduced (from 1.7/1000 live births to 0 in 3730 births, P=0.02). Unfortunately, there was a concomitant increase in the incidence of gram-negative sepsis (0.29/1000 vs. 1.3/1000, P =.02). The overall incidence of neo natal sepsis remained unchanged (2.7/1000 vs. 2.1/1000, P= .69). Conclusions: Published guidelines have encouraged physicians to increase the use of intrapartum chemoprophylaxis to reduce vertical transmission of GBS. This study confirms the efficacy of this approach. Unfortunately, this reduction comes at the cost of increasing the incidence of ampicillin- resistant gram-negative neonatal sepsis with a resultant increased mortality." |
| Lin et al 2011 (45) | Taiwan | NS S | Benefit | Neonate - not specified | Benefit more effective treatment | "GBS screening plus IAP is effective in decreasing the incidence of GBS EOS" |
| Locksmith et al 1999 (i) and (ii) (46) | USA | SOO | Harm | Potentially both neonate/maternal | Harm false negatives | "Protocol failures occurred when the protocol was followed correctly but the patient either was not a candidate for prophylaxis or was given antibiotics but still had infection develop in the infant. Protocol violations were noted when the practitioner failed to provide the indicated prophylaxis or when the patient either refused treatment or came to the labor and delivery suite too late for antibiotics to be administered"...."Despite the effectiveness of the ACOG and universal screening protocols in finding more women at risk for group B streptococcal transmission, however, at least half of the neonatal group B streptococcal infections with these protocols occurred when the mothers were not considered candidates for prophylaxis. Possible reasons for protocol failure include false-negative culture results, sepsis occurring before the mother was able to receive prophylaxis, and delivery occurring before adequate fetal antibiotic tissue levels could be achieved." |
| López Sastre et al 2005 (47) | Spain | NS S | Benefit | Neonate - not specified | Benefit more effective treatment | Authors concluded that "in this Spanish multicentre surveillance study of neonatal sepsis of vertical transmission, a substantial decline in the incidence of culture-proven vertical sepsis with a significant reduction of GBS sepsis was found, although decreases were more marked in hospitals that had established prevention policies before 1998, the time at which consensus guidelines for the adoption of intrapartum antibiotic prophylaxis were released. In this group of hospitals there was also a decrease in the mortality rate. Fluctuations in the incidence of E. coli infection suggest the need for continuing epidemiological surveillance" |
| Lu et al 2022 (48) | Taiwan | NS S | No harms or benefits reported |  |  |  |
| Lukacs & Schrag 2012 (49) | USA | NS SO | No harms or benefits reported |  |  |  |
| Ma et al 2018 (50) | Hong Kong, PRC | SR | Benefit | Neonate - not specified | Benefit more effective treatment | "The present study showed that the incidence of EOGBSD decreased by 75% to 0.24 per 1000 live births after the implementation of the universal antenatal culture-based screening program and IAP to the colonized women." |
| Ma et al 2018 (50) | Hong Kong, PRC | SR | Harm | Potentially both neonate/maternal | Harm false negatives | False negatives were specifically calculated for this paper. Authors report that "the incidence of EOGBSD was higher in the preterm infants than the term infants (0.61 per 1000 vs 0.23 per 1000 live births), but 86.7% of the EOGBSD occurred in the term infants. The main contributing factors to the latter included a false negative screening result (41.3%), lack of screening (20.7%) and an unavailability of a colonized result at labour (13.8%)...although the incidence of EOGBSD was reduced, the remaining problems included preterm deliveries, lack of screening, unavailability of GBS screening results, failure of IAP, and most importantly false negative screening. The latter contributed to as many as 40% of EOGBSD in term newborns. Prenatal screening may be more efficient if performed intrapartum than at 35–37 weeks’ gestation." |
| Main & Slagle 2000 (51) | USA | NS S | Benefit | Neonate - not specified | Benefit more effective treatment | "During the baseline period the rate of early-onset group B streptococcal infection was 1.1 cases per 1000 births (n = 8 cases per 6829 births). With the risk-based strategy the rate was also 1.1 cases per 1000 births (15 cases/13,270 births). After we switched to the culture-based protocol for 2 years, there were no cases of early-onset group B streptococcal infections among 9304 births (P = .001; χ2 = 10.9). There were no increases in other early-onset infections or in antibiotic resistance. In our setting, which included good prenatal care and good communication between laboratories and the hospital, the approach based on maternal culture at 35 to 37 weeks’ gestation and treatment during labor of all patients with positive results significantly reduced early-onset group B streptococcal infections without increasing infections from resistant organisms." |
| Matsubara et al 2013 (52) | Japan | NS S | Benefit | Neonate - not specified | Benefit more effective treatment | "Our results reveal a very low incidence of EOD and LOD, but mortality and morbidity rates remain substantial. There are significant associations between EOD case fatality and prematurity as well as low birth weight, and between sequelae and the diagnosis of meningitis. National guidelines have had no effect on the incidence of EOD but have improved the prognosis" |
| Matsubara et al 2007 (53) | Japan | NS S | Harm | Potentially both neonate/maternal | Harm Other (please specify) | "This study demonstrated that most regional hospitals had some prevention protocols, and all practices were based on a combination of universal prenatal screening and intrapartum chemoprophylaxis. None of the hospitals had implemented a risk-based approach. The preventive practices varied widely among the institutes. Given the lack of official guidelines in Japan, this variation is understandable. More than 80% of hospitals surveyed had introduced some screening-based preventive practices for EOGBS, but the methods of bacteriological screening and chemoprophylaxis varied widely....However, several aspects of the institutional practices we found would not be optimal to fully maximize the prevention of GBS vertical transmission. The first is the timing of specimen collection for bacteriological screening.... Second, most hospitals with preventive practices collected swabs from pregnant women from the vagina alone...Third, in regard to the interval between antibiotic administrations, 12 (52%) hospitals administered antibiotics only once or performed repeated administration at intervals of 8 h or more....Finally, in the majority of hospitals with bacteriological screening, oral antibiotics were prescribed antenatally" |
| Mirsky et al 2020 (54) | USA | SS | Harm | Potentially both neonate/maternal | Harm false negatives | "We found that nearly three quarter of the term cases of early-onset GBS were in the index screen negative group, consistent with prior reports that found that >60% of infants with early-onset GBS were born to GBS screen negative women [11,12]. This suggests that culture-based GBS screening may be missing a small proportion of those that are colonized (i.e. false negative results) or that those women became colonized in the interim period between screening and delivery. |
| O’Sullivan et al 2019 (55) | UK and Ireland | NS R | Unclear | Neonate - not specified | Benefit more effective treatment | "856 cases of group B streptococcus were identified in 2014–15, an incidence of 0·94 per 1000 livebirths (95% CI 0·88–1·00). Incidence for early-onset disease (n=517) was 0·57 per 1000 livebirths (95% CI 0·52–0·62), and for late-onset disease (n=339) was 0·37 per 1000 livebirths (0·33–0·41). 53 infants died (case fatality rate 6·2%), of whom 27 had early-onset disease (case fatality rate 5·2%) and 26 had late-onset disease (case fatality rate 7·7%)" The authors conclude: he incidence of invasive infant group B streptococcal disease in the UK and Ireland has increased since a comparable study done in 2000–01. The burden of early onset disease has not declined despite the introduction of national prevention guidelines. New strategies for prevention are required" |
| Petersen et al 2014 (56) | Denmark | NS R | No harms or benefits reported |  |  |  |
| Phares et al 2008 (57) | USA | SO | Benefit | Neonate - not specified | Benefit more effective treatment | "Surveillance identified 1232 cases of early onset disease. Disease incidence decreased 27% (95% CI, 16%-37%) after the 2002 release of revised early onset disease prevention guidelines, from 0.47 per 1000 live births in 1999-2001 to 0.34 per 1000 live births in 2003-2005 However, successive small increases in incidence occurred in 2004 and 2005 (Figure 2). These increases were driven primarily by black infants, in whom there was a significant increase in incidence from 0.52 per 1000 in 2003 to 0.89 per 1000 in 2005 (X2 for trend, 7.9; P = .005). From 2003-2005, white infants born at term were the only group in whom incidence trended toward a decline, from 0.23 per 1000 to 0.16 per 1000 (X2 for trend, 3.1; P =.08). |
| Poulain et al 1997 (58) | France | NS O | Benefit | Neonate - not specified | Benefit more effective treatment | "We noted a significant decrease in the rate of neonatal GBS infection (4.5 per thousand in 1993:1.6 per thousand during the study), confirming the fact that antibiotic therapy during labor reduces the rate of neonatal GBS sepsis" |
| Puopolo & Eichenwald 2010 (59) | USA | NS SR | Benefit | Neonate - not specified | Benefit more effective treatment | "Our study of EOS over an 18-year period at a single, large, maternity center reveals decreases in all-cause EOS rates for both term and VLBW infants, with no change in the incidence of ampicillin-resistant infections. In addition, the implementation of a screening-based approach to GBS IAP in our institution resulted in a significant increase in the proportion of deliveries with exposure to antibiotics predominantly active against Gram-positive organisms but no change in the use of ampicillin" |
| Puopolo & Eichenwald 2010 (59) | USA | NS SR | Benefit | Neonate - not specified | Benefit Other (please specify) | "We found that the overall incidence of neonatal EOS decreased in the era of GBS IAP, among all newborns and among VLBW infants. The primary reason for this change was the decreased incidence of EOS caused by GBS and by other streptococcal species. The significant decrease in the incidence of EOS caused by other streptococcal species coincident with IAP has not been reported previously and provides evidence that infections caused by non-GBS streptococcal organisms also may be susceptible to the strategy of IAP". The authors conclude that "Short-term use of narrow-spectrum antibiotics primarily active against Gram-positive organisms for GBS IAP resulted in decreases in the incidence of EOS in all infants and in VLBW infants, with no change in the incidence of ampicillin-resistant EOS. The association of peripartum ampicillin exposure with the development of ampicillin-resistant infections suggests that obstetricians should be encouraged to use penicillin G preferentially for routine GBS IAP" |
| Reisner 2000 (60) | USA | SR | No harms or benefits reported |  |  |  |
| Renner et al 2006 (61) | Switzerland | NS O | Benefit | Neonate - not specified | Benefit more effective treatment | "The incidence of early-onset GBS sepsis was reduced from 1/1000 (G1) [no screening] to 0.53/1000 (G2 [other screening strategy]). We observed a significant reduction of overall intrapartum risk factors in cases of GBS sepsis." |
| Riley 2003 (62) | USA | SR | Harm | Neonate - not specified | Harm Other (please specify) | "the overall compliance of these strategies is similar. However, when we evaluate ‘‘real-world’’ compliance, we are able to demonstrate that evaluating all risk factors, including lapses in the intended protocol, occurred more frequently with certain risk factors such as rupture of membranes >18 hours. More importantly, our data shows that evaluating ‘‘real-world’’ application of these strategies demonstrates the complexity of the culture-based approach. Thus, failure to perform the culture at the appropriate gestational age is a potentially harmful breach of the protocol in that the sensitivity of the culture is diminished if the culture is done <5 weeks from delivery.17 Lack of culture data in labor shifts women into the risk-based strategy and decreases the benefits of using the culture-based strategy" |
| Rottenstreich et al 2019 (63) | Israel | SR | Benefit | Neonate - not specified | Benefit more effective treatment | "This study showed that the transition from a risk-based GBS screening to universal screening led to a significant decrease in the EOGBS rate, in a single center" |
| Rottenstreich et al 2019 (63) | Israel | SR | Harm | Neonate - not specified | Harm Other (please specify) | In the time period (2005-2009): 18 diagnosed with GBS. Of these, 3 were positive, 0 were negative and 15 were not screened. In the time period (2010-2016): 18 diagnosed with GBS. Of these, 6 were positive, 4 were negative and 9 were not screened. |
| Sakata et al 2012 (64) | Japan | NS S | Harm | Neonate - not specified | Harm Other (please specify) | "The time from administration of antimicrobial agents until delivery is also a problem. ...All seven neonates found to be positive for GBS in the present series had been born within 4 h after administration of antimicrobial agents. In the present study, all our seven neonates positive for GBS were born less than 4 h after administration of antibiotics" |
| Sakata et al 2012 (64) | Japan | NS S | Harm | Neonate - not specified | Harm Other (please specify) | No significant differences of early-onset GBS infection/ 1000 live births between the no screening period (1997-2001) and the universal screening period (2009-2011). The authors highlight the issues with timing of screening, and the challenge of identifying "pregnant women who are negative for GBS at screening but positive immediately before delivery" |
| Schrag et al 2002 (65) | USA | SR | Benefit | Neonate - not specified | Benefit more effective treatment | "The risk of early-onset disease was significantly lower among the infants of screened women than among those in the risk-based group (adjusted relative risk, 0.46; 95 percent confidence interval, 0.36 to 0.60). Because women whose providers had no strategy for prophylaxis may have been misclassified in the risk-based group, we excluded all women with risk factors and adequate time for prophylaxis who did not receive antibiotics. The adjusted relative risk of early-onset disease associated with the screening approach in this secondary analysis was similar — 0.48 (95 percent confidence interval, 0.37 to 0.63)"..Authors conclude "Routine screening for group B streptococcus during pregnancy prevents more cases of early-onset disease than the risk-based approach" |
| Schuchat 2002 (66) | USA | SR | Harm | Potentially both neonate/maternal | Harm Other (please specify) | "Among women with positive prenatal screening results for group B streptococcal colonization, 78% (95% CI 67–89%) received intrapartum antibiotics.....Intrapartum antibiotics were more likely to be administered to African Americans than to whites (97 vs. 75%, p = 0:006)" |
| Schuchat 2002 (66) | USA | SR | Harm | Potentially both neonate/maternal | Harm Other (please specify) | "Among women with positive prenatal screening results for group B streptococcal colonization, 78% (95% CI 67–89%) received intrapartum antibiotics....Intrapartum antibiotics were more likely to be administered to ...patients with private insurance, compared with those covered by Medicaid (89 vs. 57%, p = 0:05)" |
| Schuchat 2002 (66) | USA | SR | Harm | Potentially both neonate/maternal | Harm Other (please specify) | "There were 173 (21.8%;95%CI 18–26%) women in the audit who were not screened for group B streptococcus prenatally but who had one of the risk criteria for intrapartum prophylaxis. Of these, 45% (95% CI 34–56%) received intrapartum prophylaxis. Intrapartum antibiotics were more likely to be administered to high risk unscreened women when delivery occurred before 34 weeks (68 vs. 31% for deliveries at 34–36 weeks and 43% for deliveries at 37 weeks or more, p = 0:03 " |
| Schuchat 2002 (66) | USA | SR | Harm | Potentially both neonate/maternal | Harm Other (please specify) | "The shorter the interval in the hospital before delivery, the less likely it was for the antibiotics to be administered (16.7 vs. 55.7% with intervals less than 8 vs. 8 or more hours before delivery, p < 0:0001). Among women with obstetric risk factors, those admitted in the evening or night shift were less likely to receive intrapartum antibiotics (34 vs. 57%, p = 0:03).Women who delivered on the weekend were also less likely to receive intrapartum antibiotics (25 vs. 53%, p = 0:009)" |
| Schuchat 2002 (66) | USA | SR | Harm | Potentially both neonate/maternal | Harm Other (please specify) | Of 992 records requested, 868 (88%) were abstracted and analyzed. Thirty-six percent of women had prenatal screening for group B streptococcus and 26% had been tested for human immunodeficiency virus (HIV), while 97–99% of women had been screened prenatally for hepatitis B surface antigen, rubella, and syphilis. Of those women tested, 17% were detected as group B streptococcus carriers, and 78% of these received intrapartum antibiotic prophylaxis. Among women who were not screened for group B streptococcus prenatally, 22% met risk-based criteria for prophylaxis, but only 45% of these received intrapartum prophylaxis".....Authors conclude "In 1996, the majority of women who delivered in Connecticut were not tested prenatally for group B streptococcus and the majority of those not tested in whom there was an indication for prophylaxis were not treated. Compliance with group B streptococcus prevention recommendations can be improved through increased prenatal testing and/or better recognition of risk-based criteria for intrapartum prophylaxis" |
| Share et al 2001 (67) | USA | NS O | Benefit | Neonate - not specified | Benefit more effective treatment | Since the implementation of the guidelines, we have documented a dramatic decrease in the incidence of early onset GBS infection from 2.7 to 0.4 per 1,000 live births. As would be anticipated with compliance with the guidelines, we noted an increase in detection of maternal colonization as well as an increase in maternal intrapartum antibiotic prophylaxis. This was accompanied by a reduced number of invasive procedures on neonates and fewer neonates requiting empirical antibiotic treatment. |
| Share et al 2001 (67) | USA | NS O | Benefit | Neonate - not specified | Neutral | "We have continued to monitor nosocomial infections at our institution and, despite more widespread intrapartum antibiotic use, there has been no evidence for emerging disease secondary to resistant gram-negative organisms in our nursery population" Data not presented in this paper |
| Sutkin et al 2005 (68) | USA | NS S | Benefit | Neonate - not specified | Benefit more effective treatment | "Institution of a protocol for GBS antibiotic prophylaxis significantly decreased the rate of GBS neonatal sepsis but did not increase the rate of non-GBS neonatal sepsis. Antibiotic resistance patterns of these organisms were not affected" |
| Towers & Briggs 2002 (69) | USA | NS SR | Neutral | Neonate - not specified | Neutral | "However, the development of early-onset group B streptococcal neonatal sepsis significantly decreased as the use of antepartum antibiotics increased. Thus, the number of prevented infections from antepartum antibiotic use may still outweigh the problems that are seen when resistant bacterial infections arise. Nevertheless, based on the current protocols, a large number of parturients are candidates for antibiotic chemoprophylaxis and this, in conjunction with the global concern of bacterial drug resistance, should be motivation to examine alternative methods, such as vaginal washing or immunotherapy, for decreasing infection" |
| Trijbels-Smeulders 2006 (70) | The Netherlands | NS O | No harms or benefits reported |  |  |  |
| Trijbels-Smeulders 2007 (71) | The Netherlands | NS O | Benefit | Neonate - not specified | Benefit more effective treatment | "After the introduction of guidelines the incidence of proven early onset sepsis decreased considerably from 0.54 per 1000 live births in 1997–8 to 0.36 per 1000 live births in 1999–2001." |
| Trijbels-Smeulders 2007 (71) | The Netherlands | NS O | Unclear | Neonate - not specified | Neutral | However, there was no decrease in the incidence of meningitis and the case fatality rate in the first week of life. The incidence of late onset sepsis also remained unchanged…the authors also state "The overall effect of the Dutch guidelines is disappointing because of the limited decrease in the incidence of proven early onset GBS sepsis, and no decrease in mortality, incidence of meningitis and probable sepsis" |
| Trollfors et al 2022 (72) | Sweden | NS R | Benefit | Neonate i.e. 7-28 days | Benefit more effective treatment | "The overall incidence among neonates was 6.0/100 000 live births/year, but there was a significant decrease from the year 2012, when intrapartum antibiotic prophylaxis was fully implemented. Before 2012, the incidence was 7.3/10 000 live births/year, while between 2012 and 2016 it was 3.4/10 000 live births/year (p=0.002, chi-squared test for trend; p=0.003,logistic regression)" Note: Although the risk-based guidelines were introduced in 2008, they were not fully implemented until end of 2011 in most hospitals. |
| Uy et al 2002 (73) | USA | NS O | Harm | Neonate - not specified | Harm Other (please specify) | A proportion of cases identified during the study period was possibly preventable by retrospective application of the current CDC guidelines. Most of the possibly preventable cases were identifiable by the risk factor arm of the CDC guidelines. However, the risk factor– based strategy failed to identify a substantial number of mothers whose infants developed EOGBS infection" Authors looked at "Possibly preventable cases: EOGBS cases whose mothers were considered candidates for intrapartum chemoprophylaxis based on either one of the strategies proposed by the CDC consensus guidelines but who did not receive recommended antibiotics. ....The current CDC consensus guidelines were applied retrospectively to determine preventability of the EOGBS cases identified(Table 5). Twenty of the case mothers (36%) received intrapartum antibiotics. Among this group of women, three received the recommended course of prophylactic antibiotics (two or more doses of prophylactic antibiotics for the presence of either clinical or culture risk factors, starting at least 4 hours before delivery — ‘‘adequate prophylaxis’’). Two of these case mothers received two doses of ampicillin and the third received two doses of clindamycin. We considered these cases to be nonpreventable. One of the three infants born to these women died due to GBS infection, despite the intrapartum maternal antibiotics received. Multiple4 maternal risk factors were found in this particular case and the mother was sick enough to warrant continued treatment postpartum. The remaining 17 case mothers in this group received a single dose of antibiotics less than 4 hours prior to delivery (‘‘inadequate prophylaxis’’). Twelve of these 17 women received their antibiotics less than an hour prior to delivery, whereas the remaining five received antibiotics 1 to 3 hours prior to delivery. EOGBS infection in this group of infants was considered possibly preventable had their mothers received the recommended course of antibiotic prophylaxis. An additional 23 case mothers with clinical risk factors and/or positive maternal cultures for GBS, who would have been eligible for prophylaxis, did not receive intrapartum antibiotics. These cases were judged to be possibly preventable. The remaining 13 cases had no identifiable maternal clinical risk factors present nor were any maternal cultures sent. If the preventability of these cases were based on the risk factor strategy of the current guidelines, these cases would be nonpreventable because their mothers would not have been identified as eligible for intrapartum prophylaxis. In the absence of culture data, preventability based on the screening culture strategy cannot be determined" |
| Uy et al 2002 (73) | USA | NS O | Benefit | Neonate - not specified | Benefit more effective treatment | Fifty - six cases of EOGBS infection occurred among 53,088 live births. The incidence declined from 1.5 / 1000 before any guidelines to 0.67 / 1000 after AAP / ACOG guidelines ( p = 0.004 ), and continued to decline after the CDC consensus statement ( 0.28 / 1000 ) ( p = 0.38 ). IAP remained stable ( 33% of at risk mothers) until after introduction of the CDC consensus guidelines ( 59%, p = 0.02 ). |
| van den Hoogen et al 2010 (74) | The Netherlands | NS O | No harms or benefits reported |  |  |  |
| van Dyke et al 2009 (75) | USA | SO | Harm | Neonate - not specified | Harm Other (please specify) | "Mothers who delivered preterm were less likely to be screened than mothers who delivered at term (relative risk, 0.56; 95% CI, 0.51 to 0.62)"…."Only 50.3% of the mothers who delivered preterm were screened before delivery, and 17.8% of the women who delivered preterm were screened at admission; among women for whom the interval between admission and delivery was 48 hours or more, 58.9% were screened at admission. In a univariate analysis, delivery at less than 34 weeks’ gestation was the only significant factor associated with not being screened before delivery (relative risk, 1.5; 95% CI, 1.2 to 1.8)." |
| van Dyke et al 2009 (75) | USA | SO | Harm | Neonate - not specified | Harm Other (please specify) | In a univariate analysis, several subgroups of mothers (Table 2) had lower screening rates than the overall population of mothers who delivered at term with the lowest rates of screening among women with inadequate prenatal care (76.9% of these women were screened) and women with a history of drug use (80.6% were screened)" |
| van Dyke et al 2009 (75) | USA | SO | Harm | Neonate - not specified | Harm Other (please specify) | In the multivariable model, black race, Hispanic ethnic group, previous delivery of a live infant, history of drug use, and inadequate prenatal care remained significantly associated with not being screened (Table 2); there were no significant interactions between variables. |
| van Dyke et al 2009 (75) | USA | SO | Harm | Neonate - not specified | Harm Other (please specify) | Mothers who delivered preterm were less likely to receive chemoprophylaxis when indicated than mothers who delivered at term (relative risk, 0.81; 95% CI, 0.75 to 0.87). Among women who delivered preterm and were positive for group B streptococcus, 84.5% received chemoprophylaxis (Table 3). However, only 63.4% of women who delivered preterm and had unknown colonization status received intrapartum antibiotics...Women were less likely to receive chemoprophylaxis when the interval between admission and delivery was less than 4 hours than when the interval was 4 hours or more (Table 3). |
| van Dyke et al 2009 (75) | USA | SO | Harm | Neonate - not specified | Harm Other (please specify) | The length of time between admission and delivery was the only factor associated with missed chemoprophylaxis in a univariate analysis. Women who were positive for group B streptococcus or had a history of group B streptococcus were less likely to receive chemoprophylaxis when the interval between admission and delivery was less than 4 hours than when the interval was 4 hours or more. |
| van Dyke et al 2009 (75) | USA | SO | Harm | Neonate - not specified | Harm Other (please specify) | "The largest portion of cases of group B streptococcal disease in term infants (61.4%) occurred in the infants of women who had been screened and who had tested negative for group B streptococcus (Table 4).....To determine whether the observed number of cases of group B streptococcal disease in infants born to mothers with negative cultures (i.e., false negative cases) was higher than the number anticipated, we estimated the number of false negative cases that would be expected, using assumptions from our cohort and findings from previous studies. We assumed that antenatal culture-based screening was 96% specific for colonization status at delivery,19 that the percentage of newborns who would be colonized with group B streptococcus when chemoprophylaxis was not administered was 50%,2 and that the incidence of disease among colonized newborns ranged from 5.1 cases per 1000 live births among newborns with no risk factors to 10 cases per 1000 live births among newborns with risk factors.20 On the basis of these assumptions, we expected that there would be 44 to 86 cases of group B streptococcal disease among term infants who were born to women with negative results of prenatal screening for group B streptococcus — 30 to 72 fewer cases than the 116 cases we observed. |
| Vergani et al 2002 (76) | Italy | NS SR | Benefit | Neonate - not specified | Benefit more effective treatment | "Compared with the no prophylaxis group (rate = 4/8,573), introduction of universal screening (rate = 0/13,754, p = 0.02) but not of prophylaxis for risk factors alone (rate = 1/10,303, p = 0.18) significantly decreased the occurrence of GBS specific neonatal mortality. Universal screening decreased, though not significantly, the GBS-specific neonatal morbidity rates compared with a policy based on risk factors alone (0.4/1000 vs. 0.8/1000, p = 0.29). Our study had a power to detect a 0.7/1000 difference in the rate of specific morbidity between the two chemoprophylaxis policies (a = 0.05, p= 0.80). Intrapartum prophylaxis for GBS, using universal screening or risk factors, is associated with a significant reduction in the specific neonatal mortality rate compared with no prophylaxis. Universal screening for GBS leads to a decrease in specific GBS morbidity compared with screening using risk factors alone". They also report that "In our experience, implementation of a strategy based on universal screening with rectovaginal cultures is associated with a greater than 10-fold reduction in the rate of early-onset GBS neonatal mortality compared with no strategy. Such a significant reduction in GBS-related mortality was not observed when risk factors alone were used for GBS prophylaxis." |
| Vergani et al 2002 (76) | Italy | NS SR | Harm | Neonate - not specified | Harm false negatives | "Among the variables that may negatively impact the results of universal screening, the chief one is poor compliance. However, in our series the majority of affected neonates during universal screening were due to precipitate delivery or false-negative culture results" |
| Wicker et al 2019 (77) | Germany | SO | Benefit | Neonate - not specified | Benefit more effective treatment | "We compared our results with those from a previous study by employing an equivalent design (2001–2003). We detected a 32% reduction in GBS incidence, from 0.47 per 1000 live births (n = 679) in 2001–2003 to 0.34 per 1000 live births (n = 450) in 2009–2010. This decline primarily is tied to a reduced number of GBS cases in children under 1 week of age"....."the decline in infant GBS infections in Germany in relation to the newly implemented, culture-based universal screening approach.....In conclusion, our analysis indicates a 32% decline in cases of infant-invasive GBS infection between the years 2003 and 2010 in Germany—a change due primarily to a decline in EOD cases. This suggests that the recommendation for universal GBS screening for all pregnant women between 35 and 37 weeks gestation has contributed to this decline. |
| Youden et al 2005 (78) | Canada | SR | Harm | Maternal | Harm Other (please specify) | "Study participants were significantly less knowledgeable about GBS infection than about other infections screened for during pregnancy, such as rubella, hepatitis B, and HIV (Figure 1). Overall, participants responded correctly to 50% (95% CI 47.2-52.8) of the questions asked. Participants screened by culture at 35 to 37 weeks' gestation answered a higher proportion of GBS knowledge questions correctly. than did those screened by risk factors (P < 0.001). When asked how serious a threat they felt a specific infection posed to their baby during pregnancy and delivery, study participants answered "very high" significantly less often for GBS than for the other infections (27.9%; 95% CI 22.7-33.6). In addition, they answered "not sure" significantly more often when asked this question about GBS than about other infections (Figure 2). |
| Youden et al 2005 (78) | Canada | SR | Benefit | Maternal | Benefit Informed decision | "Women screened by culture at 35 to 37 weeks' gestation were more knowledgeable than those screened by risk factors. This may be because appropriate counselling is more likely to take place when a procedure (vaginal-rectal swab) is performed than during labour."...Authors conclude that The overall lack of knowledge about GBS infection and its consequences and the resultant lack of concern may contribute to the poor compliance with screening recommendations. Women who are knowledgeable may be more likely to be counselled and screened according to recommendations since they are better equipped to advocate for proper care" |
| Youden et al 2005 (78) | Canada | SR | Harm | Potentially both neonate/maternal | Harm overtreatment | "A comparison of the two approaches in our study also showed that the risk factor approach identified proportionately more women needing intrapartum chemoprophylaxis than the culture-based approach (25.2% vs. 19.4%). It would appear that universal use of the culture-based approach could have reduced antibiotic usage by 23%. Minimizing antibiotic usage (and hence reducing the risk of developing antibiotic resistance) is a factor to be considered by a centre when it selects a GBS screening method" |

References

1. Abdelmaaboud M, Mohammed AF. Universal screening vs. risk-based strategy for prevention of early-onset neonatal Group-B streptococcal disease. *J Trop Pediatr* 2011; 57 6:444-50.

2. Al Luhidan L, Madani A, Albanyan EA, Al Saif S, Nasef M, AlJohani S, et al. Neonatal Group B Streptococcal Infection in a Tertiary Care Hospital in Saudi Arabia: A 13-year Experience. *Pediatr Infect Dis J* 2019; 38 7:731-4.

3. Alarcon A, Pena P, Salas S, Sancha M, Omenaca F. Neonatal early onset Escherichia coli sepsis: trends in incidence and antimicrobial resistance in the era of intrapartum antimicrobial prophylaxis. *Pediatr Infect Dis J* 2004; 23 4:295-9.

4. Angstetra D, Ferguson J, Giles WB. Institution of universal screening for Group B streptococcus (GBS) from a risk management protocol results in reduction of early-onset GBS disease in a tertiary obstetric unit. *Aust N Z J Obstet Gynaecol* 2007; 47 5:378-82.

5. Bauserman MS, Laughon MM, Hornik CP, Smith PB, Benjamin DK, Jr., Clark RH, et al. Group B Streptococcus and Escherichia coli infections in the intensive care nursery in the era of intrapartum antibiotic prophylaxis. *Pediatr Infect Dis J* 2013; 32 3:208-12.

6. Bekker V, Bijlsma MW, van de Beek D, Kuijpers TW, van der Ende A. Incidence of invasive group B streptococcal disease and pathogen genotype distribution in newborn babies in the Netherlands over 25 years: a nationwide surveillance study. *Lancet Infect Dis* 2014; 14 11:1083-9.

7. Bizzarro MJ, Raskind C, Baltimore RS, Gallagher PG. Seventy-five years of neonatal sepsis at Yale: 1928-2003. *Pediatrics* 2005; 116 3:595-602.

8. Bjorklund V, Nieminen T, Ulander VM, Ahola T, Saxen H. Replacing risk-based early-onset-disease prevention with intrapartum group B streptococcus PCR testing. *J Matern Fetal Neonatal Med* 2017; 30 3:368-73.

9. Bjornsdottir ES, Martins ER, Erlendsdottir H, Haraldsson G, Melo-Cristino J, Ramirez M, et al. Group B Streptococcal Neonatal and Early Infancy Infections in Iceland, 1976-2015. *Pediatr Infect Dis J* 2019; 38 6:620-4.

10. Brozanski BS, Jones JG, Krohn MA, Sweet RL. Effect of a screening-based prevention policy on prevalence of early-onset group B streptococcal sepsis. *Obstet Gynecol* 2000; 95 4:496-501.

11. Chan YTV, Lau SYF, Hui SYA, Ma T, Kong CW, Kwong LT, et al. Incidence of neonatal sepsis after universal antenatal culture-based screening of group B streptococcus and intrapartum antibiotics: A multicentre retrospective cohort study. *BJOG* 2023; 130 1:24-31.

12. Chen KT, Tuomala RE, Cohen AP, Eichenwald EC, Lieberman E. No increase in rates of early-onset neonatal sepsis by non-group B Streptococcus or ampicillin-resistant organisms. *Am J Obstet Gynecol* 2001; 185 4:854-8.

13. Chen KT, Puopolo KM, Eichenwald EC, Onderdonk AB, Lieberman E. No increase in rates of early-onset neonatal sepsis by antibiotic-resistant group B Streptococcus in the era of intrapartum antibiotic prophylaxis. *Am J Obstet Gynecol* 2005; 192 4:1167-71.

14. Cho CY, Tang YH, Chen YH, Wang SY, Yang YH, Wang TH, et al. Group B Streptococcal infection in neonates and colonization in pregnant women: An epidemiological retrospective analysis. *J Microbiol Immunol Infect* 2019; 52 2:265-72.

15. Clemens CJ, Gable EK. The development of a group B streptococcus prevention policy at a community hospital. *J Perinatol* 2002; 22 7:523-5.

16. Coco AS. Comparison of two prevention strategies for neonatal group B streptococcal disease. *J Am Board Fam Pract* 2002; 15 4:272-6.

17. Daniels JP, Dixon E, Gill A, Bishop J, Wilks M, Millar M, et al. Rapid intrapartum test for maternal group B streptococcal colonisation and its effect on antibiotic use in labouring women with risk factors for early-onset neonatal infection (GBS2): cluster randomised trial with nested test accuracy study. *BMC Medicine* 2022; 20 1:9.

18. Darlow BA, Voss L, Lennon DR, Grimwood K. Early-onset neonatal group B streptococcus sepsis following national risk-based prevention guidelines. *Aust N Z J Obstet Gynaecol* 2016; 56 1:69-74.

19. Davis RL, Hasselquist MB, Cardenas V, Zerr DM, Kramer J, Zavitkovsky A, et al. Introduction of the new Centers for Disease Control and Prevention group B streptococcal prevention guideline at a large West Coast health maintenance organization. *Am J Obstet Gynecol* 2001; 184 4:603-10.

20. Eberly MD, Rajnik M. The effect of universal maternal screening on the incidence of neonatal early-onset group B streptococcal disease. *Clin Pediatr (Phila)* 2009; 48 4:369-75.

21. Ecker KL, Donohue PK, Kim KS, Shepard JA, Aucott SW. The impact of group B Streptococcus prophylaxis on early onset neonatal infections. *J Neonatal Perinatal Med* 2013; 6 1:37-44.

22. Edwards RK, Jamie WE, Sterner D, Gentry S, Counts K, Duff P. Intrapartum antibiotic prophylaxis and early-onset neonatal sepsis patterns. *Infect Dis Obstet Gynecol* 2003; 11 4:221-6.

23. Eisenberg E, Craig AS, Gautam S, Khalil MM, Shaktour B, Schaffner W, et al. Beyond screening: identifying new barriers to early onset group B streptococcal disease prevention. *Pediatr Infect Dis J* 2005; 24 6:520-4.

24. El Helali N, Habibi F, Azria E, Giovangrandi Y, Autret F, Durand-Zaleski I, et al. Point-of-Care Intrapartum Group B Streptococcus Molecular Screening: Effectiveness and Costs. *Obstetrics & Gynecology* 2019; 133 2:276-81.

25. Factor SH, Levine OS, Nassar A, Potter J, Fajardo A, O'Sullivan MJ, et al. Impact of a risk-based prevention policy on neonatal group B streptococcal disease. *Am J Obstet Gynecol* 1998; 179 6 Pt 1:1568-71.

26. Garland SM. Early onset neonatal Group-B streptococcus (GBS) infection - associated obstetric risk-factors. *Aust N Z J Obstet Gynaecol* 1991; 31 2.

27. Gibbs RS, McDuffie RS, Jr., McNabb F, Fryer GE, Miyoshi T, Merenstein G. Neonatal group B streptococcal sepsis during 2 years of a universal screening program. *Obstet Gynecol* 1994; 84 4:496-500.

28. Gilson GJ, Christensen F, Romero H, Bekes K, Silva L, Qualls CR. Prevention of group B streptococcus early-onset neonatal sepsis: comparison of the Center for Disease Control and prevention screening-based protocol to a risk-based protocol in infants at greater than 37 weeks' gestation. *J Perinatol* 2000; 20 8 Pt 1:491-5.

29. Gopal Rao G, Townsend J, Stevenson D, Nartey G, Hiles S, Bassett P, et al. Early-onset group B Streptococcus (EOGBS) infection subsequent to cessation of screening-based intrapartum prophylaxis: findings of an observational study in West London, UK. *BMJ Open* 2017; 7 11:e018795.

30. Gosling IA, Stone PR, Grimwood K. Early-onset group B streptococcus prevention protocols in New Zealand public hospitals. *Aust N Z J Obstet Gynaecol* 2002; 42 4:362-4.

31. Hafner E, Sterniste W, Rosen A, Schuchter K, Plattner M, Asboth F, et al. Group B streptococci during pregnancy: a comparison of two screening and treatment protocols. *Am J Obstet Gynecol* 1998; 179 3 Pt 1:677-81.

32. Hakansson S, Lilja M, Jacobsson B, Kallen K. Reduced incidence of neonatal early-onset group B streptococcal infection after promulgation of guidelines for risk-based intrapartum antibiotic prophylaxis in Sweden: analysis of a national population-based cohort. *Acta Obstet Gynecol Scand* 2017; 96 12:1475-83.

33. Hong JY, . , Kim SH, ., Kim SM, al. e. Evaluation of the early onset neonatal sepsis according to two antenatal group B Streptococcus screening methods: risk-based versus universal screening. *Perinatology* 2019; 30 4:200-7.

34. Horvath B, Grasselly M, Bodecs T, Boncz I, Bodis J. Screening pregnant women for group B streptococcus infection between 30 and 32 weeks of pregnancy in a population at high risk for premature birth. *Int J Gynaecol Obstet* 2013; 122 1:9-12.

35. Hung LC, Kung PT, Chiu TH, Su HP, Ho M, Kao HF, et al. Risk factors for neonatal early-onset group B streptococcus-related diseases after the implementation of a universal screening program in Taiwan. *BMC Public Health* 2018; 18 1:438.

36. Isaacs D, Royle JA. Intrapartum antibiotics and early onset neonatal sepsis caused by group B Streptococcus and by other organisms in Australia. Australasian Study Group for Neonatal Infections. *Pediatr Infect Dis J* 1999; 18 6:524-8.

37. Jeffery HE, Moses Lahra M. Eight-year outcome of universal screening and intrapartum antibiotics for maternal group B streptococcal carriers. *Pediatrics* 1998; 101 1:E2.

38. Johansson Gudjónsdóttir M, Elfvin A, Hentz E, Adlerberth I, Tessin I, B. T. Changes in incidence and etiology of early-onset neonatal infections 1997-2017 - a retrospective cohort study in western Sweden. *BMC Pediatr* 2019; 19 1:490.

39. Katz VL, Moos MK, Cefalo RC, Thorp JM, Jr., Bowes WA, Jr., Wells SD. Group B streptococci: results of a protocol of antepartum screening and intrapartum treatment. *Am J Obstet Gynecol* 1994; 170 2:521-6.

40. Katz PF, Hibbard JU, Ranganathan D, Meadows W, Ismail M. Group B streptococcus: to culture or not to culture? *J Perinatol* 1999; 19 5:337-42.

41. Ko MH CH, Li ST, et al. An 18-year retrospective study on the epidemiology of early-onset neonatal sepsis - emergence of

uncommon pathogens. *Pediatr Neonatol* 2021; 62 5:491–8.

42. Kolkman DGE, Rijnders MEB, Wouters M, Dommelen PV, de Groot CJM, Fleuren MAH. Adherence to three different strategies to prevent early onset GBS infection in newborns. *Women & Birth: Journal of the Australian College of Midwives* 2020; 33 6:e527-e34.

43. Lee J, Naiduvaje K, Chew KL, Charan N, Chan YH, Lin RT, et al. Preventing early-onset group B streptococcal sepsis: clinical risk factor-based screening or culture-based screening? *Singapore Med J* 2021; 62 1:34-8.

44. Levine EM, Ghai V, Barton JJ, Strom CM. Intrapartum antibiotic prophylaxis increases the incidence of gram-negative neonatal sepsis. *Infect Dis Obstet Gynecol* 1999; 7 4:210-3.

45. Lin CY, Hsu CH, Huang FY, Chang JH, Hung HY, Kao HA, et al. The changing face of early-onset neonatal sepsis after the implementation of a maternal group B Streptococcus screening and intrapartum prophylaxis policy--a study in one medical center. *Pediatr Neonatol* 2011; 52 2:78-84.

46. Locksmith GJ, Clark P, Duff P. Maternal and neonatal infection rates with three different protocols for prevention of group B streptococcal disease. *Am J Obstet Gynecol* 1999; 180 2 Pt 1:416-22.

47. Lopez Sastre JB, Fernandez Colomer B, Coto Cotallo GD, Ramos Aparicio A, Grupo de Hospitales C. Trends in the epidemiology of neonatal sepsis of vertical transmission in the era of group B streptococcal prevention. *Acta Paediatr* 2005; 94 4:451-7.

48. Lu IC, Chang YC, Chen YT, Lin HY, Chiu HY, Tsai ML, et al. Epidemiological evolution of early-onset neonatal sepsis over 12 years: A single center, population-based study in central Taiwan. *J Neonatal Perinatal Med* 2022; 15 3:575-82.

49. Lukacs SL, Schrag SJ. Clinical sepsis in neonates and young infants, United States, 1988-2006. *J Pediatr* 2012; 160 6:960-5 e1.

50. Ma TWL, Chan V, So CH, Hui ASY, Lee CN, Hui APW, et al. Prevention of early onset group B streptococcal disease by universal antenatal culture-based screening in all public hospitals in Hong Kong. *J Matern Fetal Neonatal Med* 2018; 31 7:881-7.

51. Main EK, Slagle T. Prevention of early-onset invasive neonatal group B streptococcal disease in a private hospital setting: the superiority of culture-based protocols. *Am J Obstet Gynecol* 2000; 182 6:1344-54.

52. Matsubara K, Hoshina K, Suzuki Y. Early-onset and late-onset group B streptococcal disease in Japan: a nationwide surveillance study, 2004-2010. *Int J Infect Dis* 2013; 17 6:e379-84.

53. Matsubara K, Kawai M, Nakahata T, Kato F, Tsukahara H, Yamakawa M, et al. Procedures for prevention of perinatal group B streptococcal diseases: a multicenter questionnaire survey of hospitals in the Kyoto Neonatal Disease Study Group, Japan. *J Infect Chemother* 2007; 13 1:59-62.

54. Mirsky R, Carpenter DM, Postlethwaite DA, Regenstein AC. Preventing early-onset group B streptococcal sepsis: is there a role for rescreening near term? *Journal of Maternal-Fetal & Neonatal Medicine* 2020; 33 22:3791-7.

55. O'Sullivan CP, Lamagni T, Patel D, Efstratiou A, Cunney R, Meehan M, et al. Group B streptococcal disease in UK and Irish infants younger than 90 days, 2014-15: a prospective surveillance study. *Lancet Infect Dis* 2019; 19 1:83-90.

56. Petersen KB, Johansen HK, Rosthoj S, Krebs L, Pinborg A, Hedegaard M. Increasing prevalence of group B streptococcal infection among pregnant women. *Dan Med J* 2014; 61 9:A4908.

57. Phares CR, Lynfield R, Farley MM, Mohle-Boetani J, Harrison LH, Petit S, et al. Epidemiology of invasive group B streptococcal disease in the United States, 1999-2005. *JAMA* 2008; 299 17:2056-65.

58. Poulain P, Betremieux P, Donnio PY, Proudhon JF, Karege G, Giraud JR. Selective intrapartum anti-bioprophylaxy of group B streptococci infection of neonates: a prospective study in 2454 subsequent deliveries. *Eur J Obstet Gynecol Reprod Biol* 1997; 72 2:137-40.

59. Puopolo KM, Eichenwald EC. No change in the incidence of ampicillin-resistant, neonatal, early-onset sepsis over 18 years. *Pediatrics* 2010; 125 5:e1031-8.

60. Reisner DP, Haas MJ, Zingheim RW, Williams MA, Luthy DA. Performance of a group B streptococcal prophylaxis protocol combining high-risk treatment and low-risk screening. *Am J Obstet Gynecol* 2000; 182 6:1335-43.

61. Renner RM, Renner A, Schmid S, Hoesli I, Nars P, Holzgreve W, et al. Efficacy of a strategy to prevent neonatal early-onset group B streptococcal (GBS) sepsis. *J Perinat Med* 2006; 34 1:32-8.

62. Riley L, Appollon K, Haider S, Chan-Flynn S, Cohen A, Ecker J, et al. "Real World" compliance with strategies to prevent early-onset group B streptococcal disease. *J Perinatol* 2003; 23 4:272-7.

63. Rottenstreich M, Rotem R, Bergman M, Farkash R, Schimmel MS, Samueloff A, et al. Assessment of maternal GBS colonization and early-onset neonatal disease rate for term deliveries: a decade perspective. *J Perinat Med* 2019; 47 5:528-33.

64. Sakata H. Evaluation of intrapartum antibiotic prophylaxis for the prevention of early-onset group B streptococcal infection. *J Infect Chemother* 2012; 18 6:853-7.

65. Schrag SJ, Zell ER, Lynfield R, Roome A, Arnold KE, Craig AS, et al. A population-based comparison of strategies to prevent early-onset group B streptococcal disease in neonates. *N Engl J Med* 2002; 347 4:233-9.

66. Schuchat A, Roome A, Zell ER, Linardos H, Zywicki S, O'Brien KL. Integrated monitoring of a new group B streptococcal disease prevention program and other perinatal infections. *Matern Child Health J* 2002; 6 2:107-14.

67. Share L, Chaikin S, Pomeranets S, Kiwi R, Jacobs M, Fanaroff AA. Implementation of guidelines for preventing early onset group B streptococcal infection. *Semin Perinatol* 2001; 25 2:107-13.

68. Sutkin G, Krohn MA, Heine RP, Sweet RL. Antibiotic prophylaxis and non-group B streptococcal neonatal sepsis. *Obstet Gynecol* 2005; 105 3:581-6.

69. Towers CV, Briggs GG. Antepartum use of antibiotics and early-onset neonatal sepsis: the next 4 years. *Am J Obstet Gynecol* 2002; 187 2:495-500.

70. Trijbels-Smeulders MA, Kimpen JL, Kollee LA, Bakkers J, Melchers W, Spanjaard L, et al. Serotypes, genotypes, and antibiotic susceptibility profiles of group B streptococci causing neonatal sepsis and meningitis before and after introduction of antibiotic prophylaxis. *Pediatr Infect Dis J* 2006; 25 10:945-8.

71. Trijbels-Smeulders M, de Jonge GA, Pasker-de Jong PC, Gerards LJ, Adriaanse AH, van Lingen RA, et al. Epidemiology of neonatal group B streptococcal disease in the Netherlands before and after introduction of guidelines for prevention. *Arch Dis Child Fetal Neonatal Ed* 2007; 92 4:F271-6.

72. Trollfors B MF, Gudjonsdottir MJ, et al. . Group B streptococcus - a pathogen not restricted to neonates. *IJID Reg* 2022; 4:171-5.

73. Uy IP, D'Angio CT, Menegus M, Guillet R. Changes in early-onset group B beta hemolytic streptococcus disease with changing recommendations for prophylaxis. *J Perinatol* 2002; 22 7:516-22.

74. van den Hoogen A, Gerards LJ, Verboon-Maciolek MA, Fleer A, Krediet TG. Long-term trends in the epidemiology of neonatal sepsis and antibiotic susceptibility of causative agents. *Neonatology* 2010; 97 1:22-8.

75. Van Dyke MK, Phares CR, Lynfield R, Thomas AR, Arnold KE, Craig AS, et al. Evaluation of universal antenatal screening for group B streptococcus. *N Engl J Med* 2009; 360 25:2626-36.

76. Vergani P, Patane L, Colombo C, Borroni C, Giltri G, Ghidini A. Impact of different prevention strategies on neonatal group B streptococcal disease. *Am J Perinatol* 2002; 19 6:341-8.

77. Wicker E, Lander F, Weidemann F, Hufnagel M, Berner R, Krause G. Group B Streptococci: Declining Incidence in Infants in Germany. *Pediatr Infect Dis J* 2019; 38 5:516-9.

78. Youden L, Downing M, Halperin B, Scott H, Smith B, Halperin SA. Group B streptococcal testing during pregnancy: survey of postpartum women and audit of current prenatal screening practices. *J Obstet Gynaecol Can* 2005; 27 11:1006-12.
